# Supplementary material for: Production, Passaging Stability, and Histological Analysis of Madin–Darby Canine Kidney Cells Cultured in a Low-Serum Medium
Source: Vaccines (Basel). 2024 Aug 30;12(9):991. doi: 10.3390/vaccines12090991 (PMC11435615; doi:10.3390/vaccines12090991)
Supplement: Supplementary file 1 [file vaccines-12-00991-s001.zip › Supplementary File S1:Protocol of RNA-seq.pdf]

Protocol of RNA-seq

Section 1: Sequencing

1. Sample detection

The detection of RNA samples mainly includes four methods：

（1）Nanodrop is used to measure the purity of RNA (OD260/OD280 ratio).

（2）Qubit 3.0 accurately quantifies RNA concentration.

（3）Agarose gel electrophoresis is used to analyze the purity and integrity of RNA.

（4）The Agilent 2100 is utilized for precise detection of RNA integrity.

2. Library Construction

After the RNA samples successfully pass the qualification test, the entire library preparation process is accomplished through a series of steps, including mRNA enrichment, fragmentation, cDNA synthesis of one strand, double-strand synthesis, end repair, A-tailing, adapter ligation, purification and PCR amplification.

3. Evaluation of Library Quality

After the construction of the library, Qubit 3.0 is utilized for initial quantification of the library. Subsequently, Agilent 2100 is employed to detect the insert fragments within the library, followed by Q-PCR method for precise determination of effective concentration to ensure high-quality libraries.

4. Sequencing

After passing quality inspection, the library will be subjected to Illumina NovaSeq 6000 PE150 sequencing based on its effective concentration and data output requirements.

Section 2: Data analysis.

1. Data quality control

Fastp is used to perform quality control on the raw data obtained from sequencing, primarily removing sequencing adapters and low-quality bases. Then, SortMeRNA is employed with the default rRNA database to eliminate rRNA sequences from the samples, resulting in clean data.

2. Sequence alignment

Using STAR, the Clean Data obtained after data quality control is aligned to the reference genome (ROS_Cfam_1.0, the release of Ensembl 109), and then Qualimap2 is used to individually assess the alignment results for each sample.

3. Gene expression

Utilize the STAR algorithm to quantify the abundance of reads aligned to annotated genes in the reference genome, and subsequently standardize gene counts using TPM (Transcripts Per Million) as a metric for assessing gene expression.

4. Differential expression analysis

Differential expression analysis was performed using Deseq2 to compare gene expression between groups, and differentially expressed genes were filtered based on criteria of $|\log2(\text{FoldChange})| \geqslant 1$ and Padjust $\leqslant 0.05$.

5. Gene enrichment analysis

By utilizing clusterProfiler for KEGG enrichment analysis of differentially expressed genes, our objective is to identify gene functional categories that exhibit significant associations with the biological question under investigation within the database.
