# Supplementary material for: Production, Passaging Stability, and Histological Analysis of Madin–Darby Canine Kidney Cells Cultured in a Low-Serum Medium
Source: Vaccines (Basel). 2024 Aug 30;12(9):991. doi: 10.3390/vaccines12090991 (PMC11435615; doi:10.3390/vaccines12090991)
Supplement: Supplementary file 1 [file vaccines-12-00991-s001.zip › Supplementary File S2:Protocol of proteomic analysis.pdf]

Protocol of proteomic analysis

Section S1：Sample detection

1. Processing of the samples

The samples were treated with reaction solution (1% SDC/100 mM Tris-HCL pH=8.5/10 mM TCEP/40 mM CAA) and incubated at 60°C for 1 hour to achieve protein denaturation, reduction, and alkylation in a one-step method. After quantifying the total protein using the BCA assay, an equal volume of ultrapure water was added to each sample followed by dilution with an equal amount of protein. Enzymatic digestion was performed by adding trypsin at an enzyme-to-protein ratio of 1:50, and the mixture was incubated overnight with shaking at 37°C. On the subsequent day, TFA was added to terminate the digestion process, followed by centrifugation at 16,000 g to collect the supernatant. Desalting was carried out using a C18 solid-phase extraction membrane (3M Empore), followed by drying and storage at -20°C.

2. Mass spectrometry detection

The mass spectrometry data was acquired using an Orbitrap Exploris 480 mass spectrometer coupled with an EASY-nLC 1200 liquid chromatography system. The peptide samples were dissolved in the loading buffer and injected into the analytical column (75 μm * 25 cm, C18, 1.9 μm, 100 Å) through an autosampler for separation. A gradient of two mobile phases (mobile phase A: 0.1% formic acid and mobile phase B: 0.1% formic acid, 80% ACN) was established for analysis. The flow rate of the liquid phase was set at a constant value of 300 nL/min throughout the experiment. Mass spectrometry data were collected in DDA mode, where each scan cycle consisted of one MS full scan (R = resolving power set to be equal to or greater than 60 K, AGC = automatic gain control set to reach a target value of approximately three times higher than normal capacity i.e., AGC =300%, max IT = maximum injection time limited to a duration not exceeding twenty milliseconds per scan cycle; scan range=350 -1500 m/z), followed by subsequent twenty MS/MS scans (R=resolving power adjusted to be around fifteen thousand units, AGC=automatic gain control maintained at hundred percent capacity during acquisition process; max IT=maximum injection time automatically determined based on signal intensity; cycle time=set at two seconds).

HCD collision energy was fixed at thirty units while quadrupole isolation window width remained constant at one point six Dalton throughout all experiments conducted under this study design condition. Dynamic exclusion feature enabled ion reacquisition after elapse of thirty-five seconds from previous detection event.

Section S2：Data analysis

1. Database retrieval

The mass spectrometry data was analyzed using the MaxQuant software, employing the Andromeda database search algorithm. The protein reference database utilized for the search consisted of Canis lupus proteome from Uniprot (20231011), encompassing 134832 protein sequences. The primary search parameters included project type selection as LFQ; variable modifications selection as Oxidation (M) and Acetyl (Protein N-term); fixed modification selection as Carbamidomethyl (C); enzyme digestion selected as Trypsin/P; primary mass tolerance set to 20 ppm in initial search and 4.5 ppm in main search; secondary mass tolerance set to 20 ppm; with the "match between runs" option enabled. Subsequently, a stringent filtering approach was applied at a 1% FDR threshold at both protein and peptide levels, eliminating decoy proteins, contaminant proteins, and proteins with only one modified peptide sequence. The remaining identification information was retained for subsequent analysis.

2. Data quality control

Utilize the R programming language to compute diverse indicators and generate corresponding graphs, encompassing distributions of peptide length, peptide count, missed cleavage sites, mass deviation, common contaminant proteins, as well as missing data. Assess the precision and consistency of sample detection data.

3. Standardization and missing data imputation

Normalize the protein quantification results using log2 and perform missing value imputation with Perseus imputation.

4. Differential protein expression analysis

The differential expression analysis of missing data was performed using R, where the t-test was employed to calculate P-values and the FDR method was applied for

multiple testing correction. The criteria for selecting differentially expressed proteins were defined as |log2(FoldChange)|≥1 and Padjust≤0.05.

5. Enrichment analysis

Using clusterProfiler, we performed GO and KEGG enrichment analysis on differentially expressed proteins to identify protein functional categories that are significantly associated with the biological question under investigation in the database.
