# Supplementary material for: Production, Passaging Stability, and Histological Analysis of Madin–Darby Canine Kidney Cells Cultured in a Low-Serum Medium
Source: Vaccines (Basel). 2024 Aug 30;12(9):991. doi: 10.3390/vaccines12090991 (PMC11435615; doi:10.3390/vaccines12090991)
Supplement: Supplementary file 1 [file vaccines-12-00991-s001.zip › Supplementary File S3/proteome/4.Enrichment/gsea/5-infected_vs_5-uninfected/5-infected_vs_5-uninfected_KEGG_GSEA_ridgeplot.pdf]

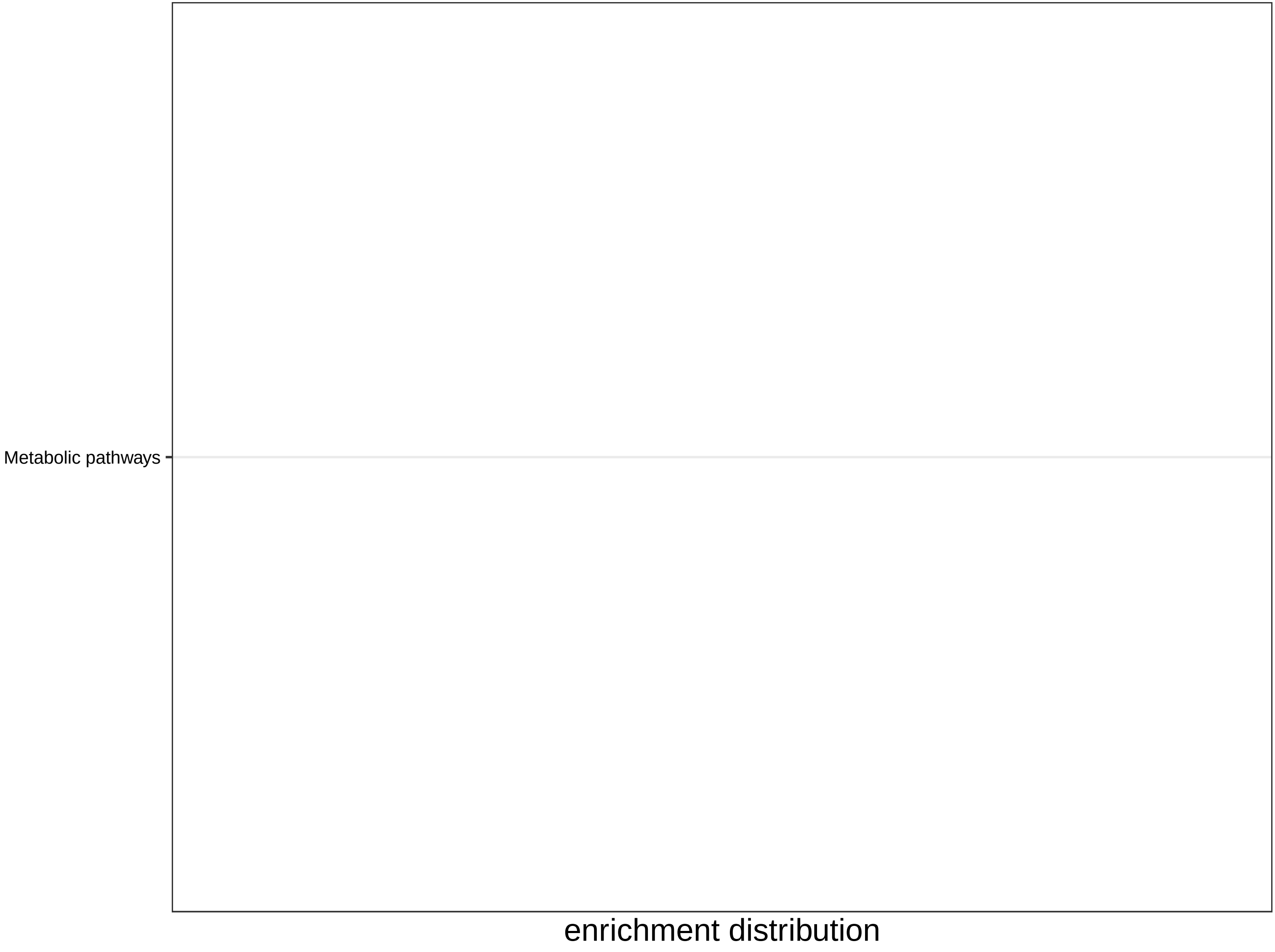

Metabolic pathways
enrichment distribution
