# Supplementary material for: Production, Passaging Stability, and Histological Analysis of Madin–Darby Canine Kidney Cells Cultured in a Low-Serum Medium
Source: Vaccines (Basel). 2024 Aug 30;12(9):991. doi: 10.3390/vaccines12090991 (PMC11435615; doi:10.3390/vaccines12090991)
Supplement: Supplementary file 1 [file vaccines-12-00991-s001.zip › Supplementary File S3/transcriptome/1.QualityControl/stats/ly_multiqc_fastp.html]

MultiQC Report


# Toggle navigation v1.19

Loading report..

- General Stats
- fastp
  - Filtered Reads
  - Insert Sizes
  - Sequence Quality
  - GC Content
  - N content
- Software Versions

Toolbox

### MultiQC Toolbox

#### Apply Highlight Samples

+

Regex mode off
help
 Clear

#### Apply Rename Samples

+

Click here for bulk input.

Paste two columns of a tab-delimited table here (eg. from Excel).

First column should be the old name, second column the new name.

Add

Regex mode off
help
 Clear

#### Apply Show / Hide Samples

Hide matching samples

Show only matching samples

+

Regex mode off
help
 Clear

#### Export Plots

- Images
- Data

px

px

Aspect ratio

PNG
JPEG
SVG

Plot scaling

X

Download the raw data used to create the plots in this report below:

Format:

Tab-separated
Comma-separated
JSON

Note that additional data was saved in `ly_multiqc_fastp_data` when this report was generated.

---

##### Choose Plots

 All
 None

---


   Download Plot Images

If you use plots from MultiQC in a publication or presentation, please cite:

> **MultiQC: Summarize analysis results for multiple tools and samples in a single report**  
> *Philip Ewels, Måns Magnusson, Sverker Lundin and Max Käller*  
> Bioinformatics (2016)  
> doi: 10.1093/bioinformatics/btw354  
> PMID: 27312411

#### Save Settings

You can save the toolbox settings for this report to the browser.

 Save


---

#### Load Settings

Choose a saved report profile from the dropdown box below:

[ select ]

Load
 Delete
 Set default
 Clear default

#### Tool Citations

Please remember to cite the tools that you use in your analysis.

To help with this, you can download publication details of the tools mentioned in this report:

List of DOIs

BibTeX file

#### About MultiQC

This report was generated using MultiQC, version 1.19

You can see a YouTube video describing how to use MultiQC reports here:
https://youtu.be/qPbIlO\_KWN0

For more information about MultiQC, including other videos and
extensive documentation, please visit http://multiqc.info

You can report bugs, suggest improvements and find the source code for MultiQC on GitHub:
https://github.com/ewels/MultiQC

MultiQC is published in Bioinformatics:

> **MultiQC: Summarize analysis results for multiple tools and samples in a single report**  
> *Philip Ewels, Måns Magnusson, Sverker Lundin and Max Käller*  
> Bioinformatics (2016)  
> doi: 10.1093/bioinformatics/btw354  
> PMID: 27312411

---

MultiQC is developed by:

# 

A modular tool to aggregate results from bioinformatics analyses across many samples into a single report.

#### JavaScript Disabled

MultiQC reports use JavaScript for plots and toolbox functions. It looks like
you have JavaScript disabled in your web browser. Please note that many of the report
functions will not work as intended.

Loading report..

---

×
don't show again

**Welcome!** Not sure where to start?  
Watch a tutorial video
  *(6:06)*

## General Statistics

 Copy table

 Configure Columns

 Sort by highlight

 Plot
Showing 12/12 rows and 5/6 columns.

| Sample Name | % > Q30 | Mb Q30 bases | M Reads After Filtering | GC content | % PF | % Adapter |
| --- | --- | --- | --- | --- | --- | --- |
| 3-1-infected | 92.0% | 7474.4 | 54.2 | 46.1% | 99.3% | 0.6% |
| 3-1-uninfected | 92.9% | 12611.0 | 90.6 | 49.6% | 99.1% | 0.9% |
| 3-2-infected | 91.9% | 8517.1 | 61.8 | 46.2% | 99.2% | 0.5% |
| 3-2-uninfected | 92.4% | 12309.8 | 88.9 | 49.2% | 99.1% | 0.9% |
| 3-3-infected | 92.2% | 7735.0 | 56.0 | 46.2% | 99.3% | 0.5% |
| 3-3-uninfected | 93.0% | 15908.0 | 114.1 | 50.6% | 99.1% | 1.0% |
| 5-1-infected | 91.9% | 7126.2 | 51.7 | 46.4% | 99.3% | 0.4% |
| 5-1-uninfected | 92.7% | 14057.9 | 101.3 | 49.0% | 99.1% | 0.9% |
| 5-2-infected | 91.8% | 8455.4 | 61.4 | 46.3% | 99.3% | 0.4% |
| 5-2-uninfected | 92.6% | 17229.3 | 124.2 | 47.9% | 99.1% | 0.9% |
| 5-3-infected | 92.1% | 9488.3 | 68.7 | 46.3% | 99.4% | 0.4% |
| 5-3-uninfected | 92.8% | 14766.7 | 106.3 | 48.0% | 99.2% | 0.9% |

×

#### General Statistics: Columns

Uncheck the tick box to hide columns. Click and drag the handle on the left to change order. Table ID: `general_stats_table`

Show All
Show None

| Sort | Visible | Group | Column | Description | ID | Scale |
| --- | --- | --- | --- | --- | --- | --- |
| || |  | fastp | % > Q30 | Percentage of reads > Q30 after filtering | `after_filtering_q30_rate` | None |
| || |  | fastp | Mb Q30 bases | Bases > Q30 after filtering (millions) | `after_filtering_q30_bases` | base\_count |
| || |  | fastp | M Reads After Filtering | Total reads after filtering (millions) | `filtering_result_passed_filter_reads` | read\_count |
| || |  | fastp | GC content | GC content after filtering | `after_filtering_gc_content` | None |
| || |  | fastp | % PF | Percent reads passing filter | `pct_surviving` | None |
| || |  | fastp | % Adapter | Percentage adapter-trimmed reads | `pct_adapter` | None |

Close

## fastp

*Version:* `0.23.4`

fastp An ultra-fast all-in-one FASTQ preprocessor (QC, adapters, trimming, filtering, splitting...).*DOI: 10.1093/bioinformatics/bty560.*

### Filtered Reads

Filtering statistics of sampled reads.

Number of Reads
Percentages

loading..

---

### Insert Sizes

Insert size estimation of sampled reads.

loading..

---

### Sequence Quality

Average sequencing quality over each base of all reads.

Read 1: Before filtering
Read 1: After filtering
Read 2: Before filtering
Read 2: After filtering

loading..

---

### GC Content

Average GC content over each base of all reads.

Read 1: Before filtering
Read 1: After filtering
Read 2: Before filtering
Read 2: After filtering

loading..

---

### N content

Average N content over each base of all reads.

Read 1: Before filtering
Read 1: After filtering
Read 2: Before filtering
Read 2: After filtering

loading..

---

## Software Versions

Software Versions lists versions of software tools extracted from file contents.

Copy table

| Software | Version |
| --- | --- |
| fastp | `0.23.4` |

**MultiQC v1.19**
- Written by Phil Ewels,
available on GitHub.

This report uses HighCharts,
jQuery,
jQuery UI,
Bootstrap,
FileSaver.js and
clipboard.js.

×

### Plot Table Data

Select Column

Select Column

Please select two table columns.

Close

×

### Regex Help

Toolbox search strings can behave as regular expressions (regexes). Click a button below to see an example of it in action. Try modifying them yourself in the text box.

`^` (start of string)
`$` (end of string)
`[]` (character choice)
`\d` (shorthand for `[0-9]`)
`\w` (shorthand for `[0-9a-zA-Z_]`)
`.` (any character)
`\.` (literal full stop)
`()` `|` (group / separator)
`*` (prev char 0 or more)
`+` (prev char 1 or more)
`?` (prev char 0 or 1)
`{}` (char num times)
`{,}` (count range)

```
samp_1
samp_1_edited
samp_2
samp_2_edited
samp_3
samp_3_edited
prepended_samp_1
tmp_samp_1_edited
tmpp_samp_1_edited
tmppp_samp_1_edited
#samp_1_edited.tmp
samp_11
samp_11111
```

See regex101.com for a more heavy duty testing suite.

Close
