# Supplementary figures and images for: Production, Passaging Stability, and Histological Analysis of Madin–Darby Canine Kidney Cells Cultured in a Low-Serum Medium (part 2 of 2)
Source: Vaccines (Basel). 2024 Aug 30;12(9):991. doi: 10.3390/vaccines12090991 (PMC11435615; doi:10.3390/vaccines12090991)

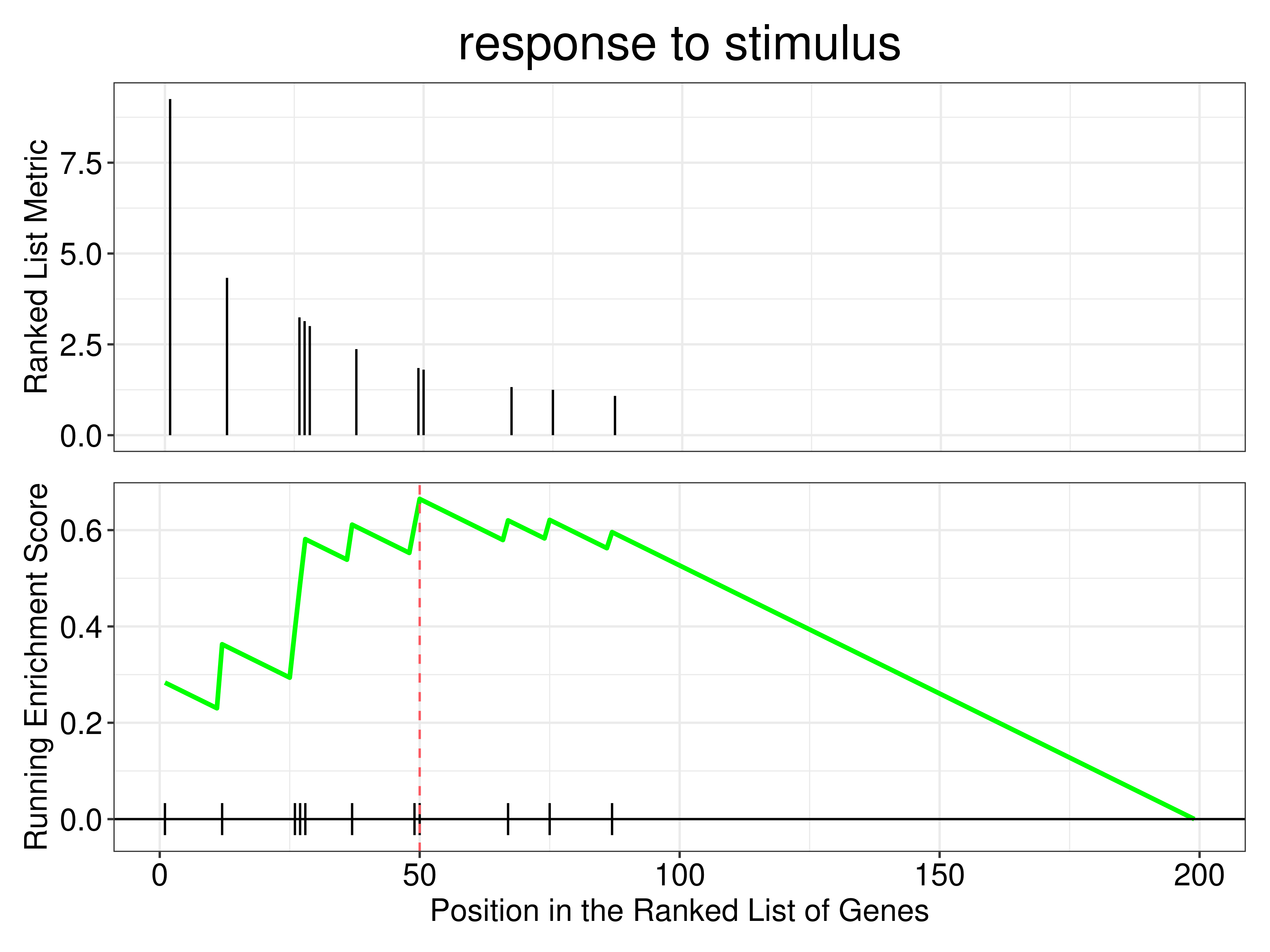

Supplement: Supplementary file 1 [file vaccines-12-00991-s001.zip › Supplementary File S3/proteome/4.Enrichment/gsea/5-infected_vs_5-uninfected/5-infected_vs_5-uninfected_GO_BP_GSEA_gseaplot.png]

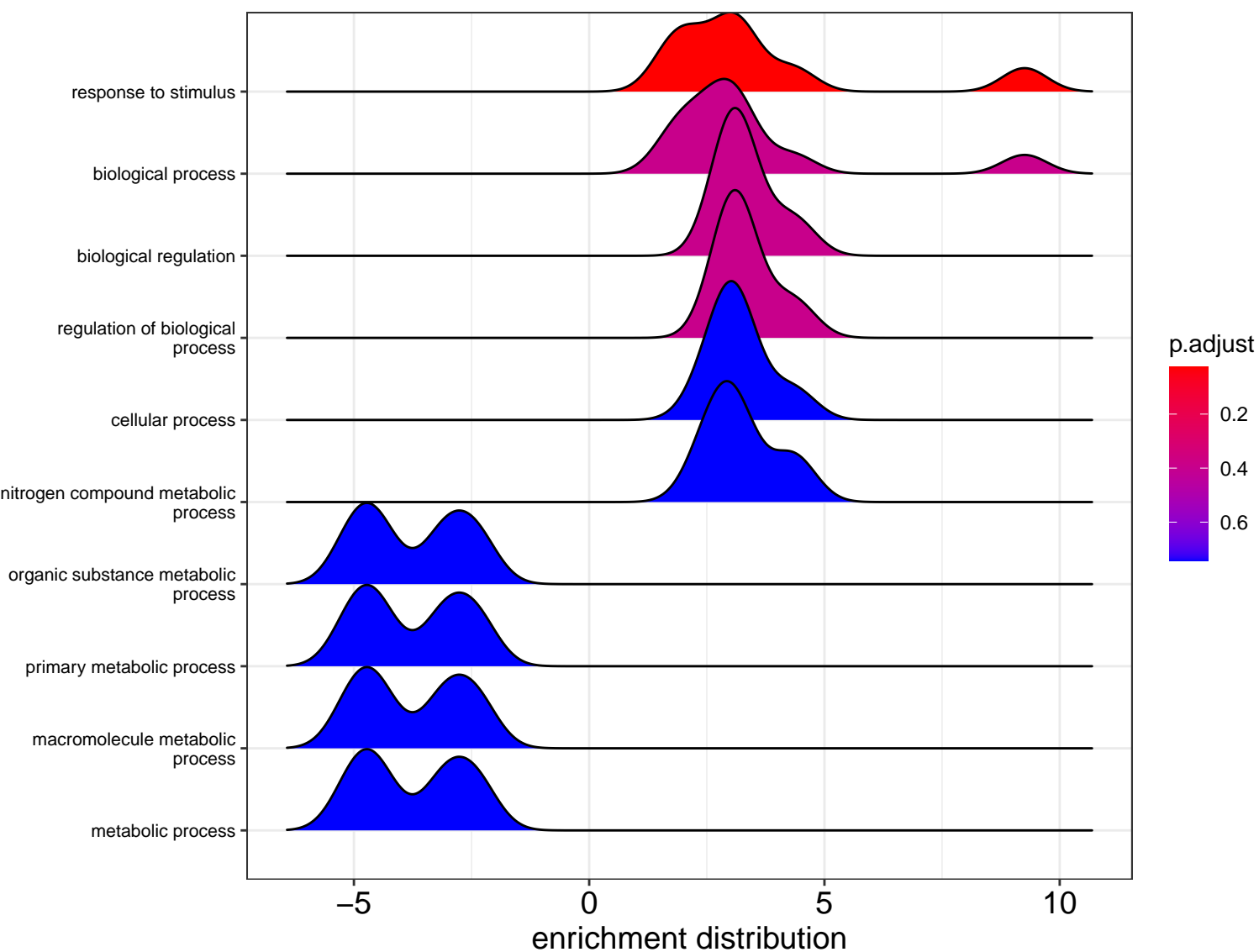

Supplement: Supplementary file 1 [file vaccines-12-00991-s001.zip › Supplementary File S3/proteome/4.Enrichment/gsea/5-infected_vs_5-uninfected/5-infected_vs_5-uninfected_GO_BP_GSEA_ridgeplot.pdf]

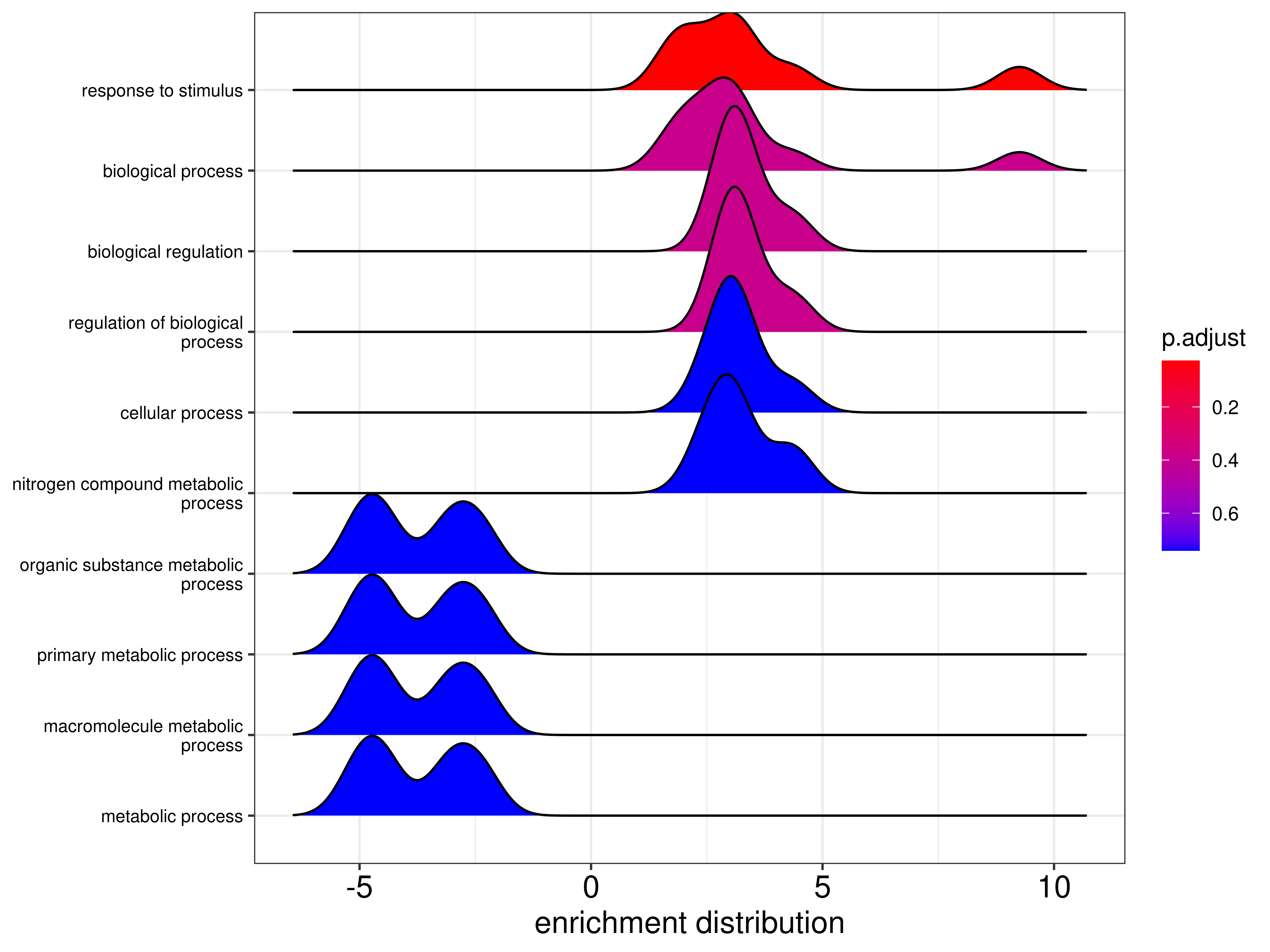

Supplement: Supplementary file 1 [file vaccines-12-00991-s001.zip › Supplementary File S3/proteome/4.Enrichment/gsea/5-infected_vs_5-uninfected/5-infected_vs_5-uninfected_GO_BP_GSEA_ridgeplot.png]

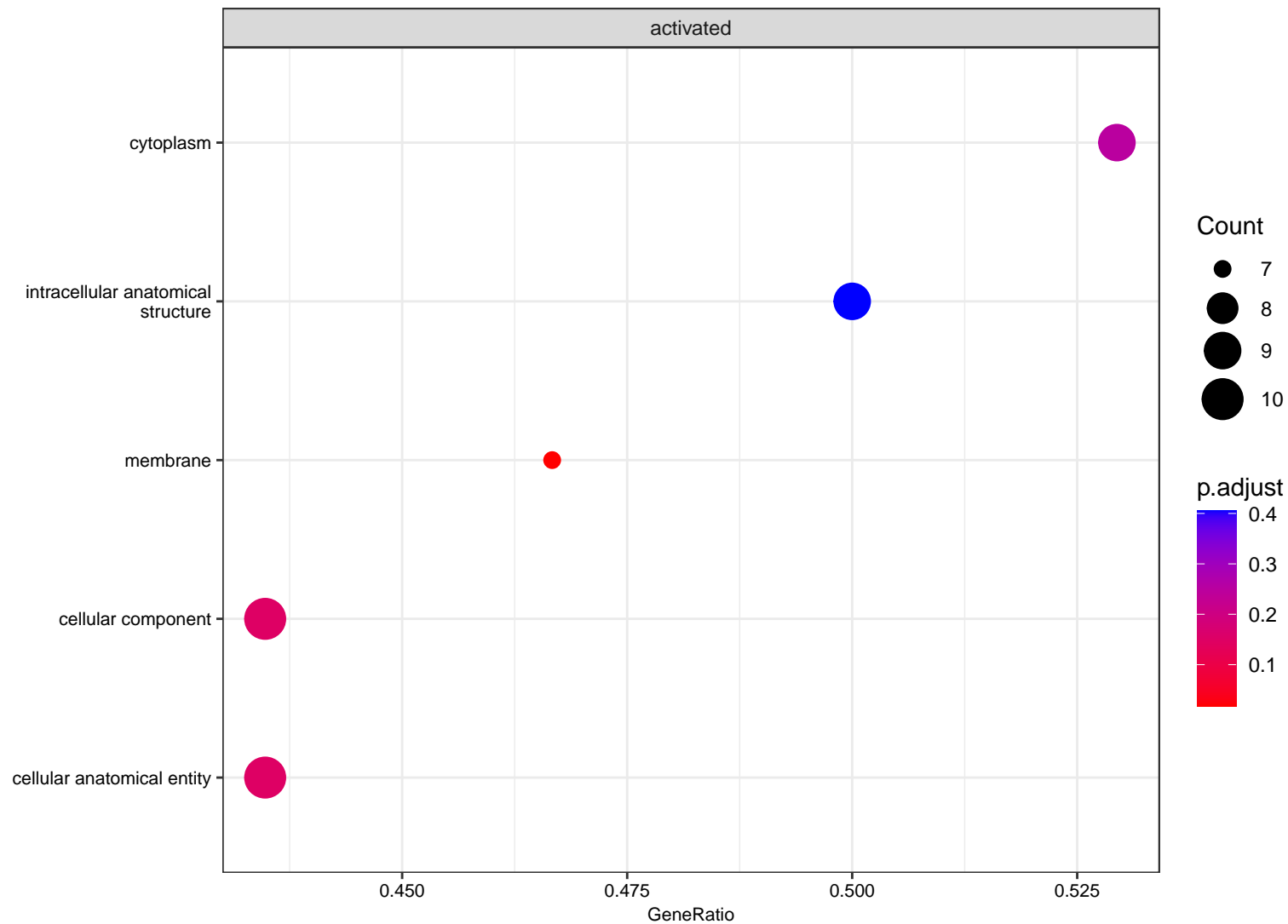

Supplement: Supplementary file 1 [file vaccines-12-00991-s001.zip › Supplementary File S3/proteome/4.Enrichment/gsea/5-infected_vs_5-uninfected/5-infected_vs_5-uninfected_GO_CC_GSEA_dotplot.pdf]

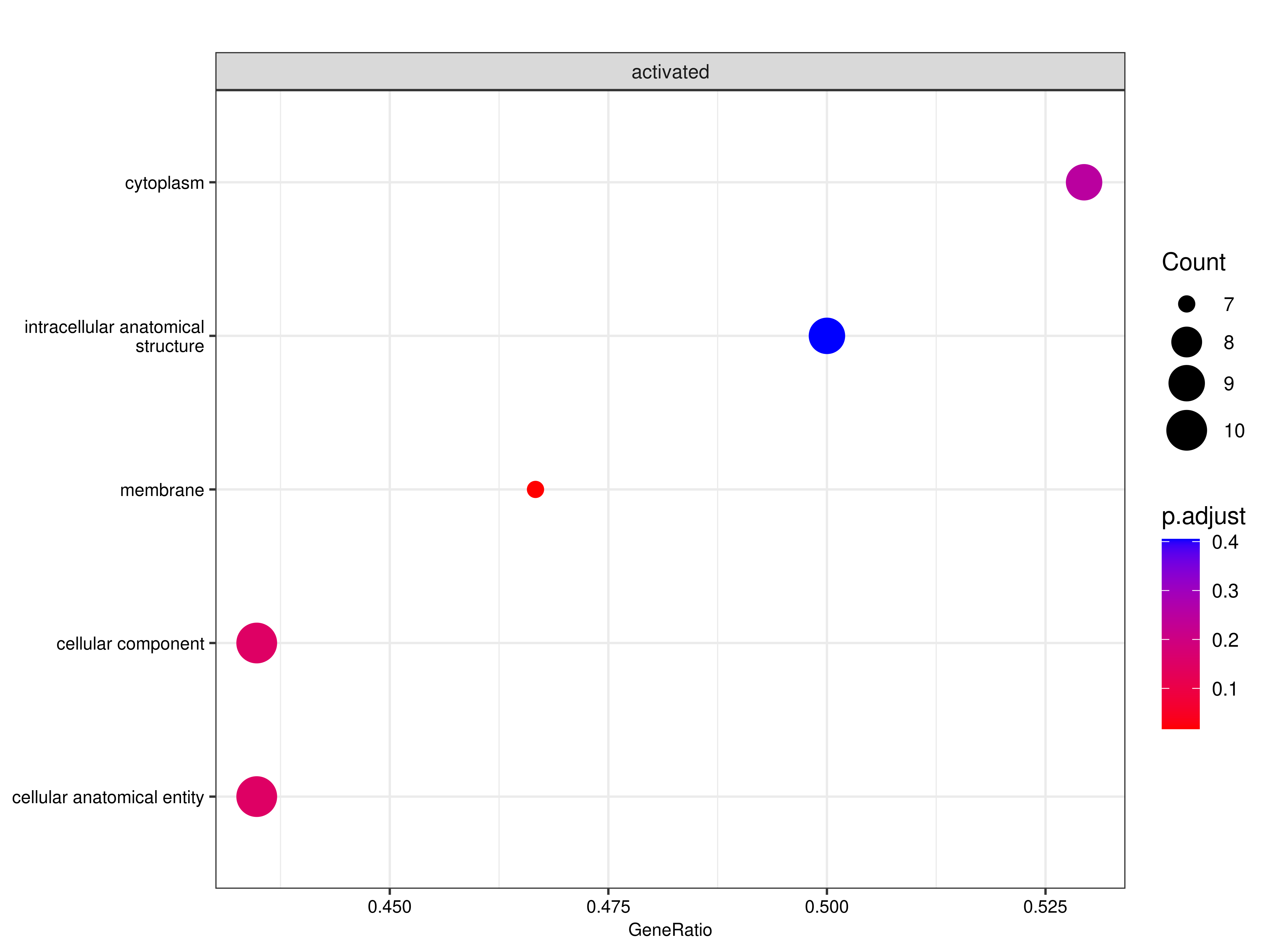

Supplement: Supplementary file 1 [file vaccines-12-00991-s001.zip › Supplementary File S3/proteome/4.Enrichment/gsea/5-infected_vs_5-uninfected/5-infected_vs_5-uninfected_GO_CC_GSEA_dotplot.png]

# membrane

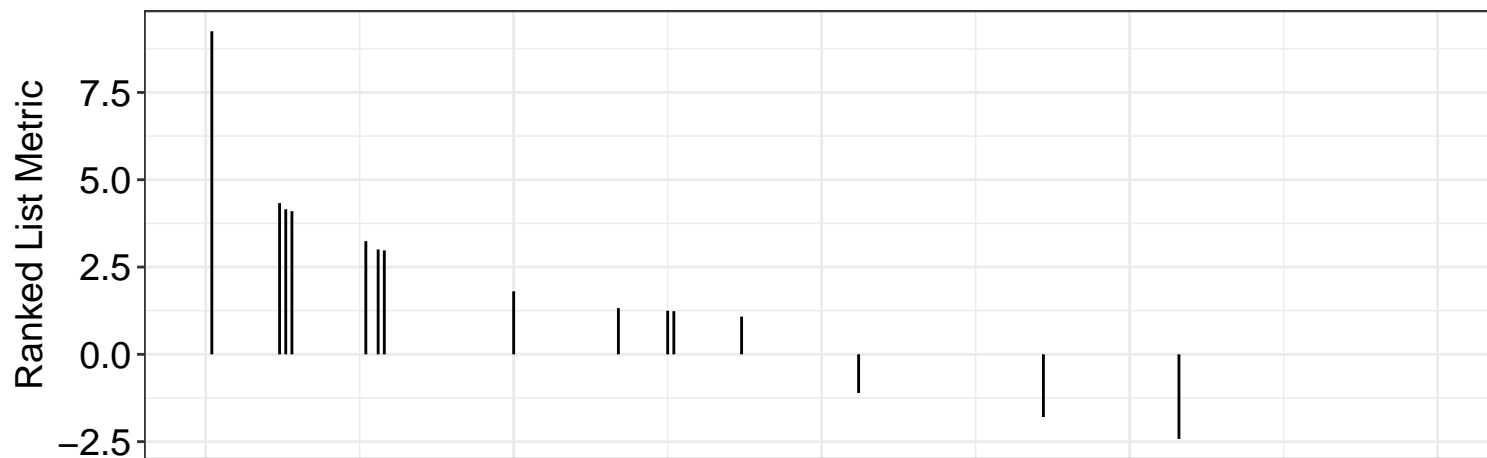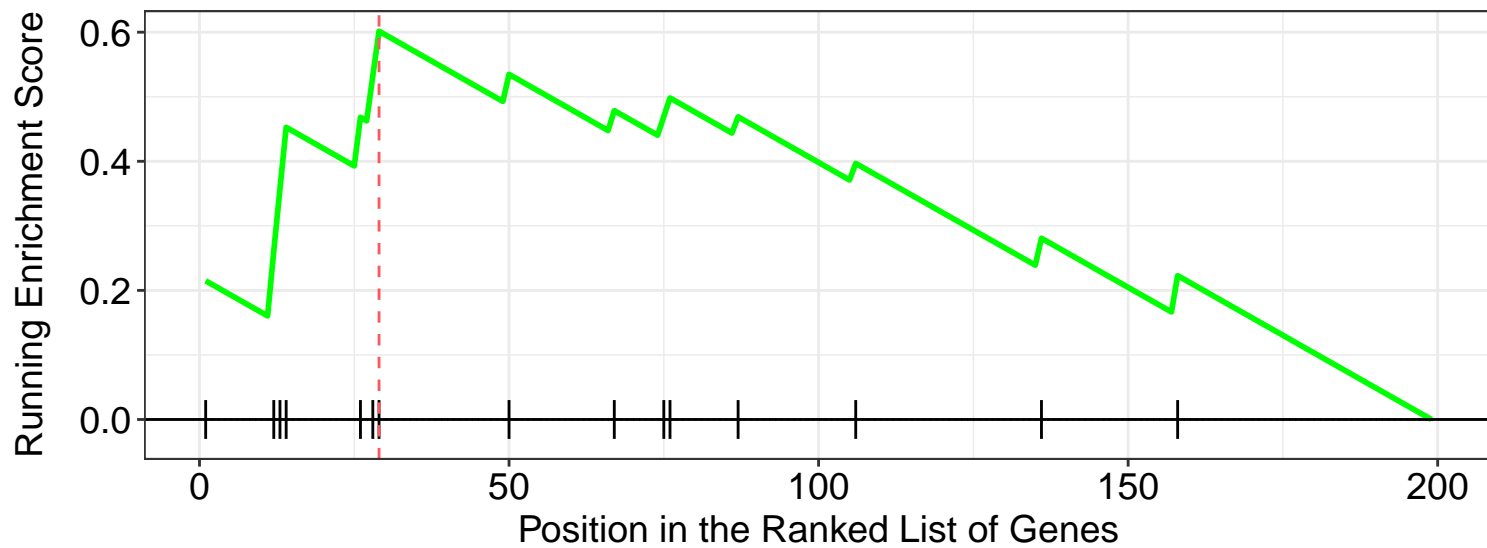

Supplement: Supplementary file 1 [file vaccines-12-00991-s001.zip › Supplementary File S3/proteome/4.Enrichment/gsea/5-infected_vs_5-uninfected/5-infected_vs_5-uninfected_GO_CC_GSEA_gseaplot.pdf]

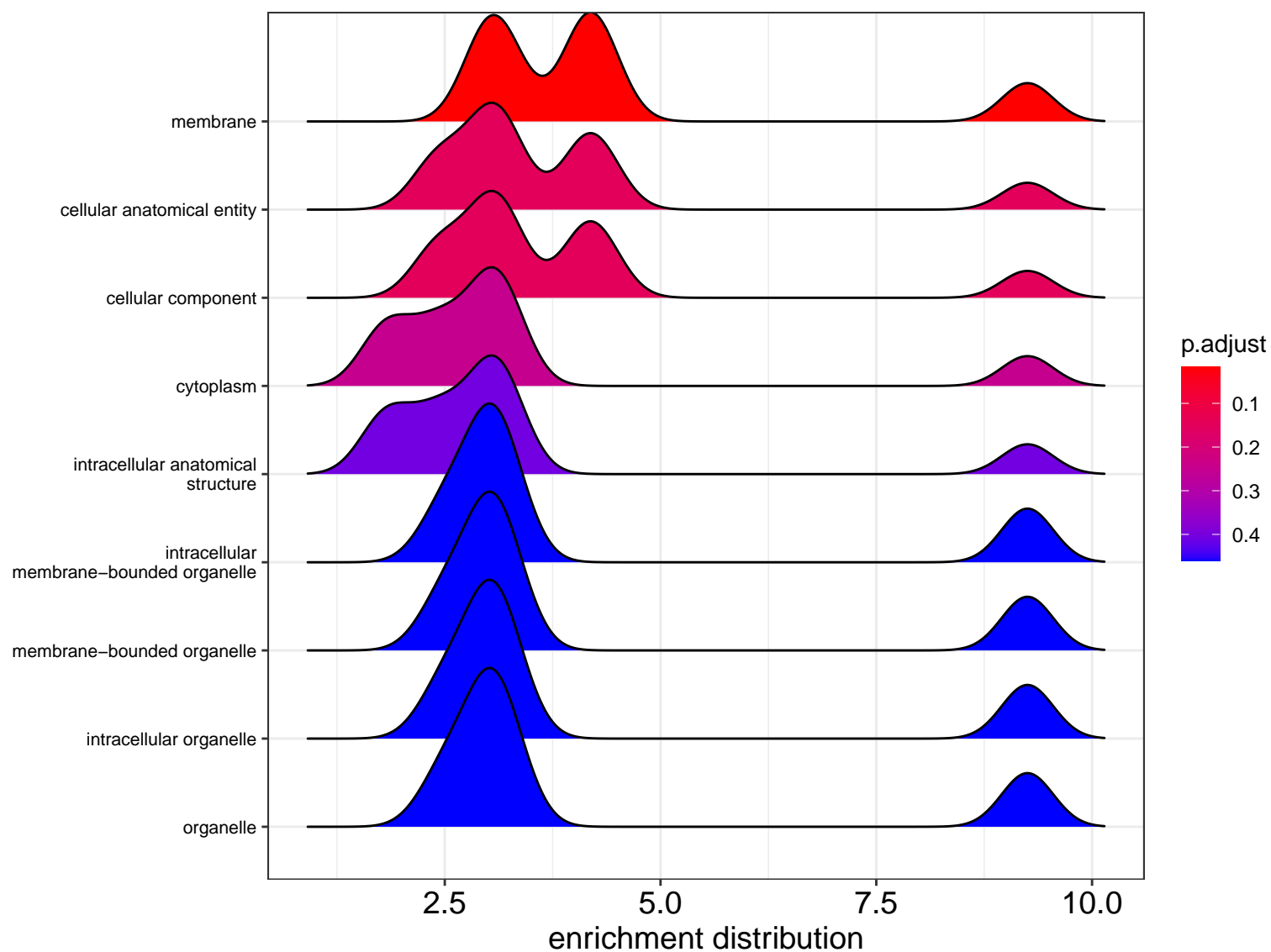

Supplement: Supplementary file 1 [file vaccines-12-00991-s001.zip › Supplementary File S3/proteome/4.Enrichment/gsea/5-infected_vs_5-uninfected/5-infected_vs_5-uninfected_GO_CC_GSEA_ridgeplot.pdf]

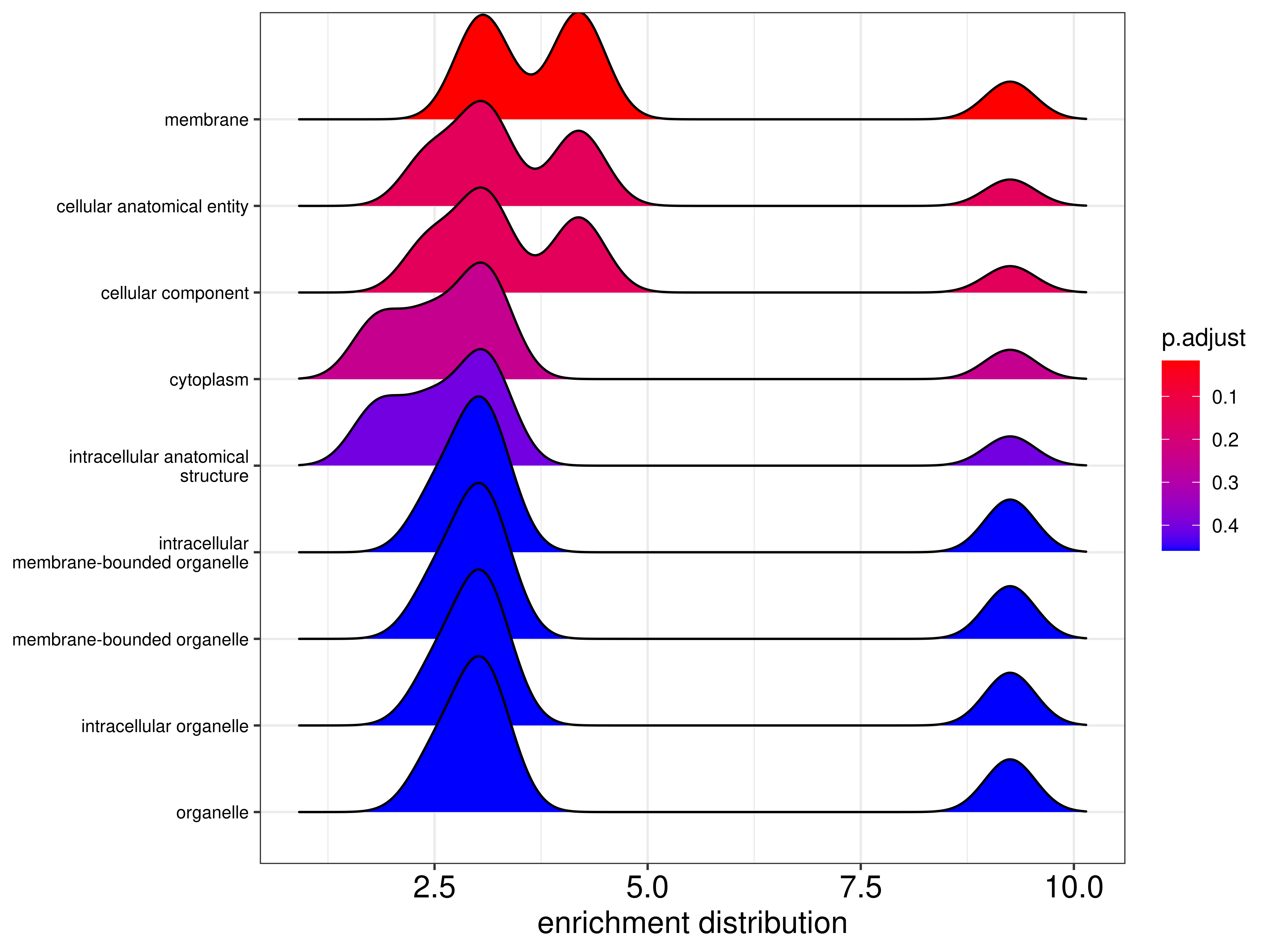

Supplement: Supplementary file 1 [file vaccines-12-00991-s001.zip › Supplementary File S3/proteome/4.Enrichment/gsea/5-infected_vs_5-uninfected/5-infected_vs_5-uninfected_GO_CC_GSEA_ridgeplot.png]

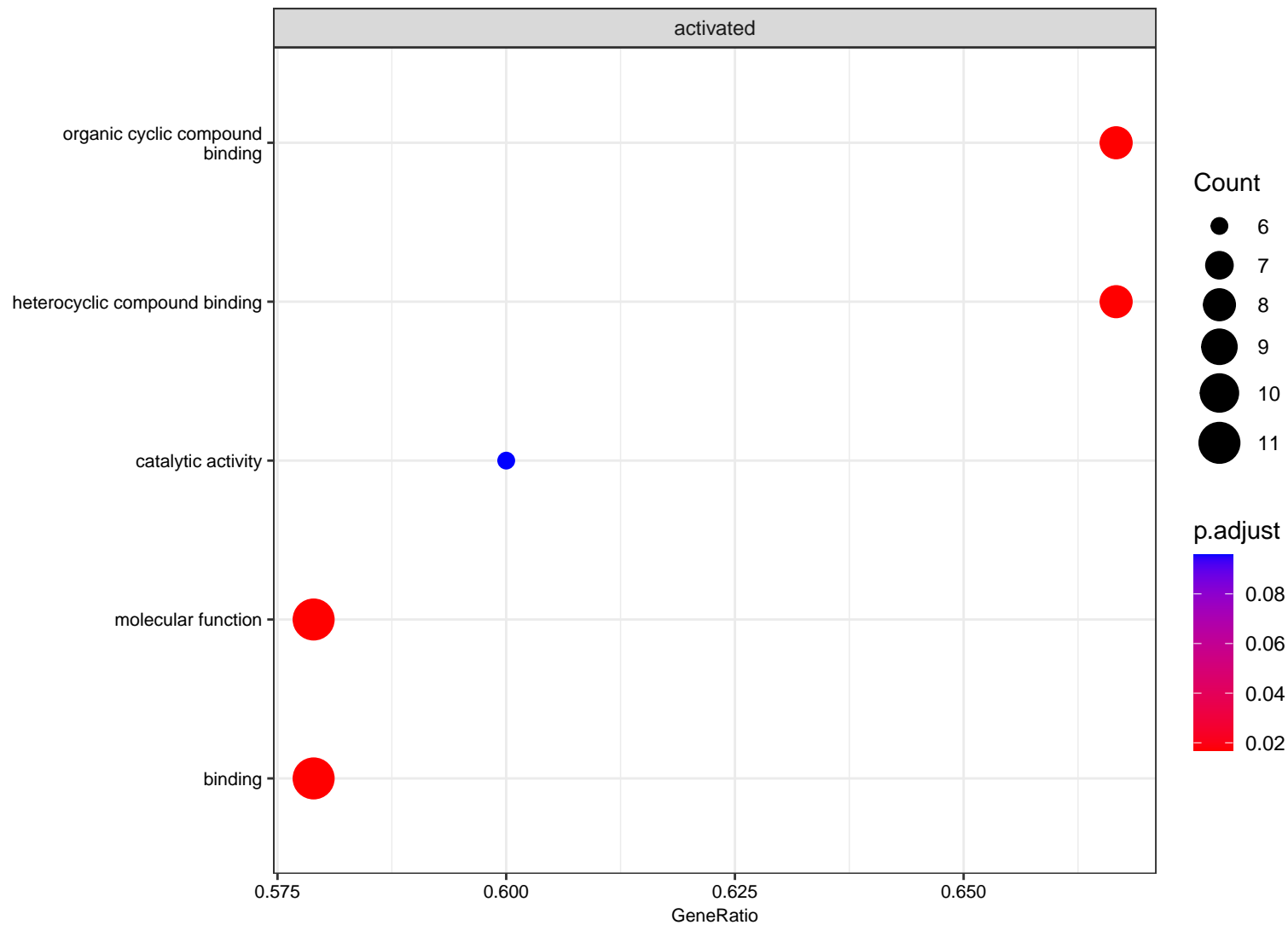

Supplement: Supplementary file 1 [file vaccines-12-00991-s001.zip › Supplementary File S3/proteome/4.Enrichment/gsea/5-infected_vs_5-uninfected/5-infected_vs_5-uninfected_GO_MF_GSEA_dotplot.pdf]

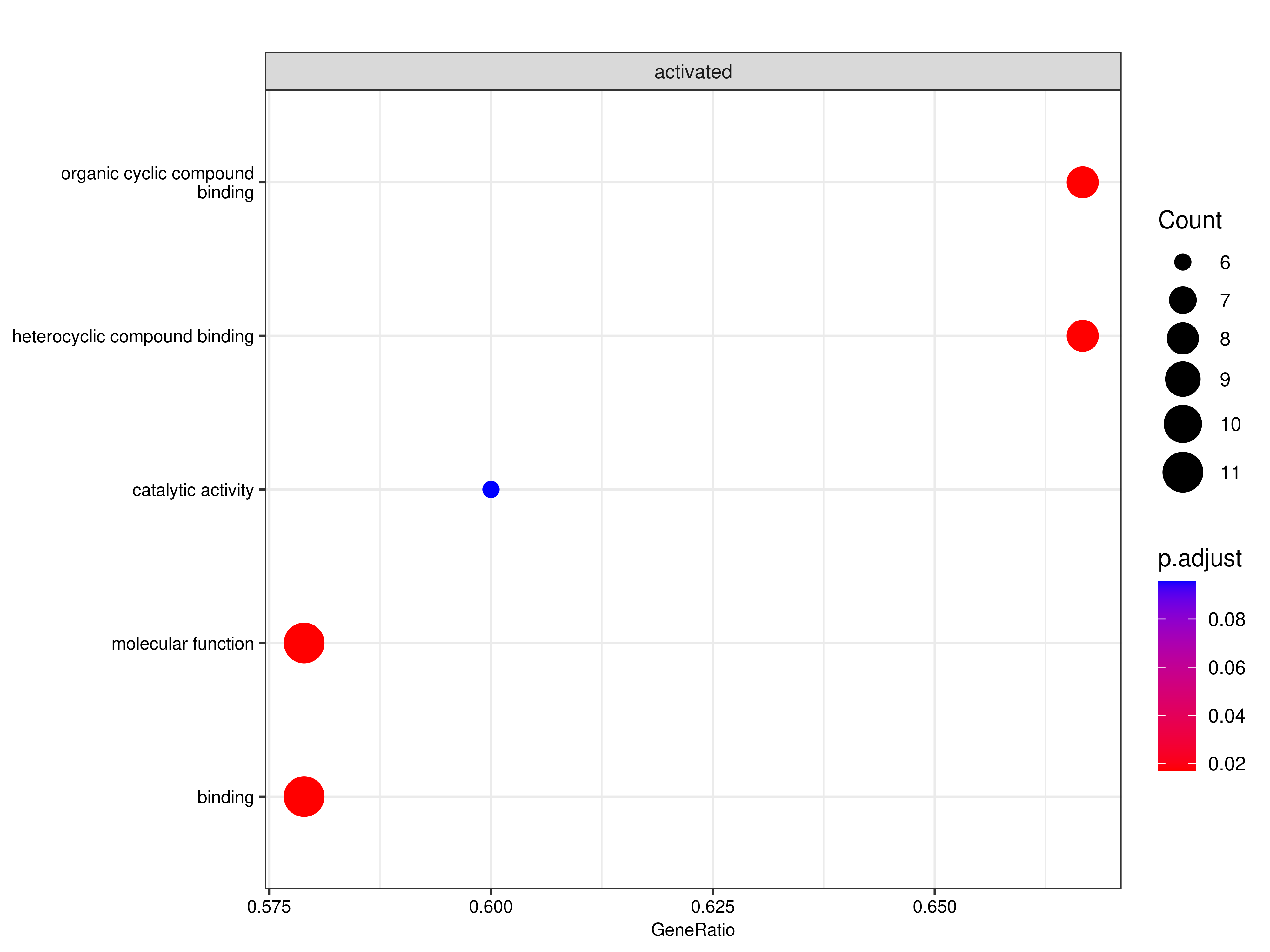

Supplement: Supplementary file 1 [file vaccines-12-00991-s001.zip › Supplementary File S3/proteome/4.Enrichment/gsea/5-infected_vs_5-uninfected/5-infected_vs_5-uninfected_GO_MF_GSEA_dotplot.png]

# molecular\_function

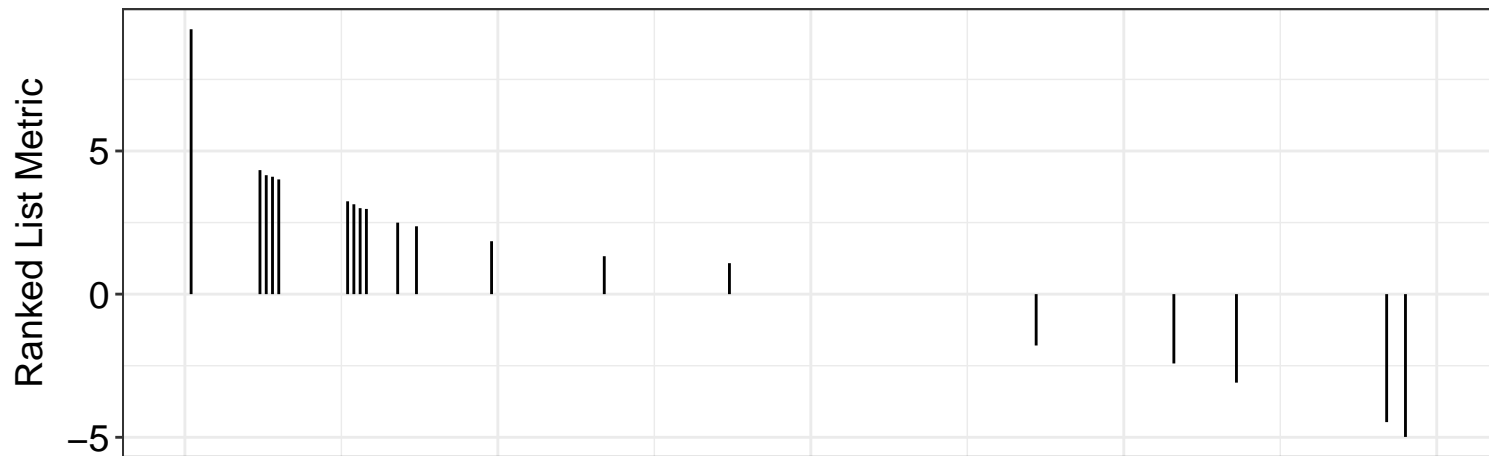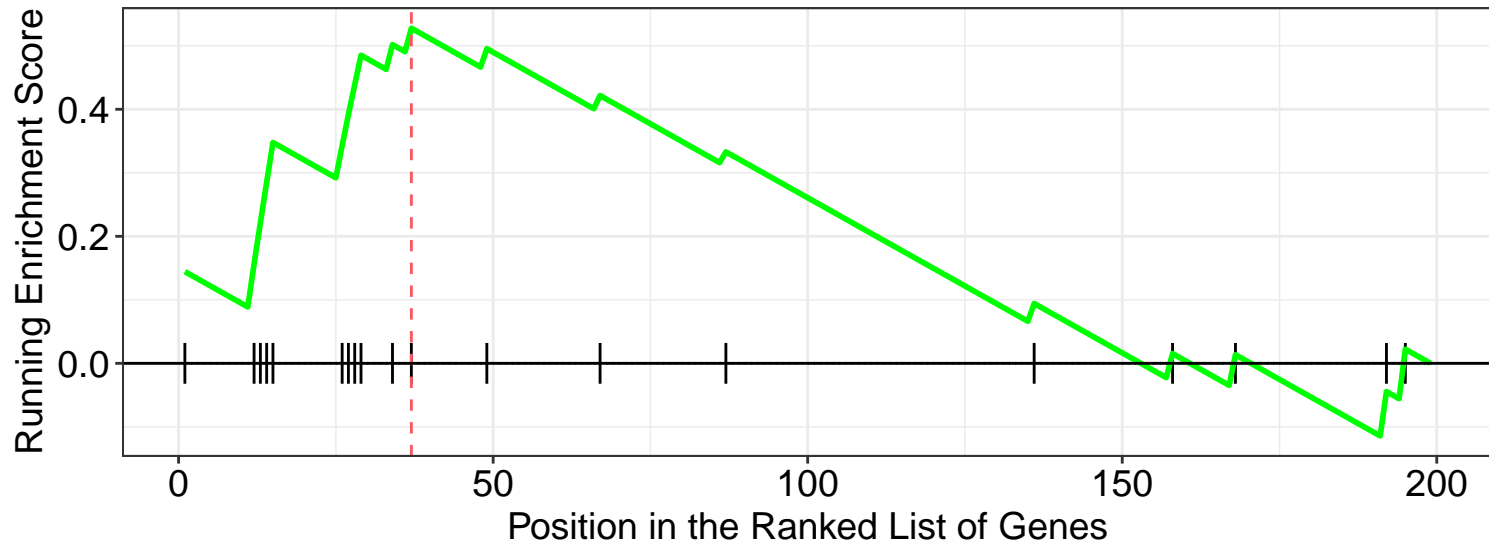

Supplement: Supplementary file 1 [file vaccines-12-00991-s001.zip › Supplementary File S3/proteome/4.Enrichment/gsea/5-infected_vs_5-uninfected/5-infected_vs_5-uninfected_GO_MF_GSEA_gseaplot.pdf]

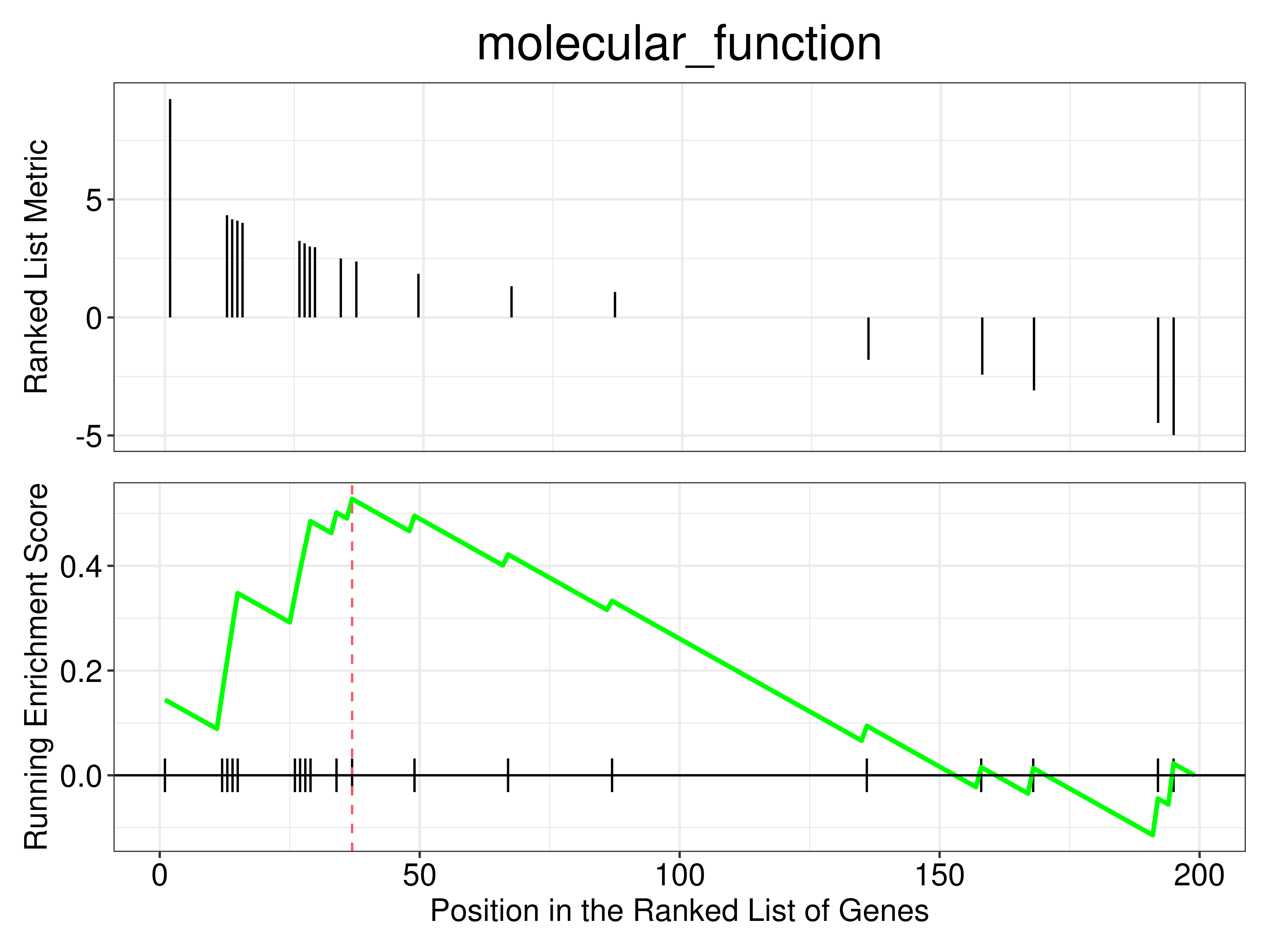

Supplement: Supplementary file 1 [file vaccines-12-00991-s001.zip › Supplementary File S3/proteome/4.Enrichment/gsea/5-infected_vs_5-uninfected/5-infected_vs_5-uninfected_GO_MF_GSEA_gseaplot.png]

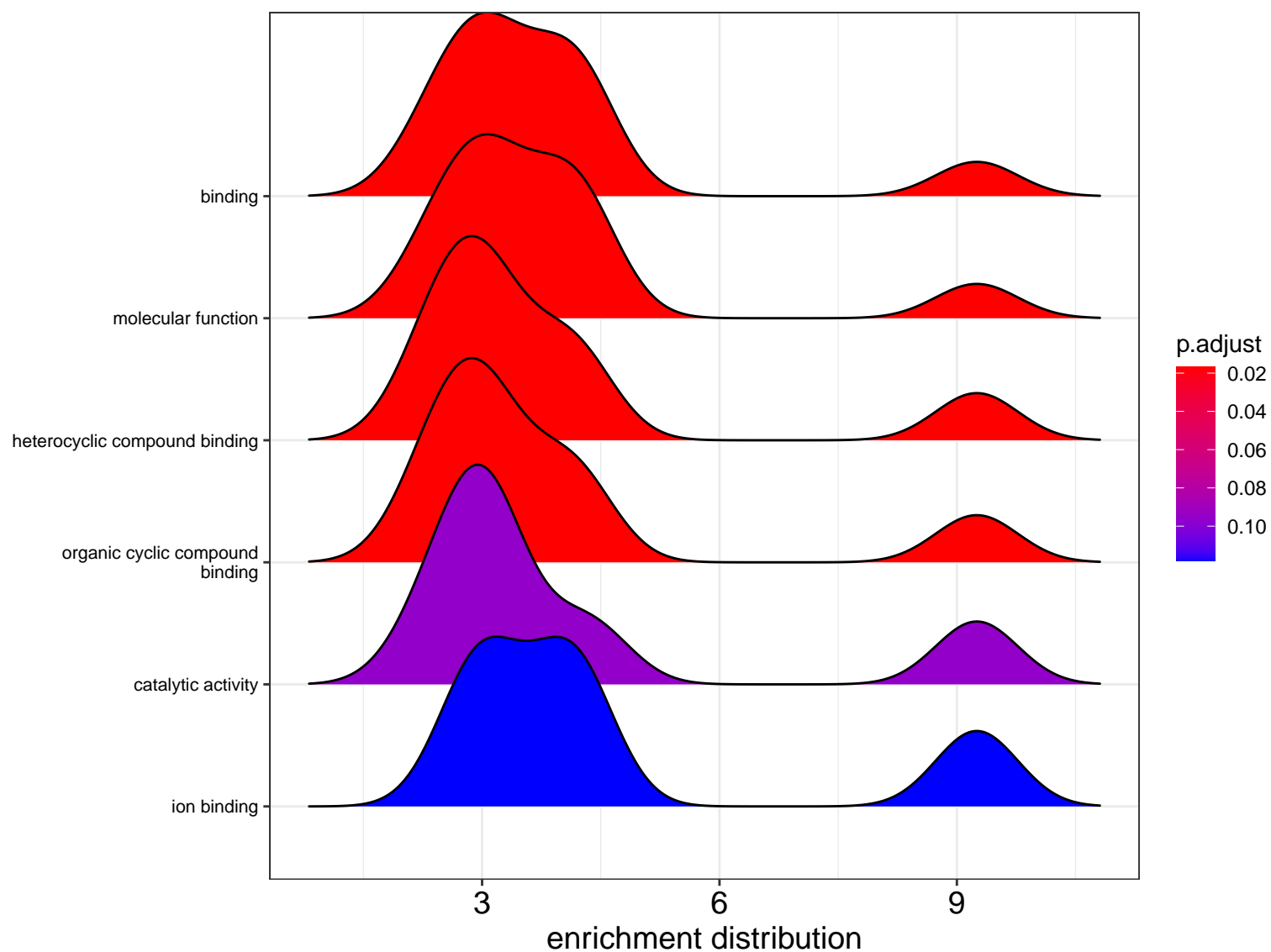

Supplement: Supplementary file 1 [file vaccines-12-00991-s001.zip › Supplementary File S3/proteome/4.Enrichment/gsea/5-infected_vs_5-uninfected/5-infected_vs_5-uninfected_GO_MF_GSEA_ridgeplot.pdf]

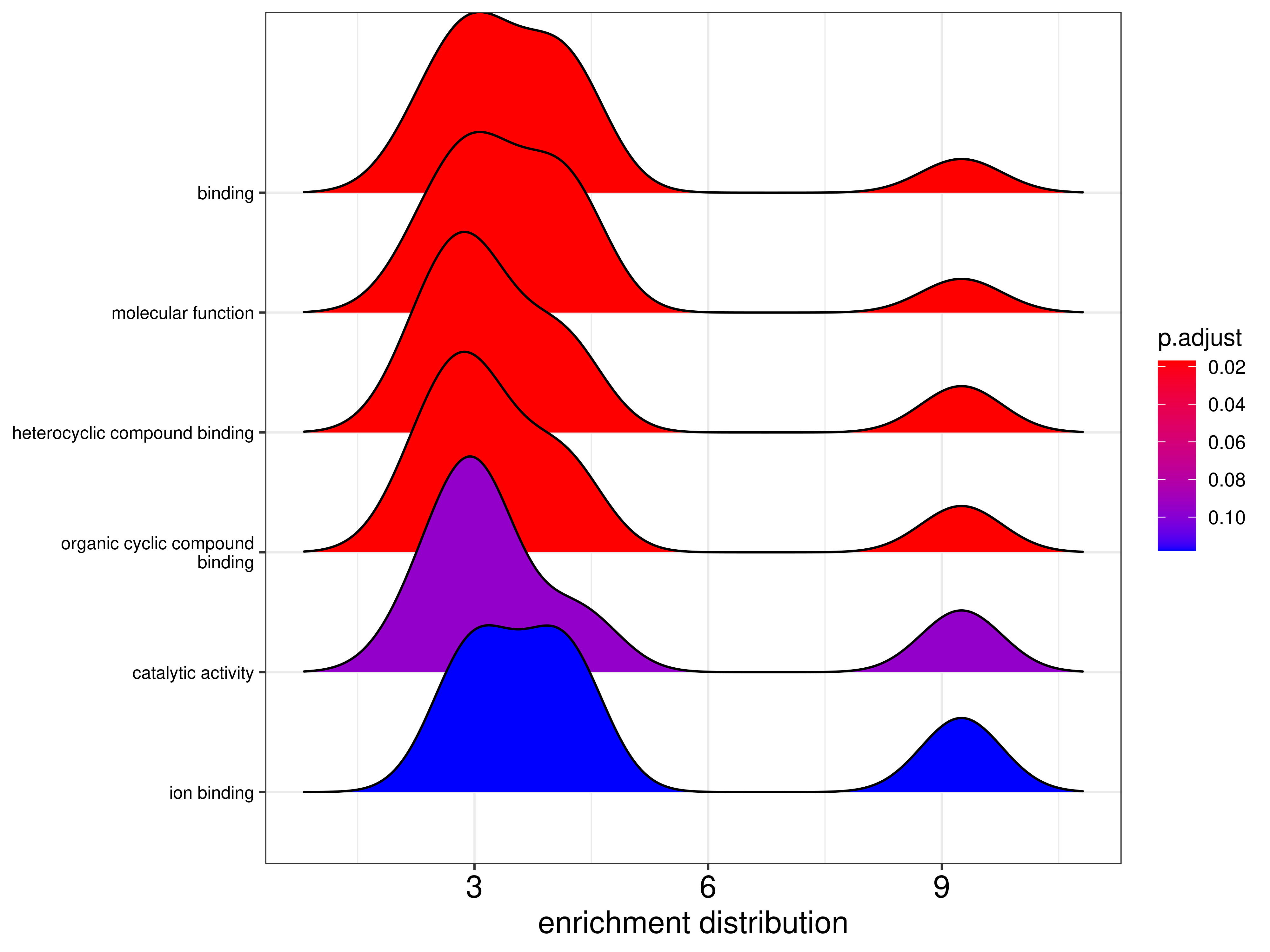

Supplement: Supplementary file 1 [file vaccines-12-00991-s001.zip › Supplementary File S3/proteome/4.Enrichment/gsea/5-infected_vs_5-uninfected/5-infected_vs_5-uninfected_GO_MF_GSEA_ridgeplot.png]

activated

Count

2

p.adjust

0.6635514

Metabolic pathways

0.050

0.075

0.100

0.125

GeneRatio

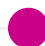

Supplement: Supplementary file 1 [file vaccines-12-00991-s001.zip › Supplementary File S3/proteome/4.Enrichment/gsea/5-infected_vs_5-uninfected/5-infected_vs_5-uninfected_KEGG_GSEA_dotplot.pdf]

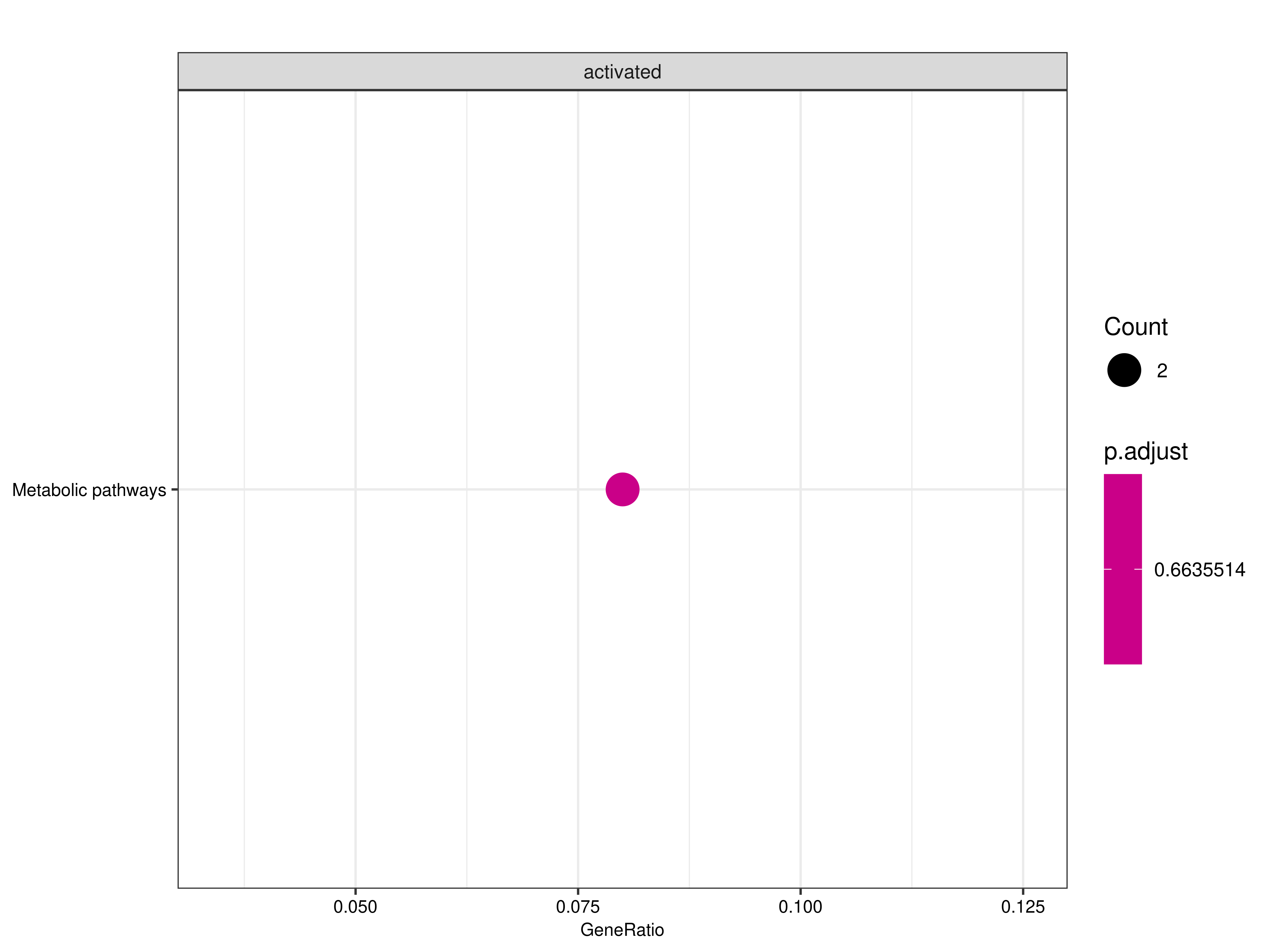

Supplement: Supplementary file 1 [file vaccines-12-00991-s001.zip › Supplementary File S3/proteome/4.Enrichment/gsea/5-infected_vs_5-uninfected/5-infected_vs_5-uninfected_KEGG_GSEA_dotplot.png]

# Metabolic pathways

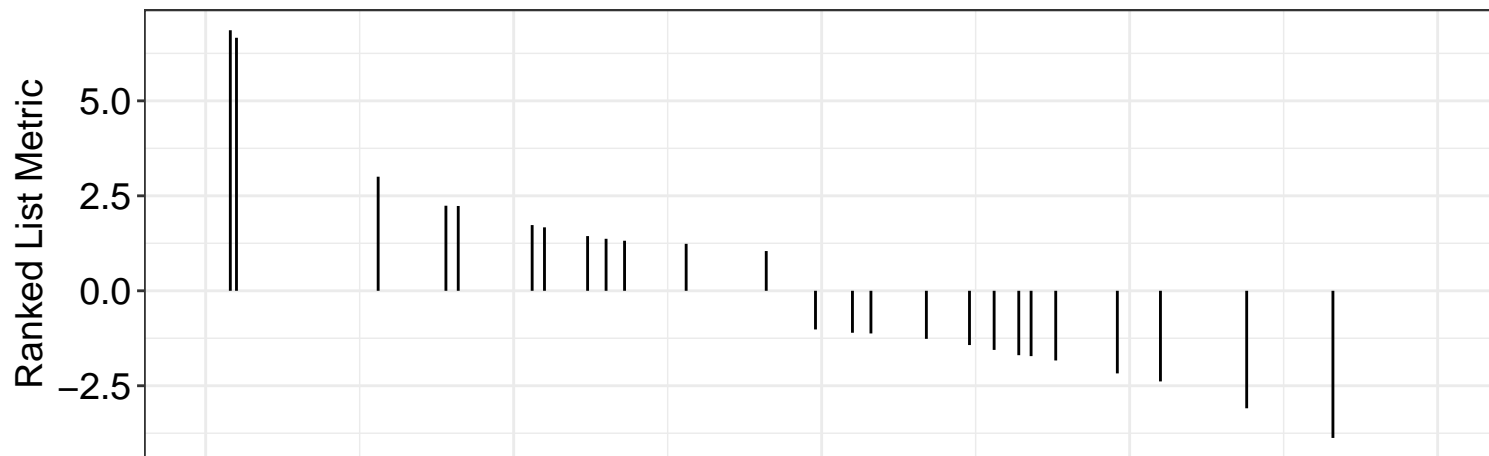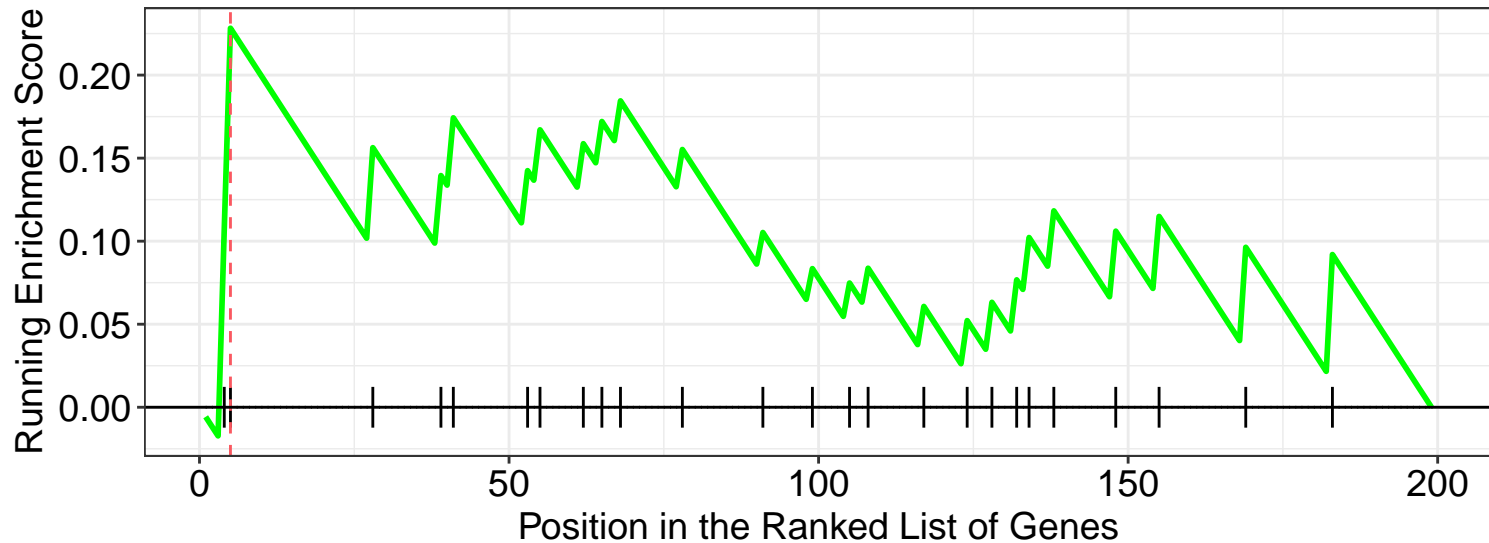

Supplement: Supplementary file 1 [file vaccines-12-00991-s001.zip › Supplementary File S3/proteome/4.Enrichment/gsea/5-infected_vs_5-uninfected/5-infected_vs_5-uninfected_KEGG_GSEA_gseaplot.pdf]

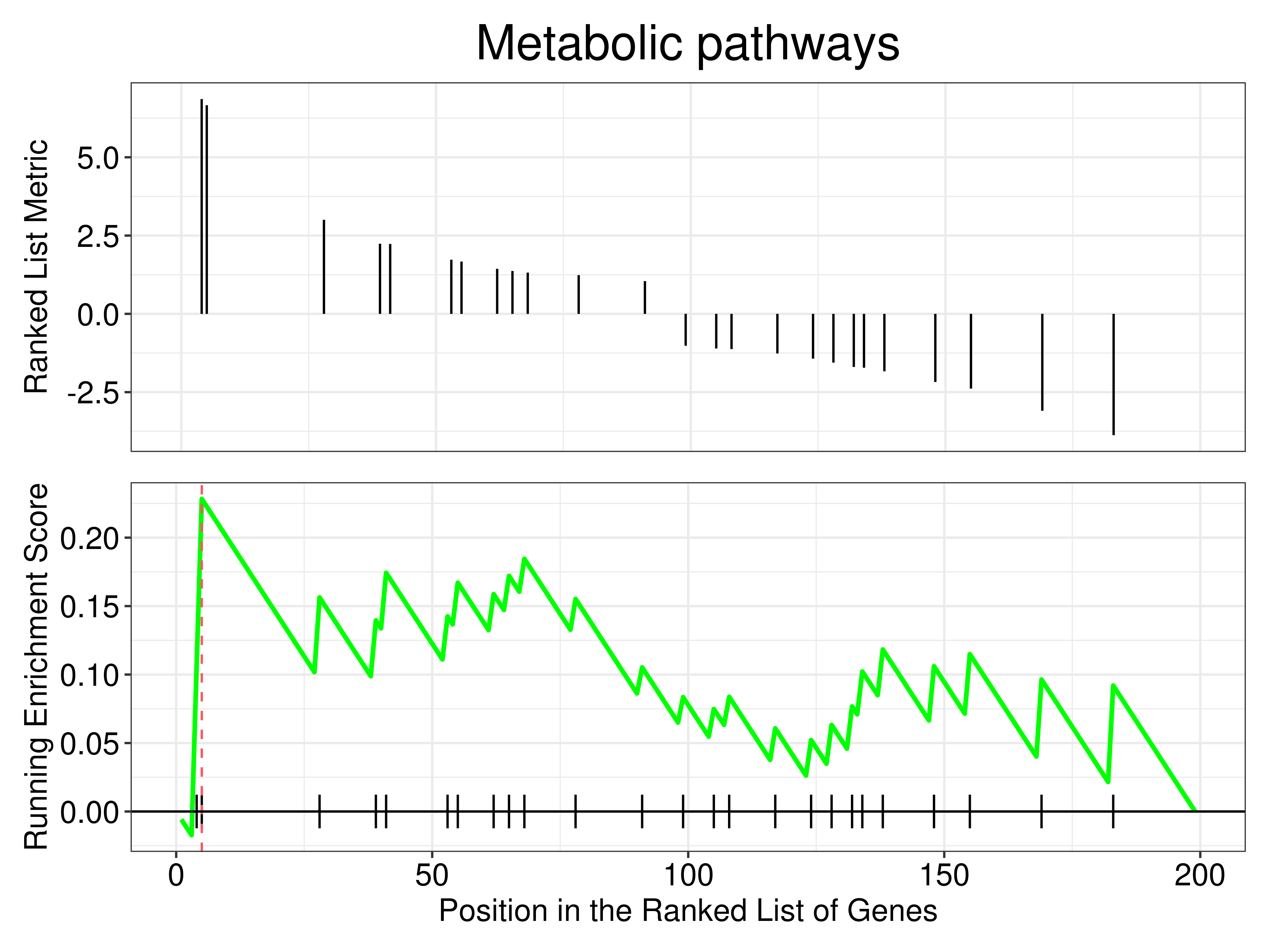

Supplement: Supplementary file 1 [file vaccines-12-00991-s001.zip › Supplementary File S3/proteome/4.Enrichment/gsea/5-infected_vs_5-uninfected/5-infected_vs_5-uninfected_KEGG_GSEA_gseaplot.png]

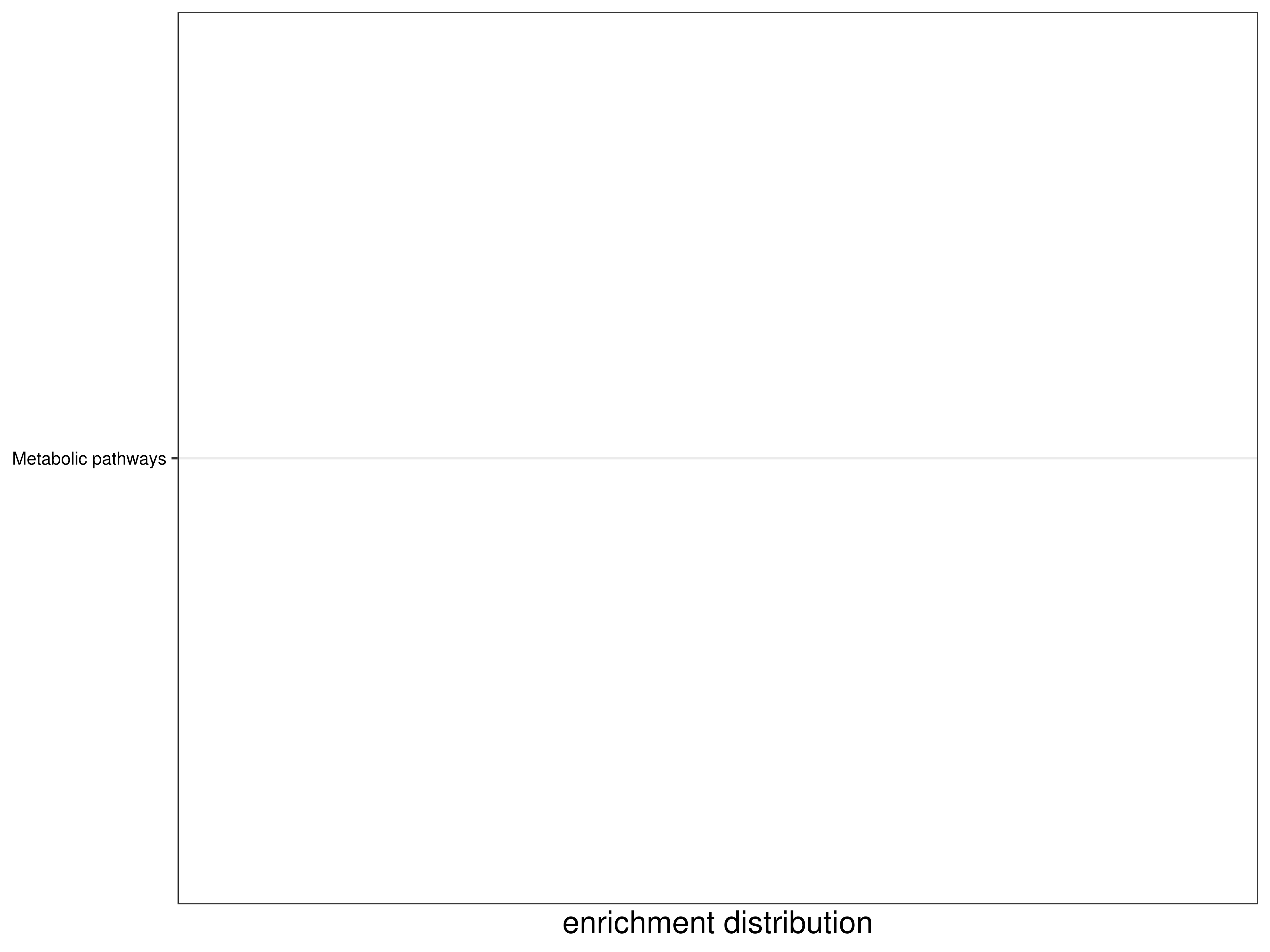

Supplement: Supplementary file 1 [file vaccines-12-00991-s001.zip › Supplementary File S3/proteome/4.Enrichment/gsea/5-infected_vs_5-uninfected/5-infected_vs_5-uninfected_KEGG_GSEA_ridgeplot.png]
