# Supplementary figures and images for: Production, Passaging Stability, and Histological Analysis of Madin–Darby Canine Kidney Cells Cultured in a Low-Serum Medium (part 1 of 2)
Source: Vaccines (Basel). 2024 Aug 30;12(9):991. doi: 10.3390/vaccines12090991 (PMC11435615; doi:10.3390/vaccines12090991)

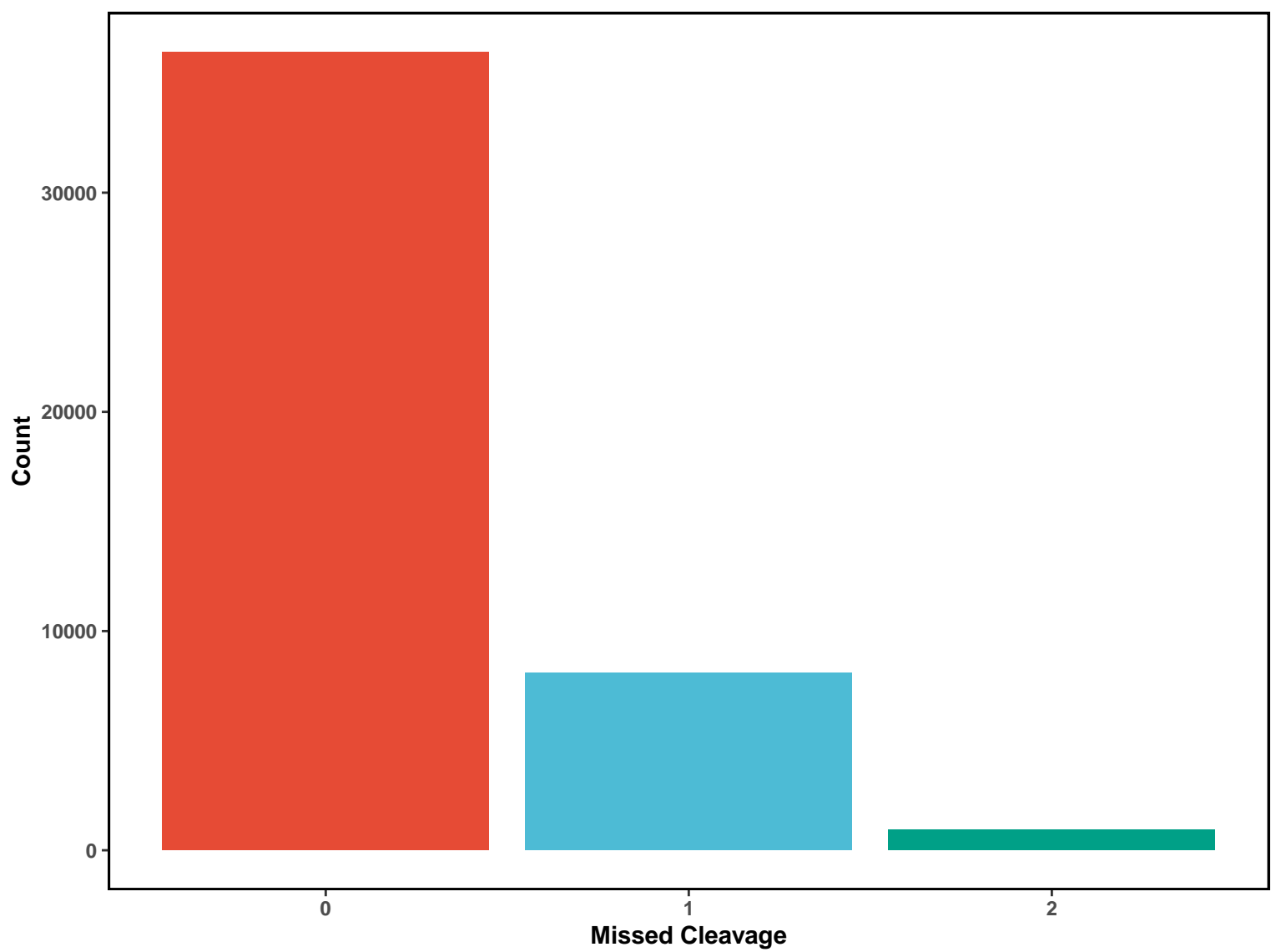

Supplement: Supplementary file 1 [file vaccines-12-00991-s001.zip › Supplementary File S3/proteome/1.QualityControl/stats/ly_missed_cleavages.pdf]

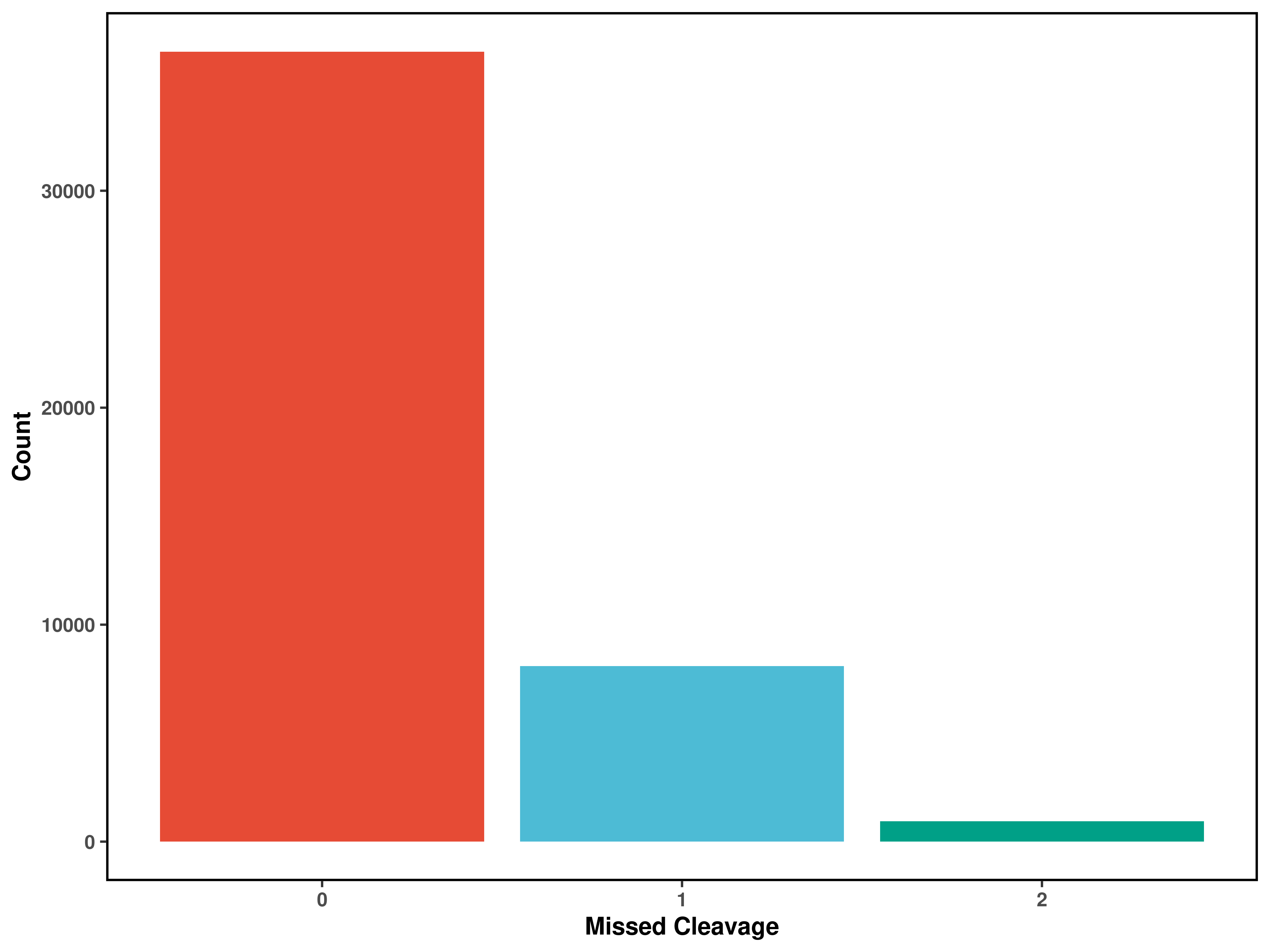

Supplement: Supplementary file 1 [file vaccines-12-00991-s001.zip › Supplementary File S3/proteome/1.QualityControl/stats/ly_missed_cleavages.png]

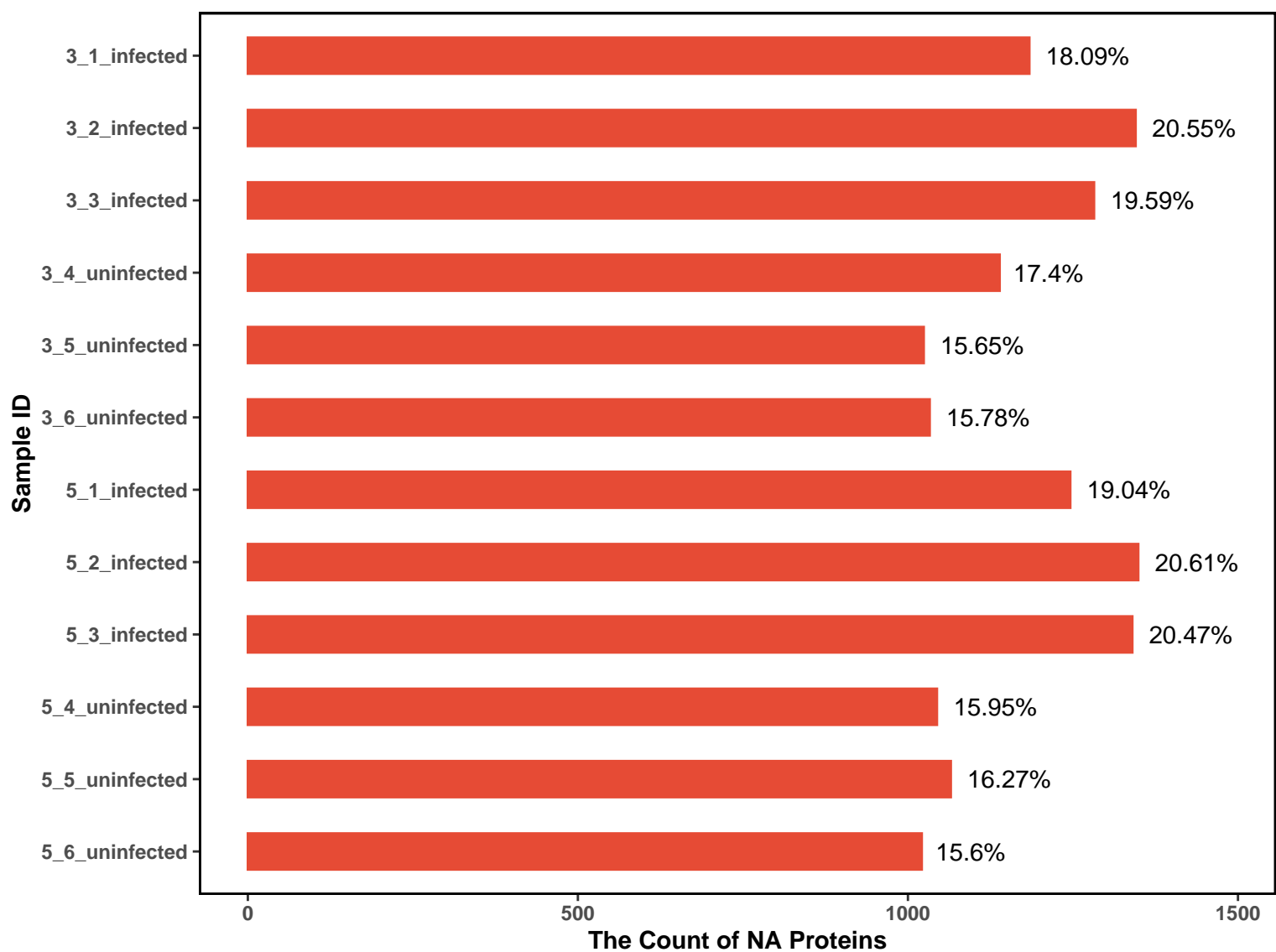

Supplement: Supplementary file 1 [file vaccines-12-00991-s001.zip › Supplementary File S3/proteome/1.QualityControl/stats/ly_missing_stats.pdf]

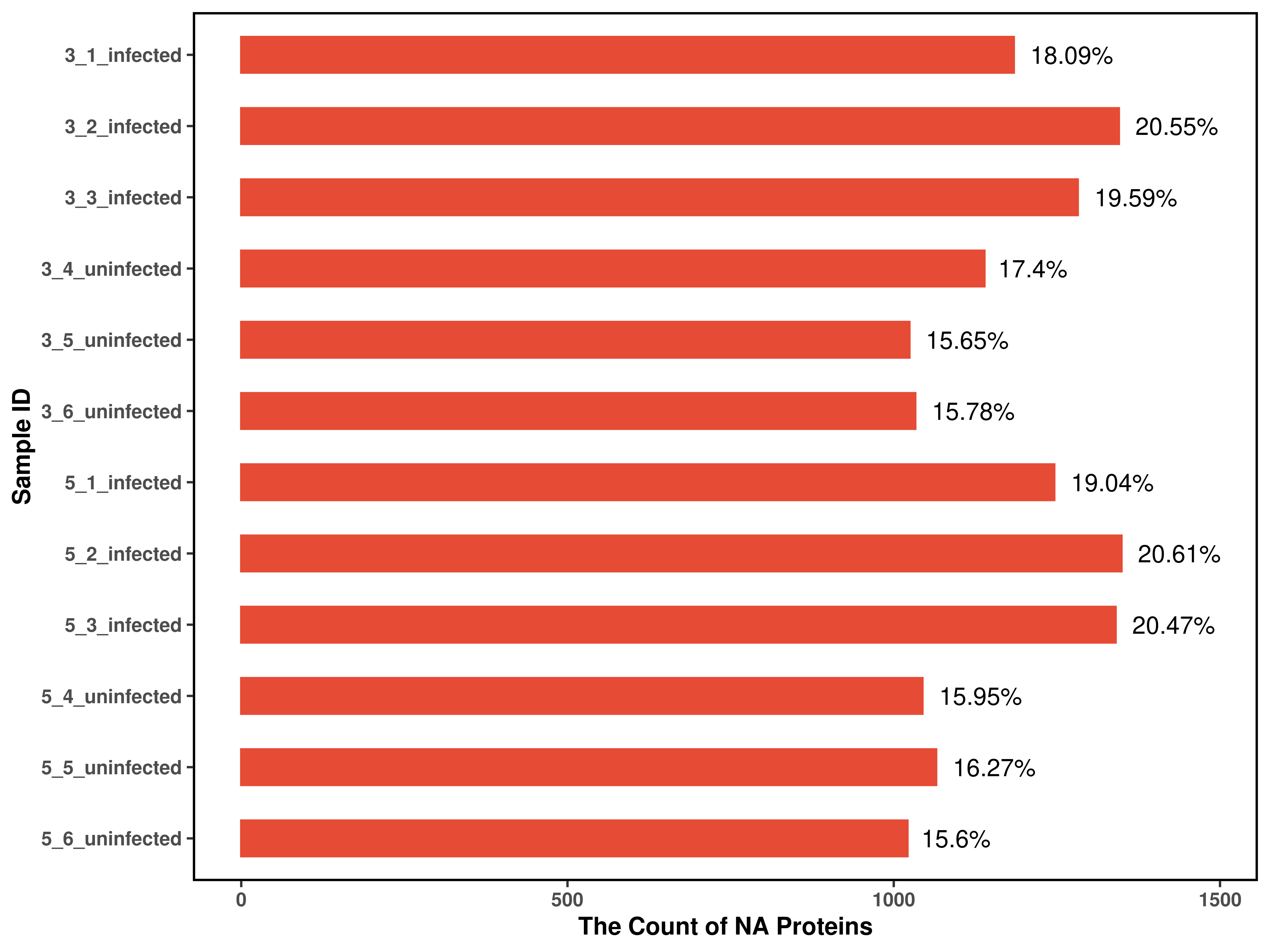

Supplement: Supplementary file 1 [file vaccines-12-00991-s001.zip › Supplementary File S3/proteome/1.QualityControl/stats/ly_missing_stats.png]

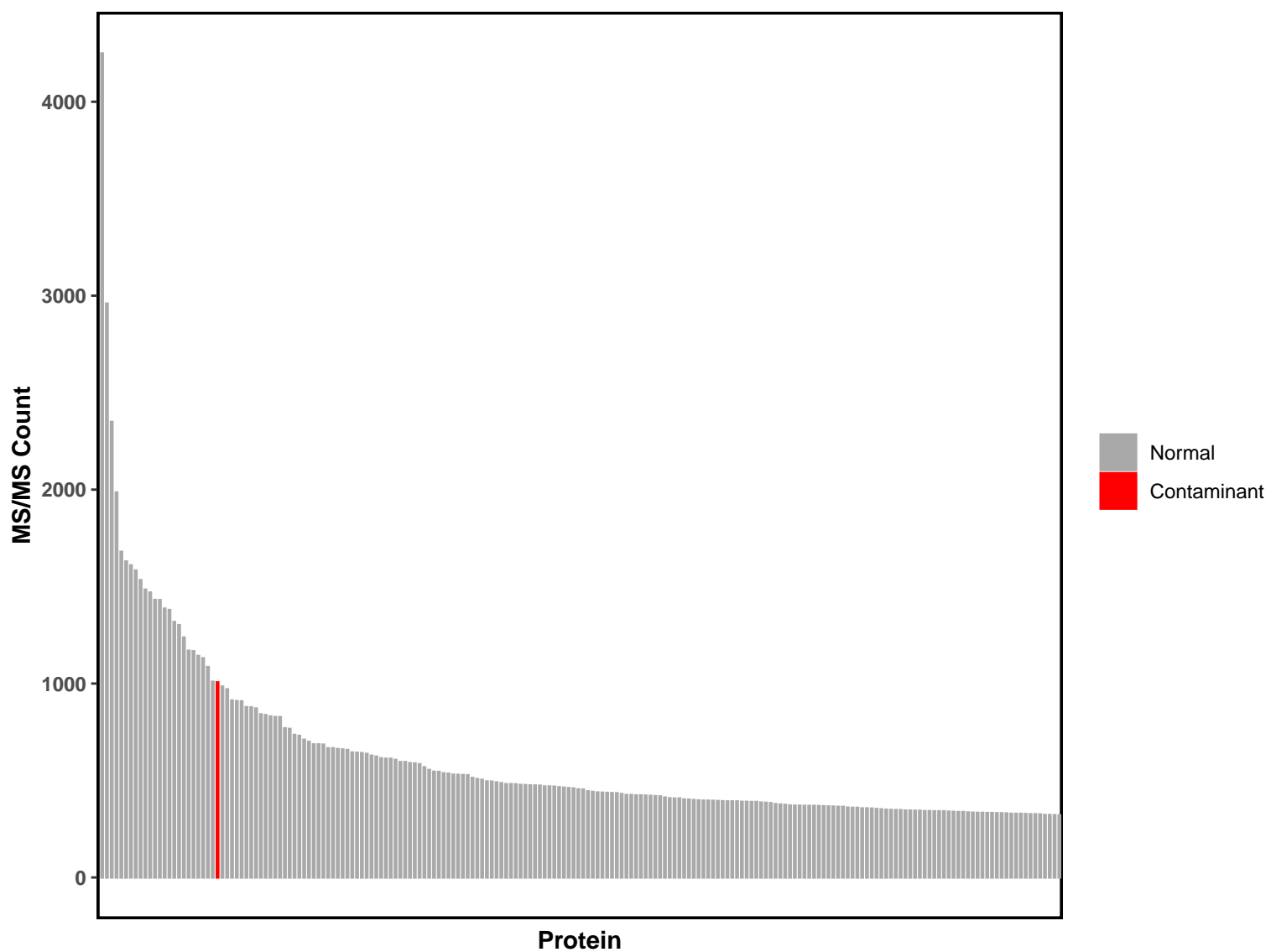

Supplement: Supplementary file 1 [file vaccines-12-00991-s001.zip › Supplementary File S3/proteome/1.QualityControl/stats/ly_msms_count.pdf]

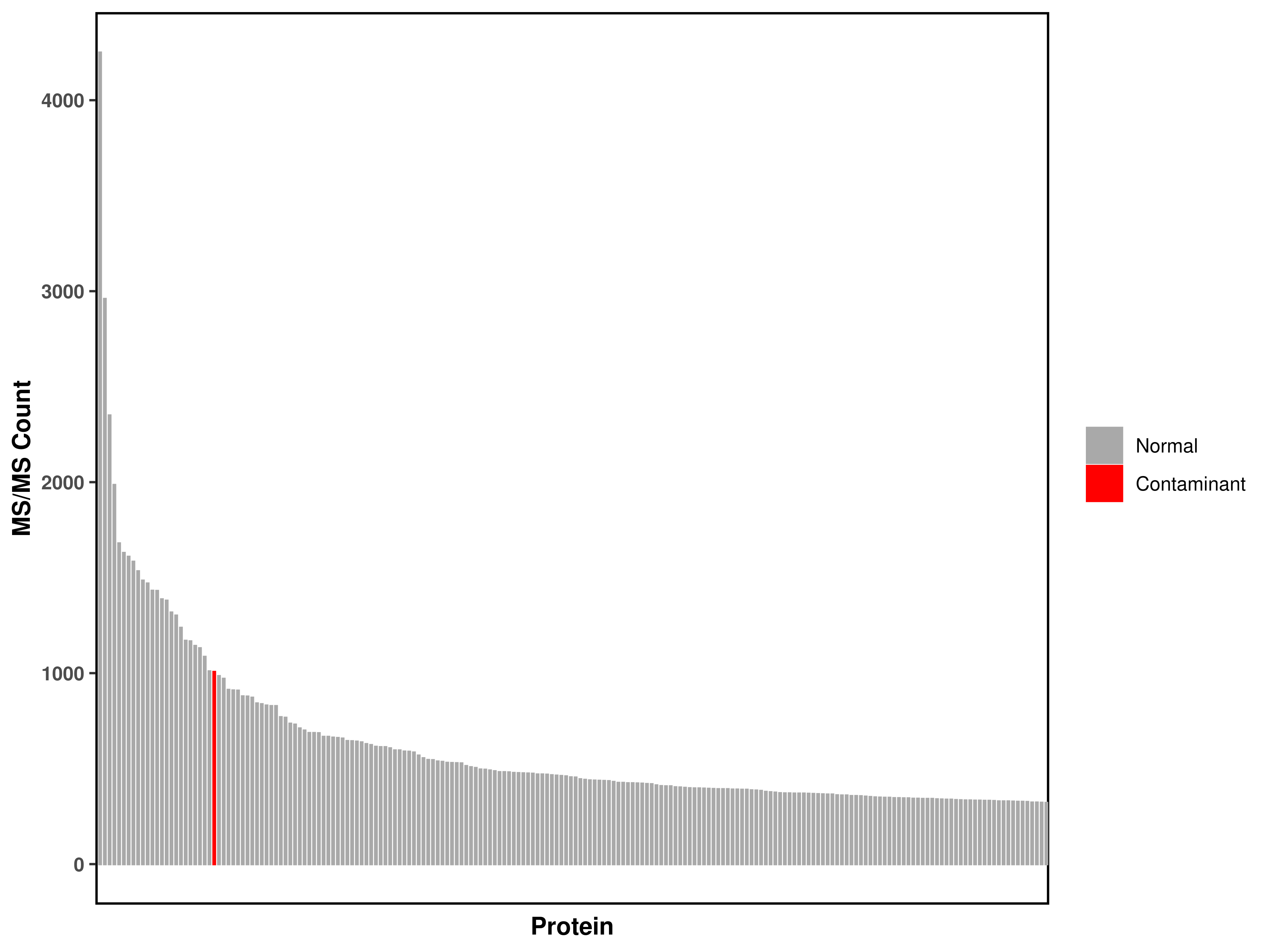

Supplement: Supplementary file 1 [file vaccines-12-00991-s001.zip › Supplementary File S3/proteome/1.QualityControl/stats/ly_msms_count.png]

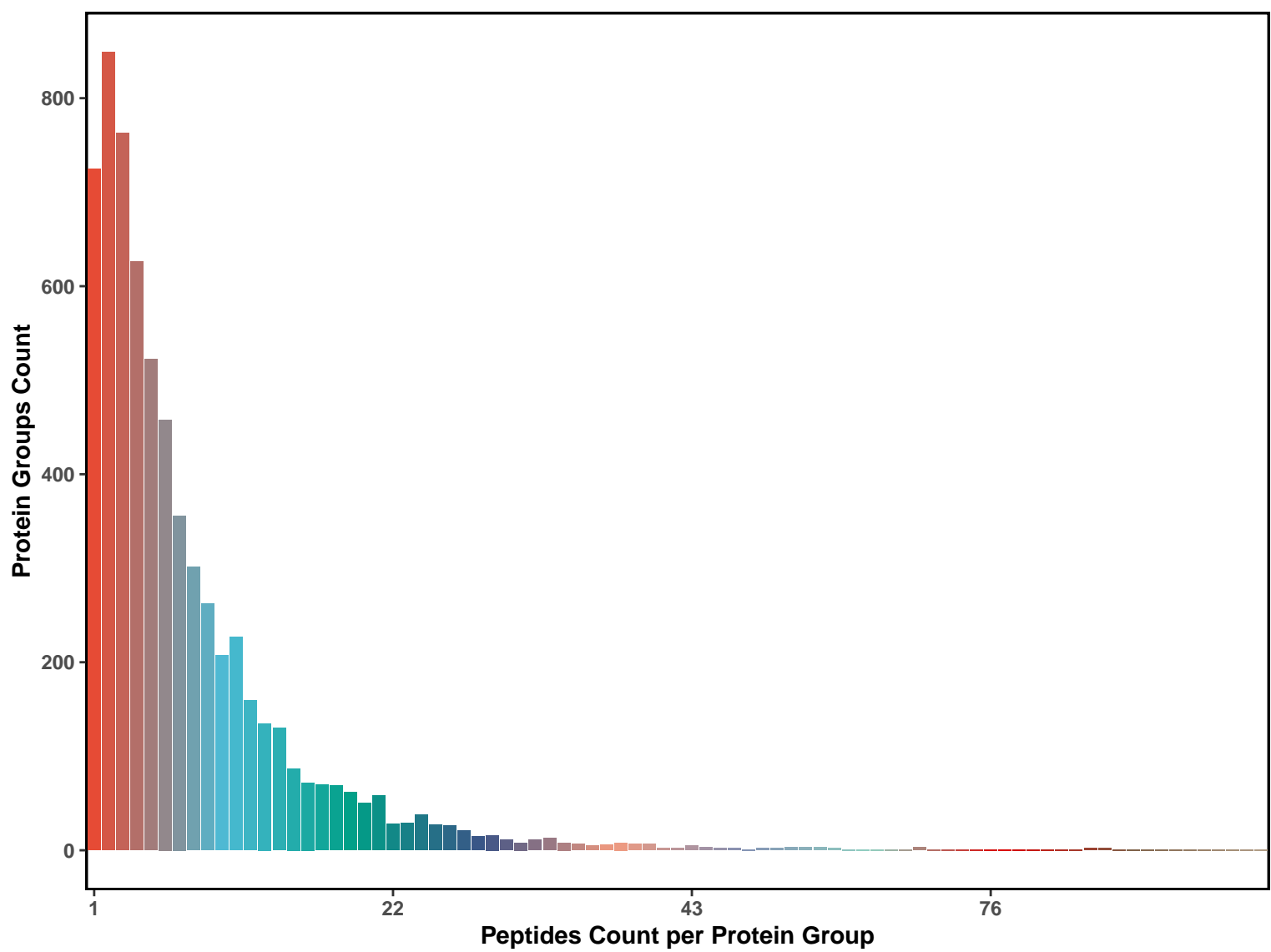

Supplement: Supplementary file 1 [file vaccines-12-00991-s001.zip › Supplementary File S3/proteome/1.QualityControl/stats/ly_peptides_count.pdf]

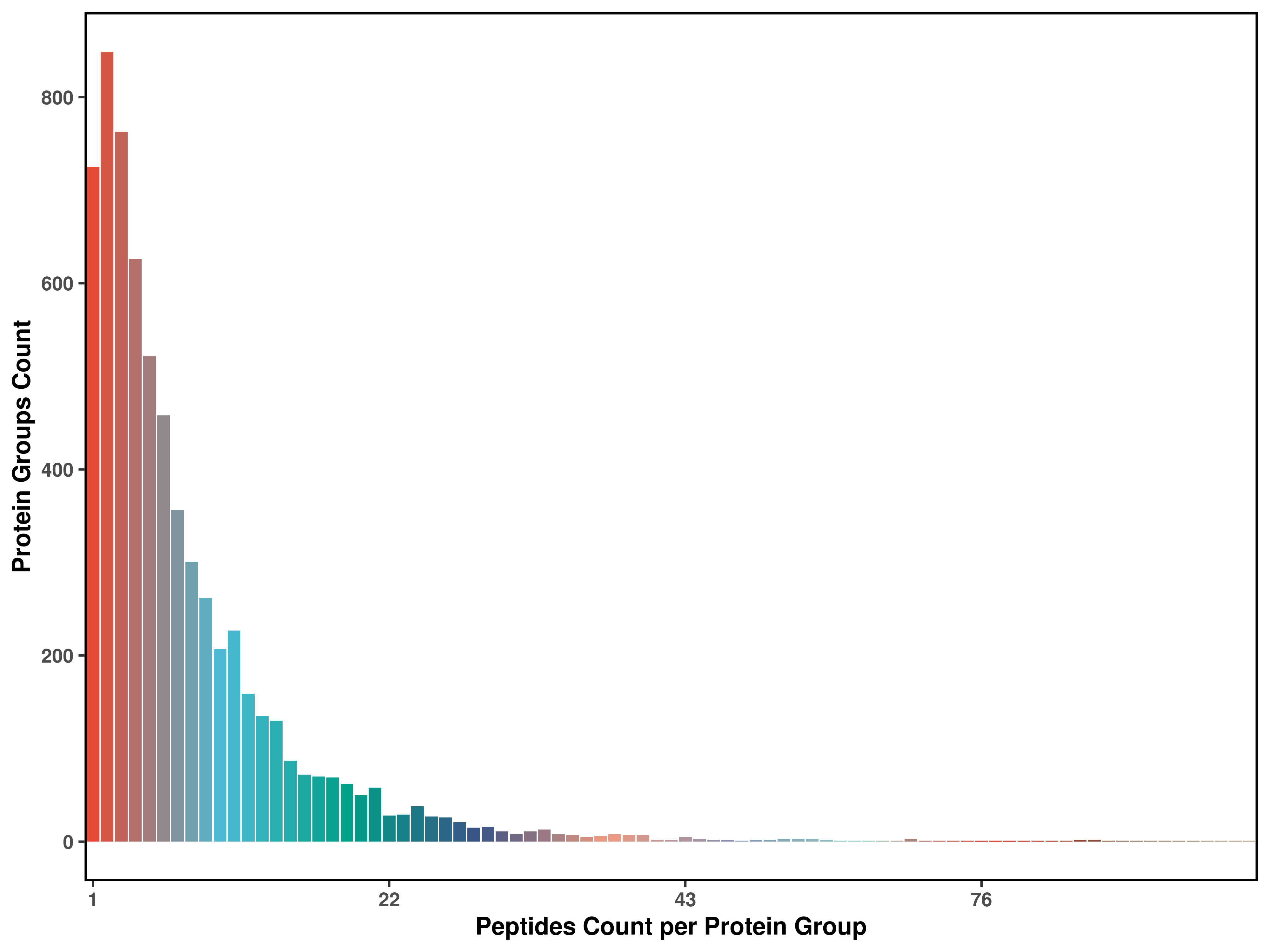

Supplement: Supplementary file 1 [file vaccines-12-00991-s001.zip › Supplementary File S3/proteome/1.QualityControl/stats/ly_peptides_count.png]

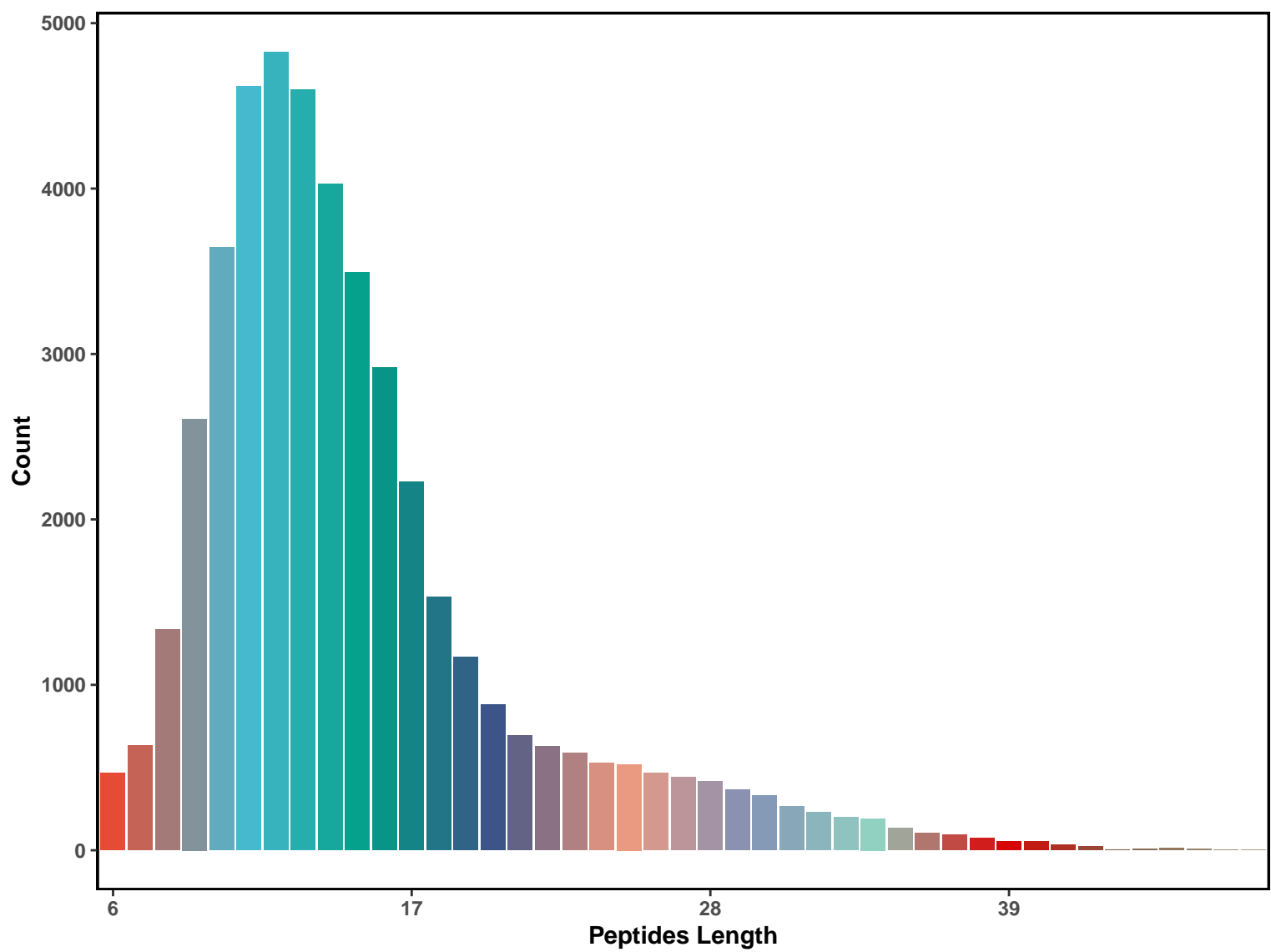

Supplement: Supplementary file 1 [file vaccines-12-00991-s001.zip › Supplementary File S3/proteome/1.QualityControl/stats/ly_peptides_length.pdf]

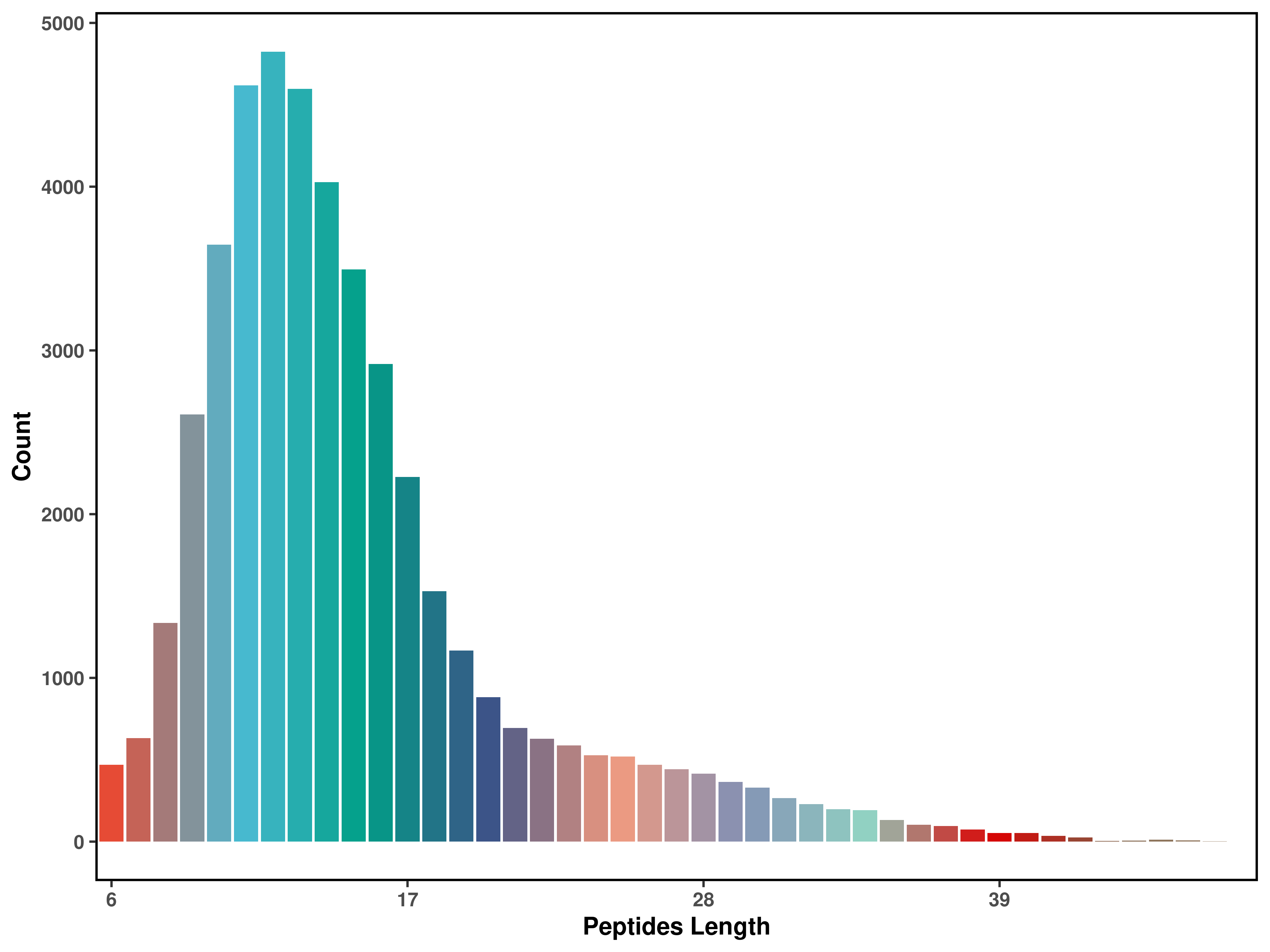

Supplement: Supplementary file 1 [file vaccines-12-00991-s001.zip › Supplementary File S3/proteome/1.QualityControl/stats/ly_peptides_length.png]

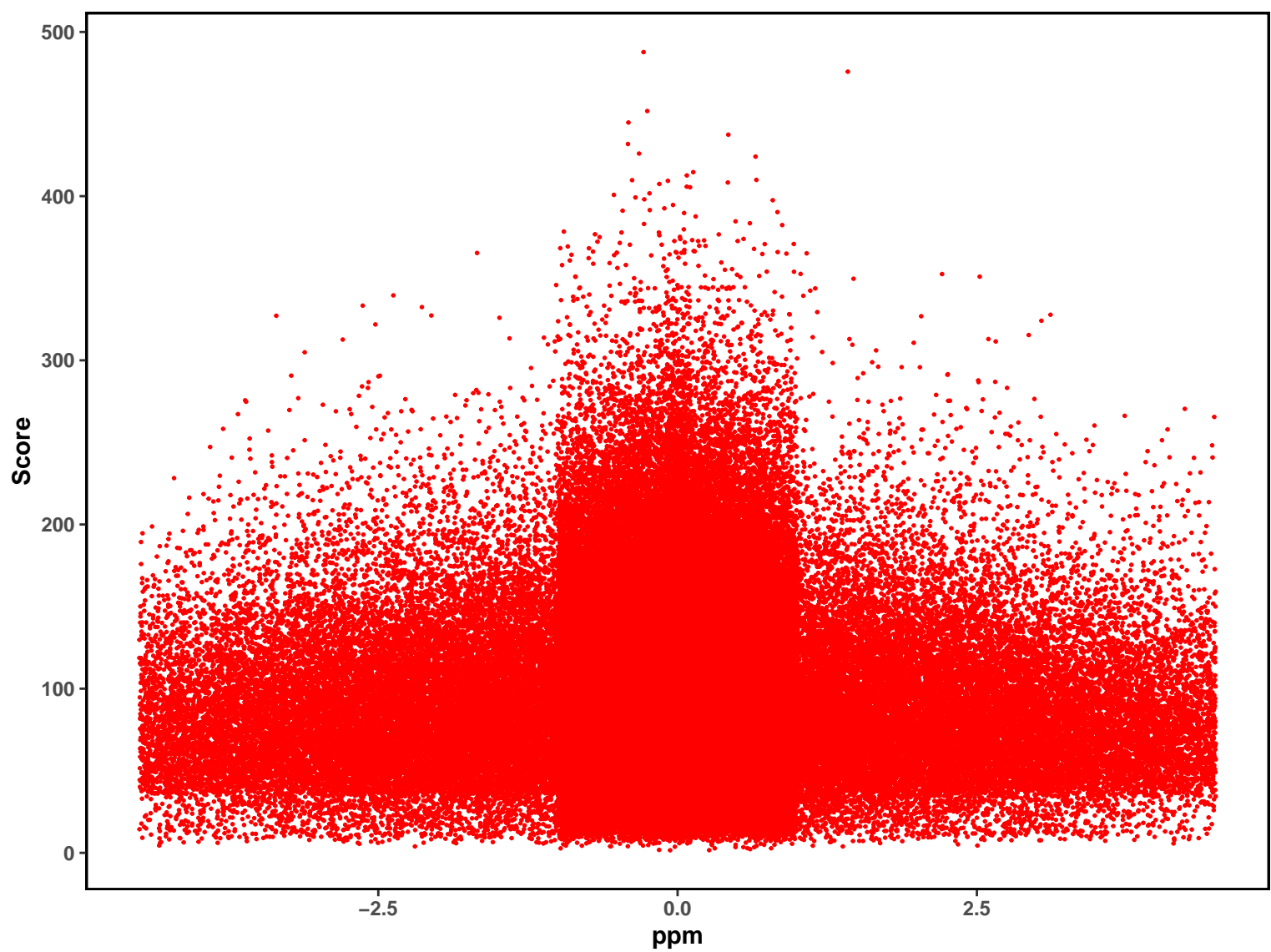

Supplement: Supplementary file 1 [file vaccines-12-00991-s001.zip › Supplementary File S3/proteome/1.QualityControl/stats/ly_ppm_score.pdf]

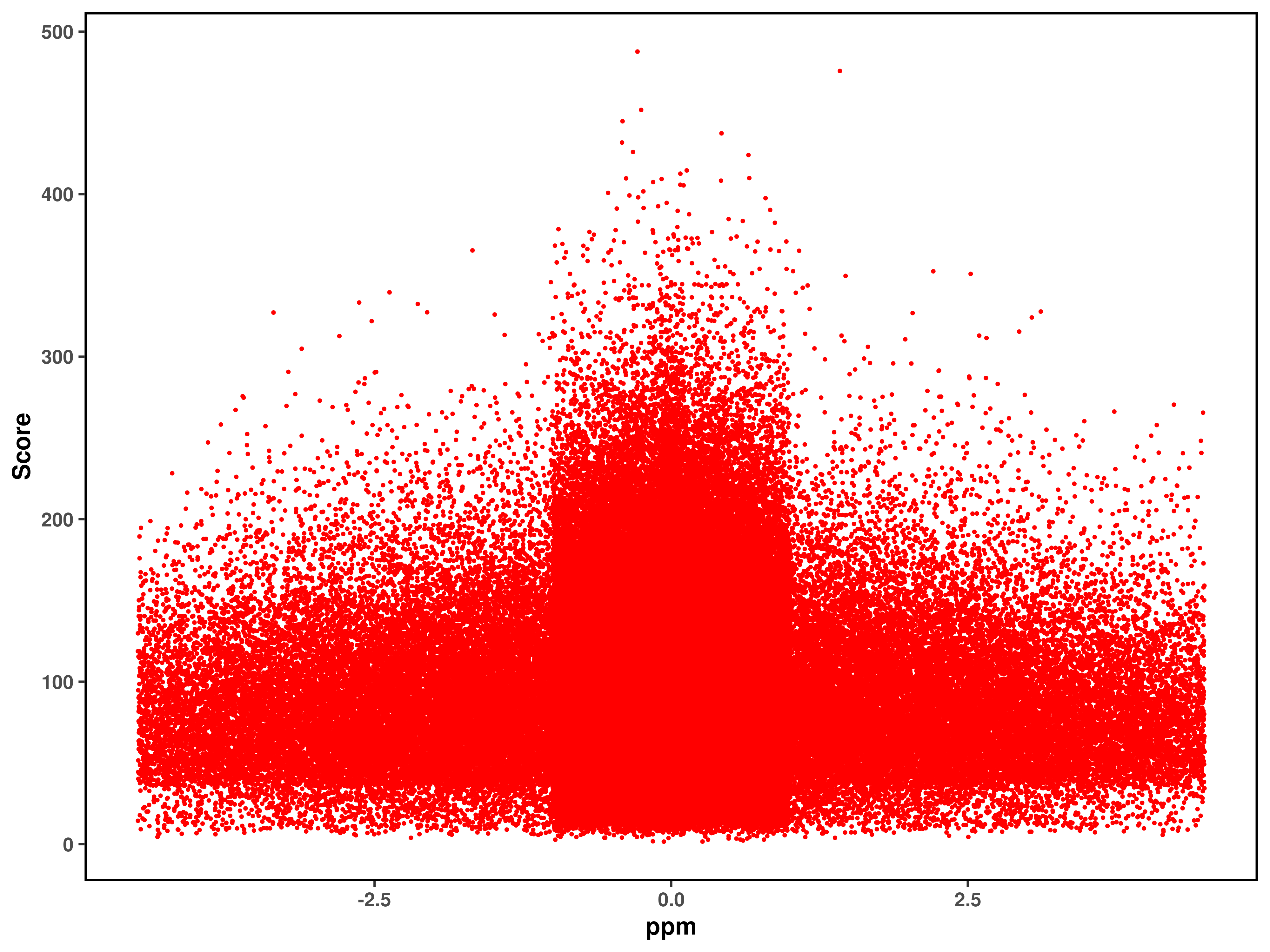

Supplement: Supplementary file 1 [file vaccines-12-00991-s001.zip › Supplementary File S3/proteome/1.QualityControl/stats/ly_ppm_score.png]

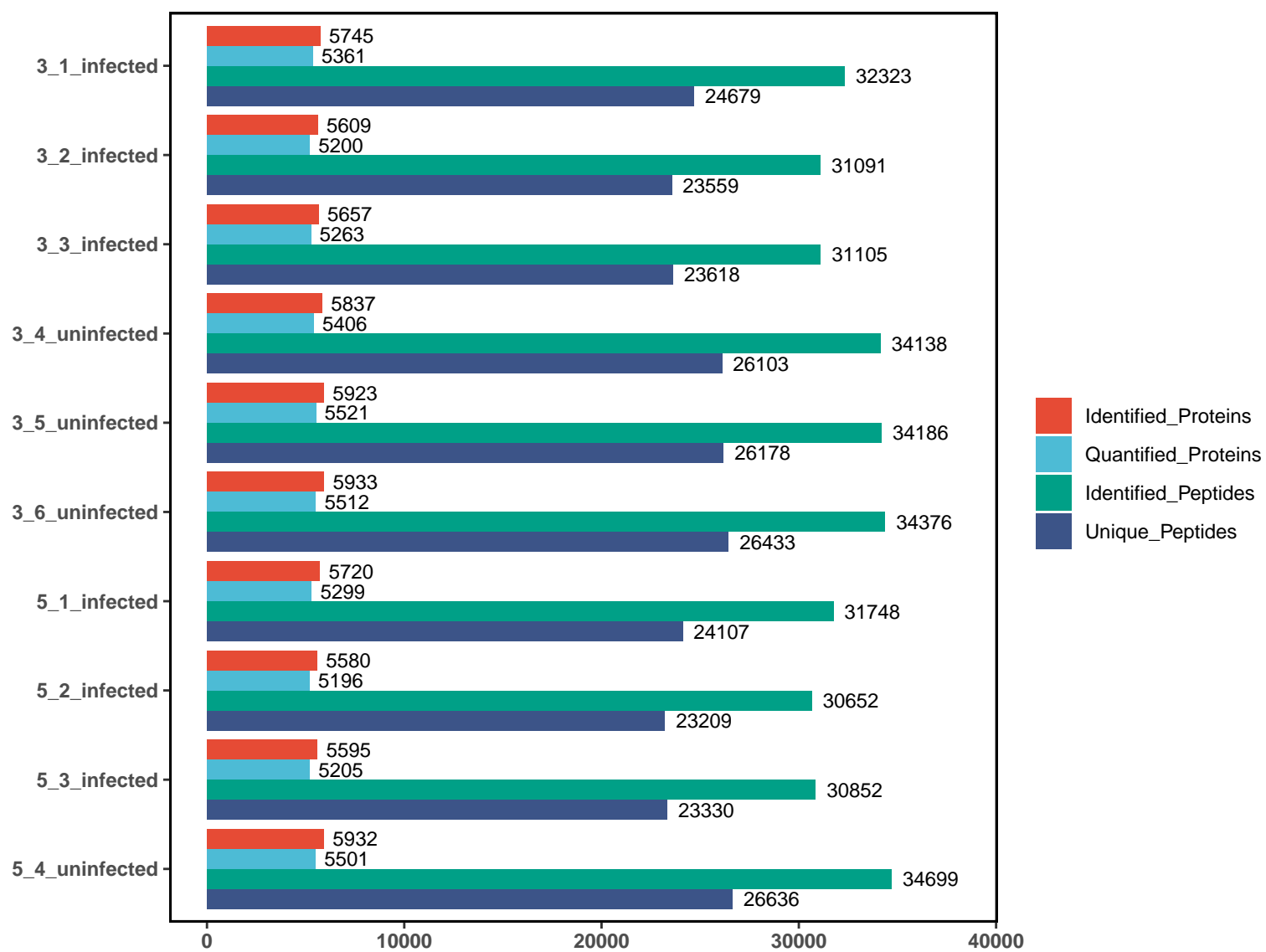

Supplement: Supplementary file 1 [file vaccines-12-00991-s001.zip › Supplementary File S3/proteome/1.QualityControl/stats/ly_sample_stats.pdf]

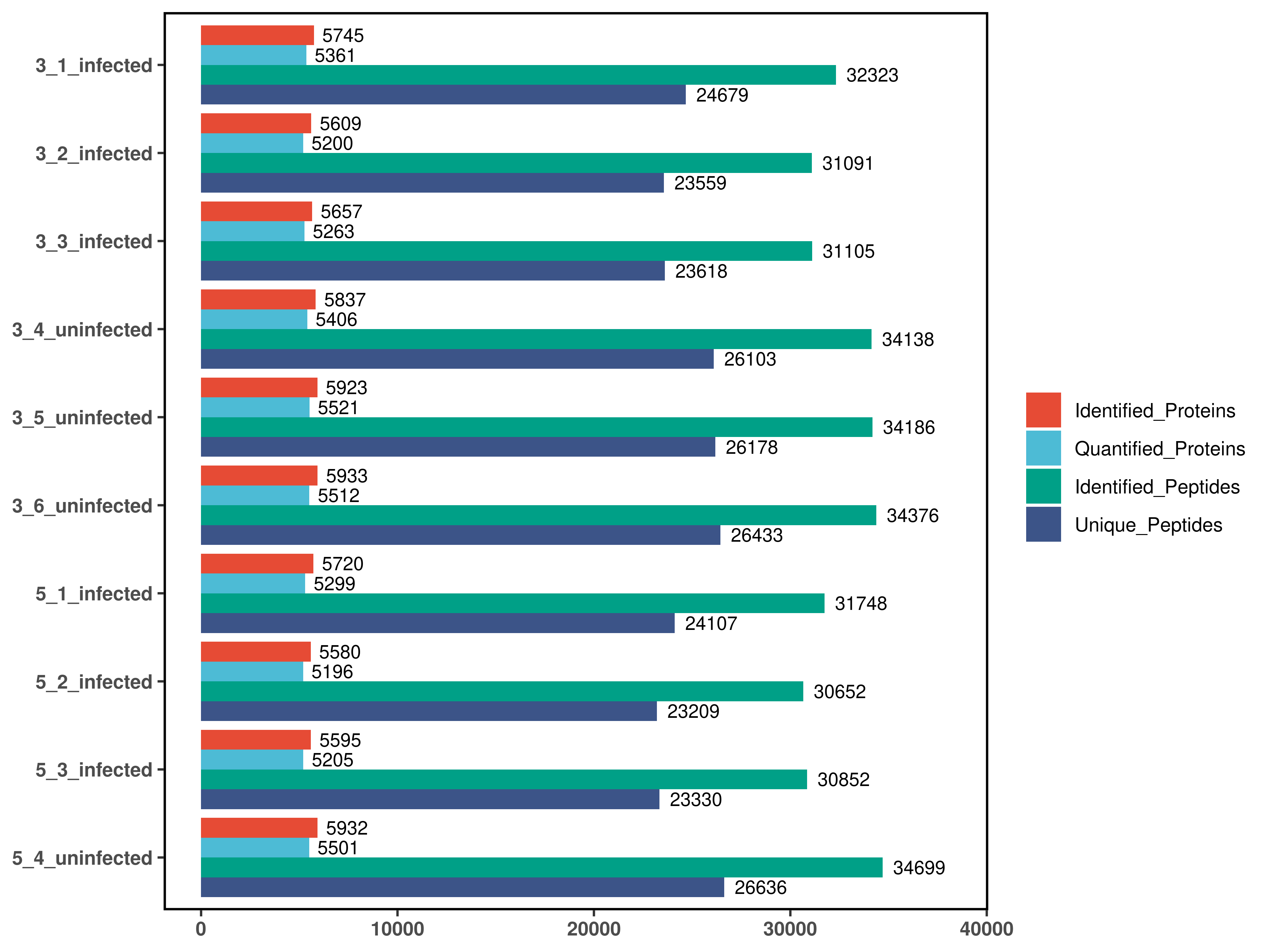

Supplement: Supplementary file 1 [file vaccines-12-00991-s001.zip › Supplementary File S3/proteome/1.QualityControl/stats/ly_sample_stats.png]

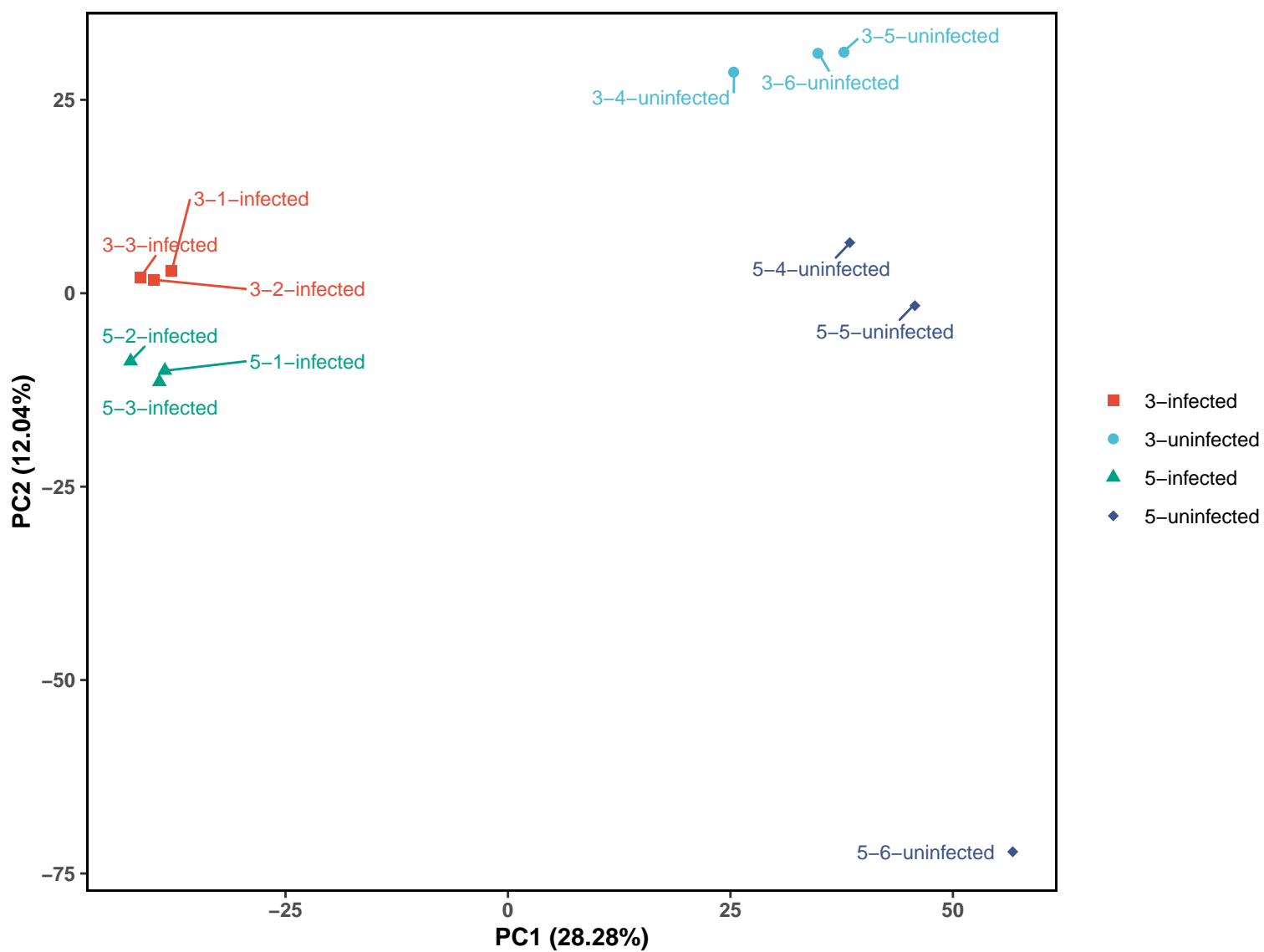

Supplement: Supplementary file 1 [file vaccines-12-00991-s001.zip › Supplementary File S3/proteome/2.Quantification/stats/ly_2d_pca.pdf]

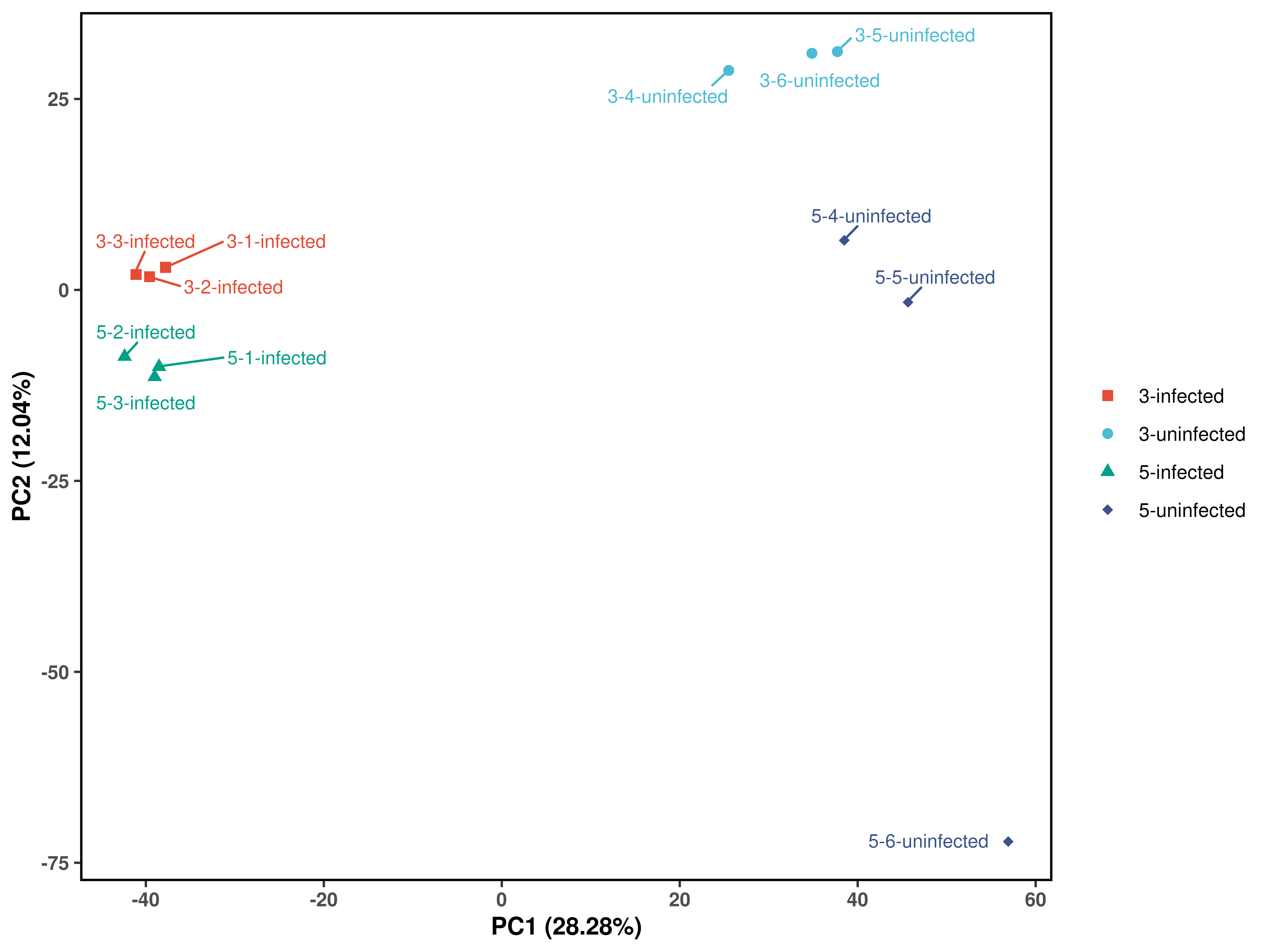

Supplement: Supplementary file 1 [file vaccines-12-00991-s001.zip › Supplementary File S3/proteome/2.Quantification/stats/ly_2d_pca.png]

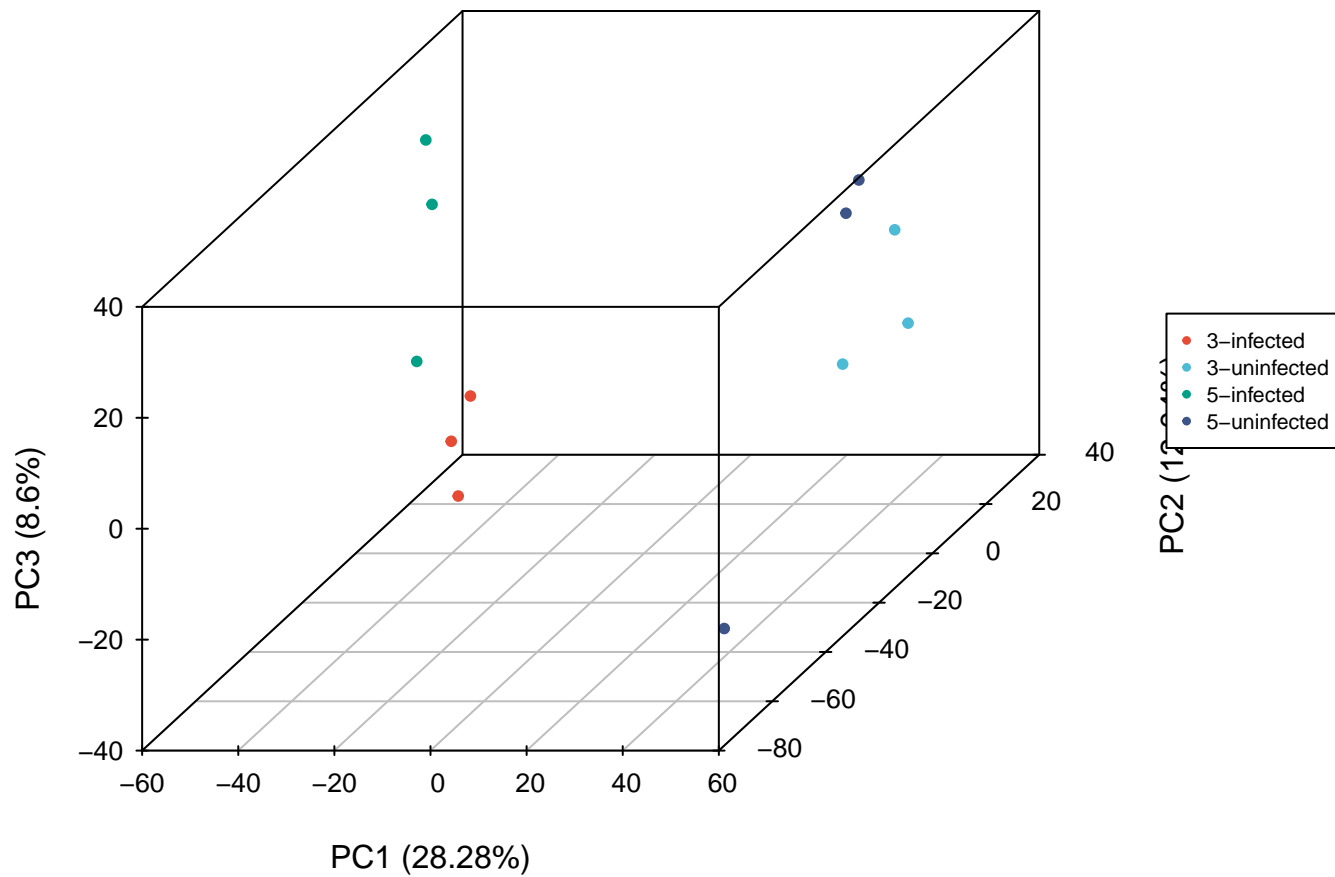

Supplement: Supplementary file 1 [file vaccines-12-00991-s001.zip › Supplementary File S3/proteome/2.Quantification/stats/ly_3d_pca.pdf]

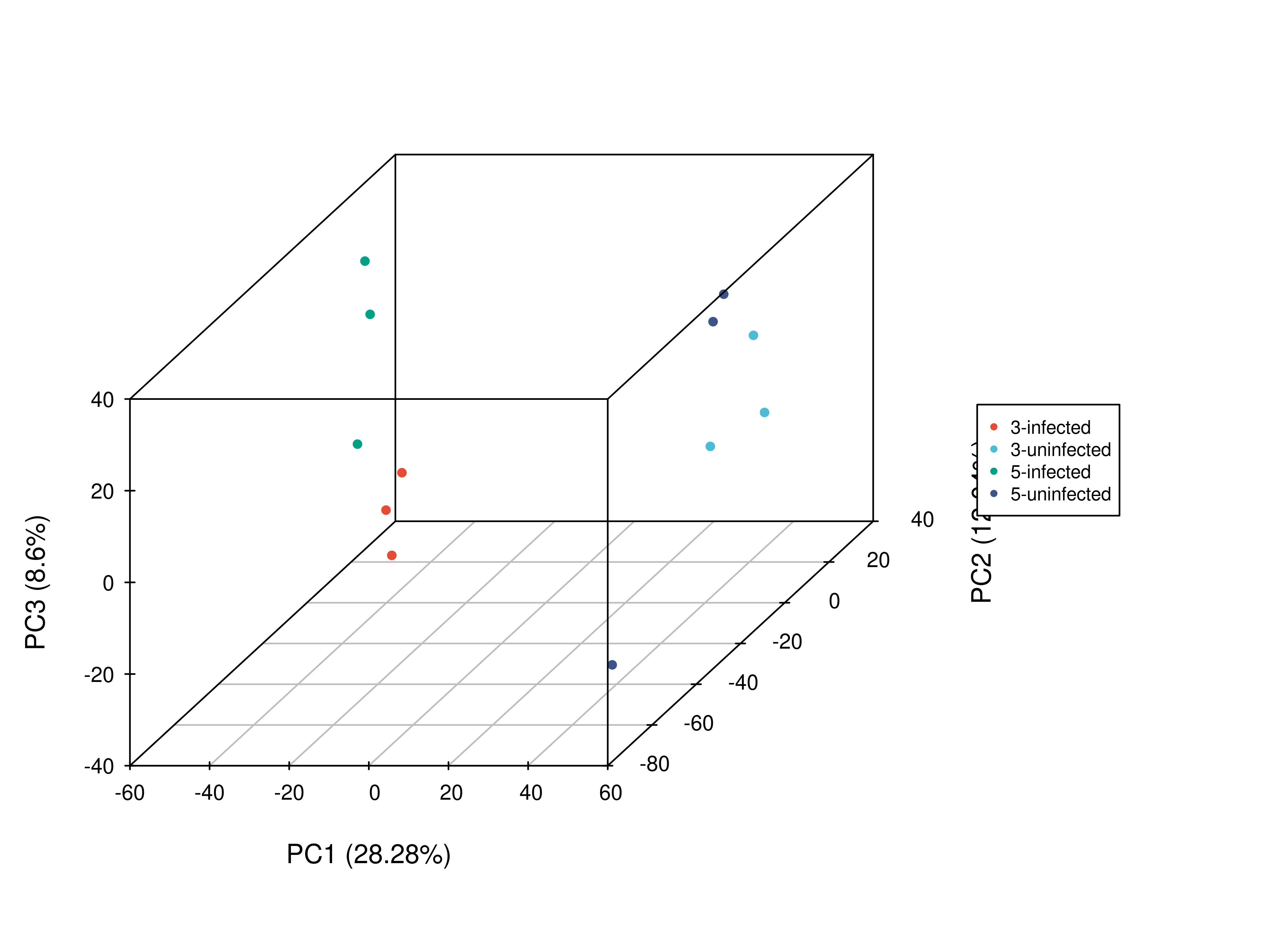

Supplement: Supplementary file 1 [file vaccines-12-00991-s001.zip › Supplementary File S3/proteome/2.Quantification/stats/ly_3d_pca.png]

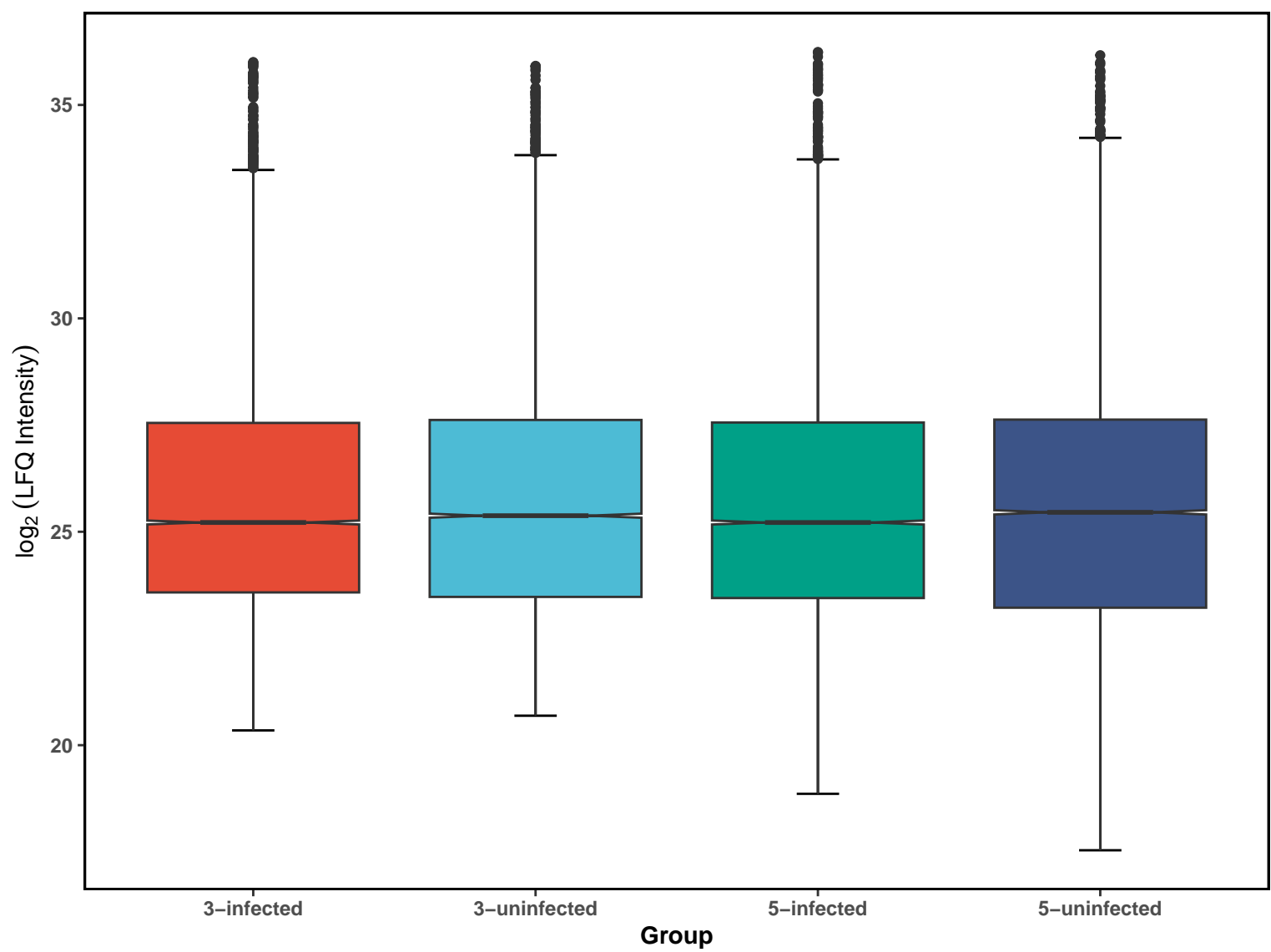

Supplement: Supplementary file 1 [file vaccines-12-00991-s001.zip › Supplementary File S3/proteome/2.Quantification/stats/ly_boxplot.pdf]

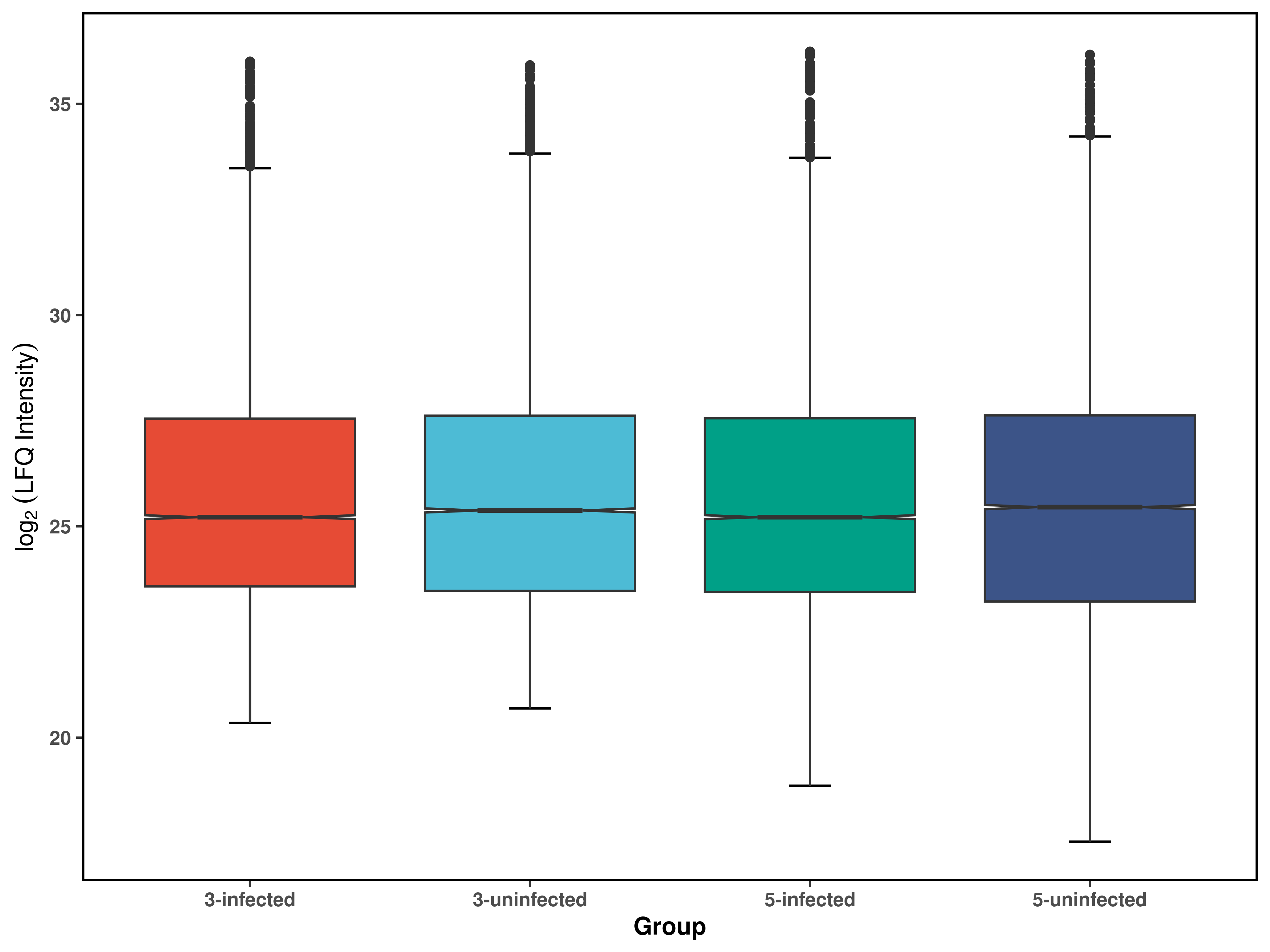

Supplement: Supplementary file 1 [file vaccines-12-00991-s001.zip › Supplementary File S3/proteome/2.Quantification/stats/ly_boxplot.png]

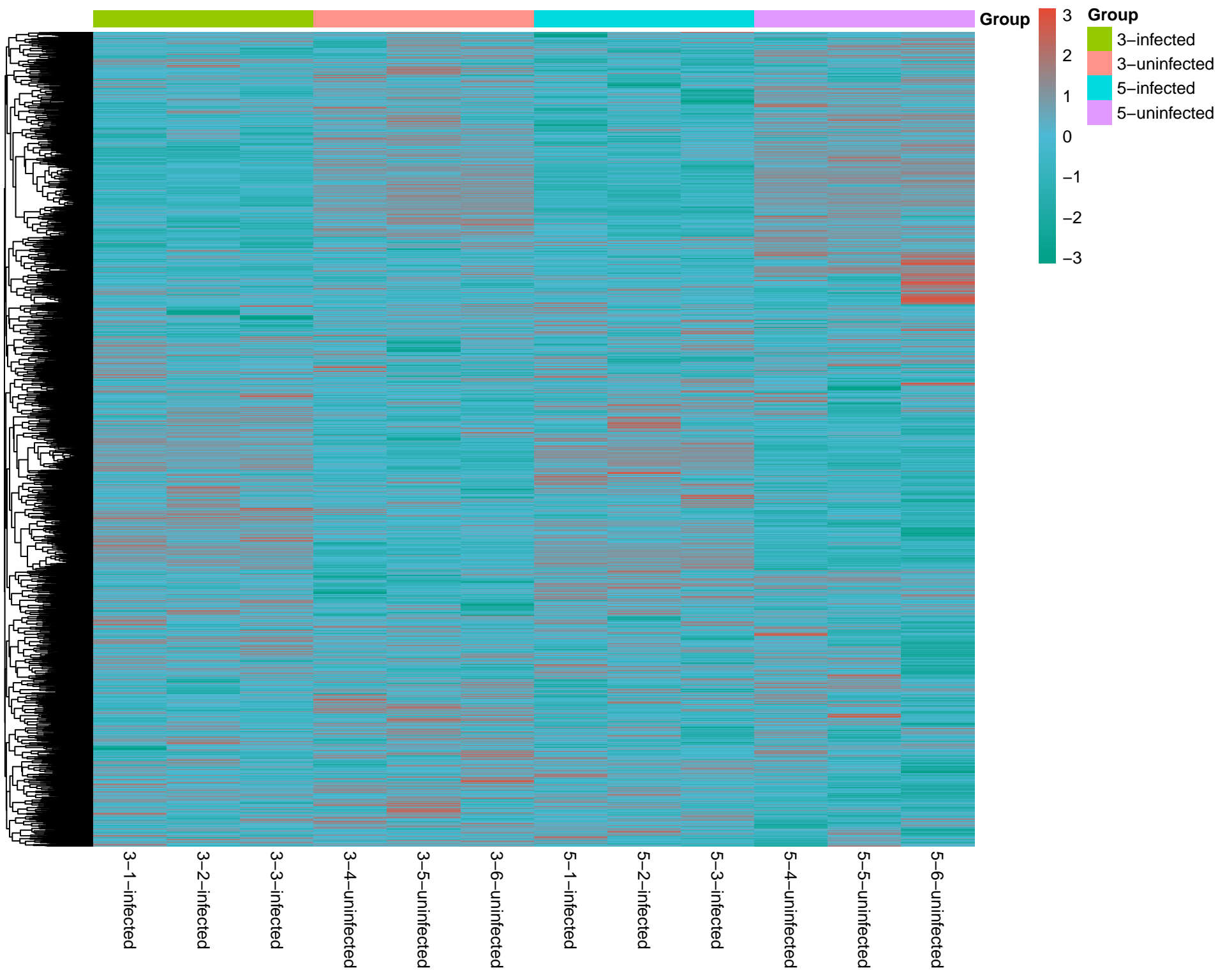

Supplement: Supplementary file 1 [file vaccines-12-00991-s001.zip › Supplementary File S3/proteome/2.Quantification/stats/ly_cluster_heatmap.pdf]

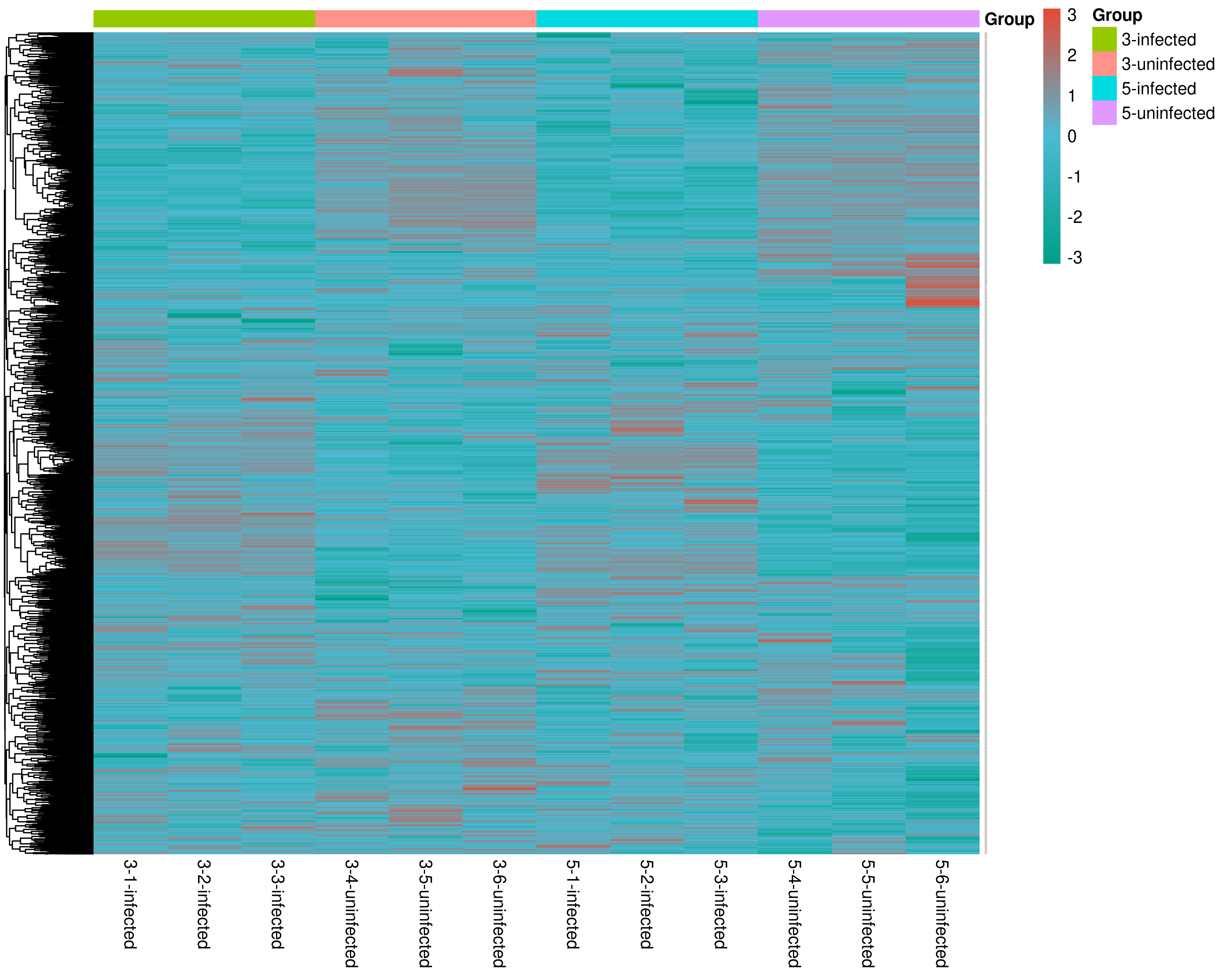

Supplement: Supplementary file 1 [file vaccines-12-00991-s001.zip › Supplementary File S3/proteome/2.Quantification/stats/ly_cluster_heatmap.png]

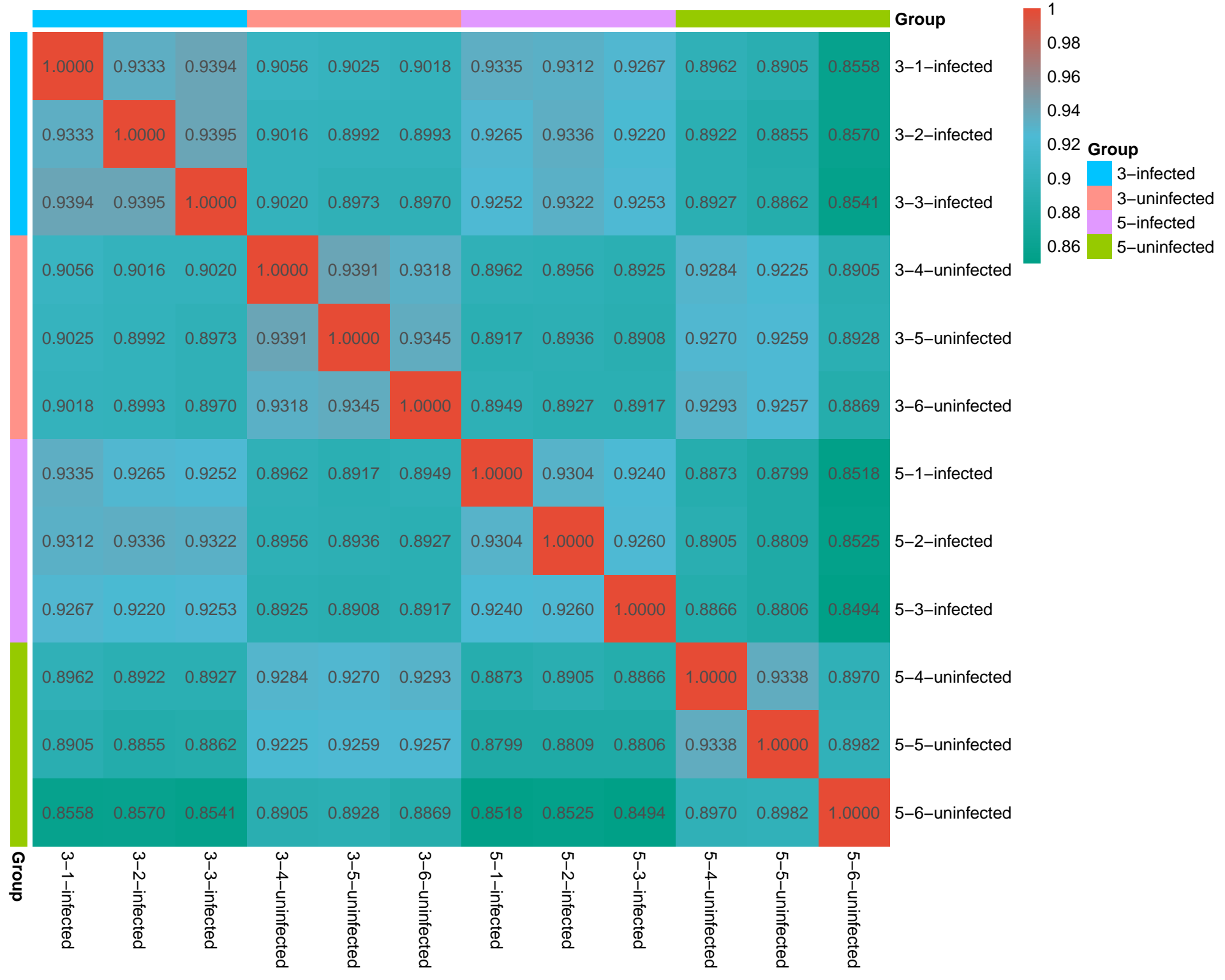

Supplement: Supplementary file 1 [file vaccines-12-00991-s001.zip › Supplementary File S3/proteome/2.Quantification/stats/ly_correlation_heatmap.pdf]

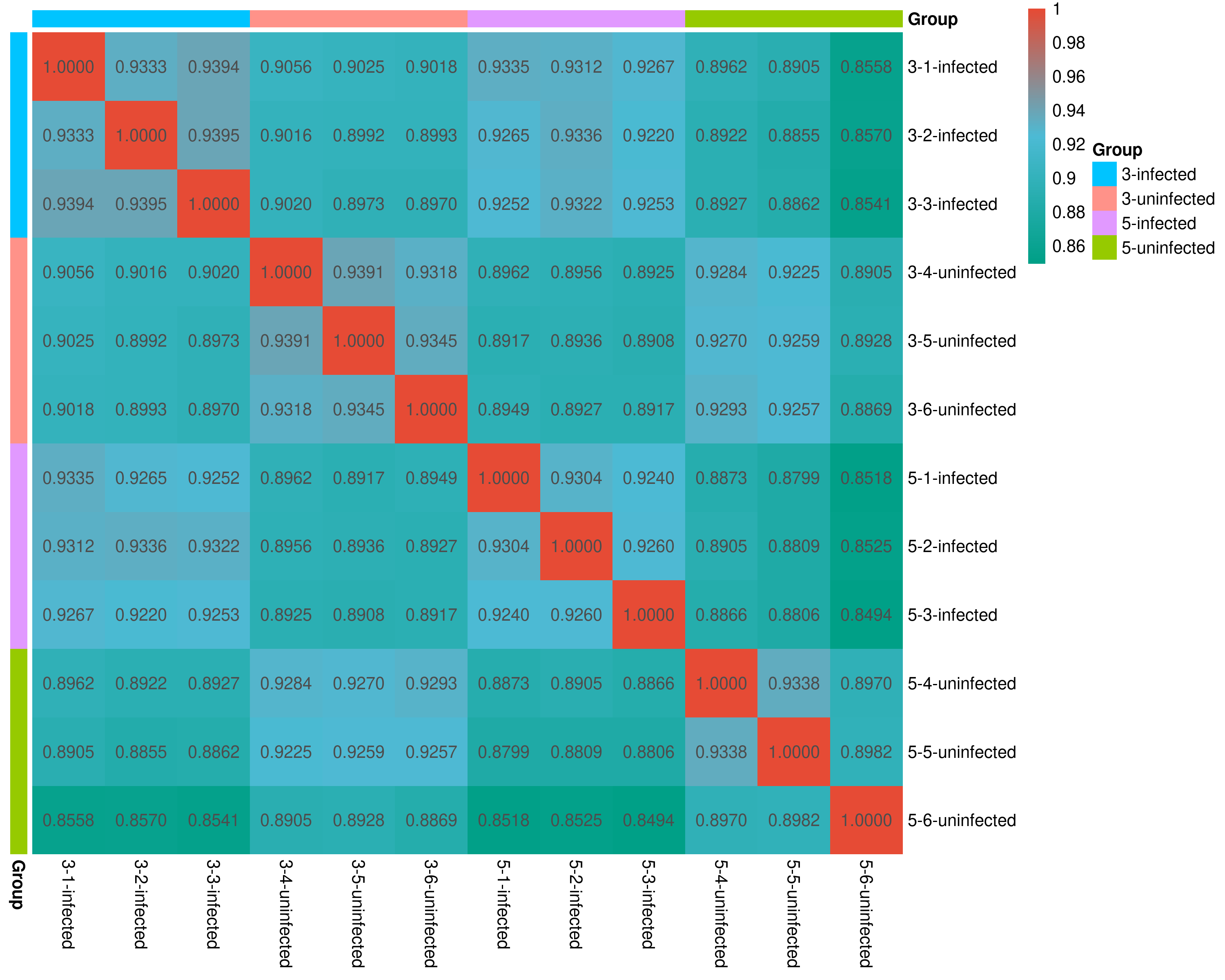

Supplement: Supplementary file 1 [file vaccines-12-00991-s001.zip › Supplementary File S3/proteome/2.Quantification/stats/ly_correlation_heatmap.png]

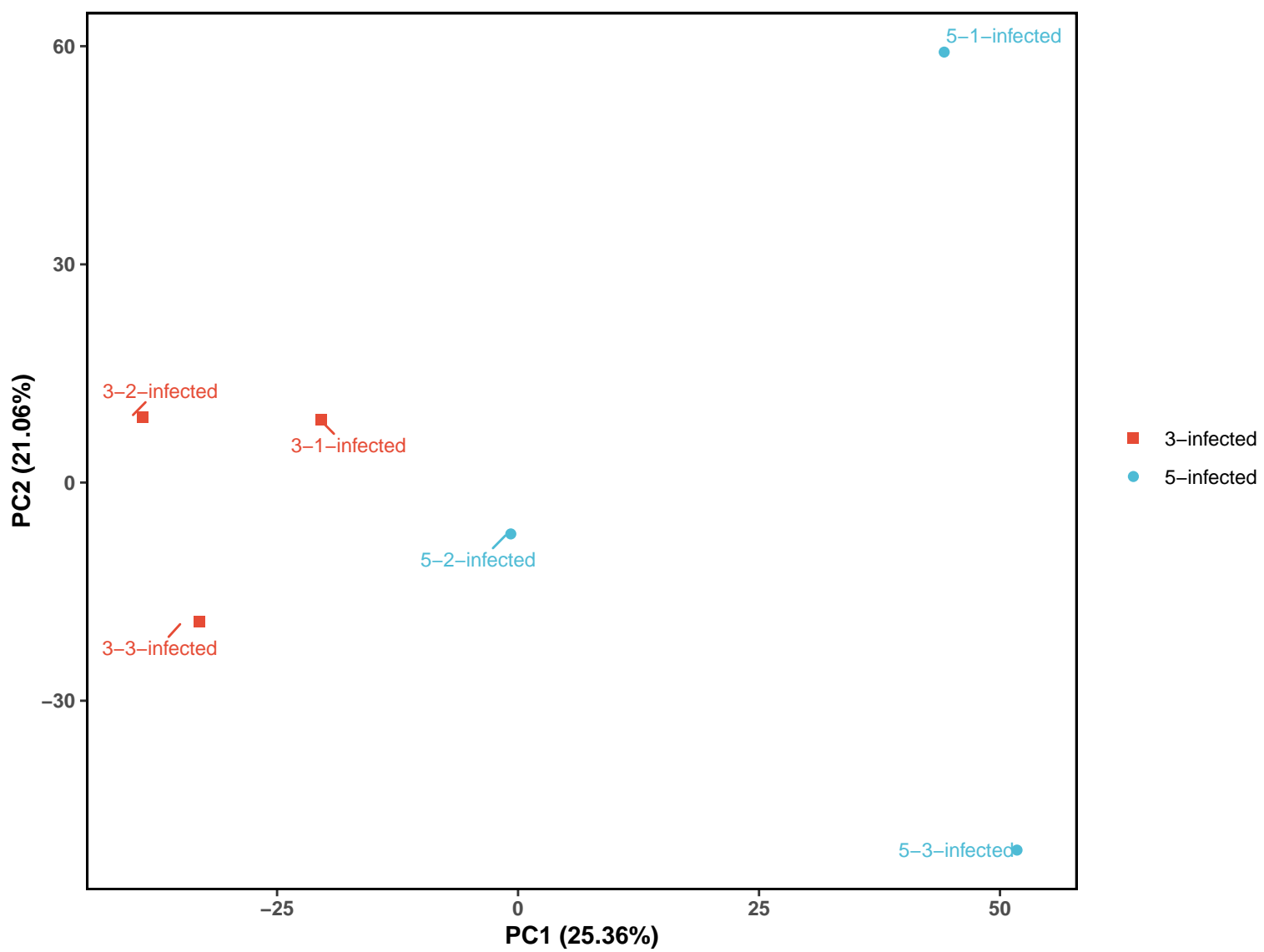

Supplement: Supplementary file 1 [file vaccines-12-00991-s001.zip › Supplementary File S3/proteome/3.Difference/compare/3-infected_vs_5-infected/3-infected_vs_5-infected_2d_pca.pdf]

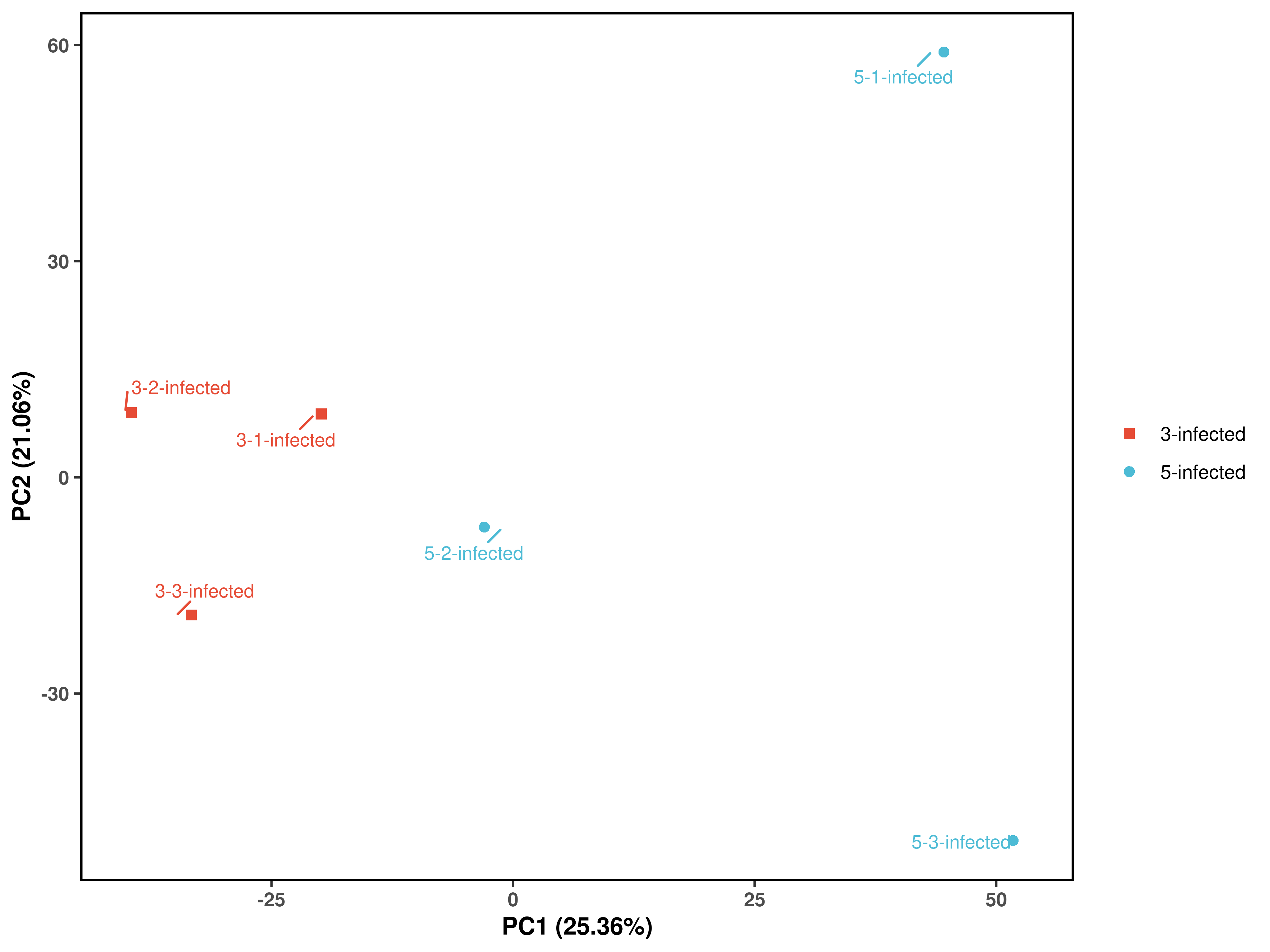

Supplement: Supplementary file 1 [file vaccines-12-00991-s001.zip › Supplementary File S3/proteome/3.Difference/compare/3-infected_vs_5-infected/3-infected_vs_5-infected_2d_pca.png]

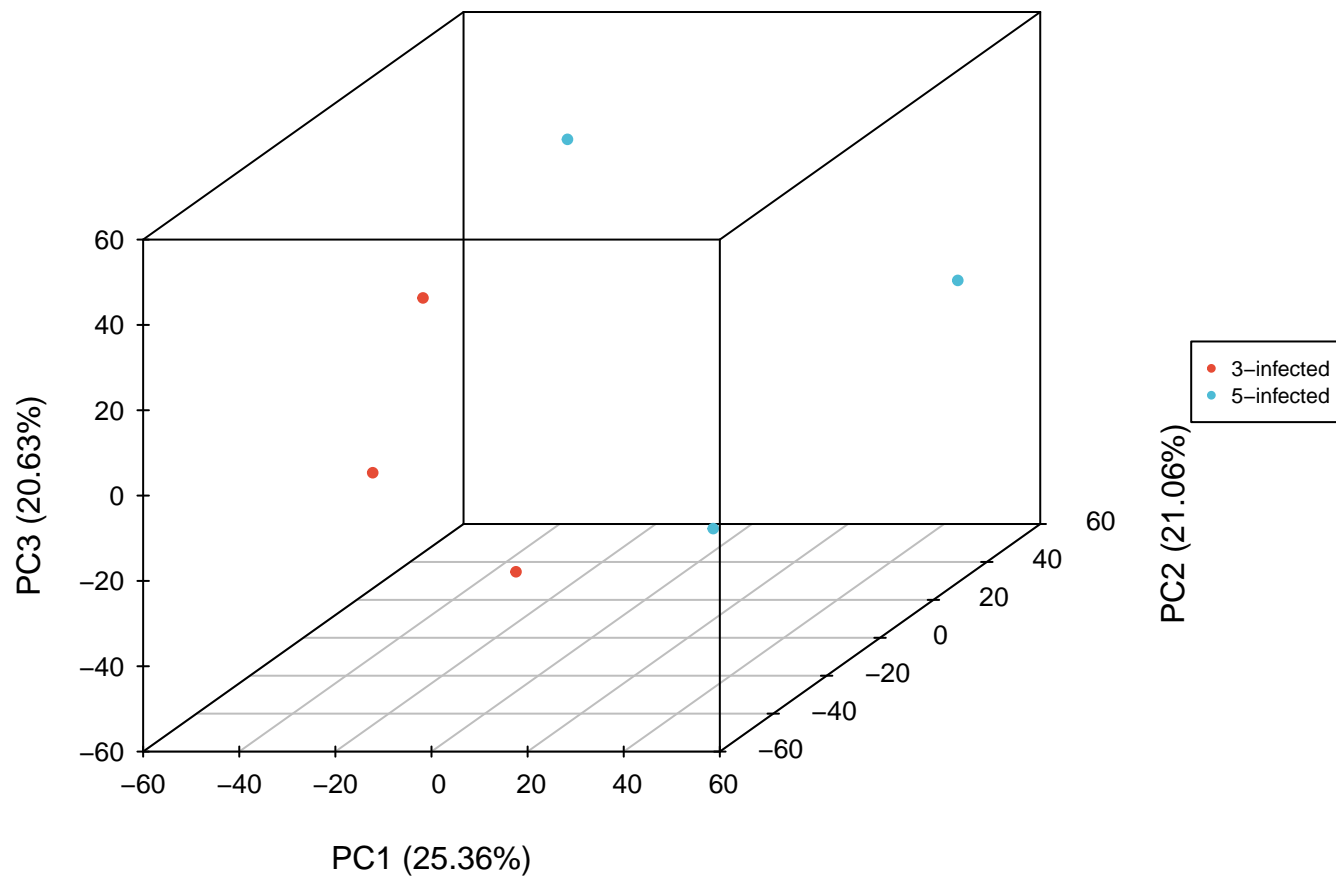

Supplement: Supplementary file 1 [file vaccines-12-00991-s001.zip › Supplementary File S3/proteome/3.Difference/compare/3-infected_vs_5-infected/3-infected_vs_5-infected_3d_pca.pdf]

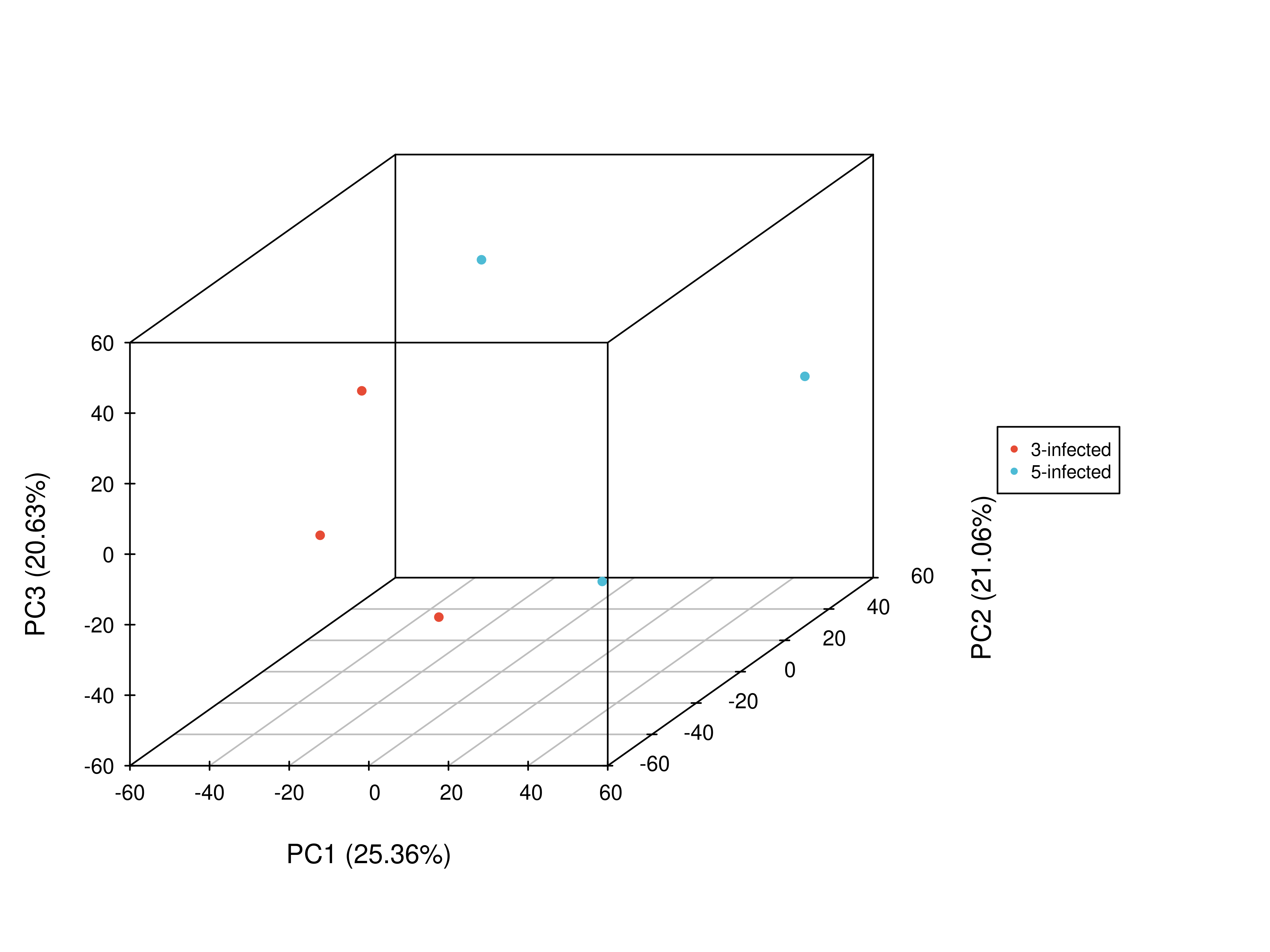

Supplement: Supplementary file 1 [file vaccines-12-00991-s001.zip › Supplementary File S3/proteome/3.Difference/compare/3-infected_vs_5-infected/3-infected_vs_5-infected_3d_pca.png]

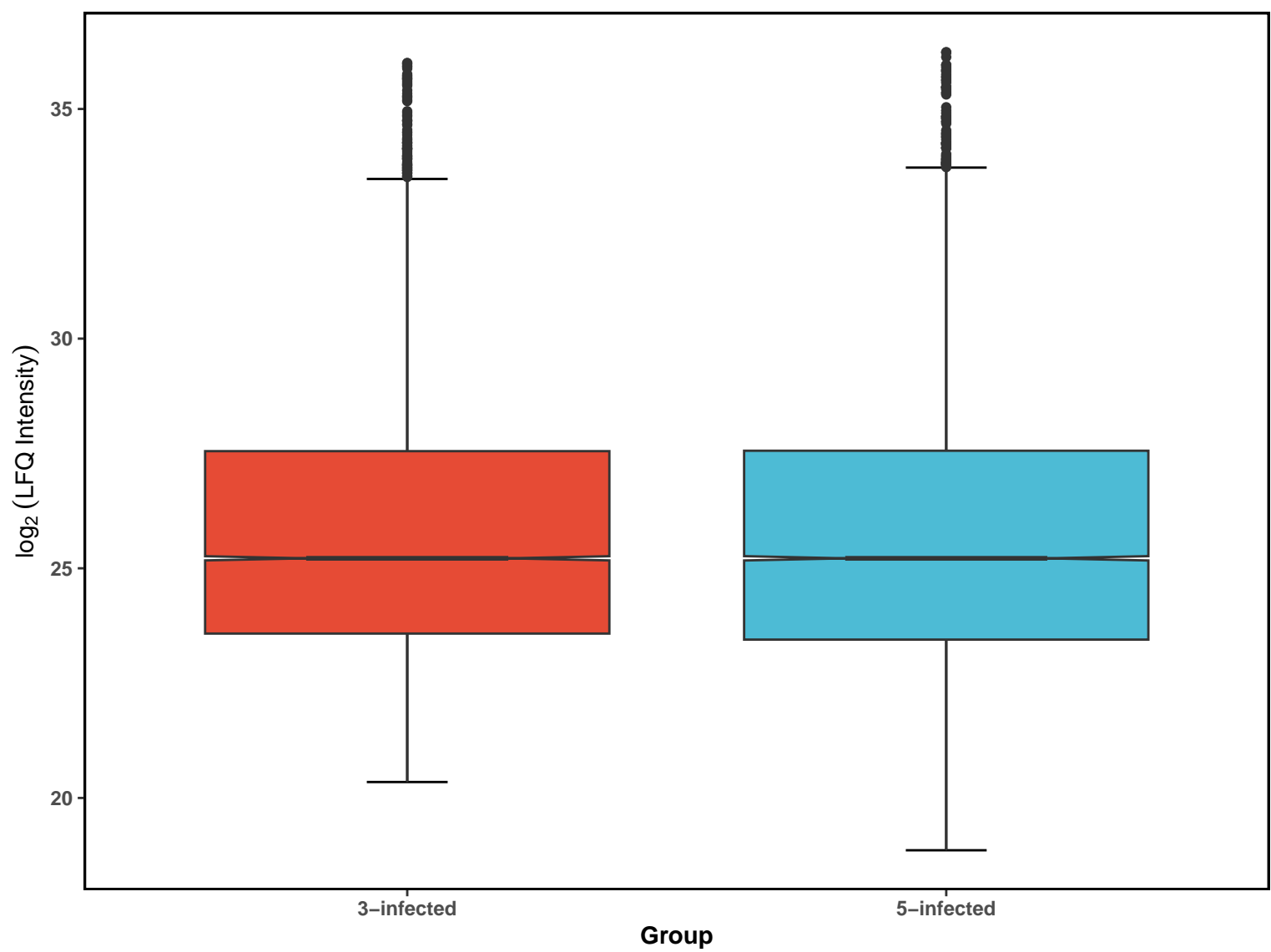

Supplement: Supplementary file 1 [file vaccines-12-00991-s001.zip › Supplementary File S3/proteome/3.Difference/compare/3-infected_vs_5-infected/3-infected_vs_5-infected_boxplot.pdf]

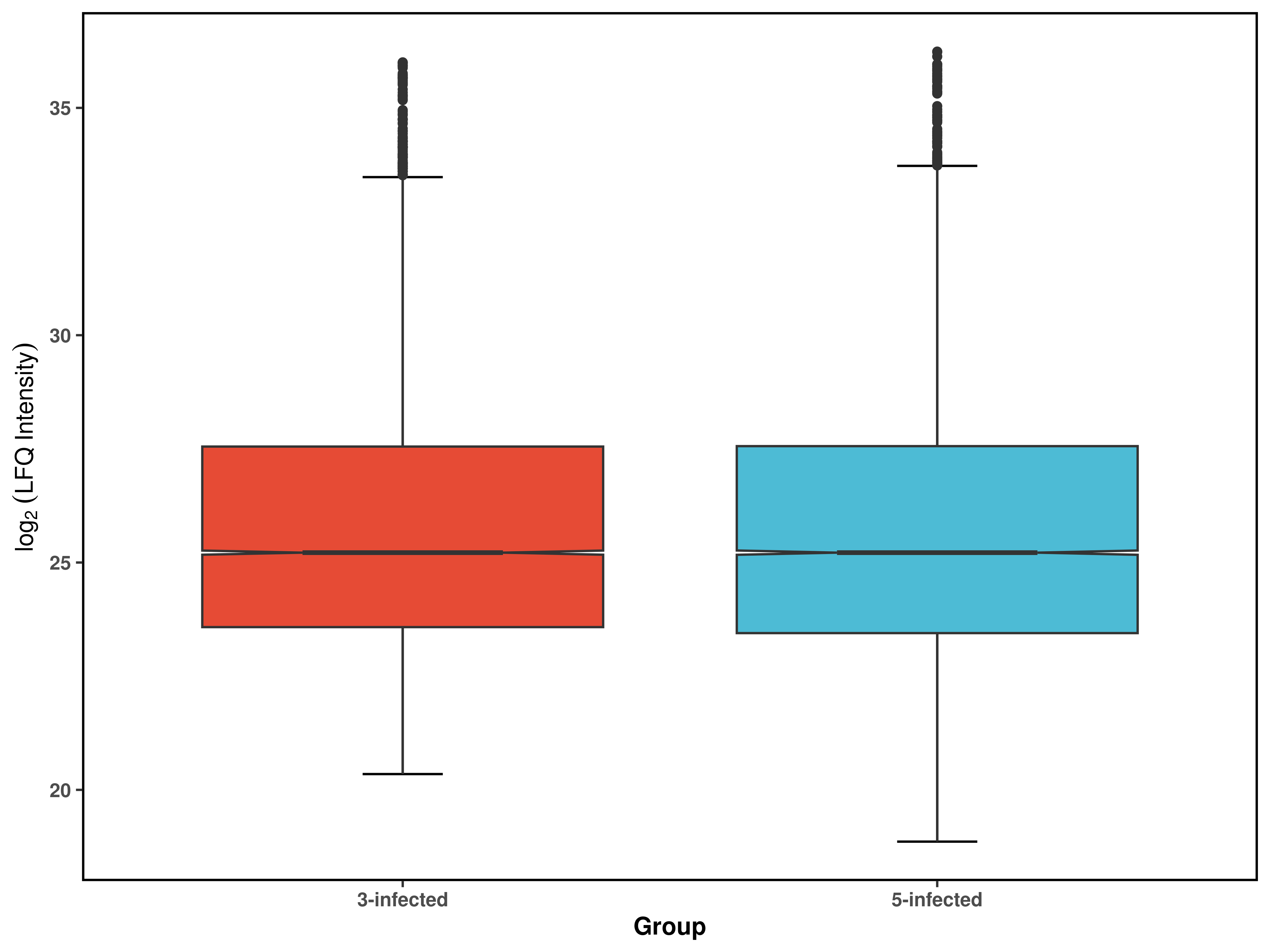

Supplement: Supplementary file 1 [file vaccines-12-00991-s001.zip › Supplementary File S3/proteome/3.Difference/compare/3-infected_vs_5-infected/3-infected_vs_5-infected_boxplot.png]

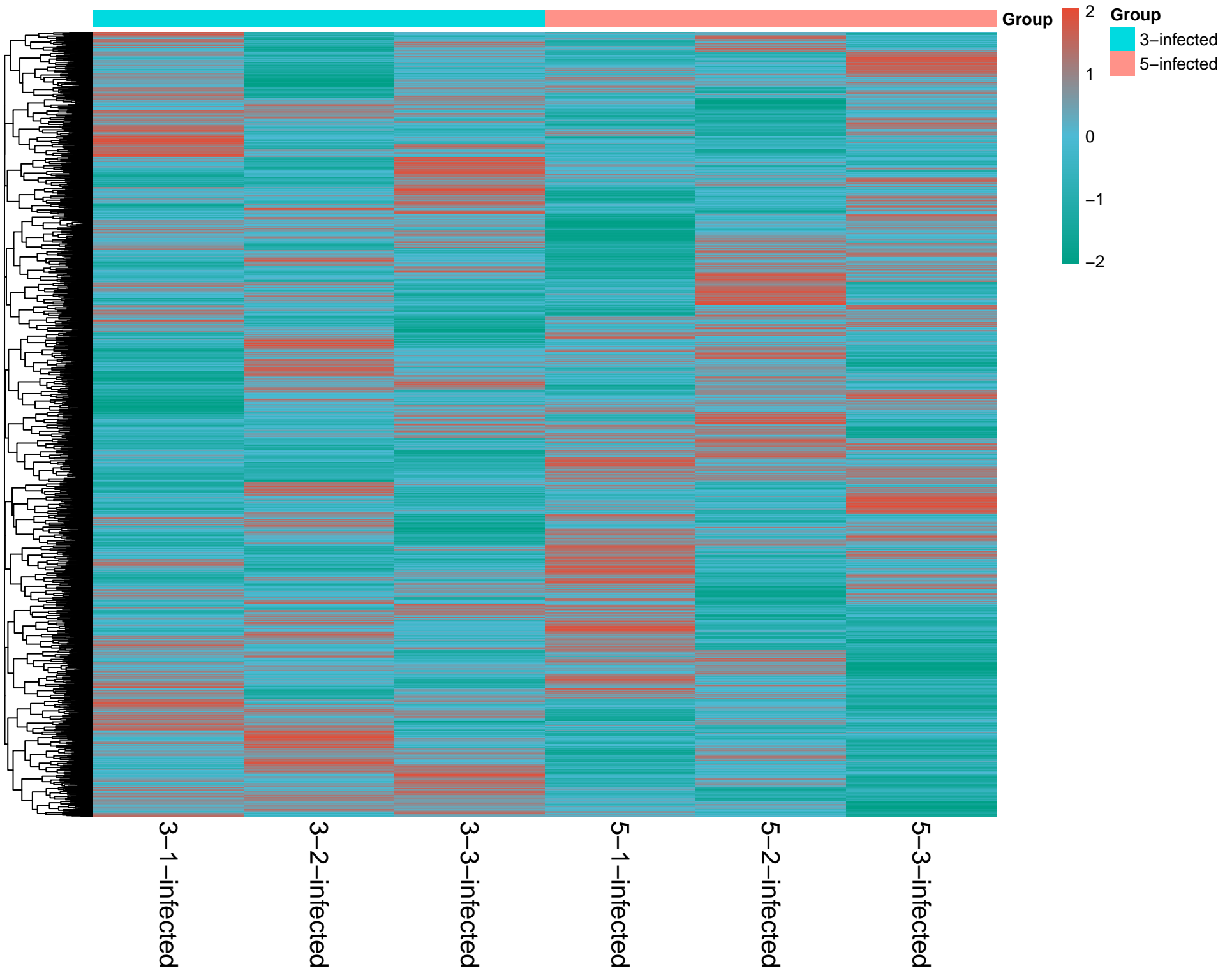

Supplement: Supplementary file 1 [file vaccines-12-00991-s001.zip › Supplementary File S3/proteome/3.Difference/compare/3-infected_vs_5-infected/3-infected_vs_5-infected_cluster_heatmap.pdf]

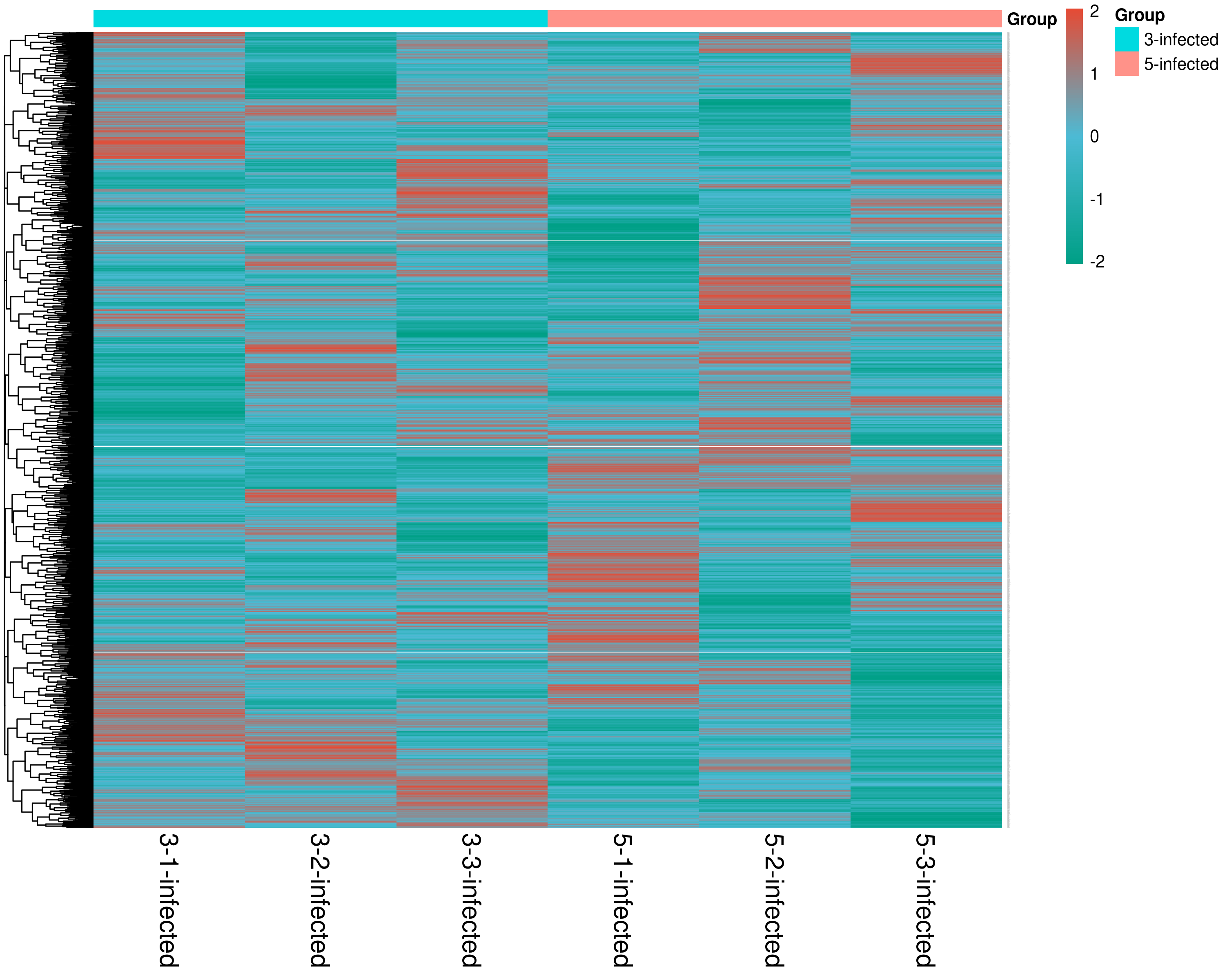

Supplement: Supplementary file 1 [file vaccines-12-00991-s001.zip › Supplementary File S3/proteome/3.Difference/compare/3-infected_vs_5-infected/3-infected_vs_5-infected_cluster_heatmap.png]

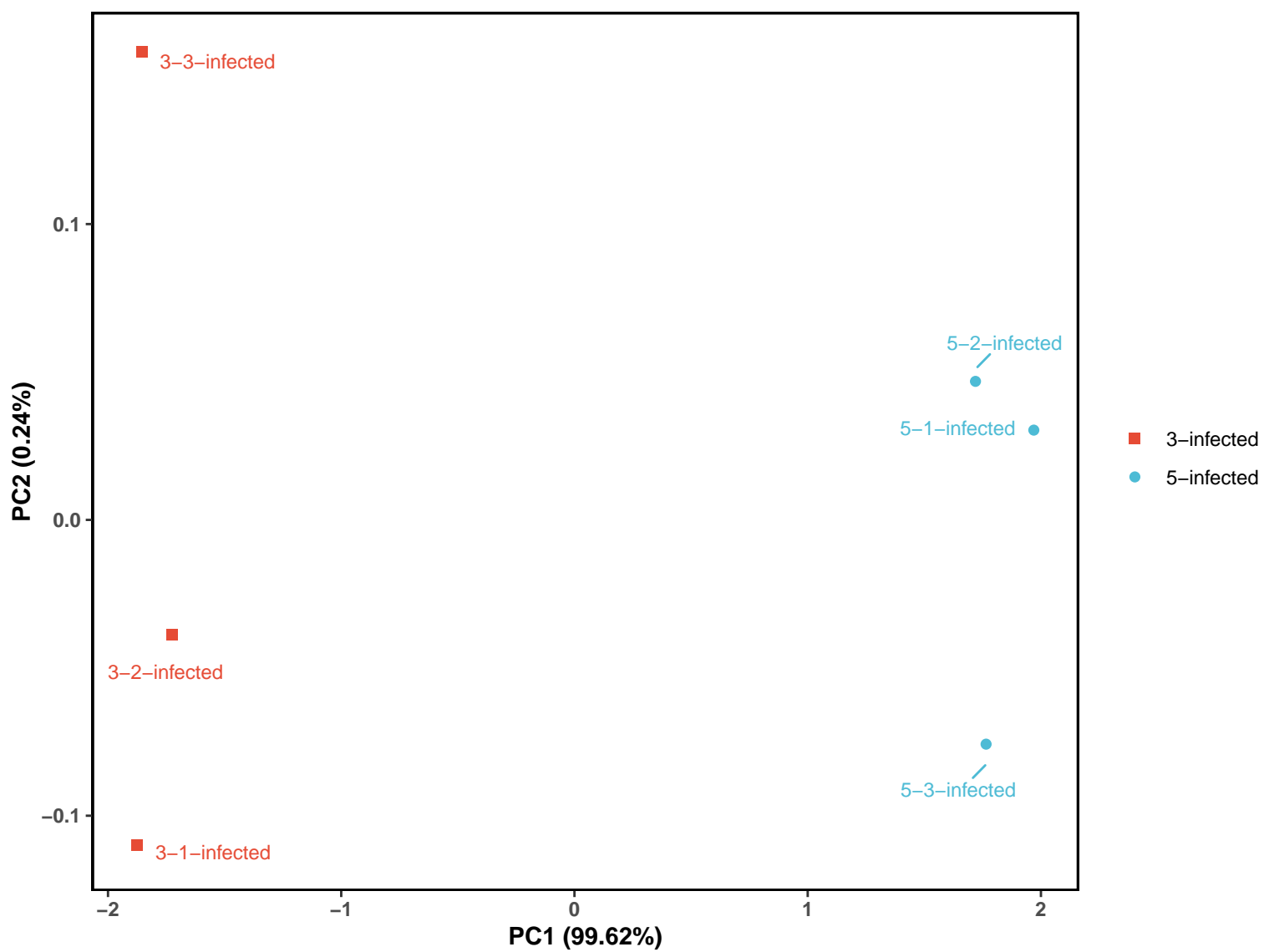

Supplement: Supplementary file 1 [file vaccines-12-00991-s001.zip › Supplementary File S3/proteome/3.Difference/compare/3-infected_vs_5-infected/3-infected_vs_5-infected_diff_2d_pca.pdf]

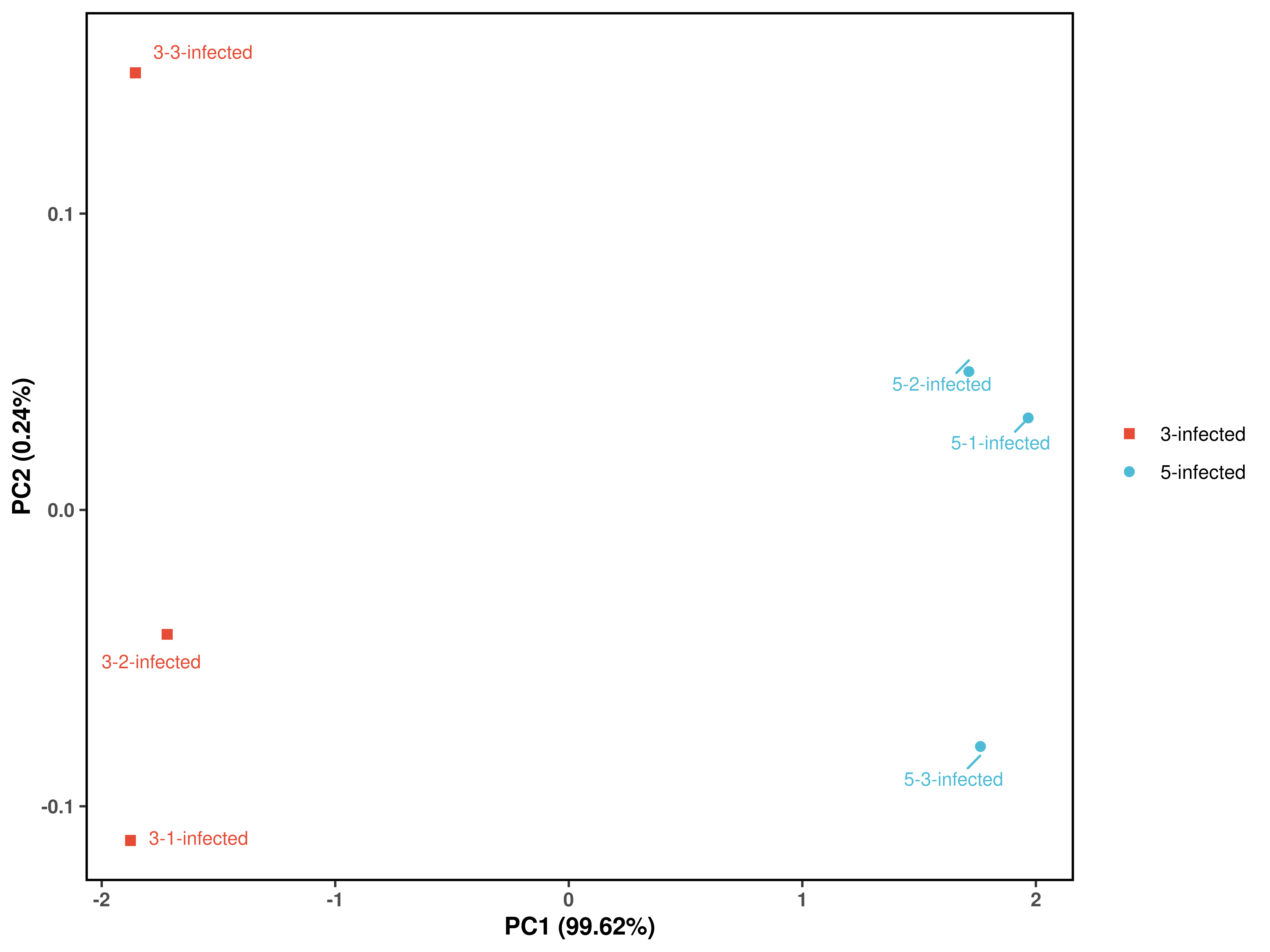

Supplement: Supplementary file 1 [file vaccines-12-00991-s001.zip › Supplementary File S3/proteome/3.Difference/compare/3-infected_vs_5-infected/3-infected_vs_5-infected_diff_2d_pca.png]

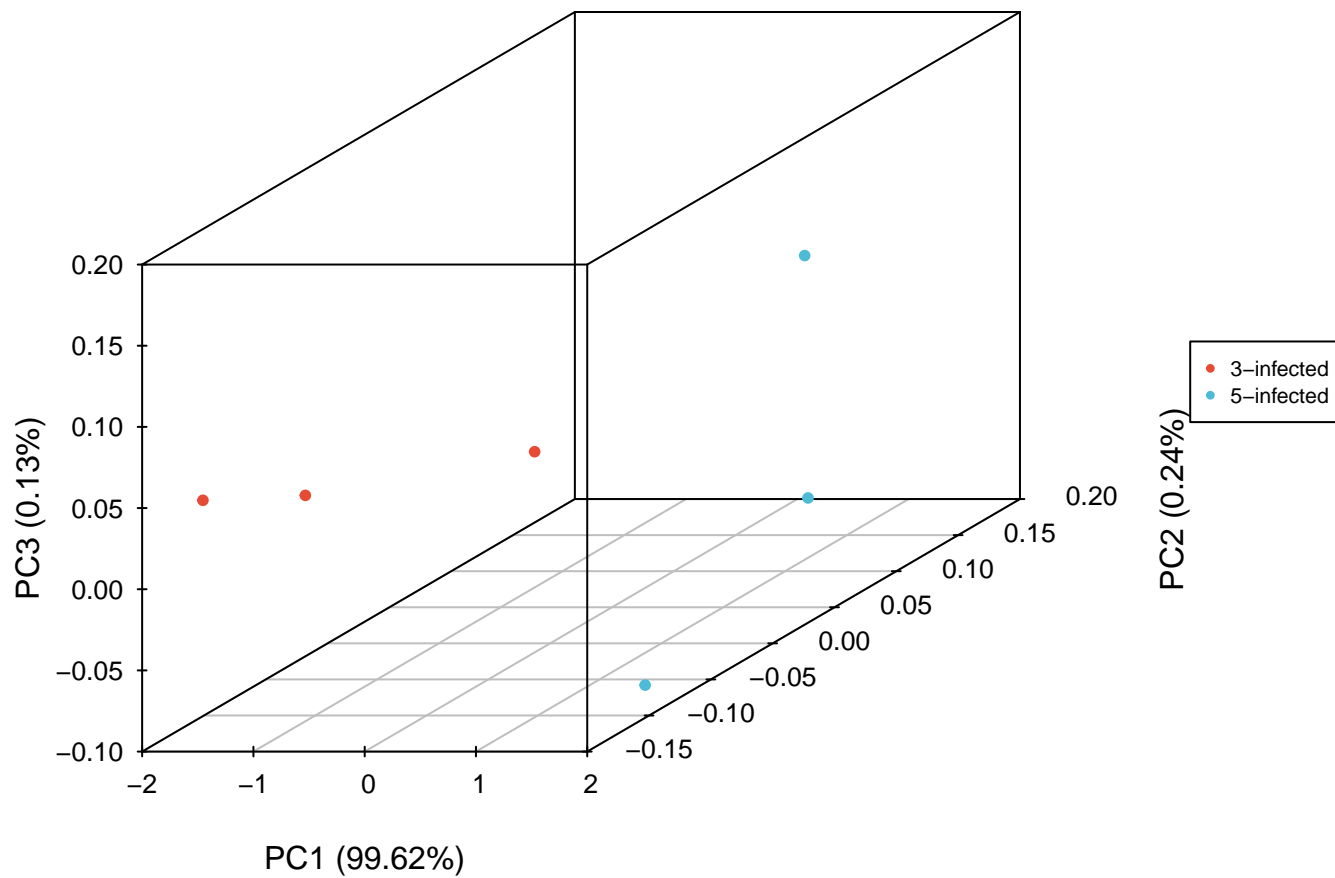

Supplement: Supplementary file 1 [file vaccines-12-00991-s001.zip › Supplementary File S3/proteome/3.Difference/compare/3-infected_vs_5-infected/3-infected_vs_5-infected_diff_3d_pca.pdf]

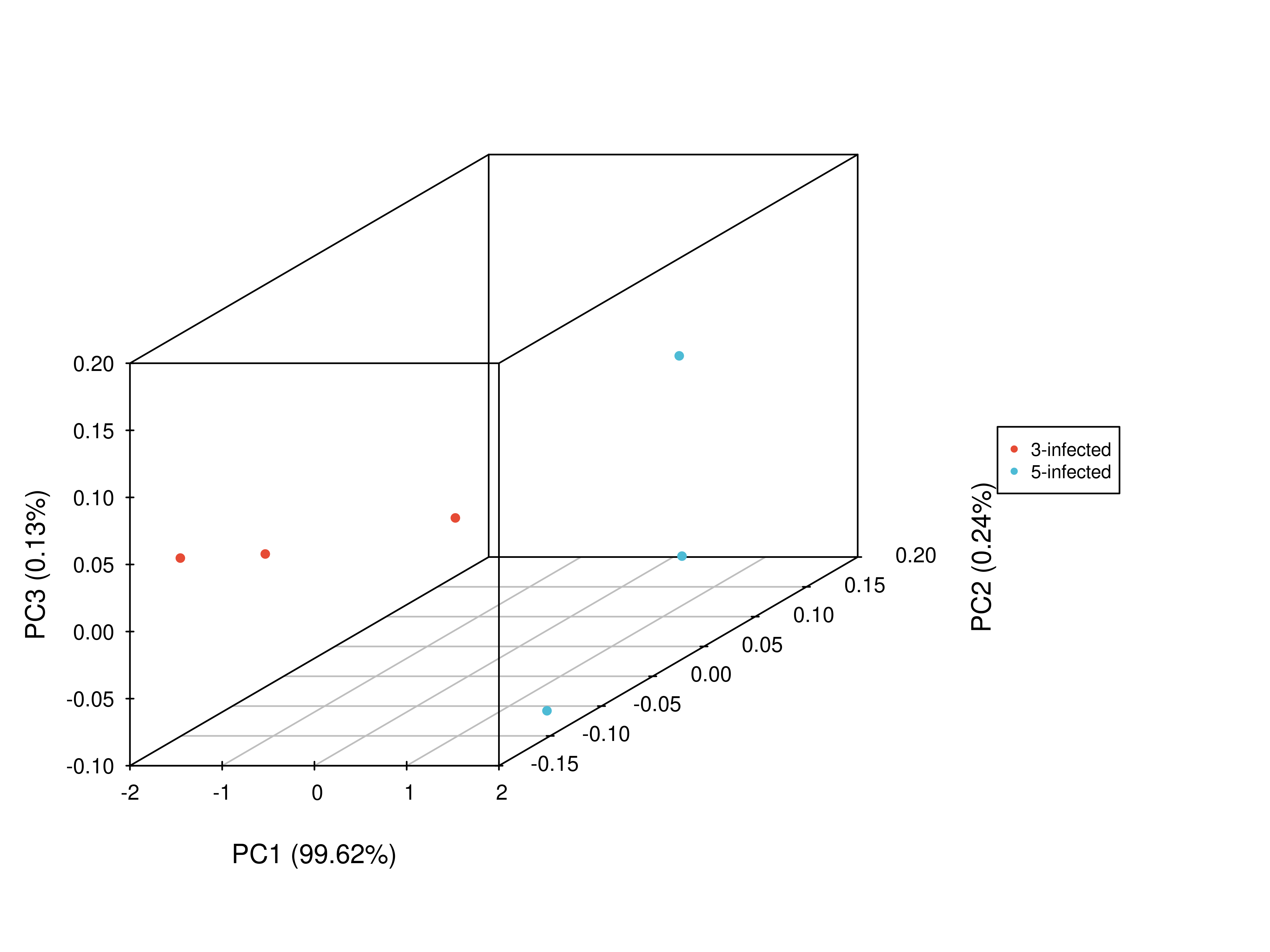

Supplement: Supplementary file 1 [file vaccines-12-00991-s001.zip › Supplementary File S3/proteome/3.Difference/compare/3-infected_vs_5-infected/3-infected_vs_5-infected_diff_3d_pca.png]

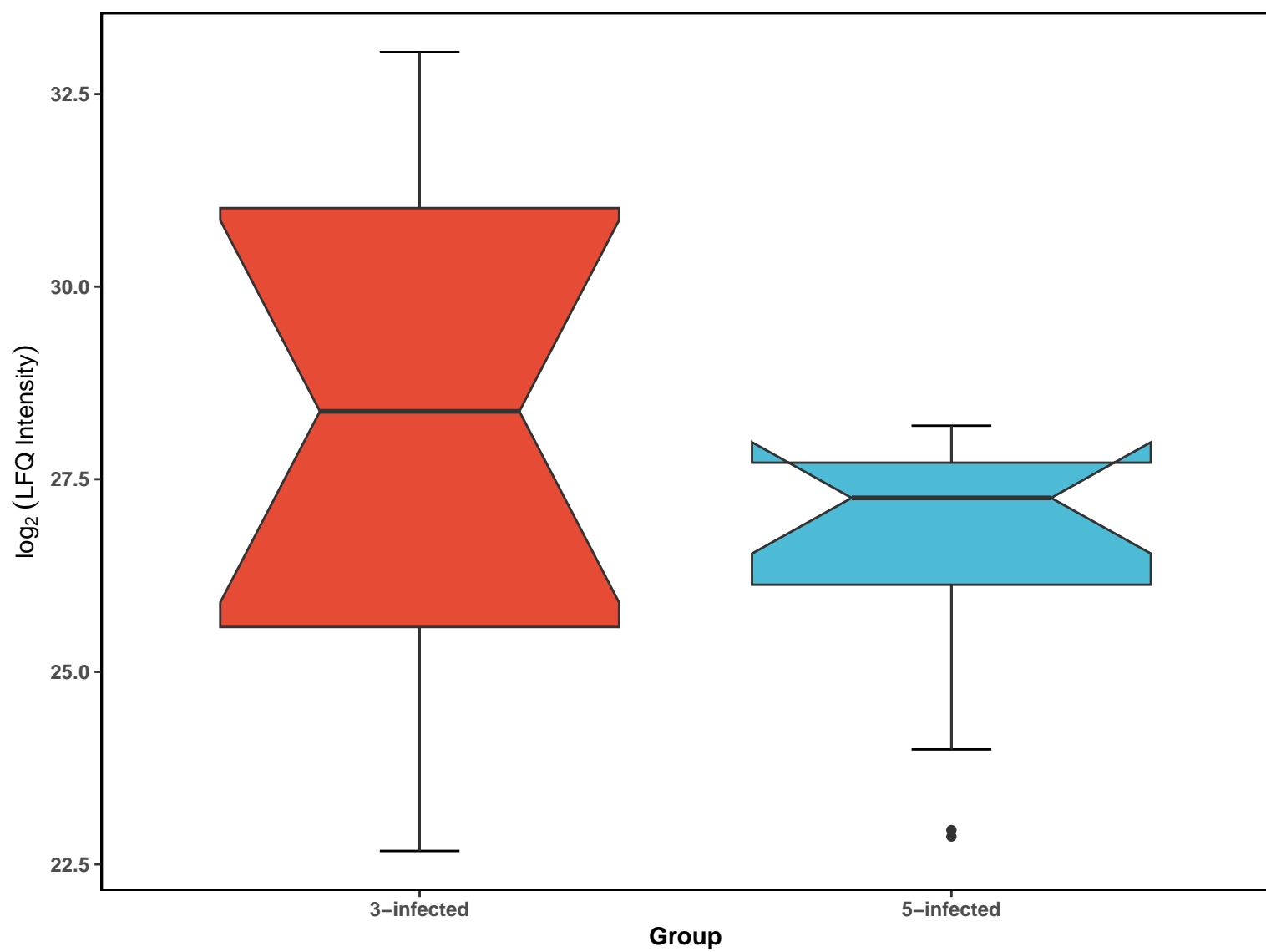

Supplement: Supplementary file 1 [file vaccines-12-00991-s001.zip › Supplementary File S3/proteome/3.Difference/compare/3-infected_vs_5-infected/3-infected_vs_5-infected_diff_boxplot.pdf]

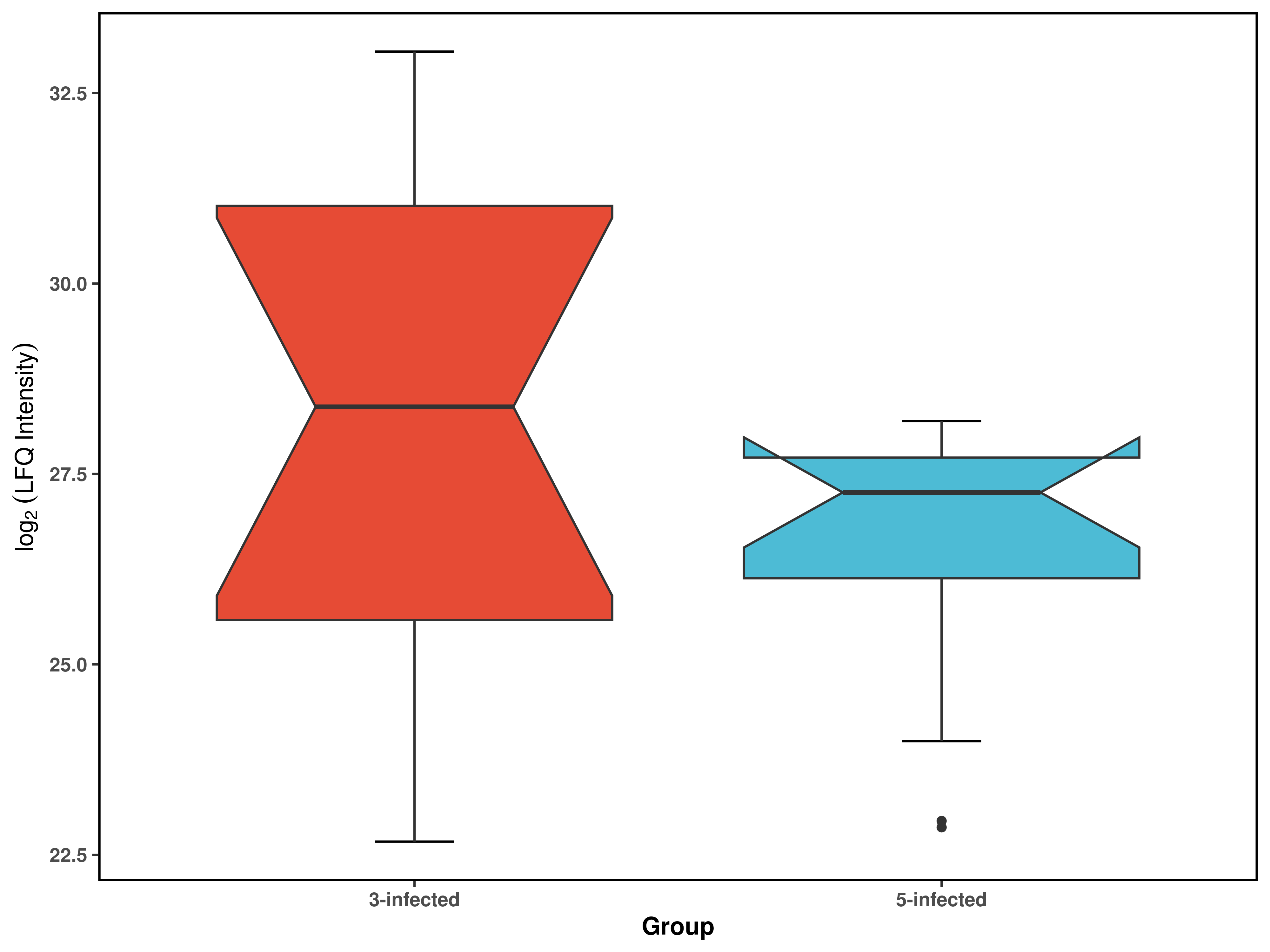

Supplement: Supplementary file 1 [file vaccines-12-00991-s001.zip › Supplementary File S3/proteome/3.Difference/compare/3-infected_vs_5-infected/3-infected_vs_5-infected_diff_boxplot.png]

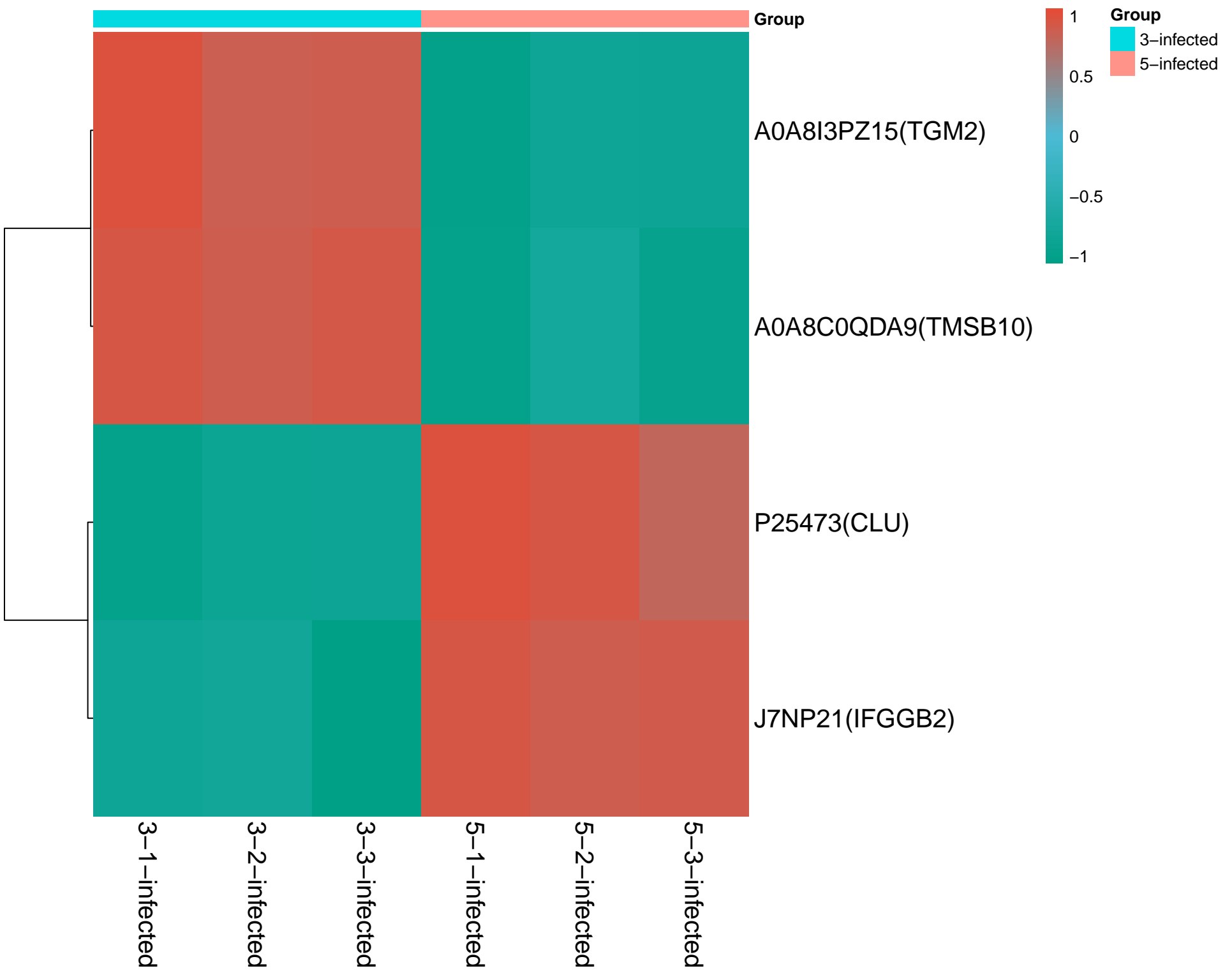

Supplement: Supplementary file 1 [file vaccines-12-00991-s001.zip › Supplementary File S3/proteome/3.Difference/compare/3-infected_vs_5-infected/3-infected_vs_5-infected_diff_cluster_heatmap.pdf]

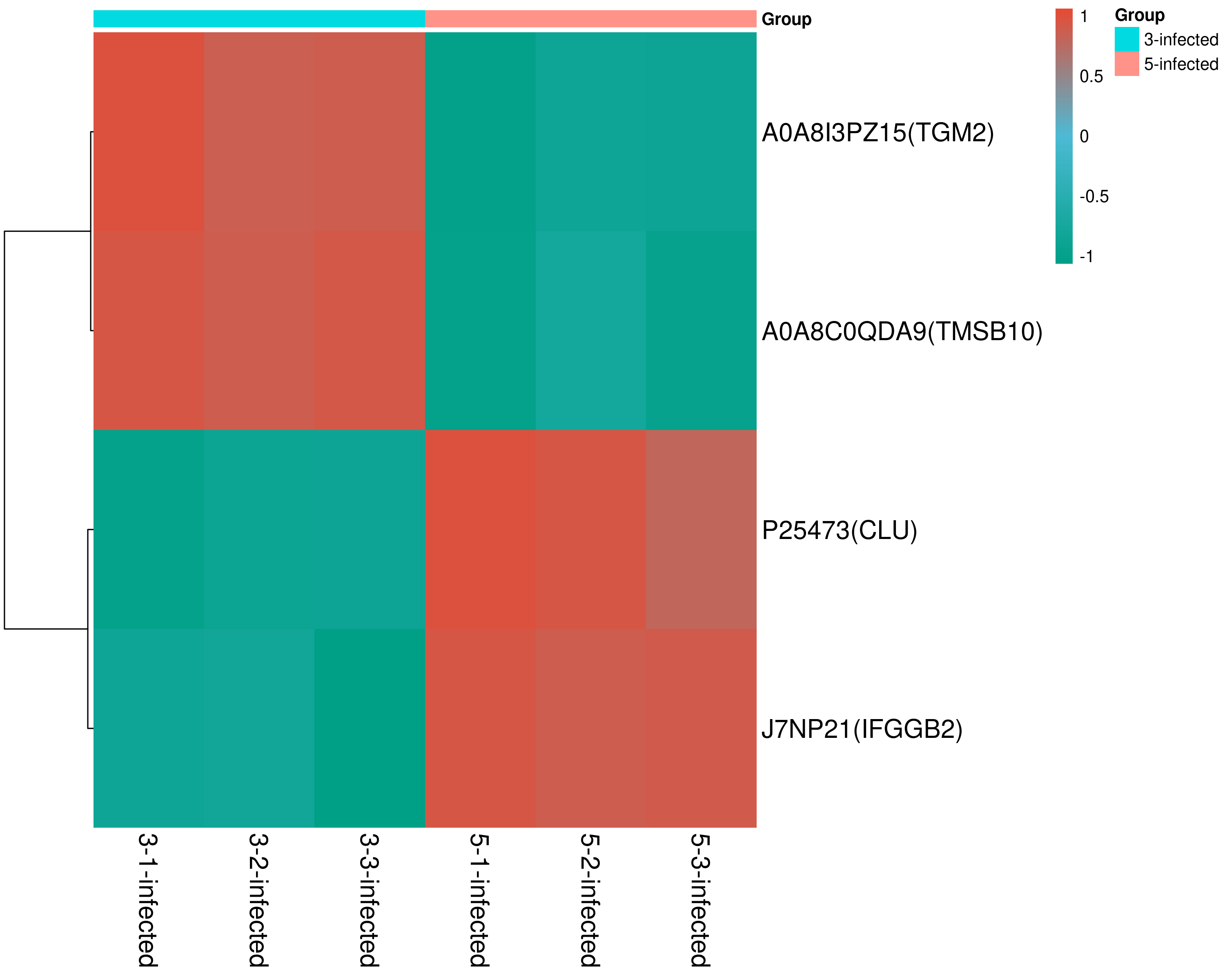

Supplement: Supplementary file 1 [file vaccines-12-00991-s001.zip › Supplementary File S3/proteome/3.Difference/compare/3-infected_vs_5-infected/3-infected_vs_5-infected_diff_cluster_heatmap.png]

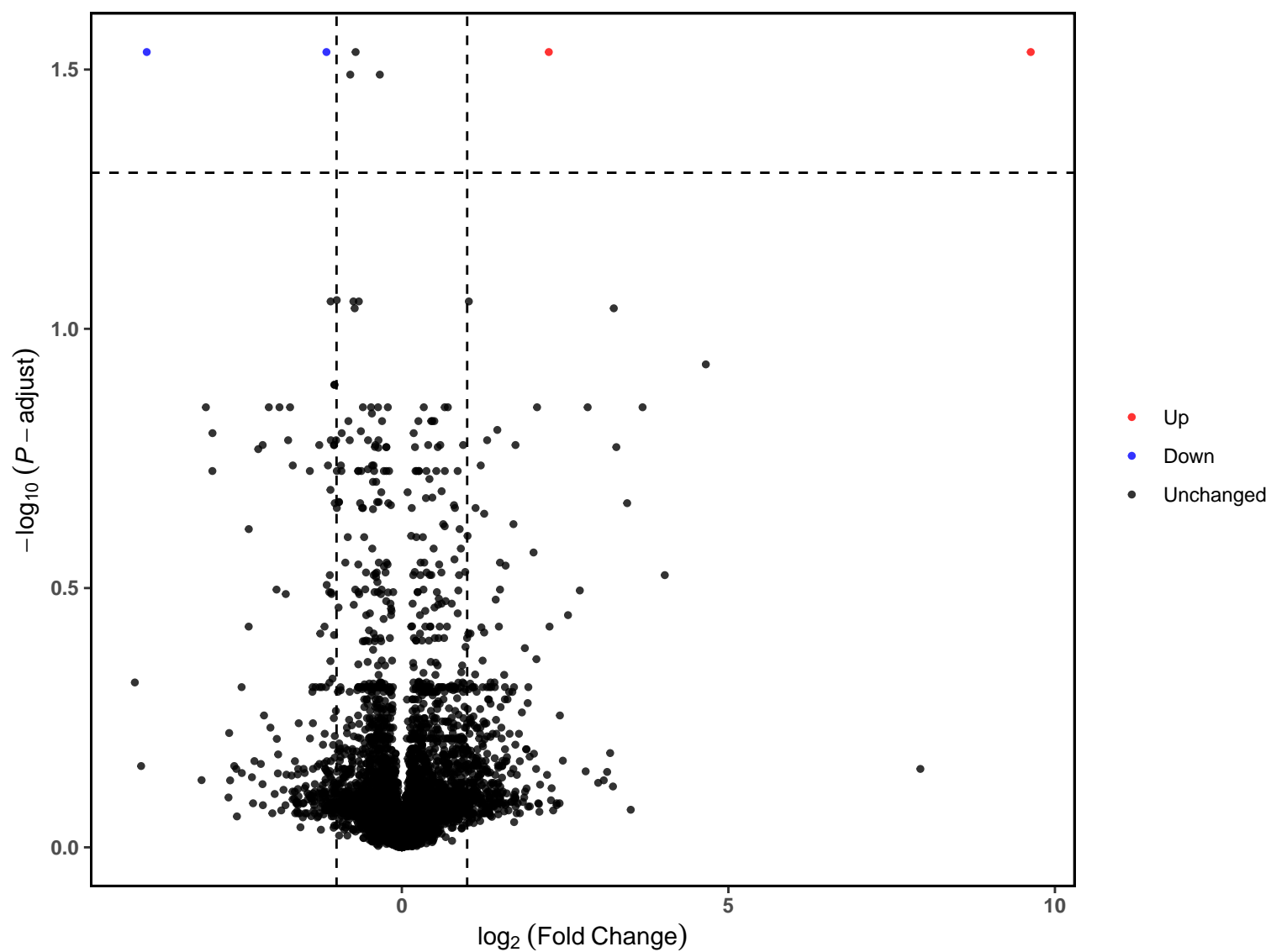

Supplement: Supplementary file 1 [file vaccines-12-00991-s001.zip › Supplementary File S3/proteome/3.Difference/compare/3-infected_vs_5-infected/3-infected_vs_5-infected_volcano.pdf]

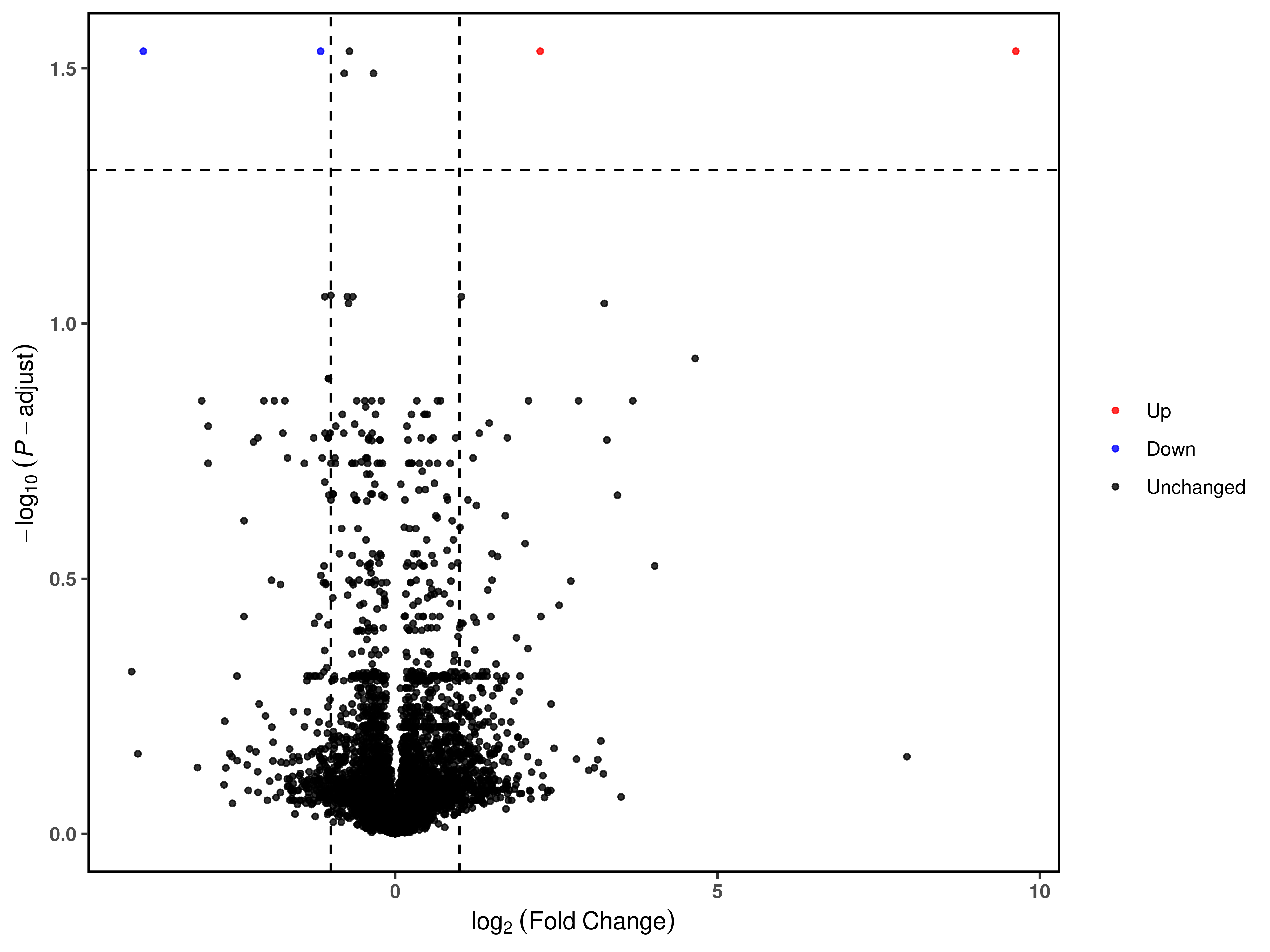

Supplement: Supplementary file 1 [file vaccines-12-00991-s001.zip › Supplementary File S3/proteome/3.Difference/compare/3-infected_vs_5-infected/3-infected_vs_5-infected_volcano.png]

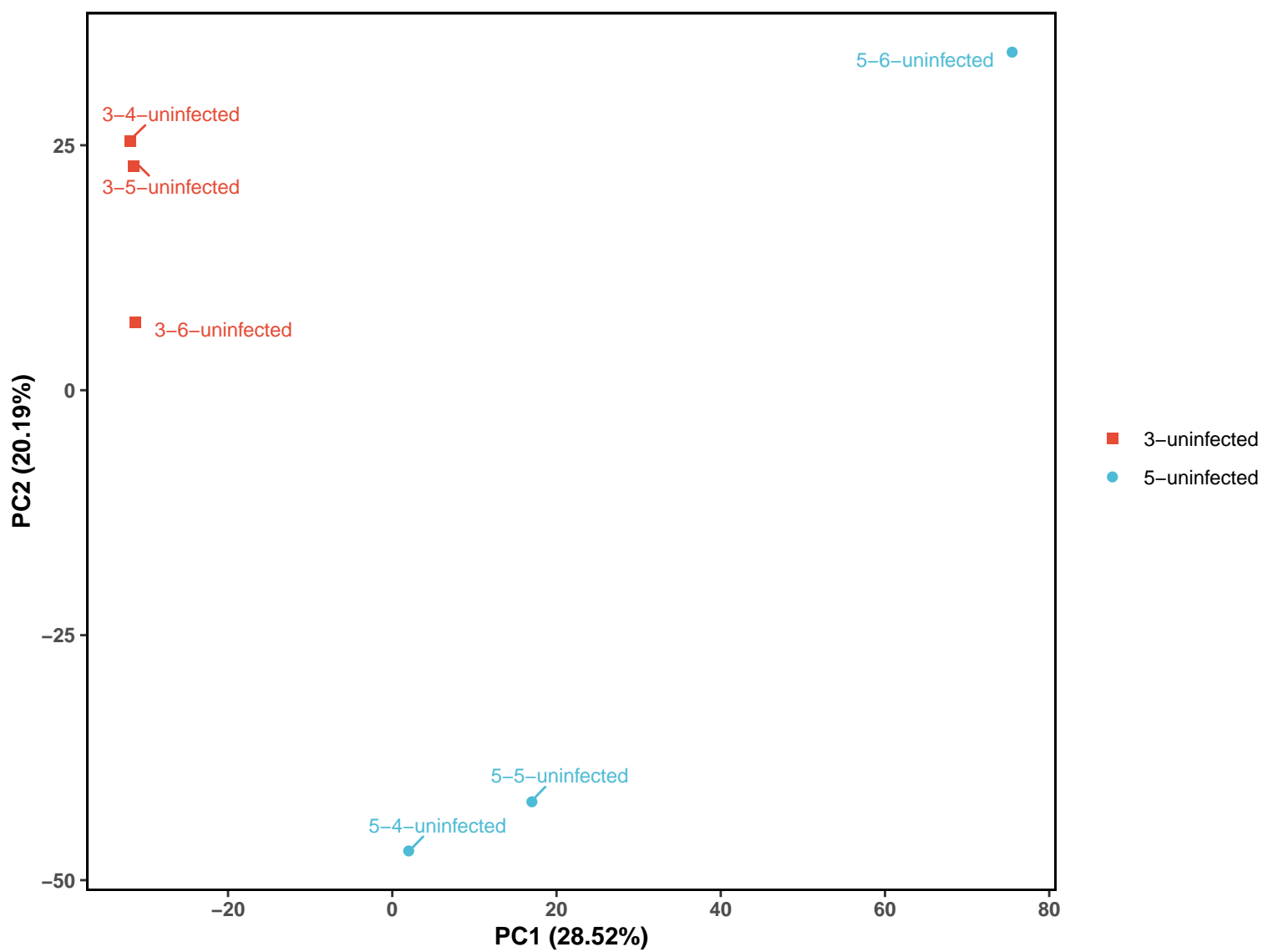

Supplement: Supplementary file 1 [file vaccines-12-00991-s001.zip › Supplementary File S3/proteome/3.Difference/compare/3-uninfected_vs_5-uninfected/3-uninfected_vs_5-uninfected_2d_pca.pdf]

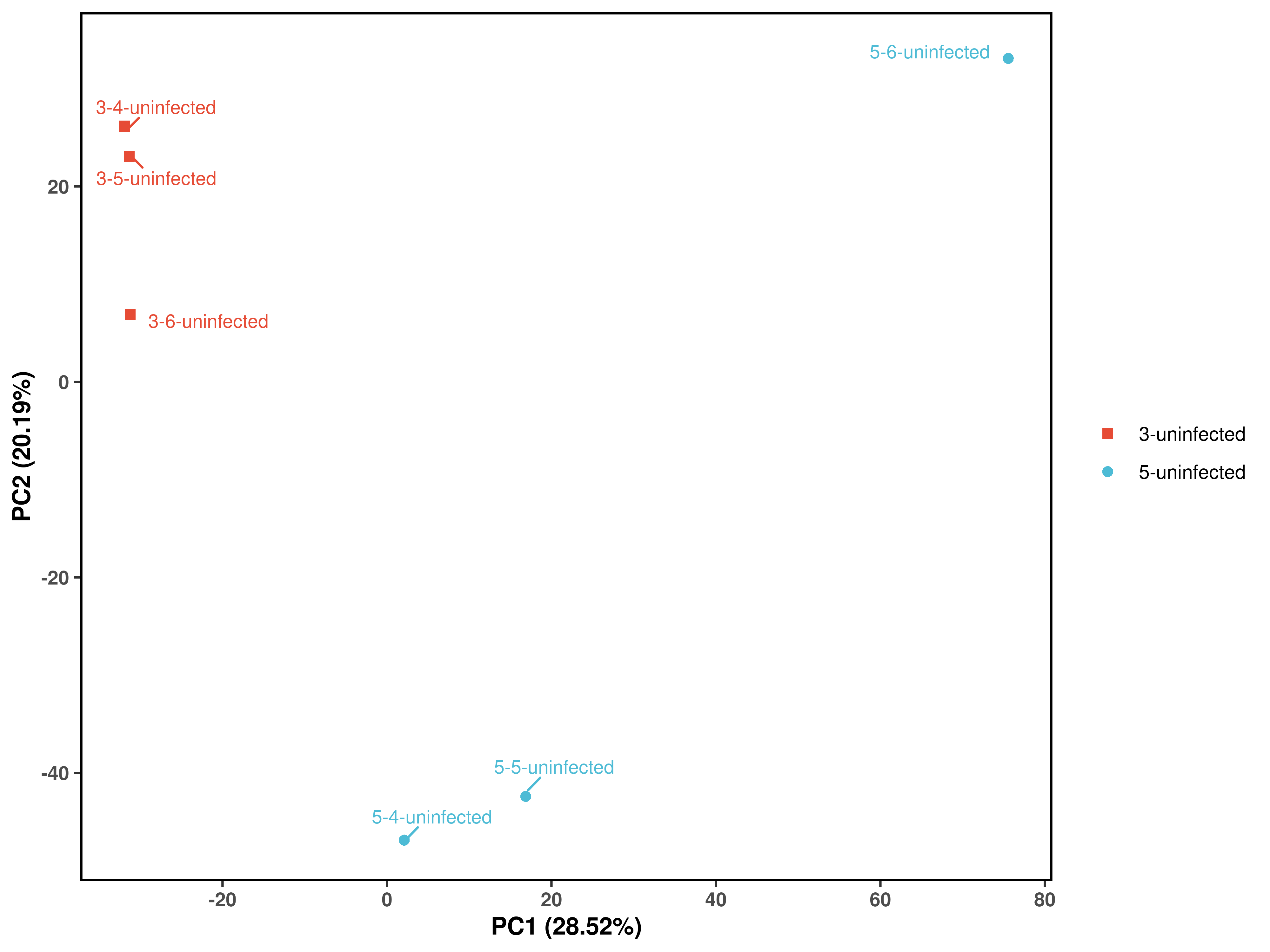

Supplement: Supplementary file 1 [file vaccines-12-00991-s001.zip › Supplementary File S3/proteome/3.Difference/compare/3-uninfected_vs_5-uninfected/3-uninfected_vs_5-uninfected_2d_pca.png]

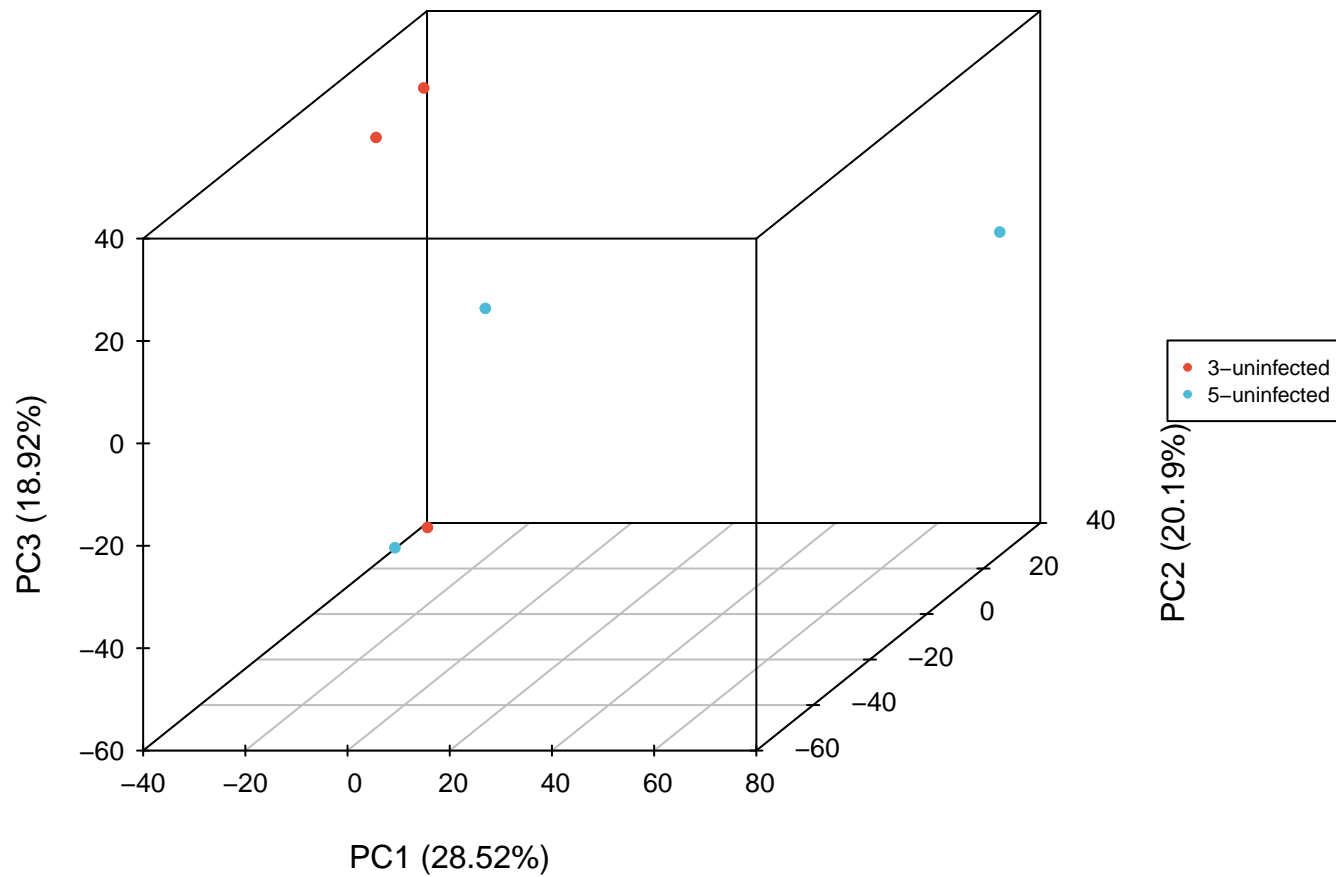

Supplement: Supplementary file 1 [file vaccines-12-00991-s001.zip › Supplementary File S3/proteome/3.Difference/compare/3-uninfected_vs_5-uninfected/3-uninfected_vs_5-uninfected_3d_pca.pdf]

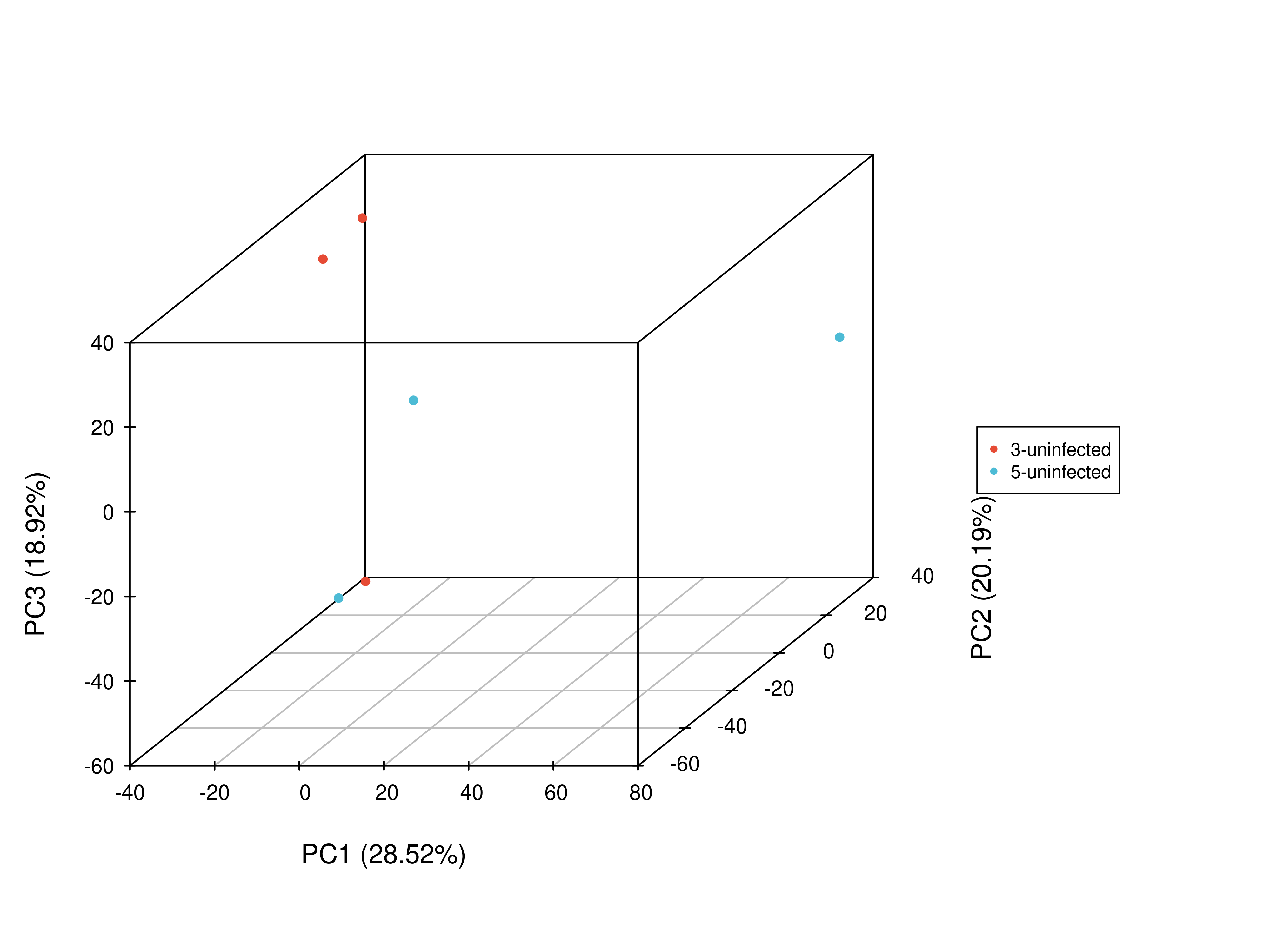

Supplement: Supplementary file 1 [file vaccines-12-00991-s001.zip › Supplementary File S3/proteome/3.Difference/compare/3-uninfected_vs_5-uninfected/3-uninfected_vs_5-uninfected_3d_pca.png]

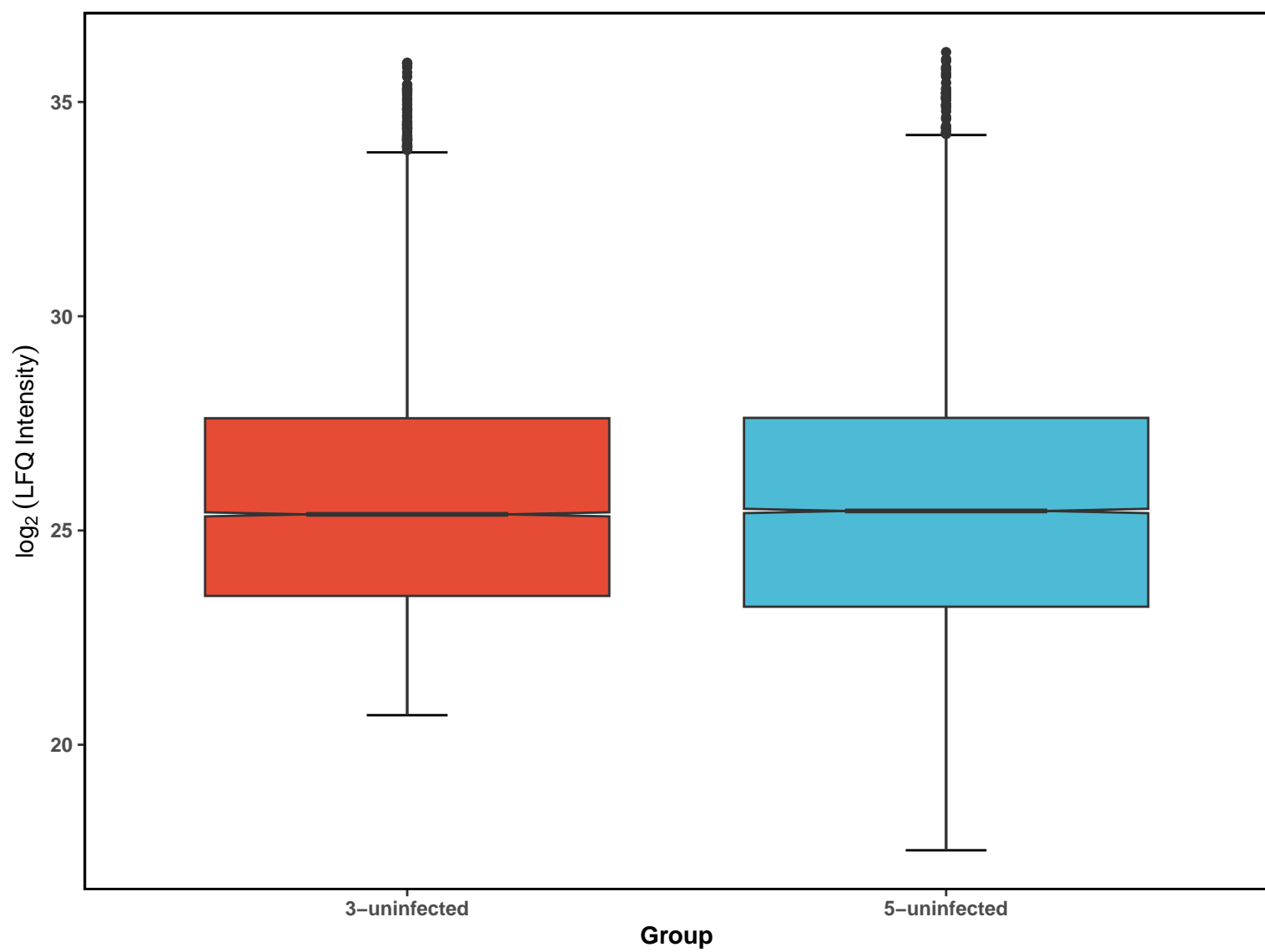

Supplement: Supplementary file 1 [file vaccines-12-00991-s001.zip › Supplementary File S3/proteome/3.Difference/compare/3-uninfected_vs_5-uninfected/3-uninfected_vs_5-uninfected_boxplot.pdf]

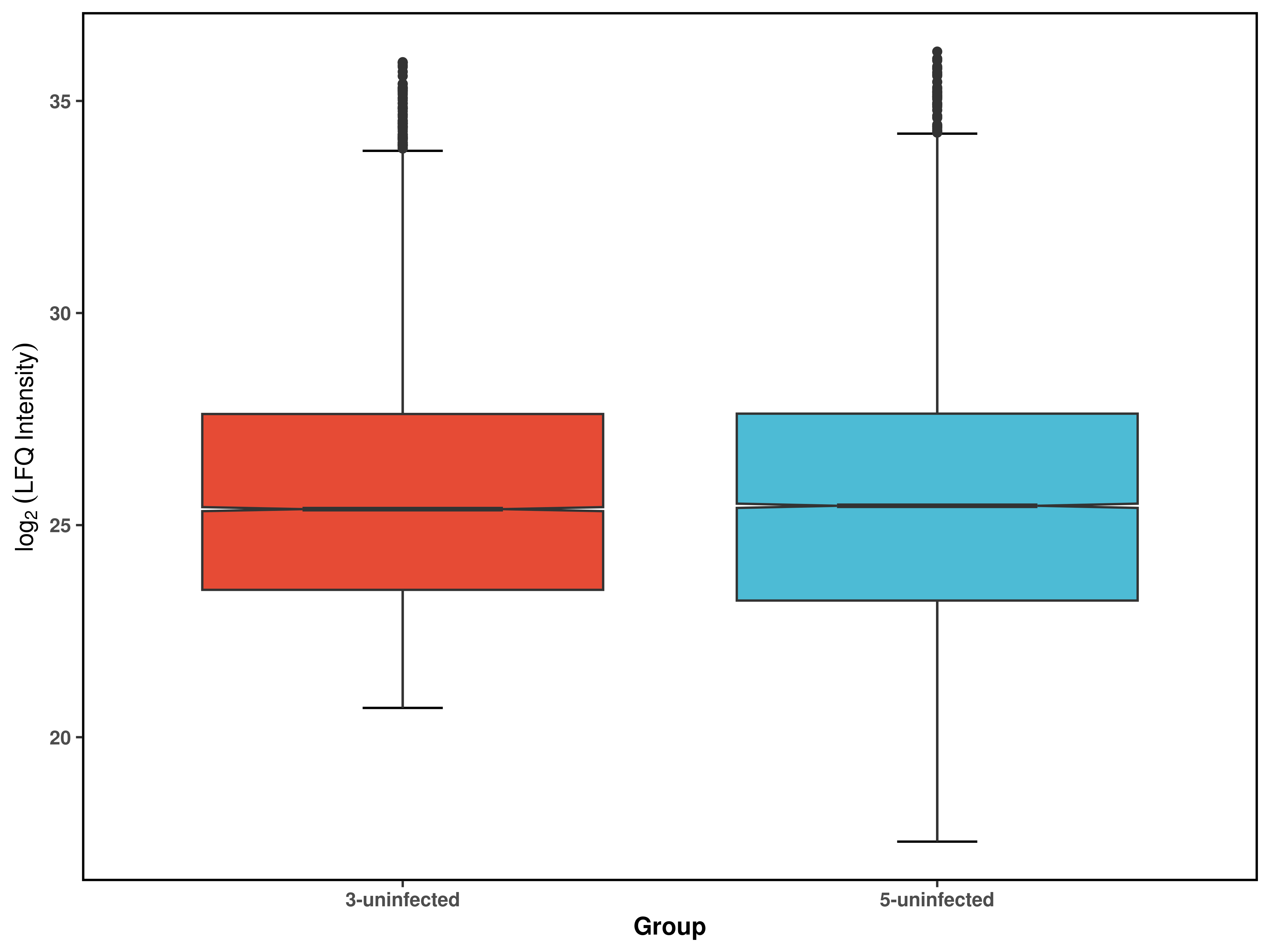

Supplement: Supplementary file 1 [file vaccines-12-00991-s001.zip › Supplementary File S3/proteome/3.Difference/compare/3-uninfected_vs_5-uninfected/3-uninfected_vs_5-uninfected_boxplot.png]

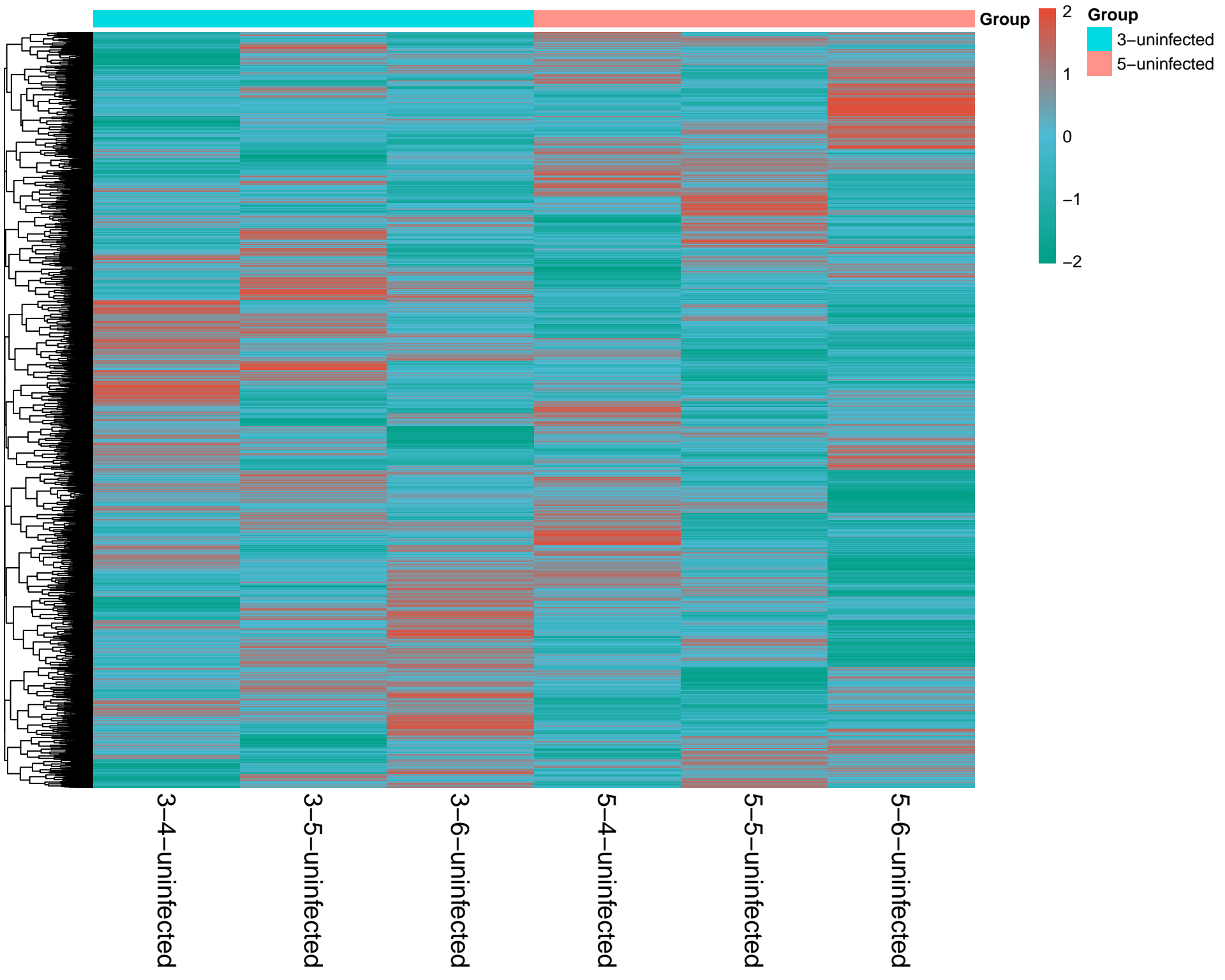

Supplement: Supplementary file 1 [file vaccines-12-00991-s001.zip › Supplementary File S3/proteome/3.Difference/compare/3-uninfected_vs_5-uninfected/3-uninfected_vs_5-uninfected_cluster_heatmap.pdf]

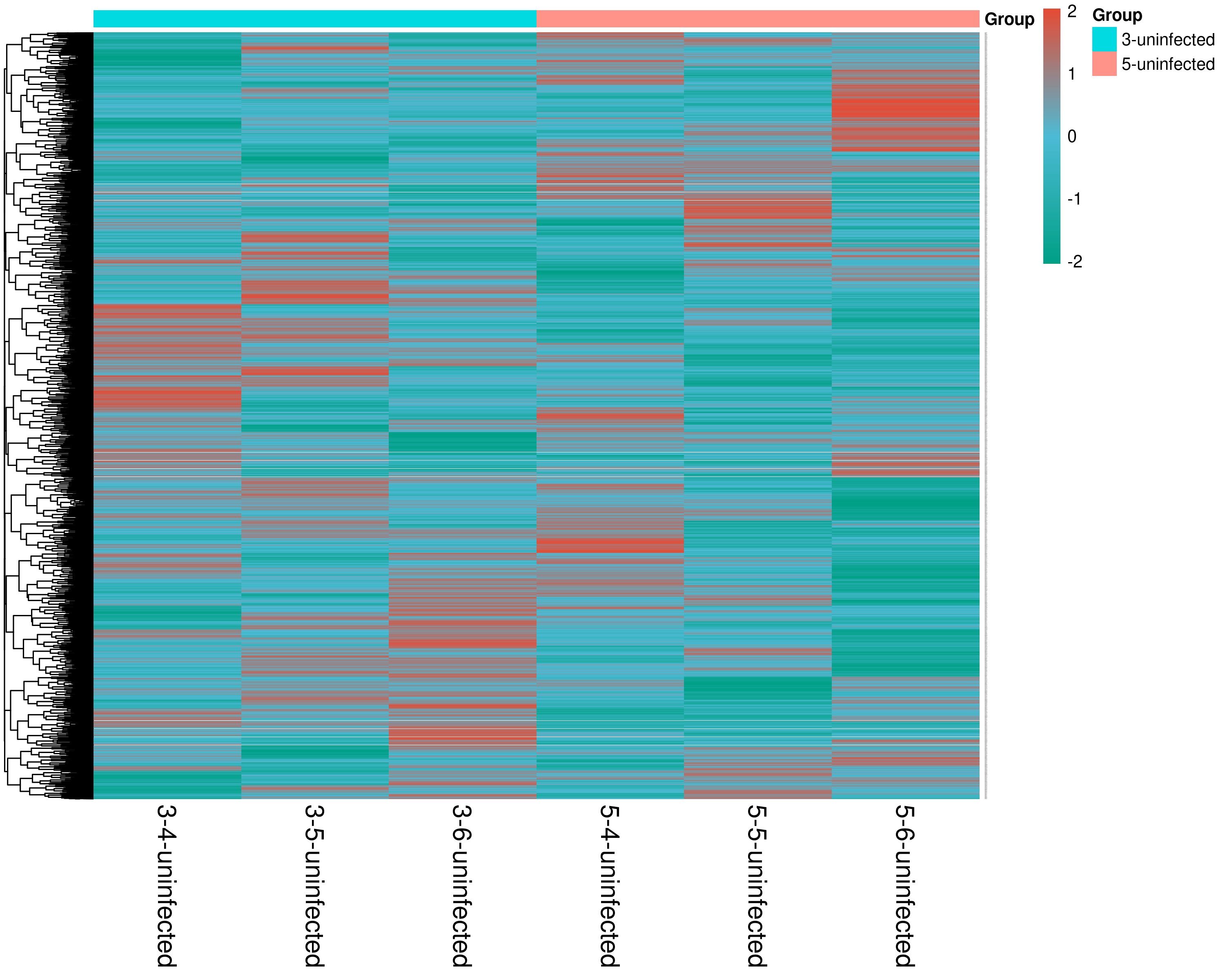

Supplement: Supplementary file 1 [file vaccines-12-00991-s001.zip › Supplementary File S3/proteome/3.Difference/compare/3-uninfected_vs_5-uninfected/3-uninfected_vs_5-uninfected_cluster_heatmap.png]

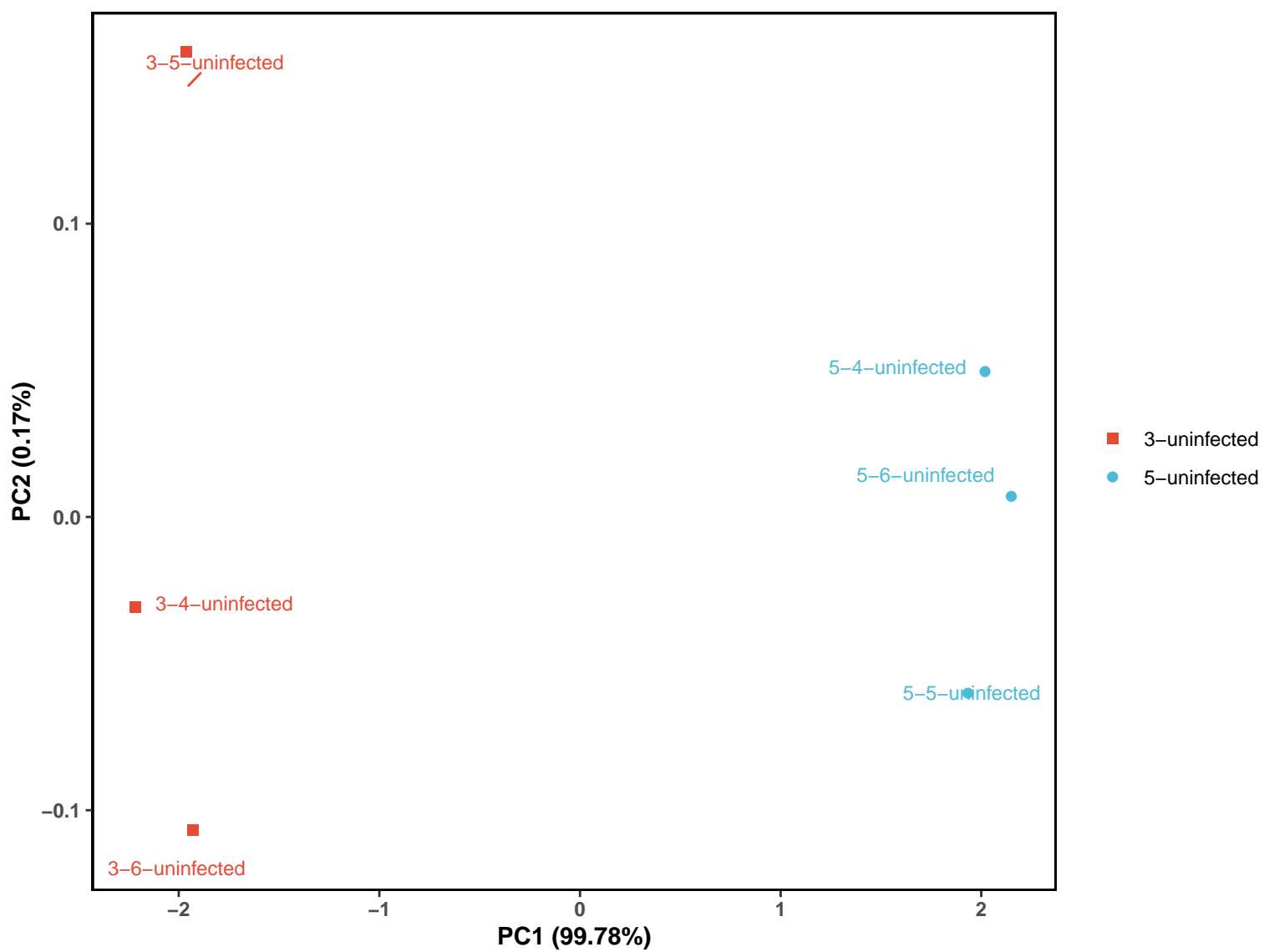

Supplement: Supplementary file 1 [file vaccines-12-00991-s001.zip › Supplementary File S3/proteome/3.Difference/compare/3-uninfected_vs_5-uninfected/3-uninfected_vs_5-uninfected_diff_2d_pca.pdf]

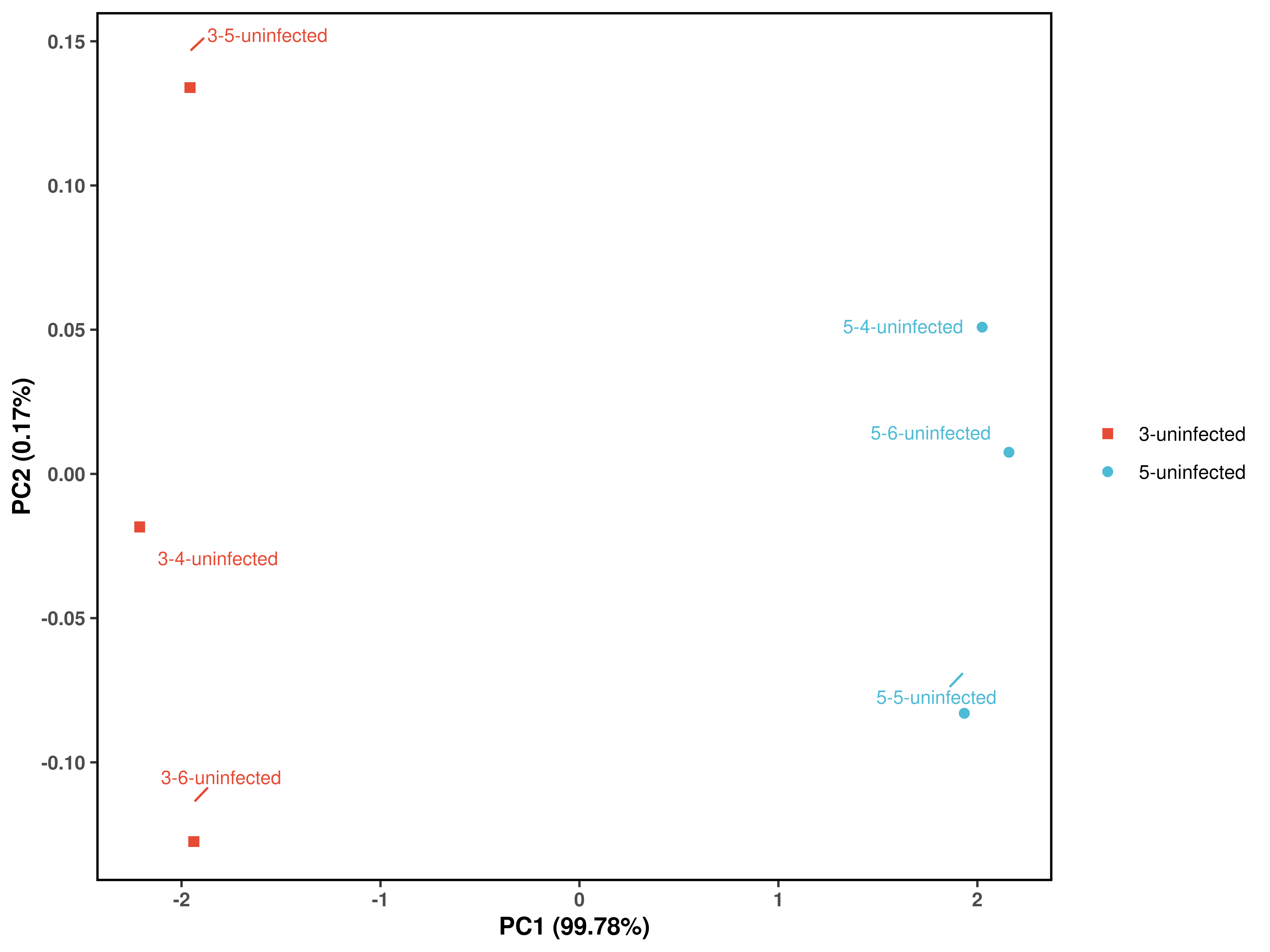

Supplement: Supplementary file 1 [file vaccines-12-00991-s001.zip › Supplementary File S3/proteome/3.Difference/compare/3-uninfected_vs_5-uninfected/3-uninfected_vs_5-uninfected_diff_2d_pca.png]

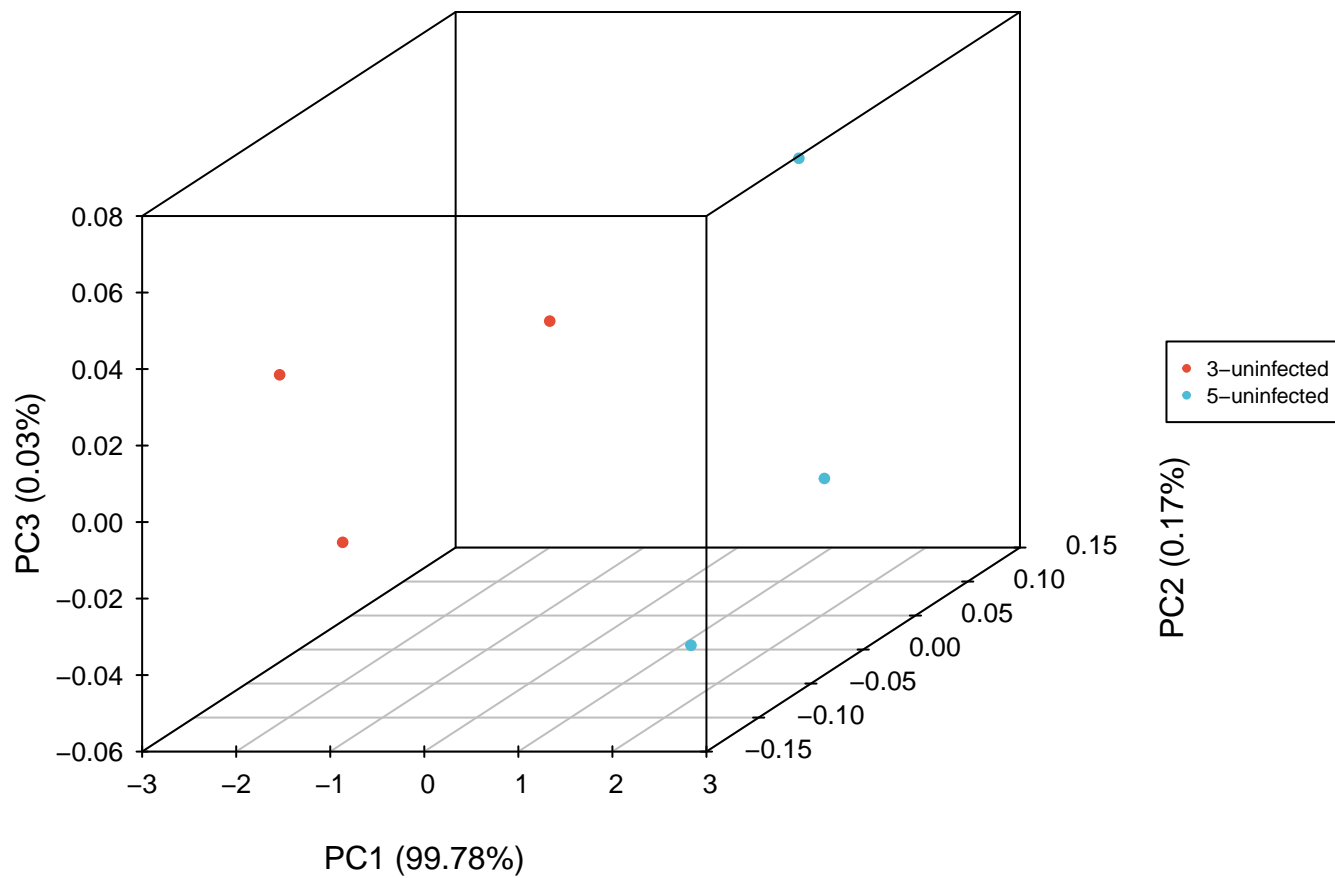

Supplement: Supplementary file 1 [file vaccines-12-00991-s001.zip › Supplementary File S3/proteome/3.Difference/compare/3-uninfected_vs_5-uninfected/3-uninfected_vs_5-uninfected_diff_3d_pca.pdf]

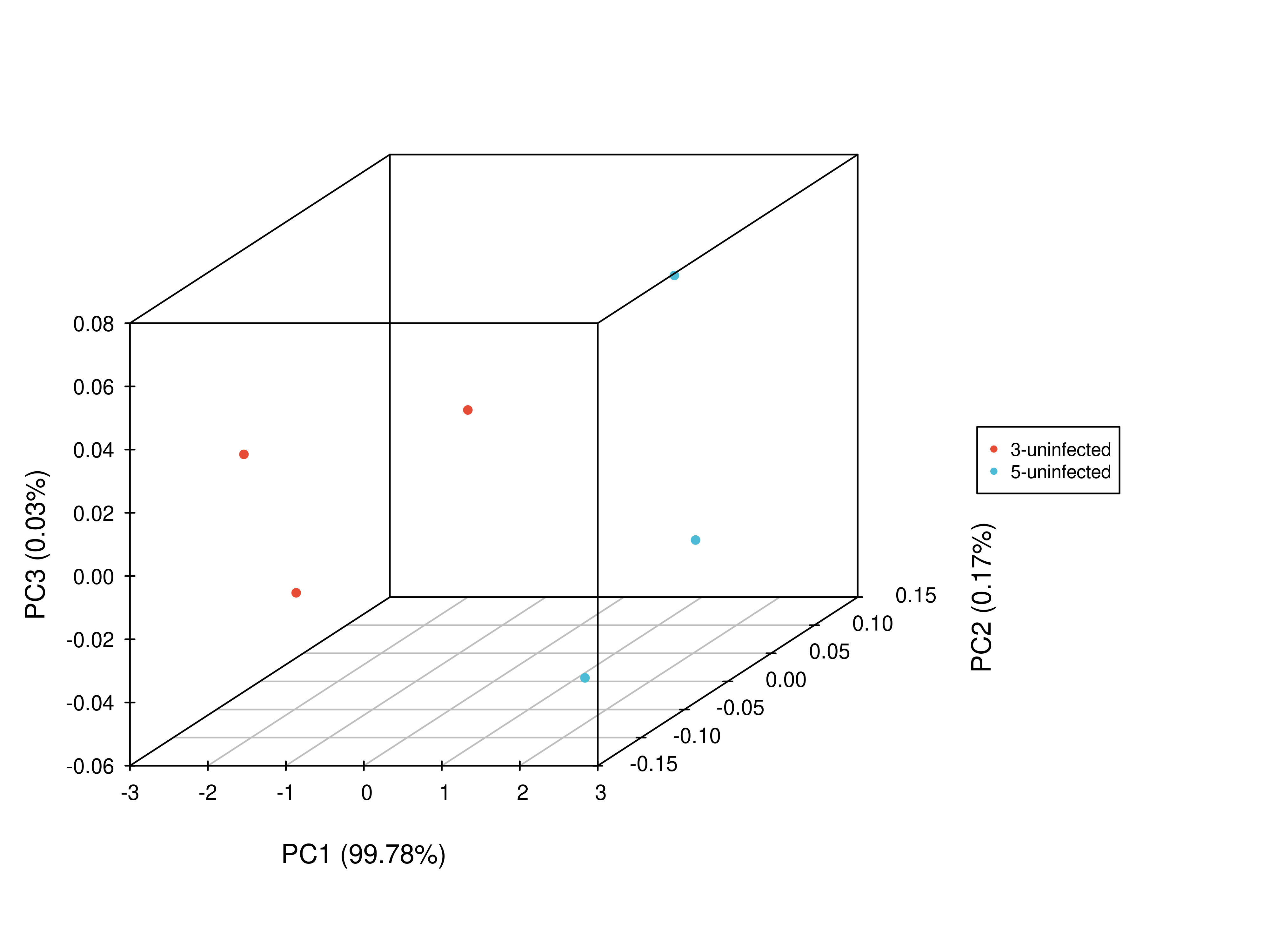

Supplement: Supplementary file 1 [file vaccines-12-00991-s001.zip › Supplementary File S3/proteome/3.Difference/compare/3-uninfected_vs_5-uninfected/3-uninfected_vs_5-uninfected_diff_3d_pca.png]

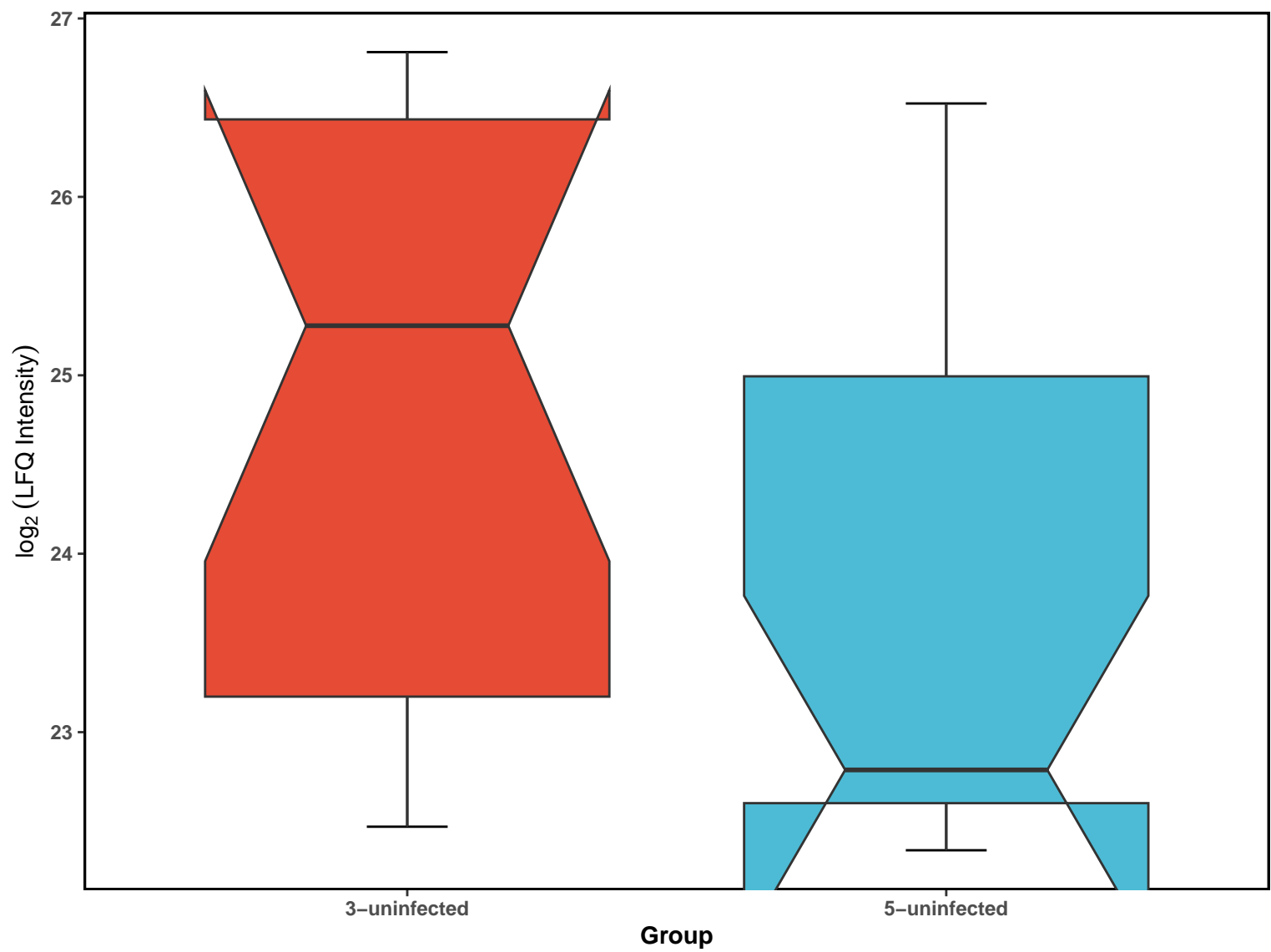

Supplement: Supplementary file 1 [file vaccines-12-00991-s001.zip › Supplementary File S3/proteome/3.Difference/compare/3-uninfected_vs_5-uninfected/3-uninfected_vs_5-uninfected_diff_boxplot.pdf]

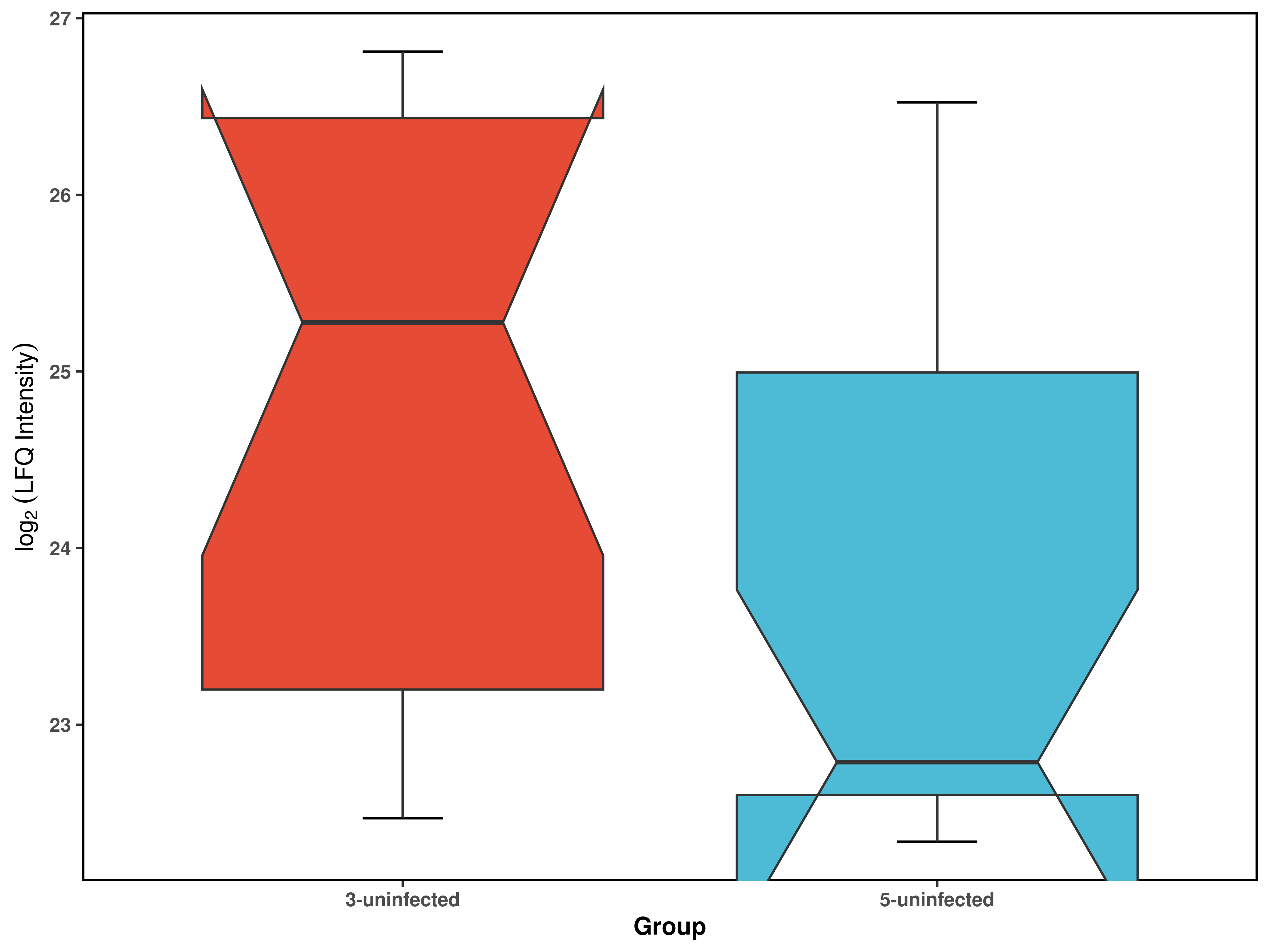

Supplement: Supplementary file 1 [file vaccines-12-00991-s001.zip › Supplementary File S3/proteome/3.Difference/compare/3-uninfected_vs_5-uninfected/3-uninfected_vs_5-uninfected_diff_boxplot.png]

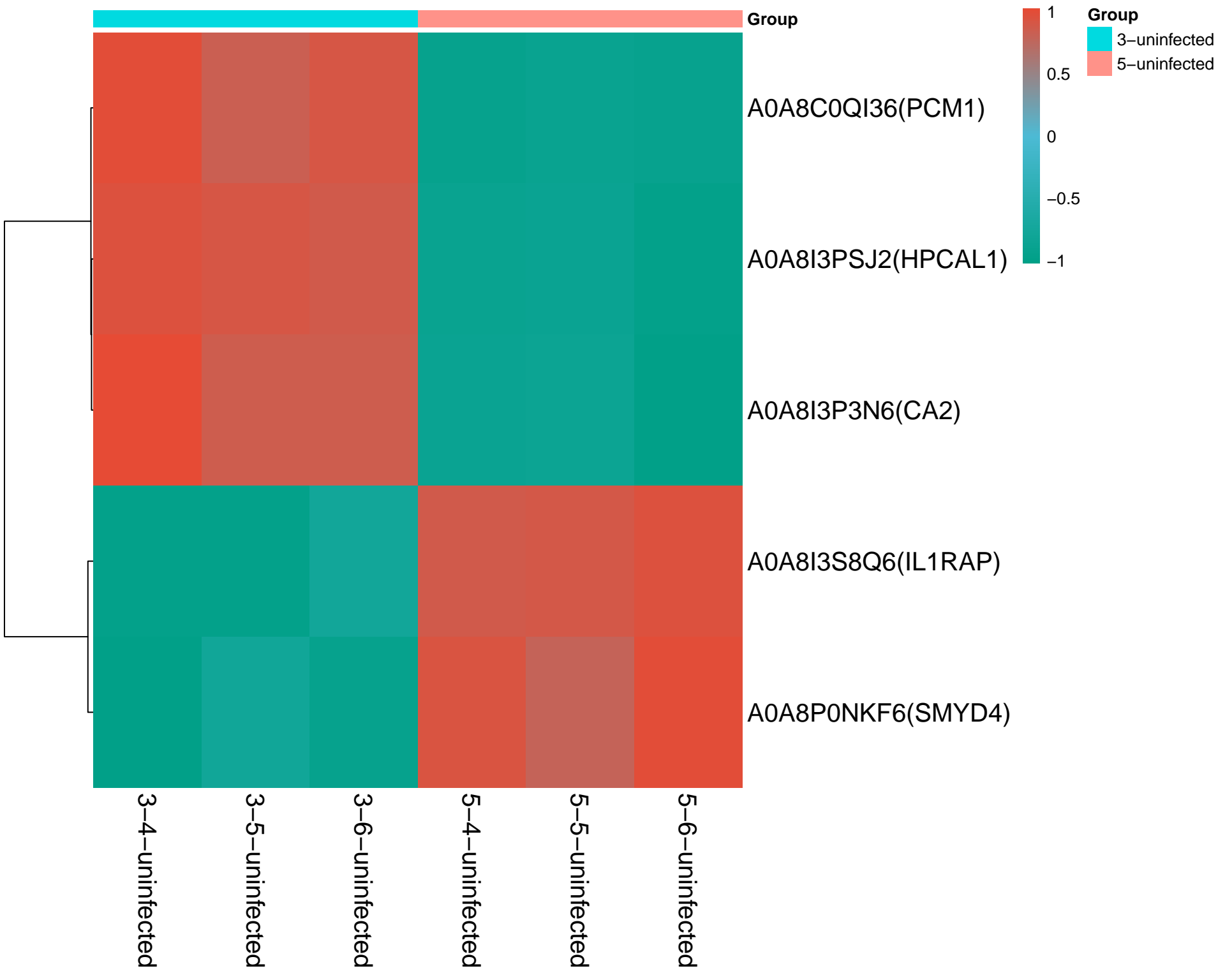

Supplement: Supplementary file 1 [file vaccines-12-00991-s001.zip › Supplementary File S3/proteome/3.Difference/compare/3-uninfected_vs_5-uninfected/3-uninfected_vs_5-uninfected_diff_cluster_heatmap.pdf]

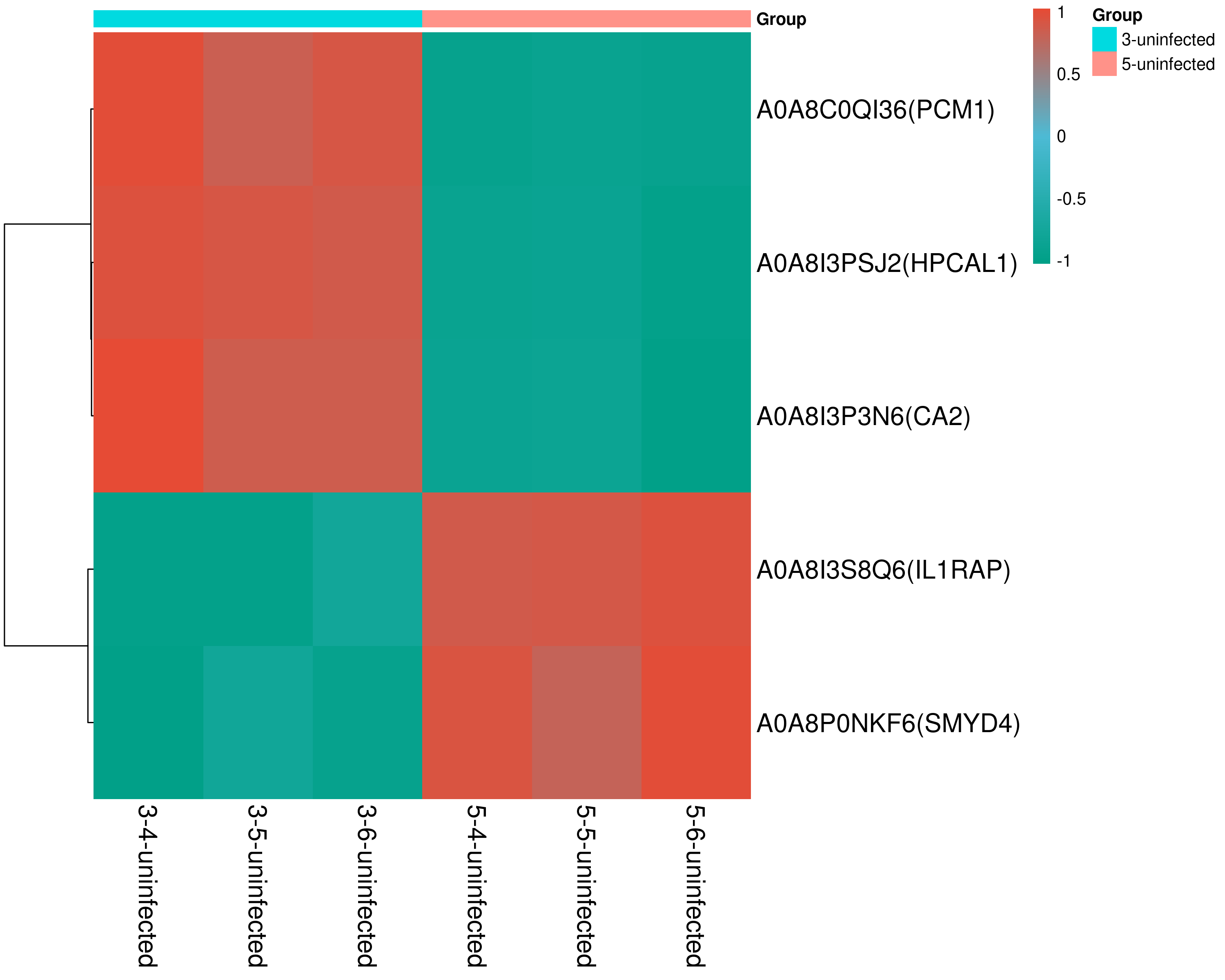

Supplement: Supplementary file 1 [file vaccines-12-00991-s001.zip › Supplementary File S3/proteome/3.Difference/compare/3-uninfected_vs_5-uninfected/3-uninfected_vs_5-uninfected_diff_cluster_heatmap.png]

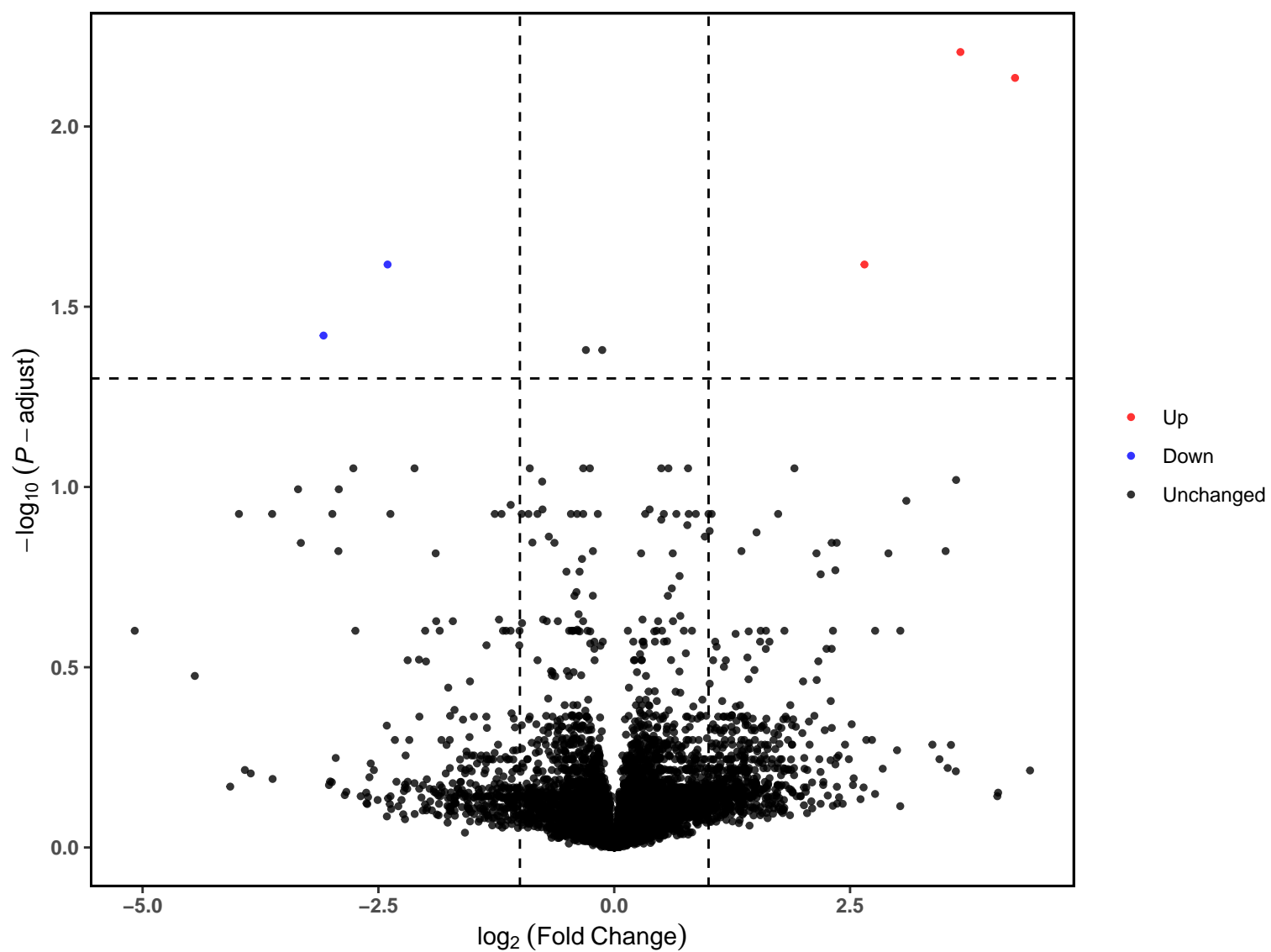

Supplement: Supplementary file 1 [file vaccines-12-00991-s001.zip › Supplementary File S3/proteome/3.Difference/compare/3-uninfected_vs_5-uninfected/3-uninfected_vs_5-uninfected_volcano.pdf]

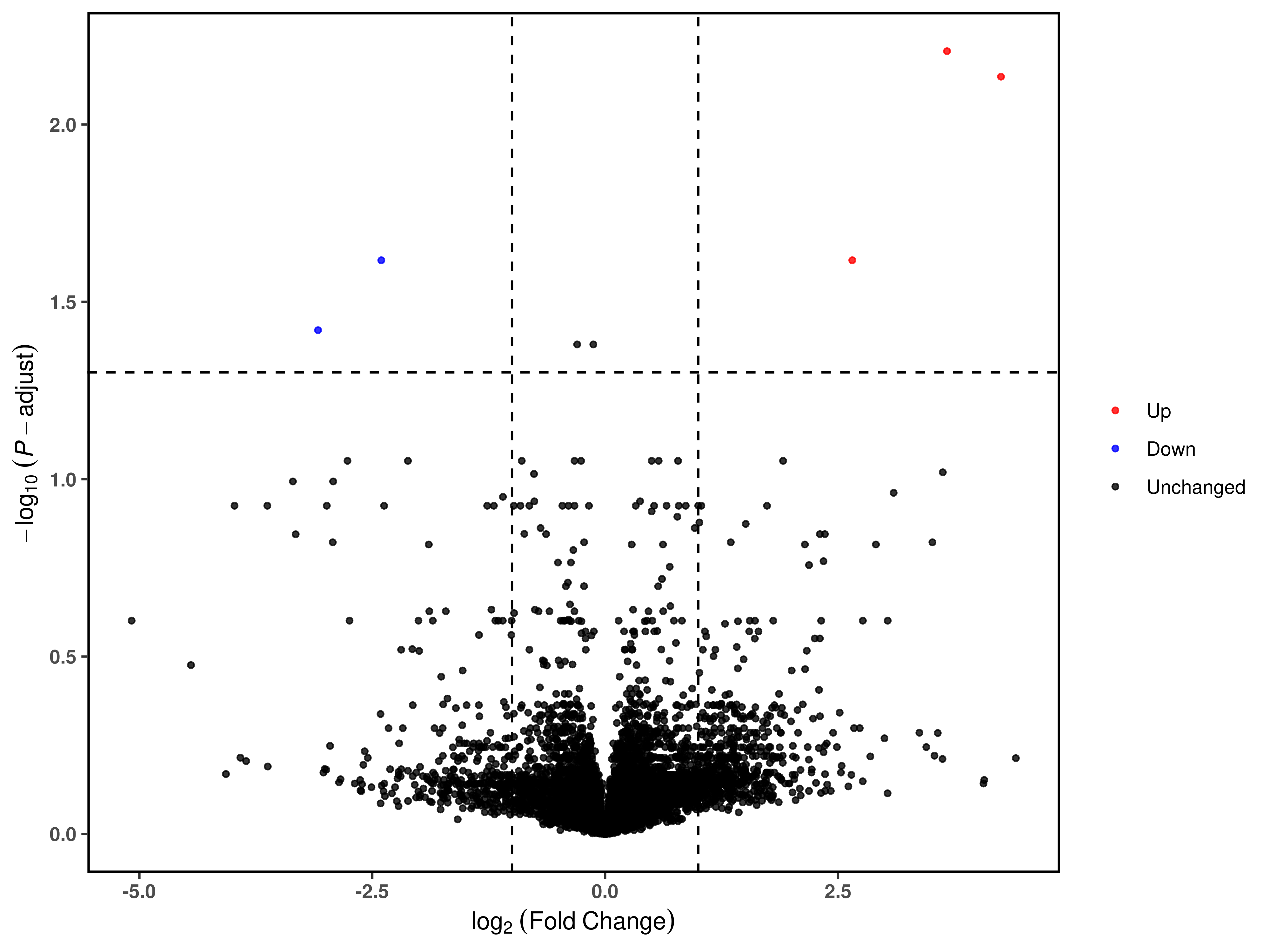

Supplement: Supplementary file 1 [file vaccines-12-00991-s001.zip › Supplementary File S3/proteome/3.Difference/compare/3-uninfected_vs_5-uninfected/3-uninfected_vs_5-uninfected_volcano.png]

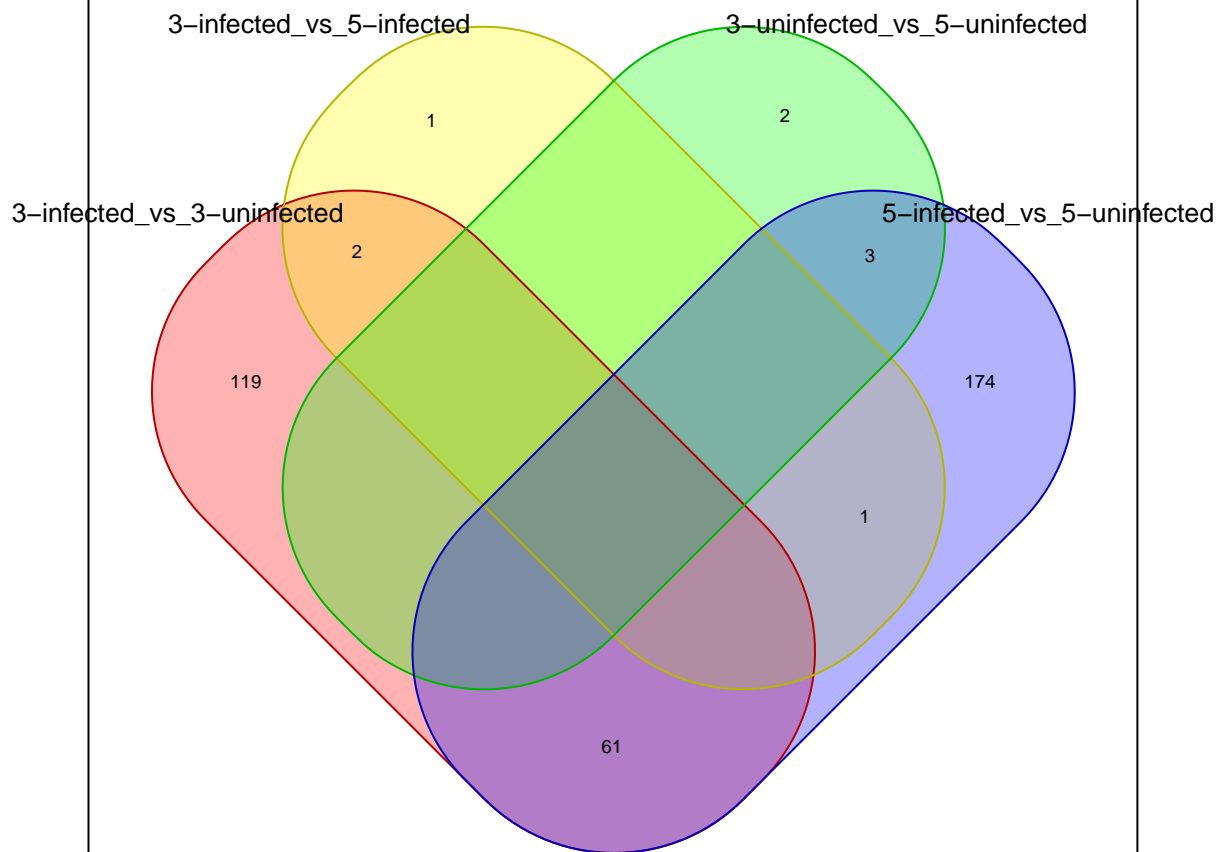

Supplement: Supplementary file 1 [file vaccines-12-00991-s001.zip › Supplementary File S3/proteome/3.Difference/stats/ly_diff_venn.pdf]

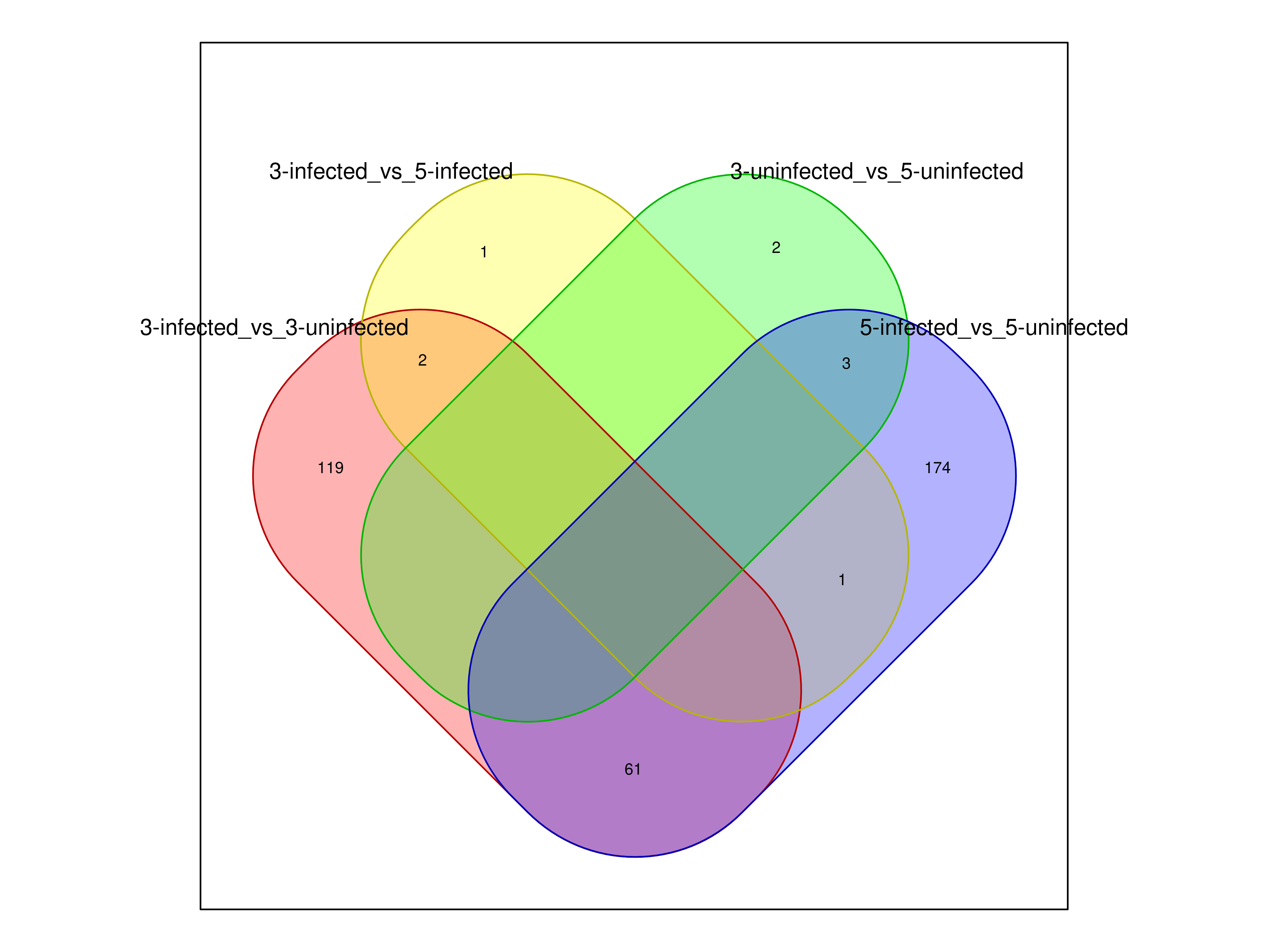

Supplement: Supplementary file 1 [file vaccines-12-00991-s001.zip › Supplementary File S3/proteome/3.Difference/stats/ly_diff_venn.png]

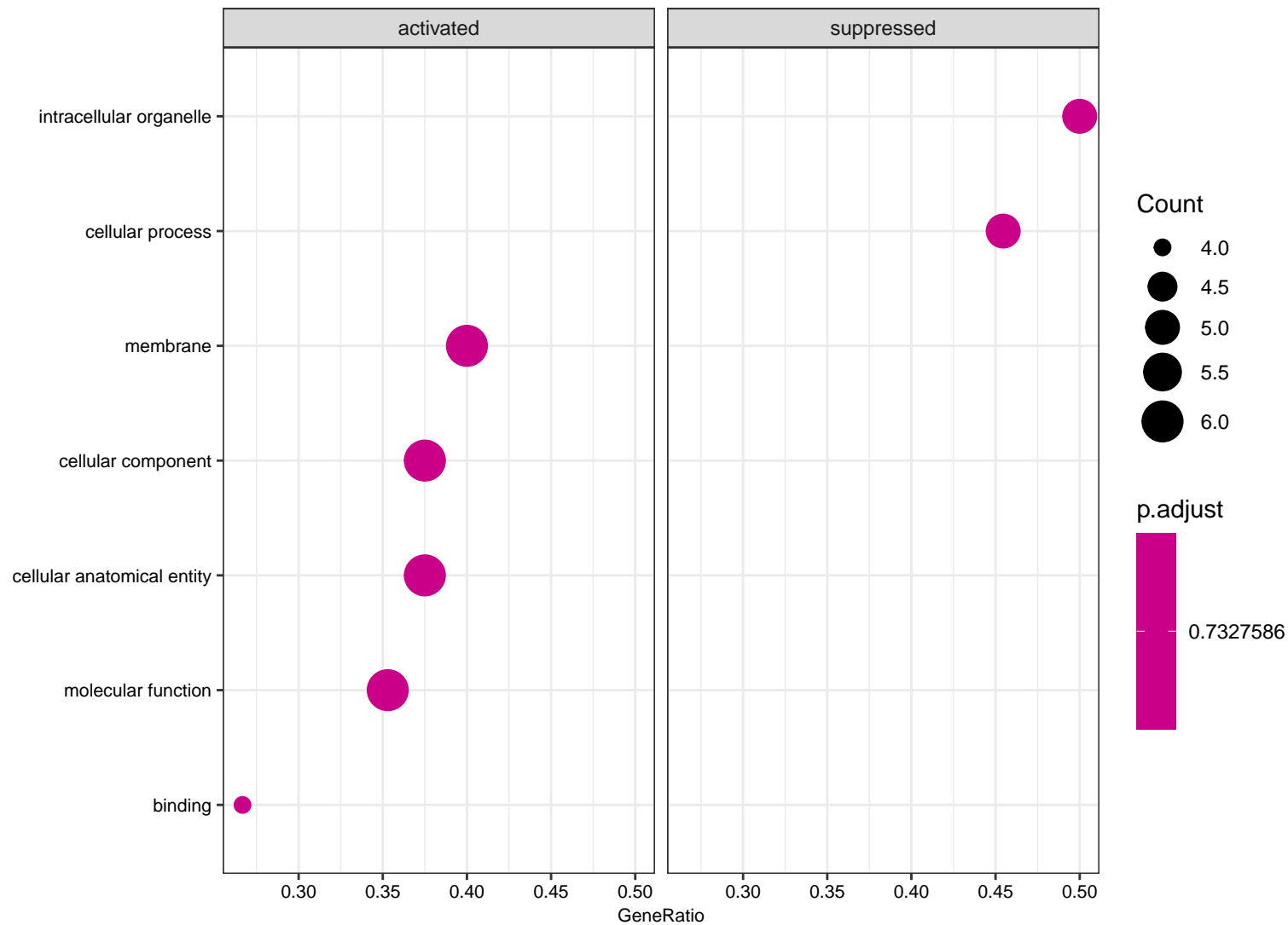

Supplement: Supplementary file 1 [file vaccines-12-00991-s001.zip › Supplementary File S3/proteome/4.Enrichment/gsea/3-infected_vs_3-uninfected/3-infected_vs_3-uninfected_GO_ALL_GSEA_dotplot.pdf]

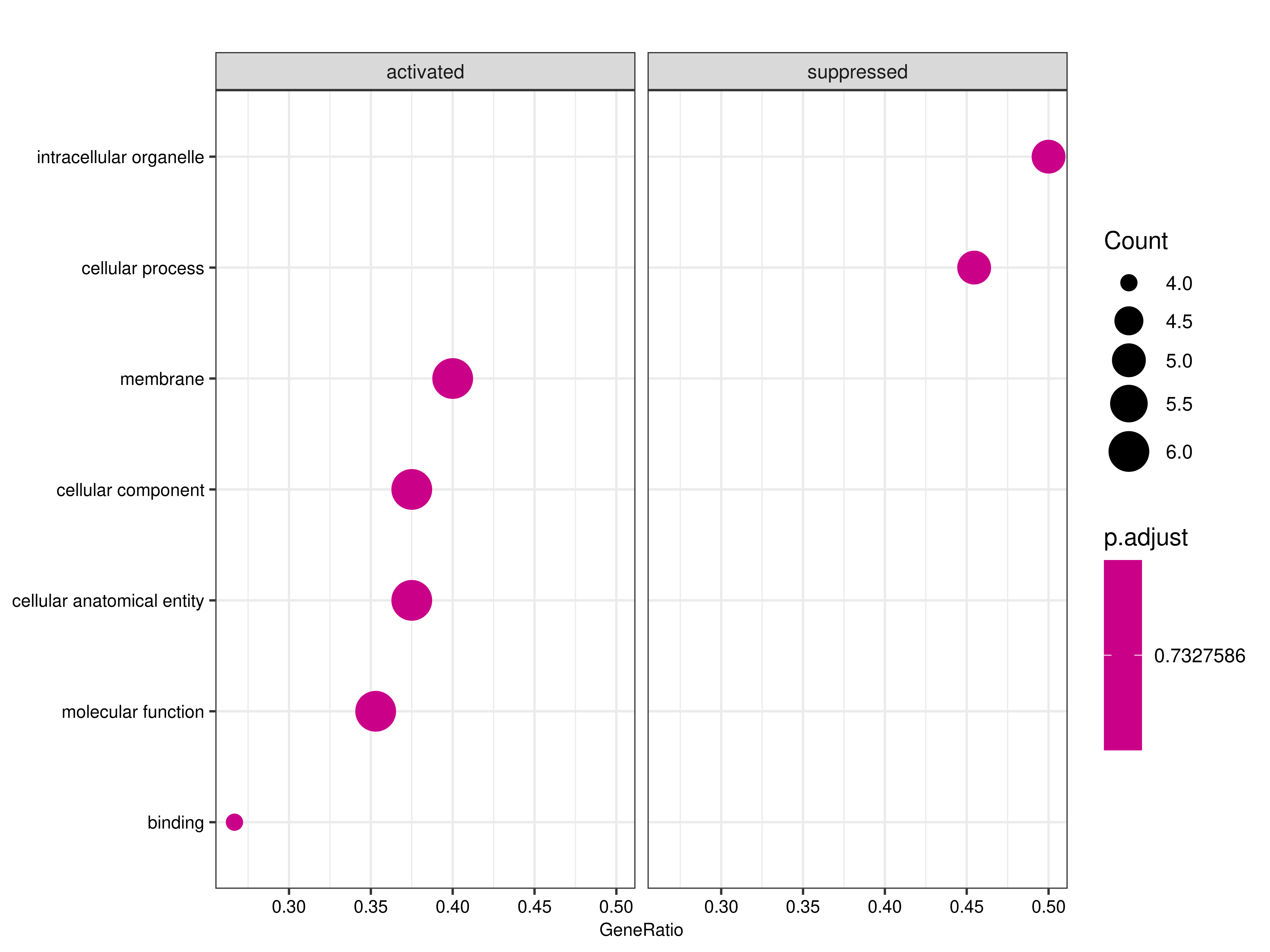

Supplement: Supplementary file 1 [file vaccines-12-00991-s001.zip › Supplementary File S3/proteome/4.Enrichment/gsea/3-infected_vs_3-uninfected/3-infected_vs_3-uninfected_GO_ALL_GSEA_dotplot.png]

# cellular process

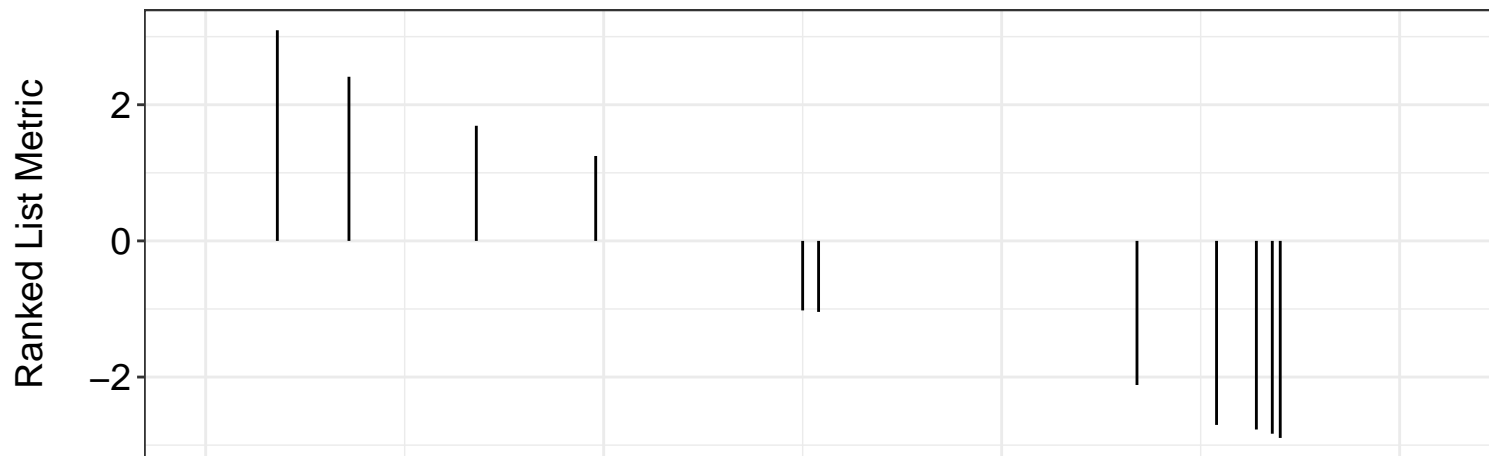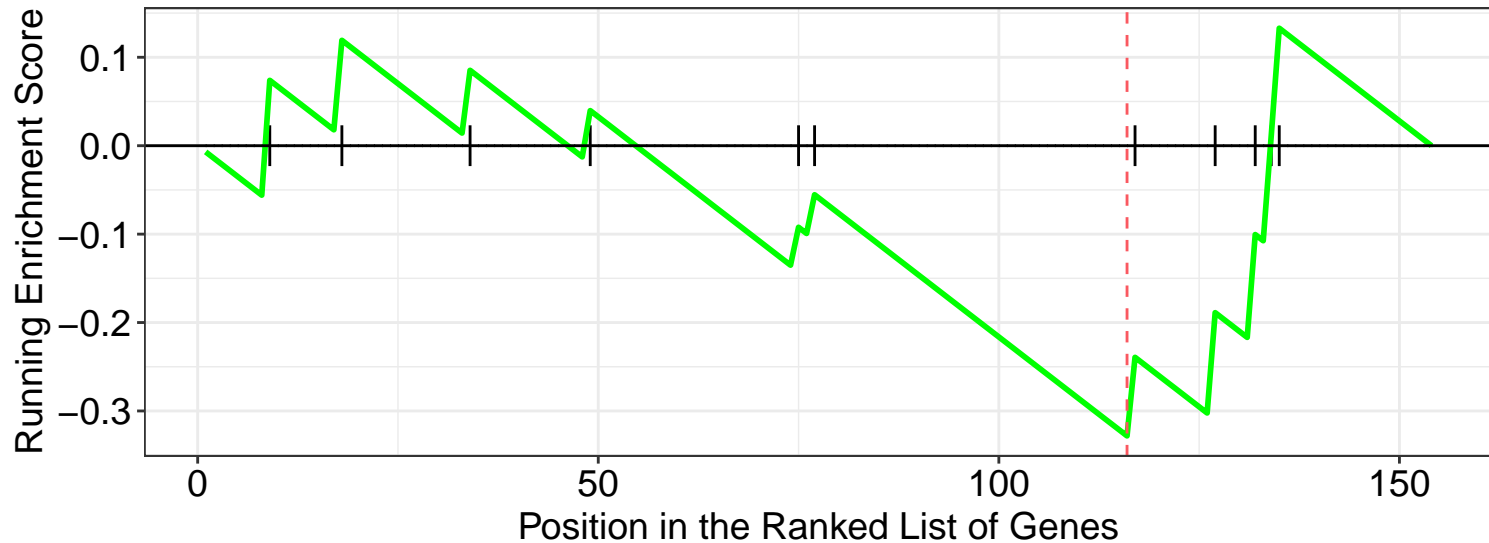

Supplement: Supplementary file 1 [file vaccines-12-00991-s001.zip › Supplementary File S3/proteome/4.Enrichment/gsea/3-infected_vs_3-uninfected/3-infected_vs_3-uninfected_GO_ALL_GSEA_gseaplot.pdf]

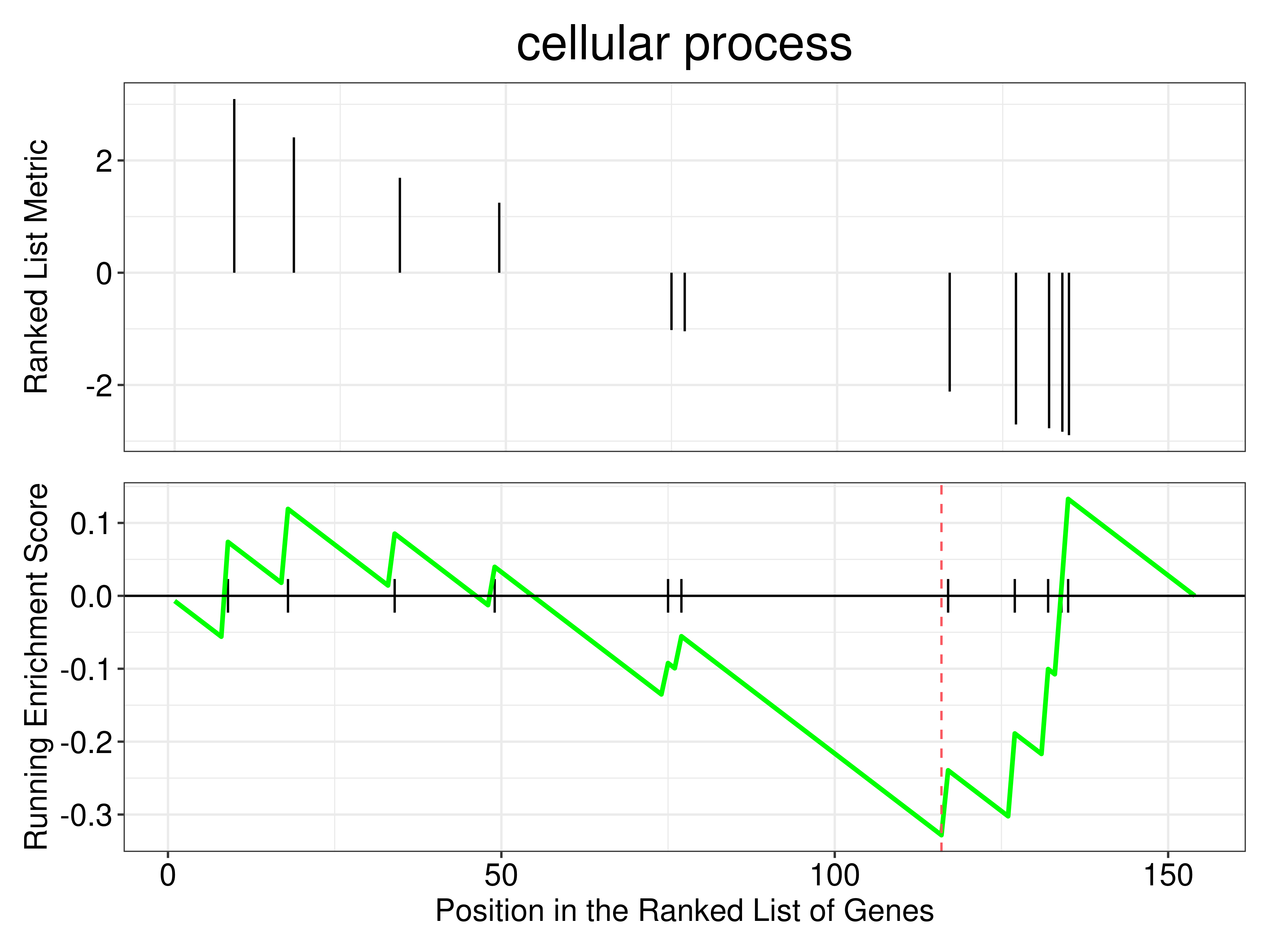

Supplement: Supplementary file 1 [file vaccines-12-00991-s001.zip › Supplementary File S3/proteome/4.Enrichment/gsea/3-infected_vs_3-uninfected/3-infected_vs_3-uninfected_GO_ALL_GSEA_gseaplot.png]

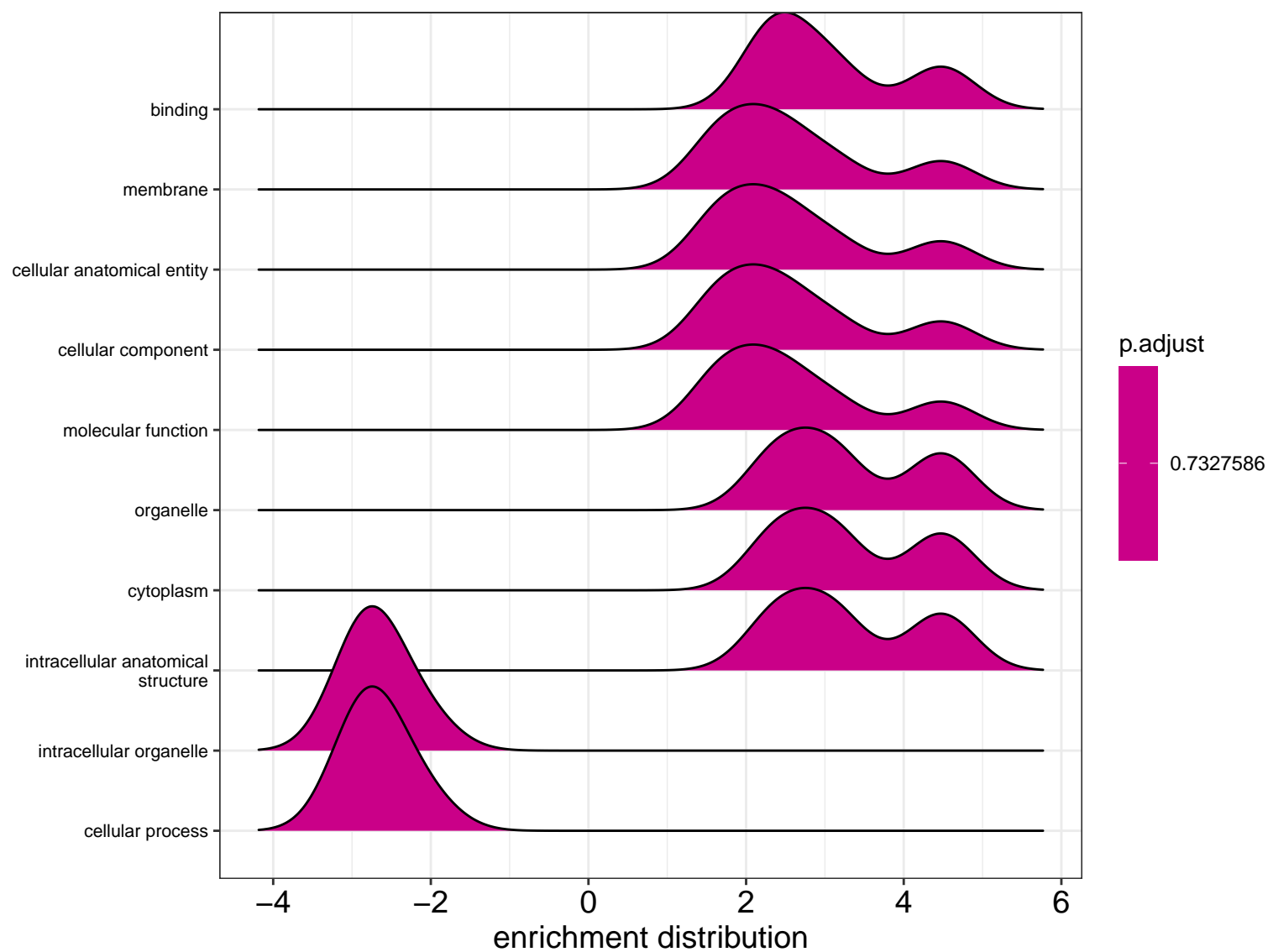

Supplement: Supplementary file 1 [file vaccines-12-00991-s001.zip › Supplementary File S3/proteome/4.Enrichment/gsea/3-infected_vs_3-uninfected/3-infected_vs_3-uninfected_GO_ALL_GSEA_ridgeplot.pdf]

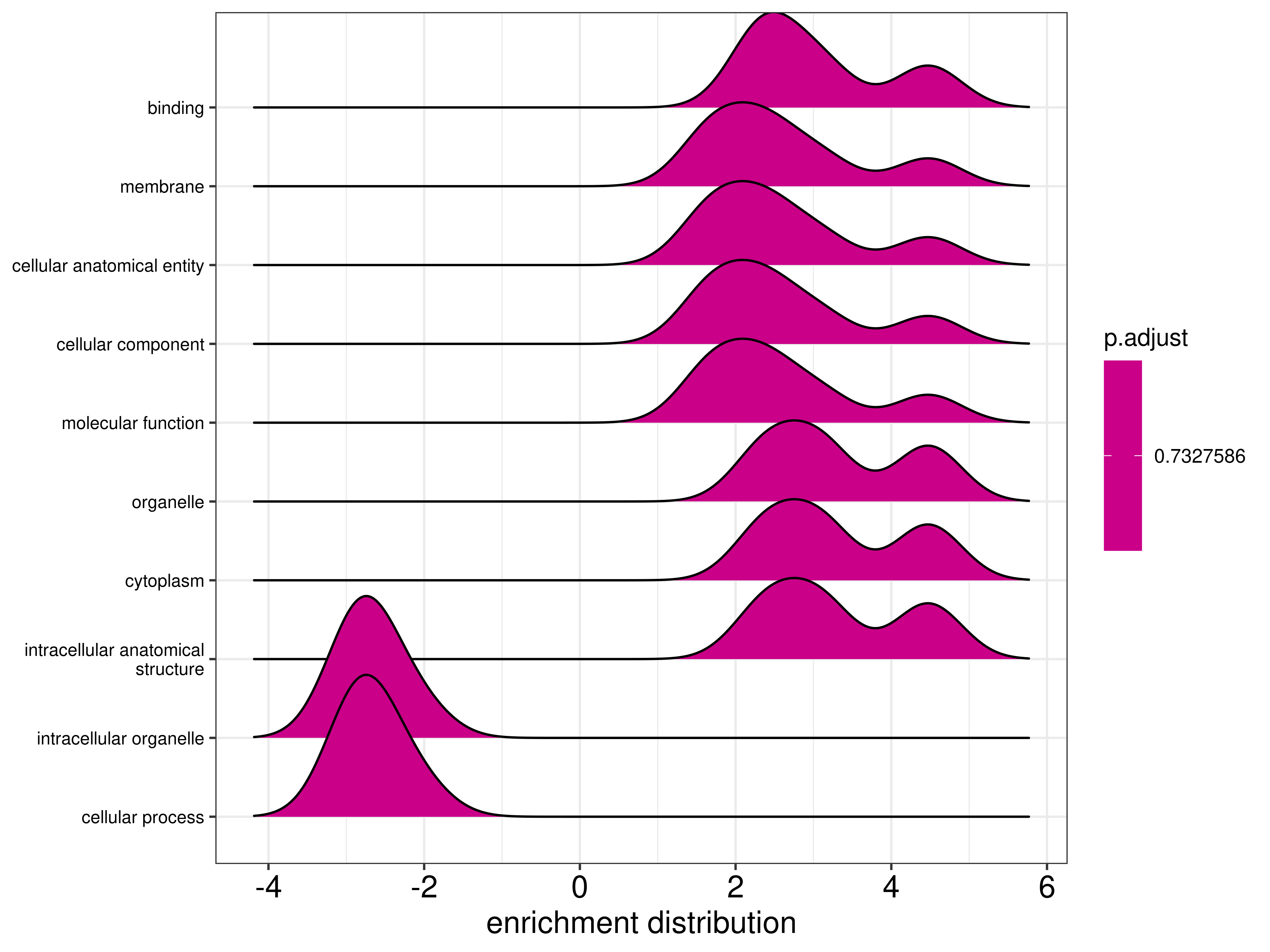

Supplement: Supplementary file 1 [file vaccines-12-00991-s001.zip › Supplementary File S3/proteome/4.Enrichment/gsea/3-infected_vs_3-uninfected/3-infected_vs_3-uninfected_GO_ALL_GSEA_ridgeplot.png]

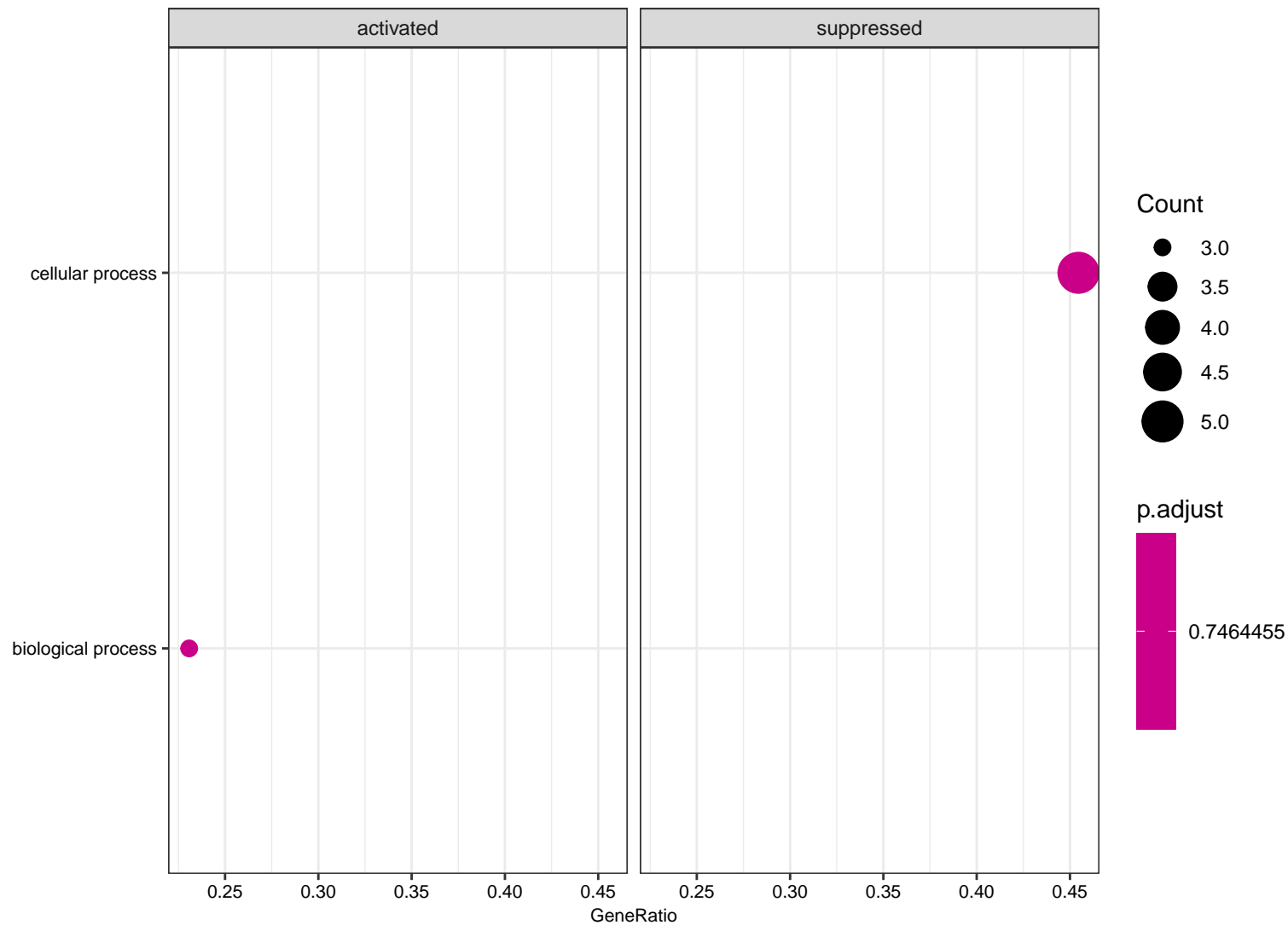

Supplement: Supplementary file 1 [file vaccines-12-00991-s001.zip › Supplementary File S3/proteome/4.Enrichment/gsea/3-infected_vs_3-uninfected/3-infected_vs_3-uninfected_GO_BP_GSEA_dotplot.pdf]

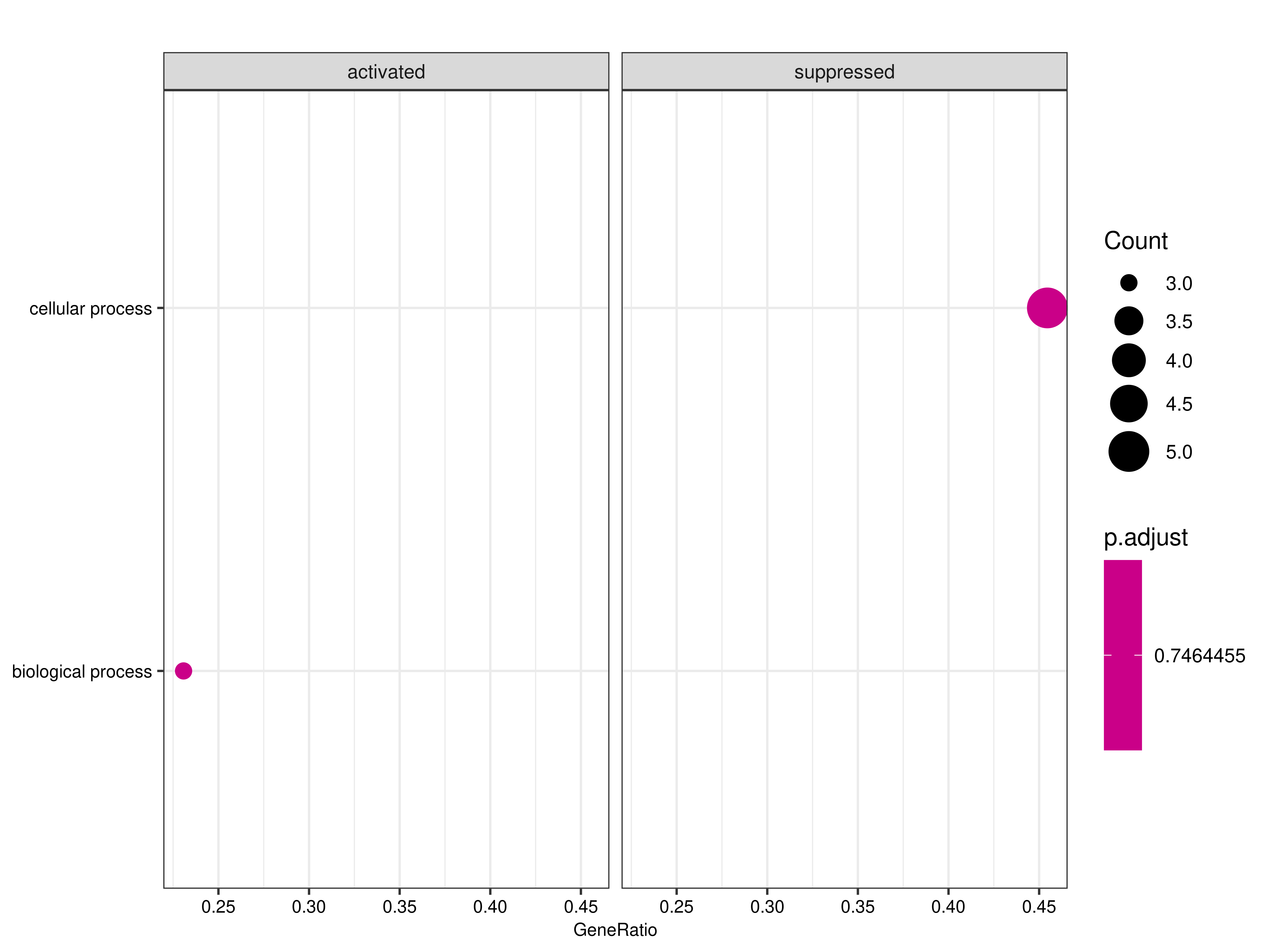

Supplement: Supplementary file 1 [file vaccines-12-00991-s001.zip › Supplementary File S3/proteome/4.Enrichment/gsea/3-infected_vs_3-uninfected/3-infected_vs_3-uninfected_GO_BP_GSEA_dotplot.png]

# cellular process

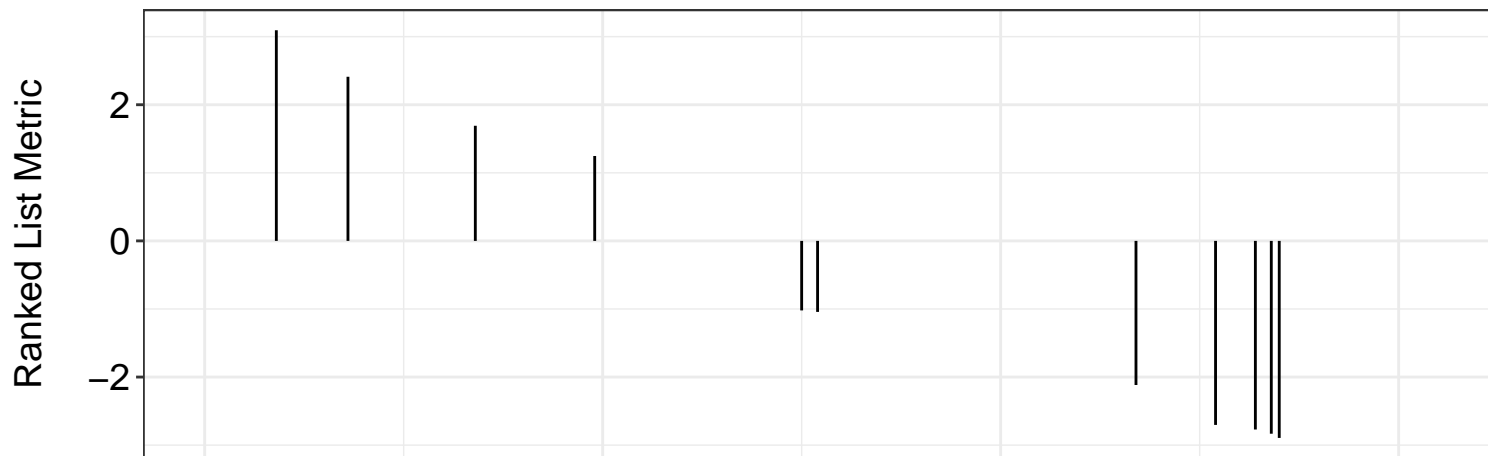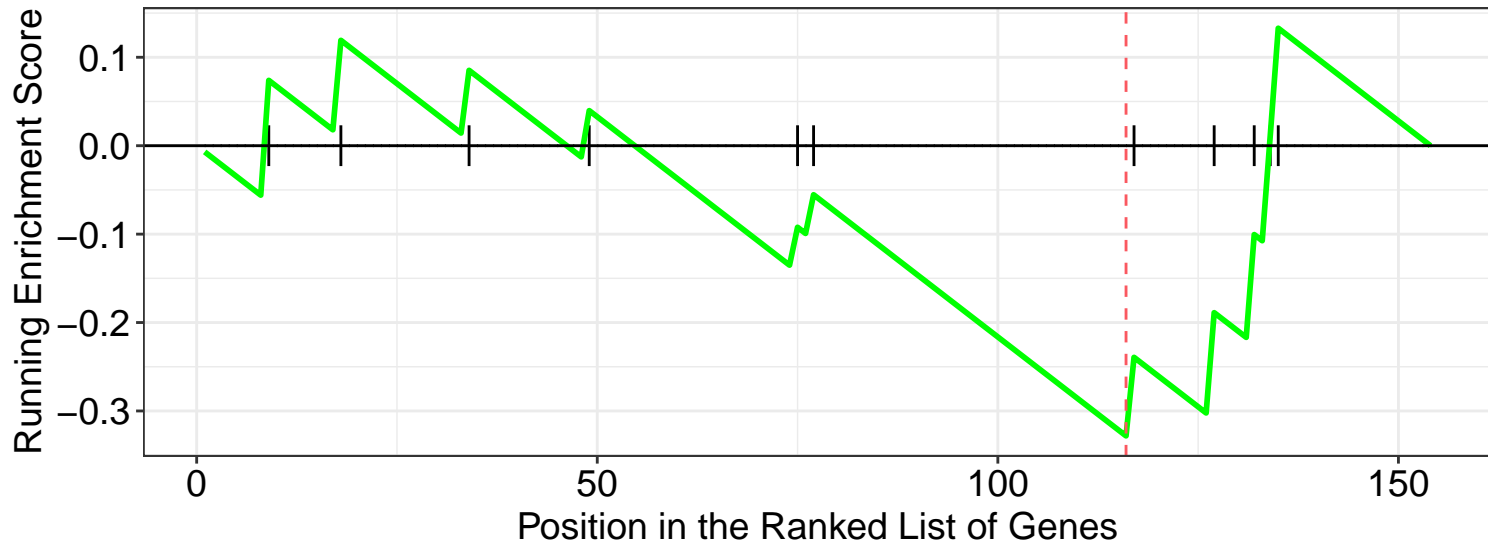

Supplement: Supplementary file 1 [file vaccines-12-00991-s001.zip › Supplementary File S3/proteome/4.Enrichment/gsea/3-infected_vs_3-uninfected/3-infected_vs_3-uninfected_GO_BP_GSEA_gseaplot.pdf]

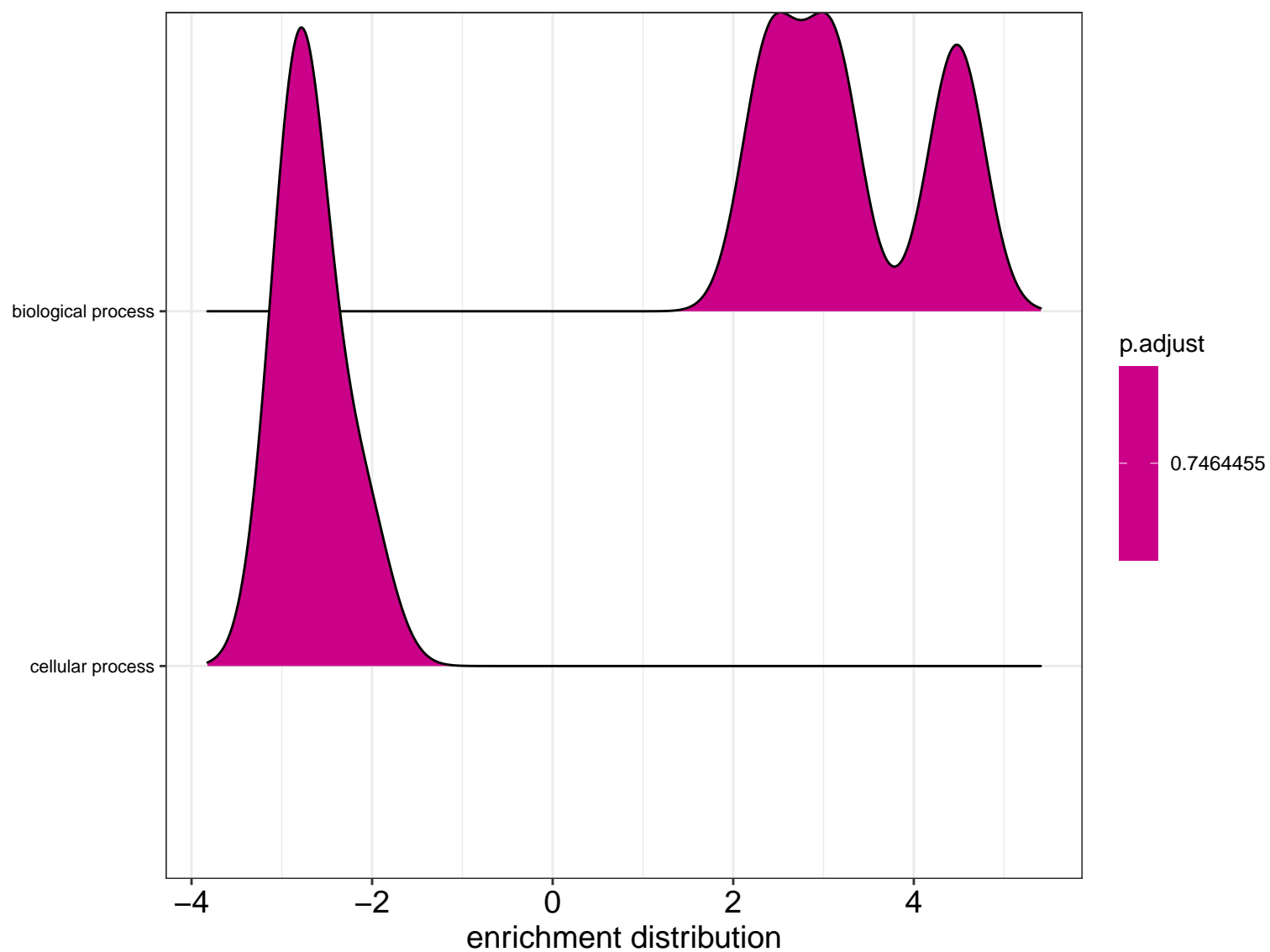

Supplement: Supplementary file 1 [file vaccines-12-00991-s001.zip › Supplementary File S3/proteome/4.Enrichment/gsea/3-infected_vs_3-uninfected/3-infected_vs_3-uninfected_GO_BP_GSEA_ridgeplot.pdf]

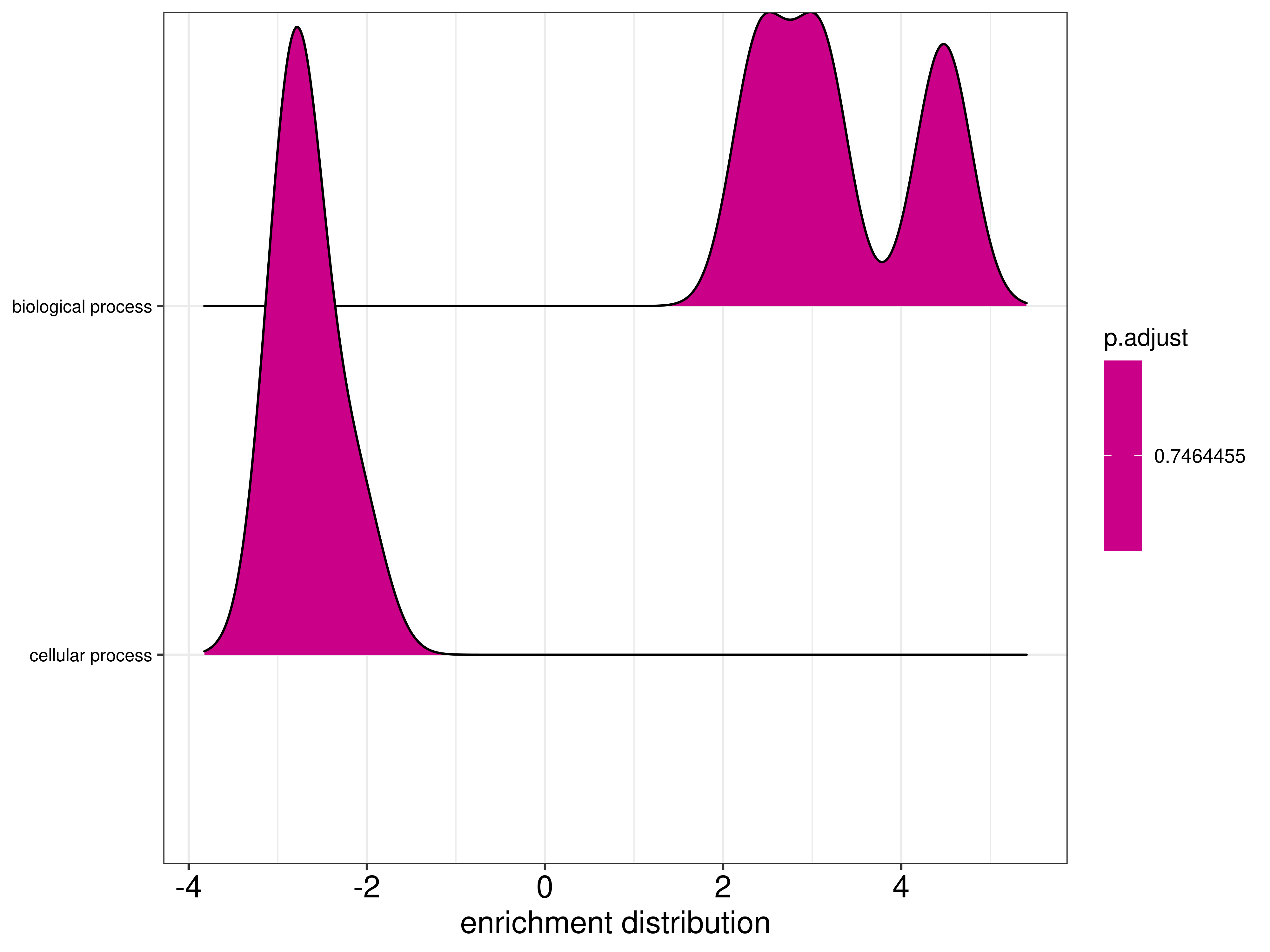

Supplement: Supplementary file 1 [file vaccines-12-00991-s001.zip › Supplementary File S3/proteome/4.Enrichment/gsea/3-infected_vs_3-uninfected/3-infected_vs_3-uninfected_GO_BP_GSEA_ridgeplot.png]

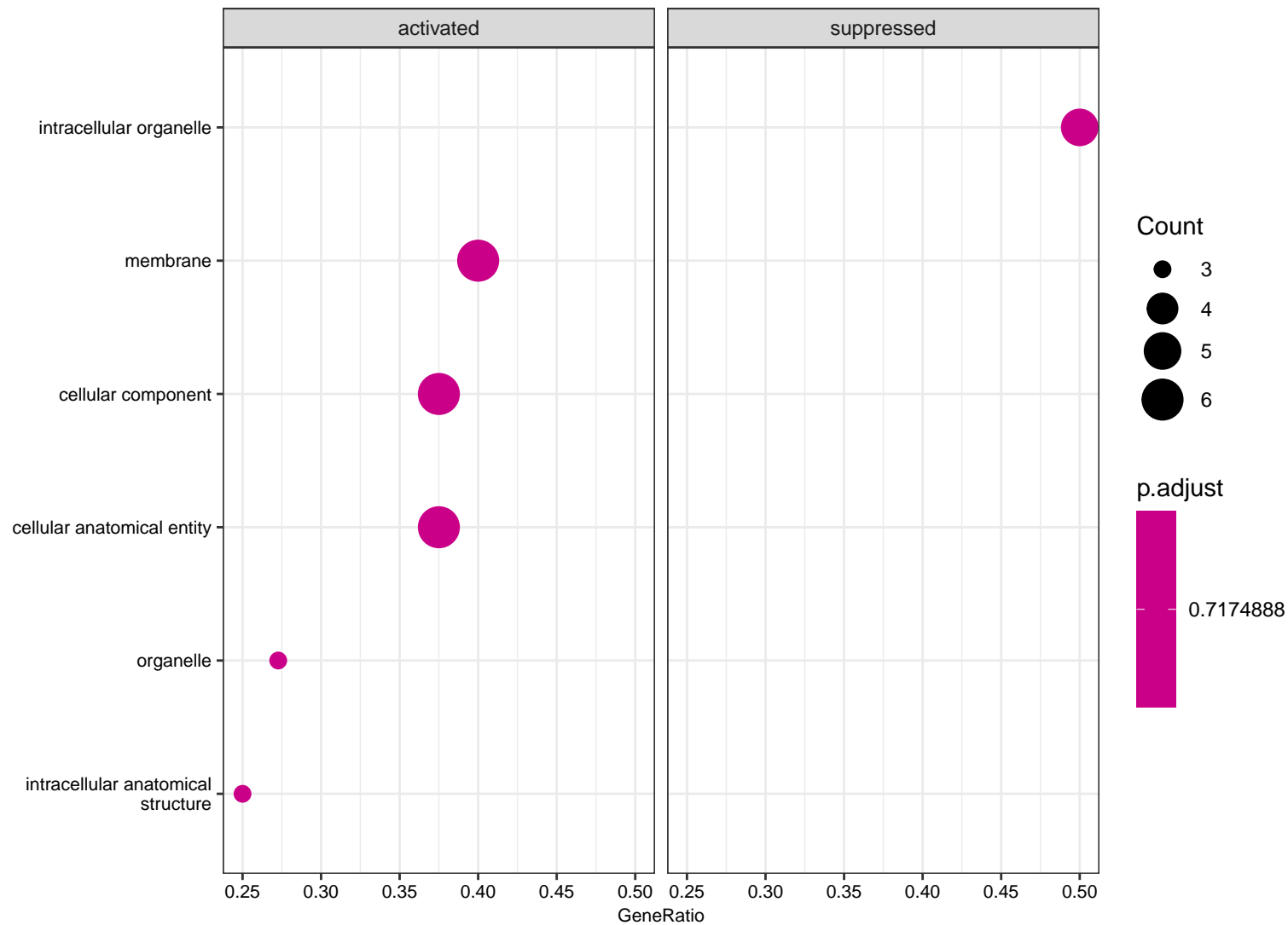

Supplement: Supplementary file 1 [file vaccines-12-00991-s001.zip › Supplementary File S3/proteome/4.Enrichment/gsea/3-infected_vs_3-uninfected/3-infected_vs_3-uninfected_GO_CC_GSEA_dotplot.pdf]

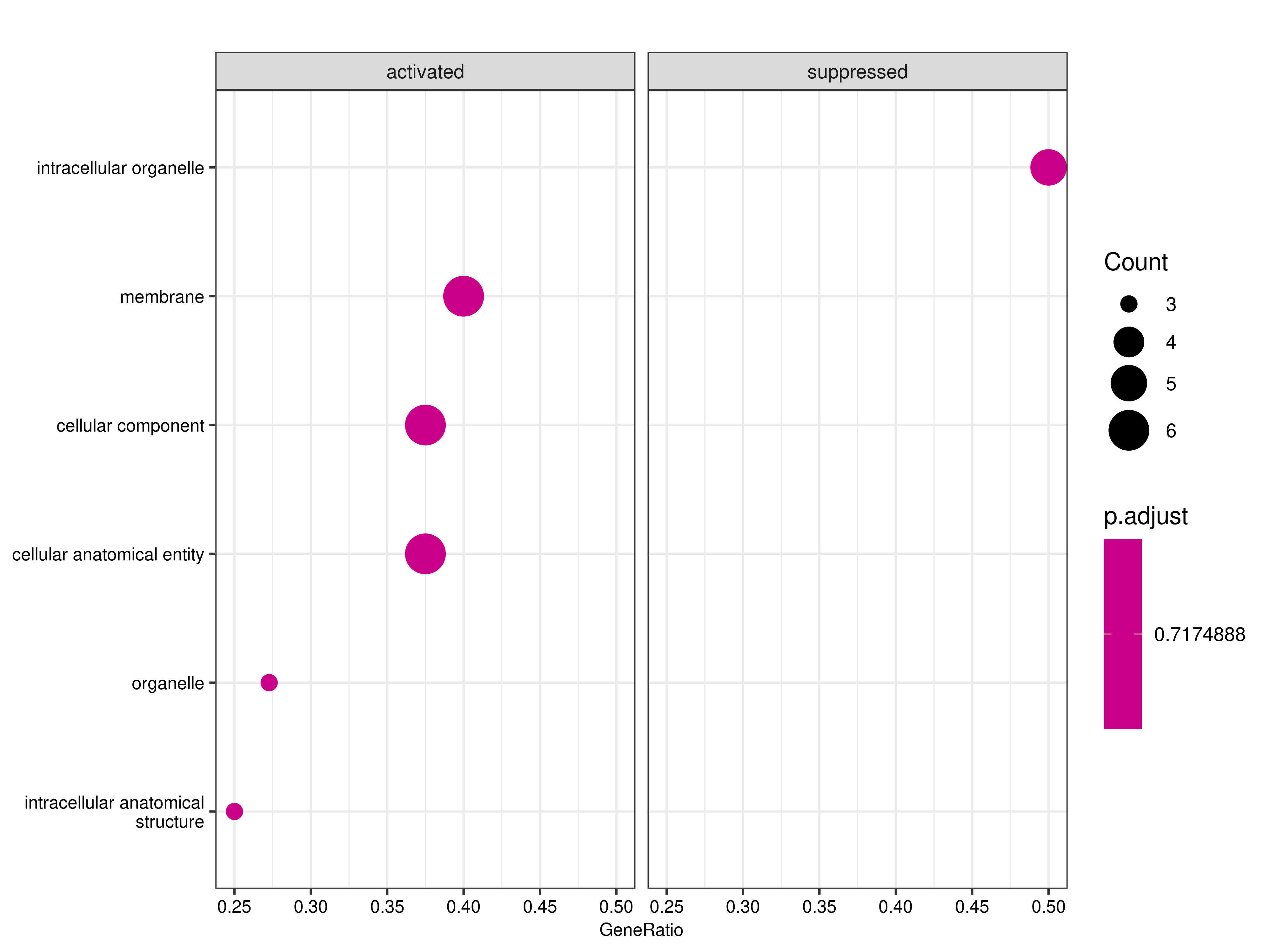

Supplement: Supplementary file 1 [file vaccines-12-00991-s001.zip › Supplementary File S3/proteome/4.Enrichment/gsea/3-infected_vs_3-uninfected/3-infected_vs_3-uninfected_GO_CC_GSEA_dotplot.png]

# membrane

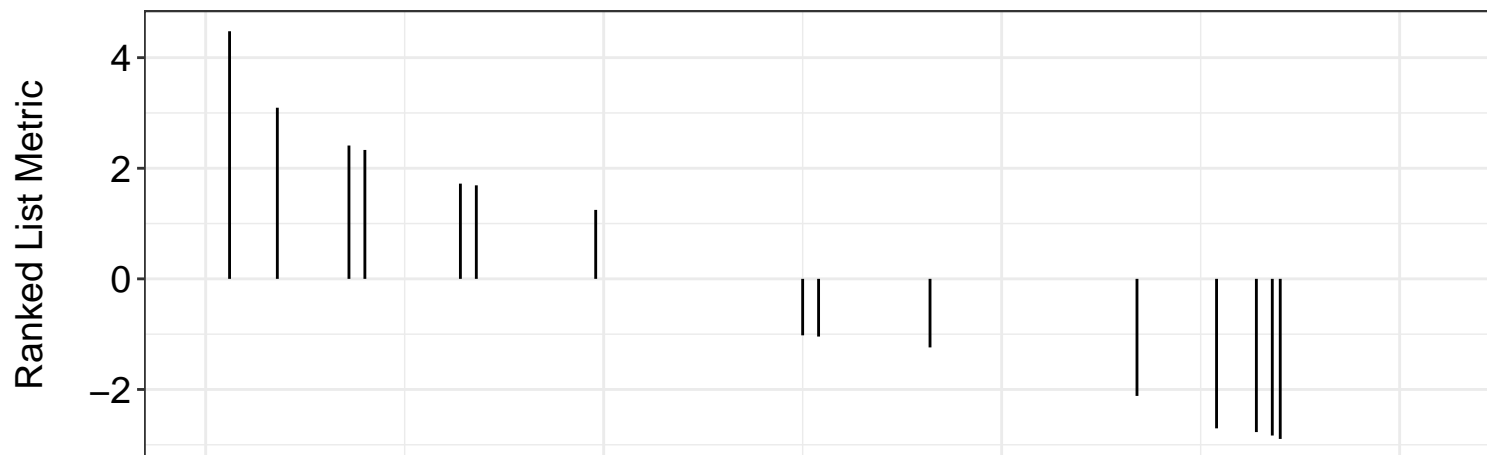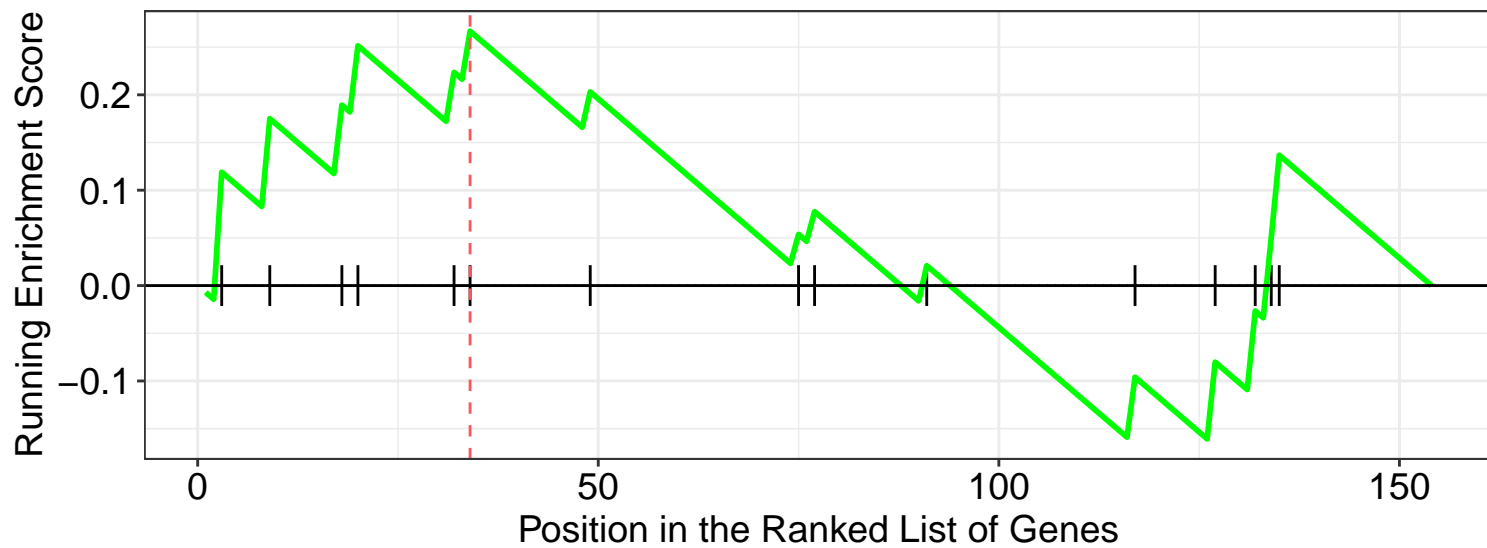

Supplement: Supplementary file 1 [file vaccines-12-00991-s001.zip › Supplementary File S3/proteome/4.Enrichment/gsea/3-infected_vs_3-uninfected/3-infected_vs_3-uninfected_GO_CC_GSEA_gseaplot.pdf]

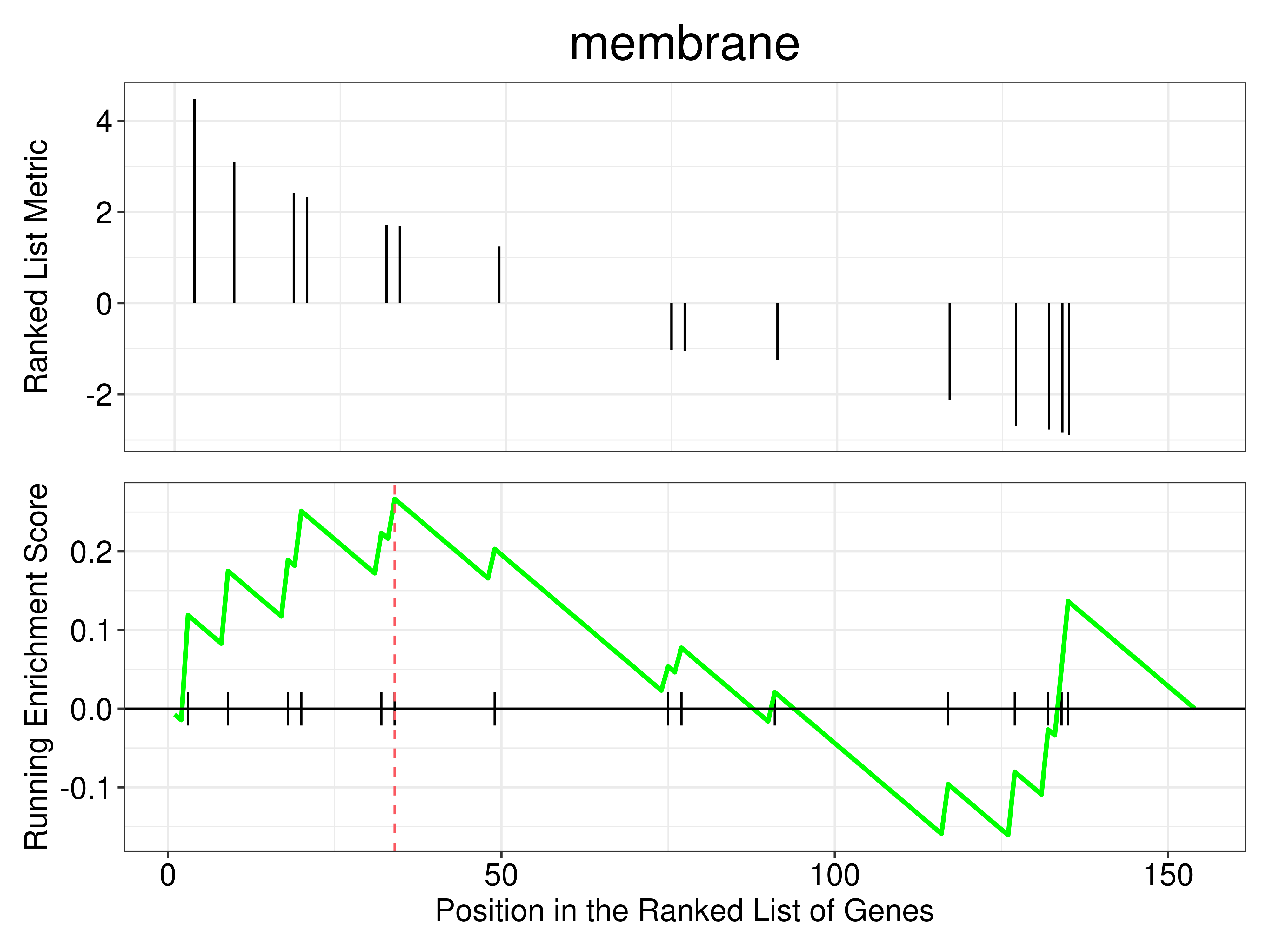

Supplement: Supplementary file 1 [file vaccines-12-00991-s001.zip › Supplementary File S3/proteome/4.Enrichment/gsea/3-infected_vs_3-uninfected/3-infected_vs_3-uninfected_GO_CC_GSEA_gseaplot.png]

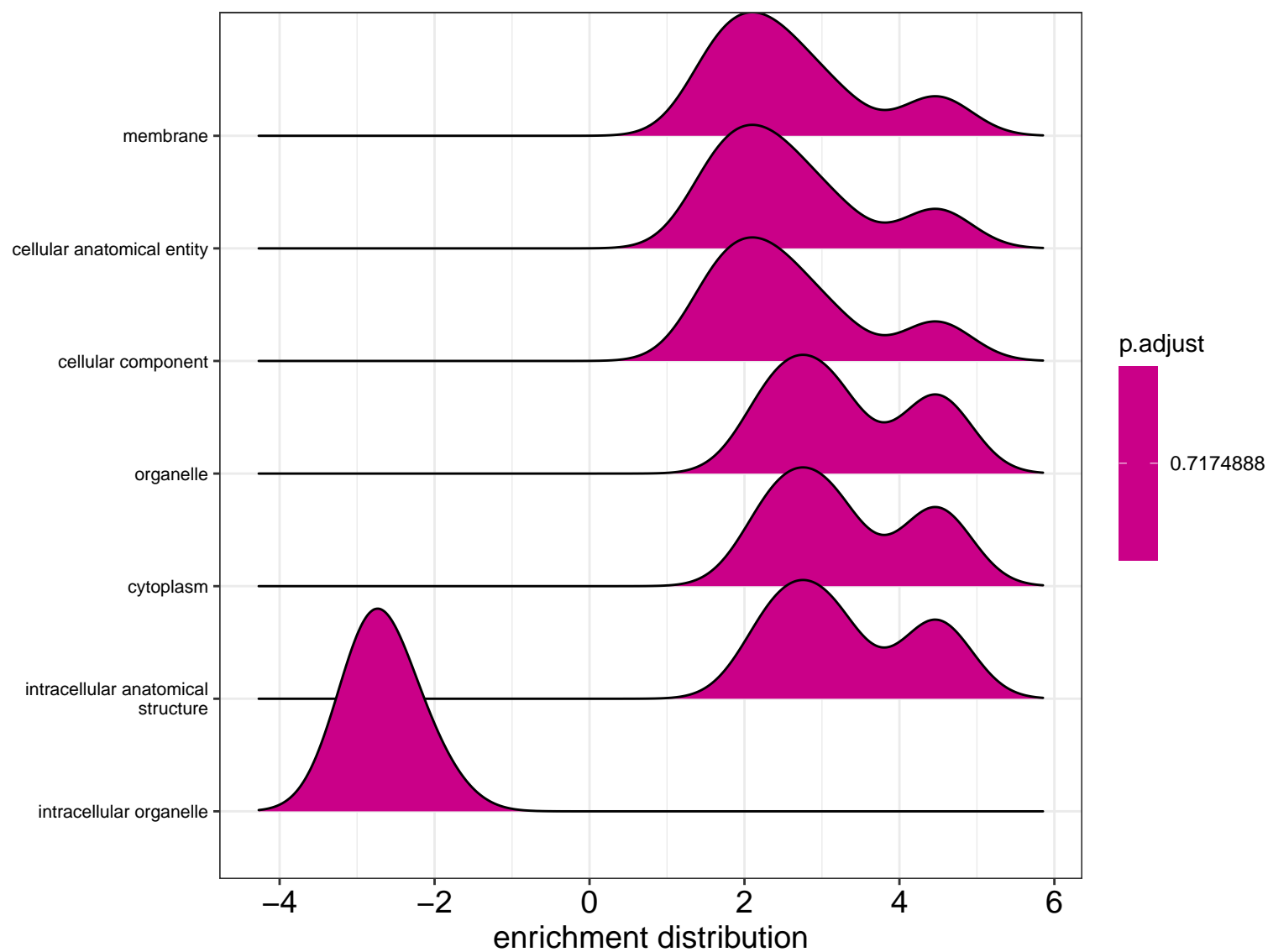

Supplement: Supplementary file 1 [file vaccines-12-00991-s001.zip › Supplementary File S3/proteome/4.Enrichment/gsea/3-infected_vs_3-uninfected/3-infected_vs_3-uninfected_GO_CC_GSEA_ridgeplot.pdf]

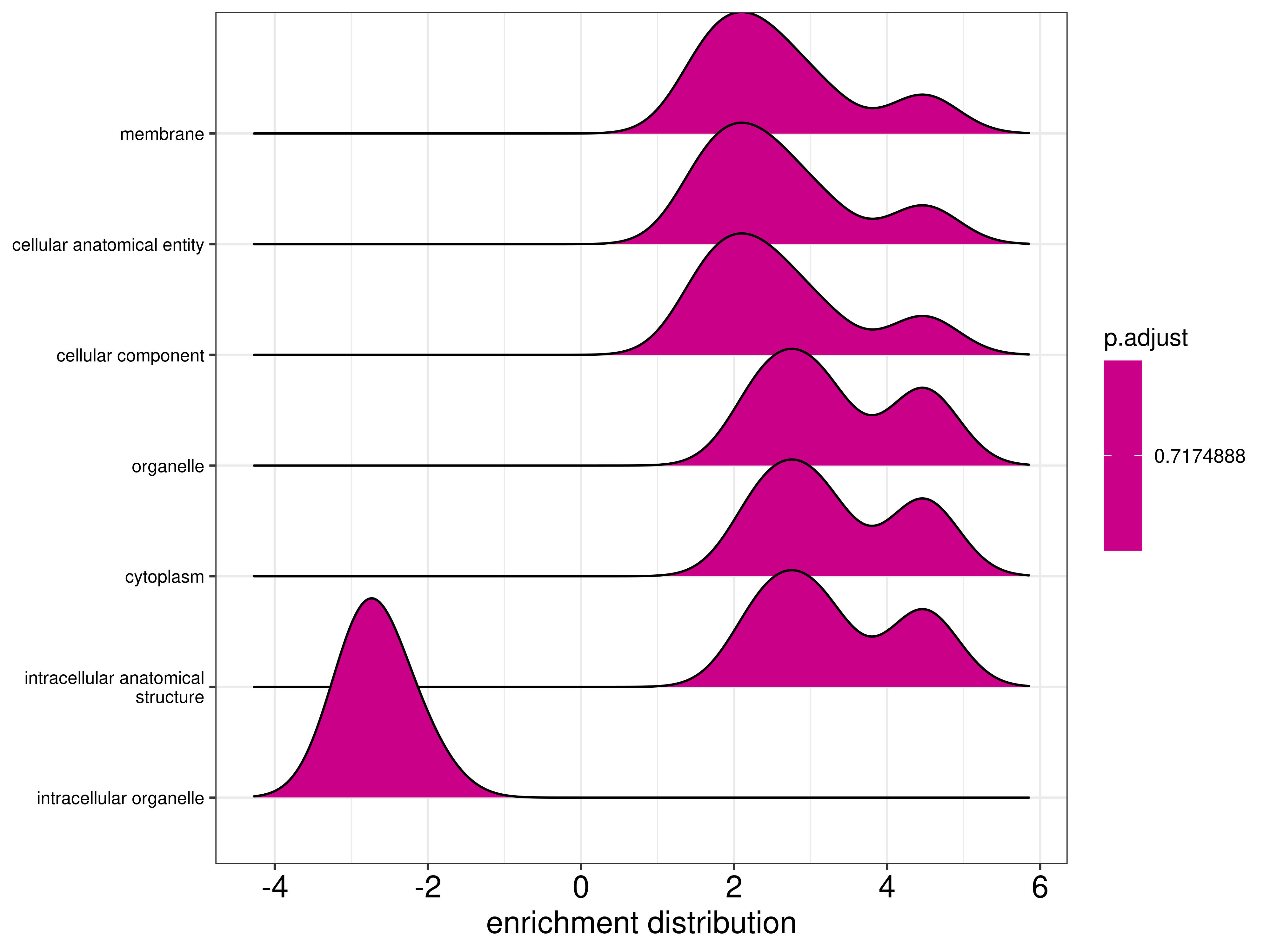

Supplement: Supplementary file 1 [file vaccines-12-00991-s001.zip › Supplementary File S3/proteome/4.Enrichment/gsea/3-infected_vs_3-uninfected/3-infected_vs_3-uninfected_GO_CC_GSEA_ridgeplot.png]

activated

Count

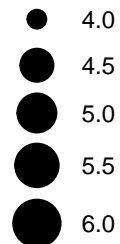

p.adjust

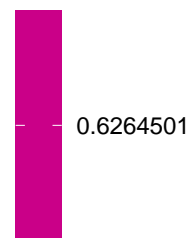

molecular function

binding

0.28

0.30

0.32

0.34

GeneRatio

Supplement: Supplementary file 1 [file vaccines-12-00991-s001.zip › Supplementary File S3/proteome/4.Enrichment/gsea/3-infected_vs_3-uninfected/3-infected_vs_3-uninfected_GO_MF_GSEA_dotplot.pdf]

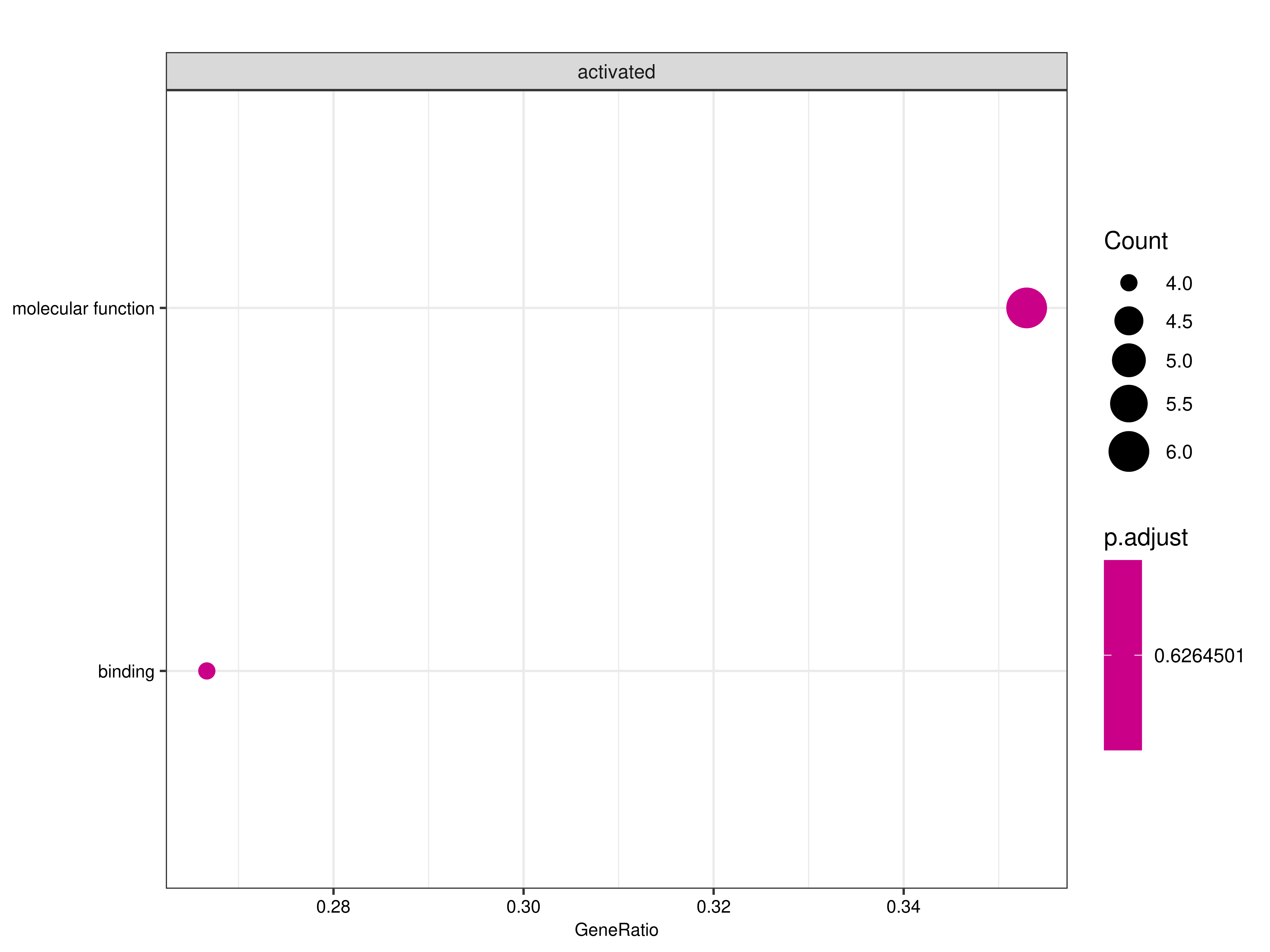

Supplement: Supplementary file 1 [file vaccines-12-00991-s001.zip › Supplementary File S3/proteome/4.Enrichment/gsea/3-infected_vs_3-uninfected/3-infected_vs_3-uninfected_GO_MF_GSEA_dotplot.png]

# binding

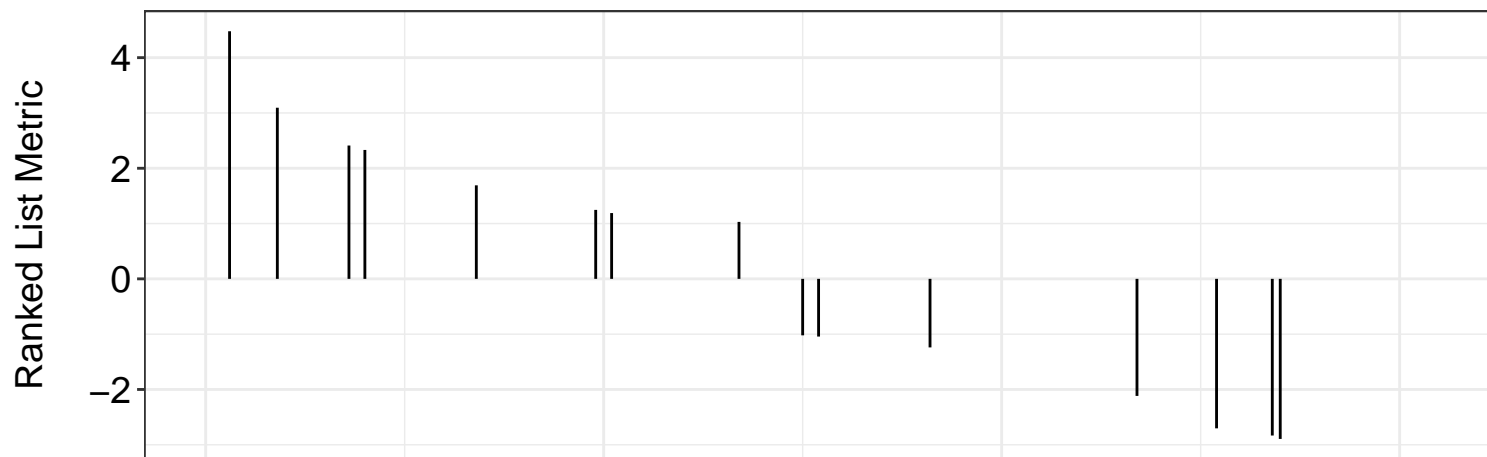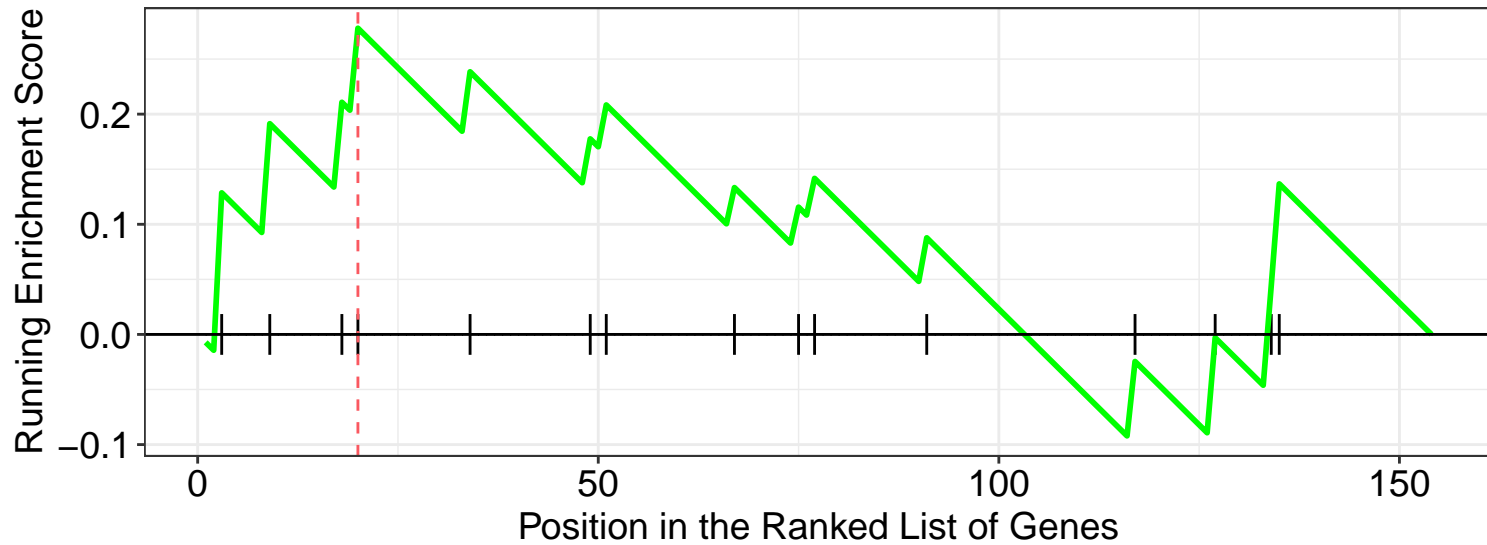

Supplement: Supplementary file 1 [file vaccines-12-00991-s001.zip › Supplementary File S3/proteome/4.Enrichment/gsea/3-infected_vs_3-uninfected/3-infected_vs_3-uninfected_GO_MF_GSEA_gseaplot.pdf]

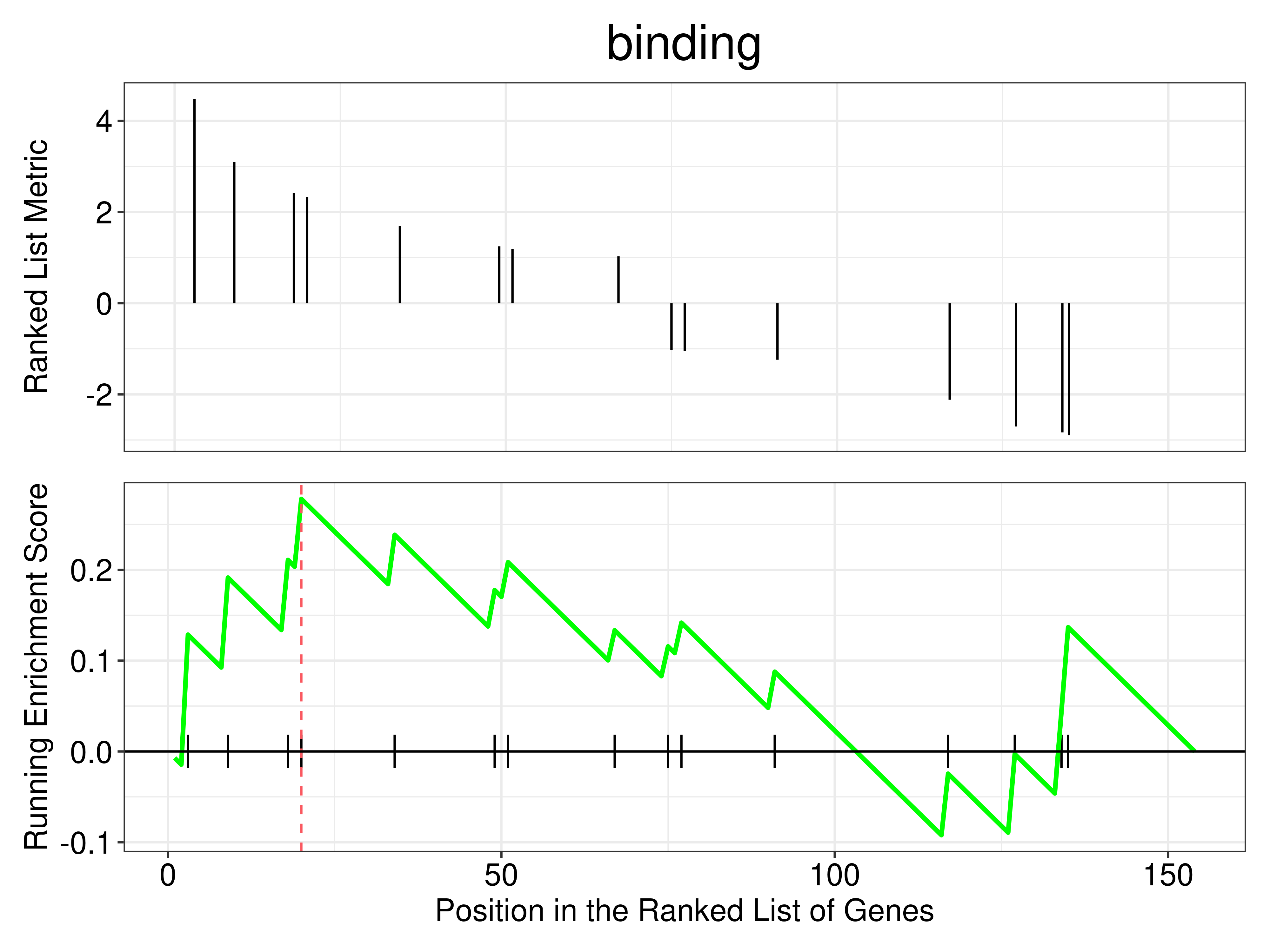

Supplement: Supplementary file 1 [file vaccines-12-00991-s001.zip › Supplementary File S3/proteome/4.Enrichment/gsea/3-infected_vs_3-uninfected/3-infected_vs_3-uninfected_GO_MF_GSEA_gseaplot.png]

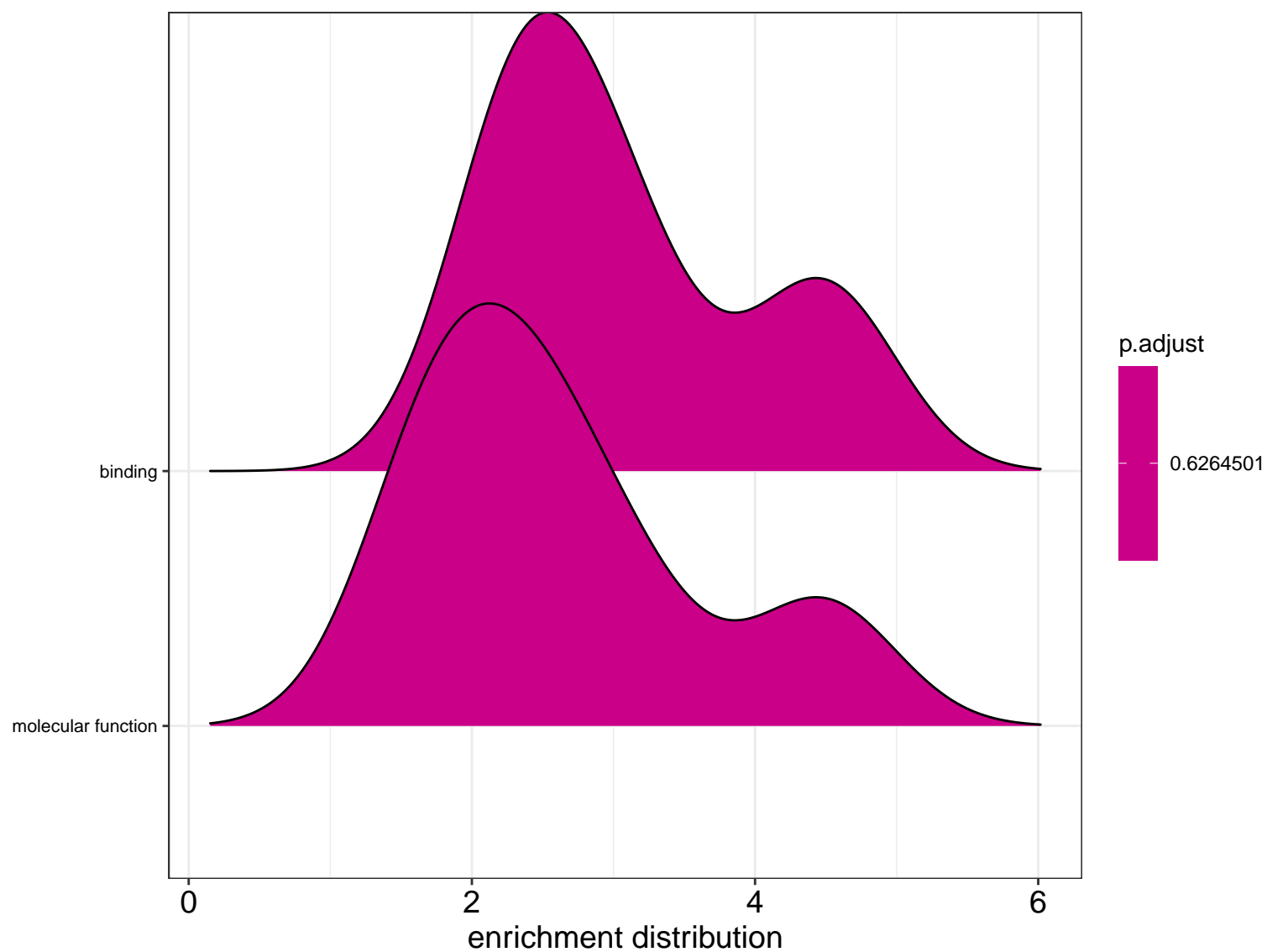

Supplement: Supplementary file 1 [file vaccines-12-00991-s001.zip › Supplementary File S3/proteome/4.Enrichment/gsea/3-infected_vs_3-uninfected/3-infected_vs_3-uninfected_GO_MF_GSEA_ridgeplot.pdf]

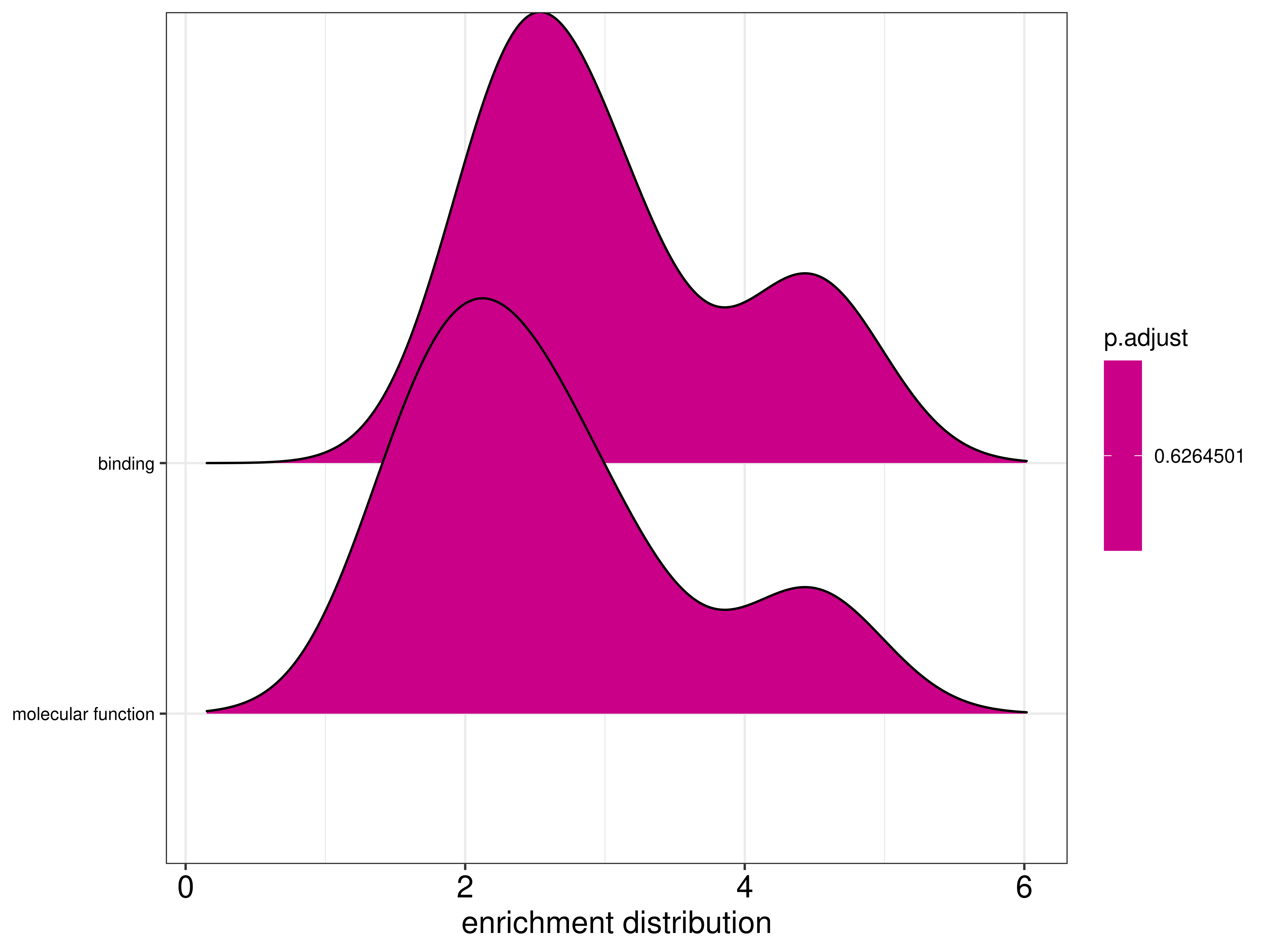

Supplement: Supplementary file 1 [file vaccines-12-00991-s001.zip › Supplementary File S3/proteome/4.Enrichment/gsea/3-infected_vs_3-uninfected/3-infected_vs_3-uninfected_GO_MF_GSEA_ridgeplot.png]

activated

Count

4

p.adjust

0.1525

Metabolic pathways

0.18

0.20

0.22

0.24

0.26

GeneRatio

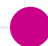

Supplement: Supplementary file 1 [file vaccines-12-00991-s001.zip › Supplementary File S3/proteome/4.Enrichment/gsea/3-infected_vs_3-uninfected/3-infected_vs_3-uninfected_KEGG_GSEA_dotplot.pdf]

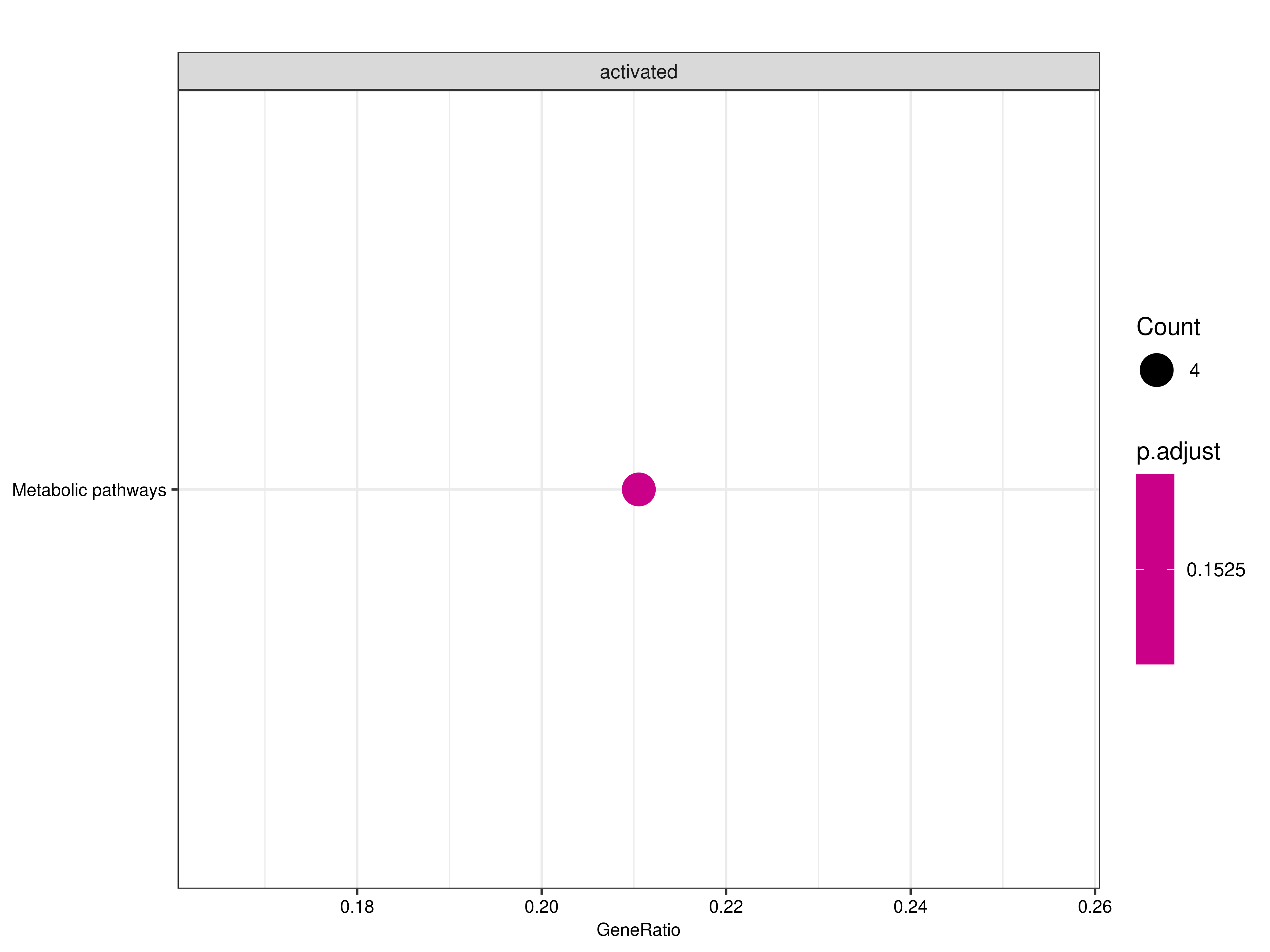

Supplement: Supplementary file 1 [file vaccines-12-00991-s001.zip › Supplementary File S3/proteome/4.Enrichment/gsea/3-infected_vs_3-uninfected/3-infected_vs_3-uninfected_KEGG_GSEA_dotplot.png]

# Metabolic pathways

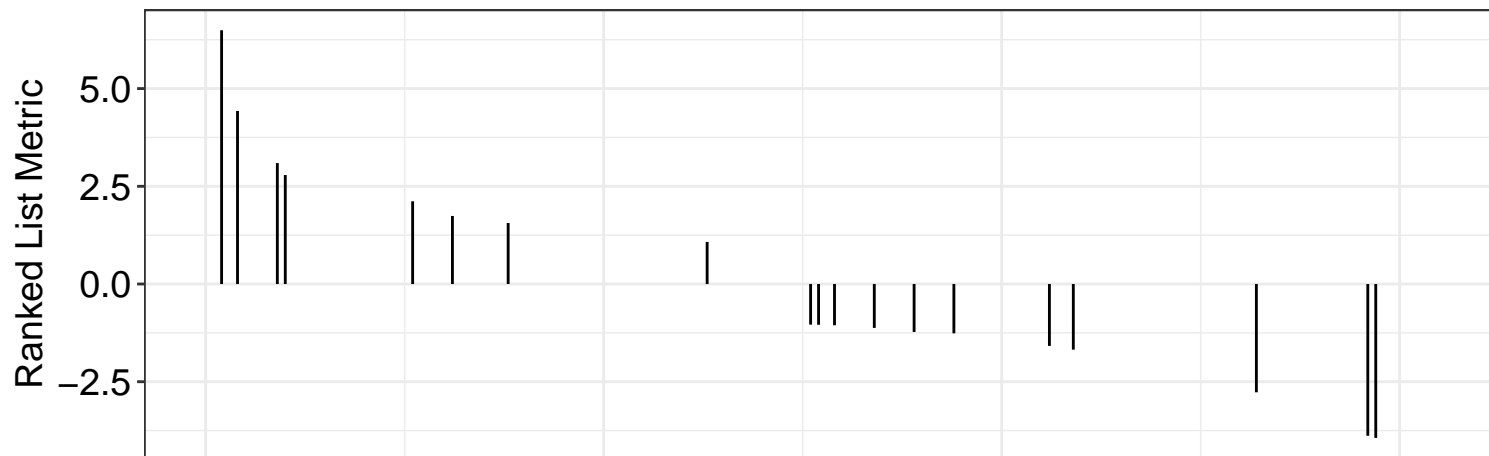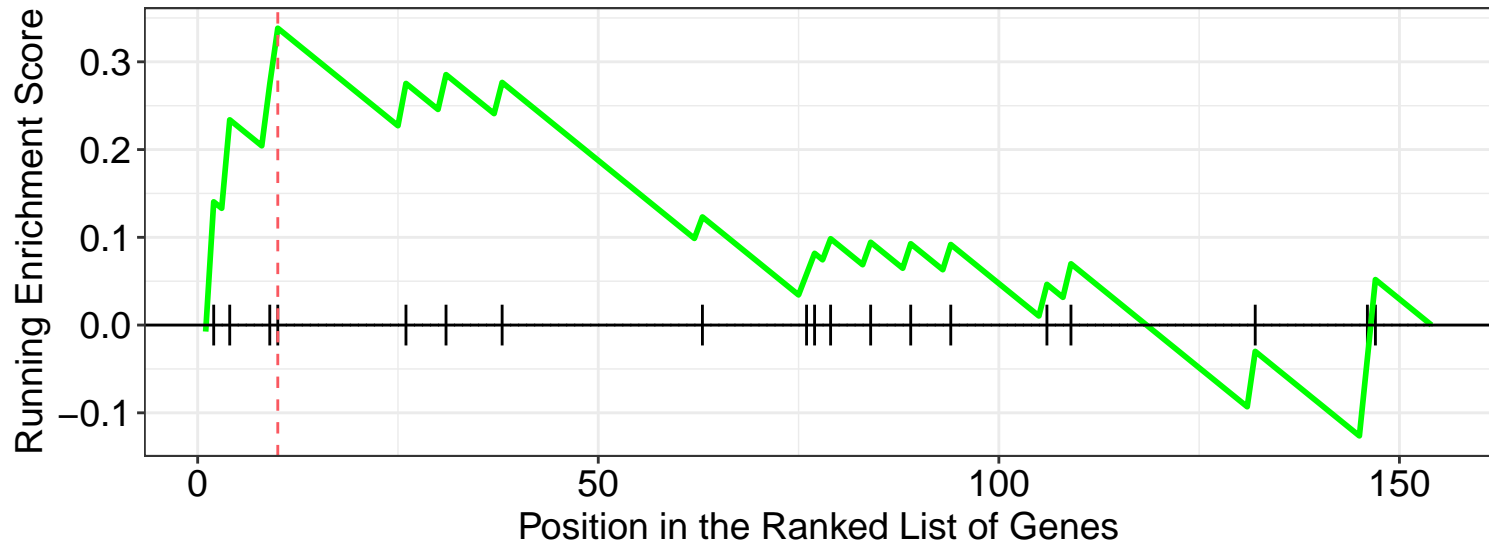

Supplement: Supplementary file 1 [file vaccines-12-00991-s001.zip › Supplementary File S3/proteome/4.Enrichment/gsea/3-infected_vs_3-uninfected/3-infected_vs_3-uninfected_KEGG_GSEA_gseaplot.pdf]

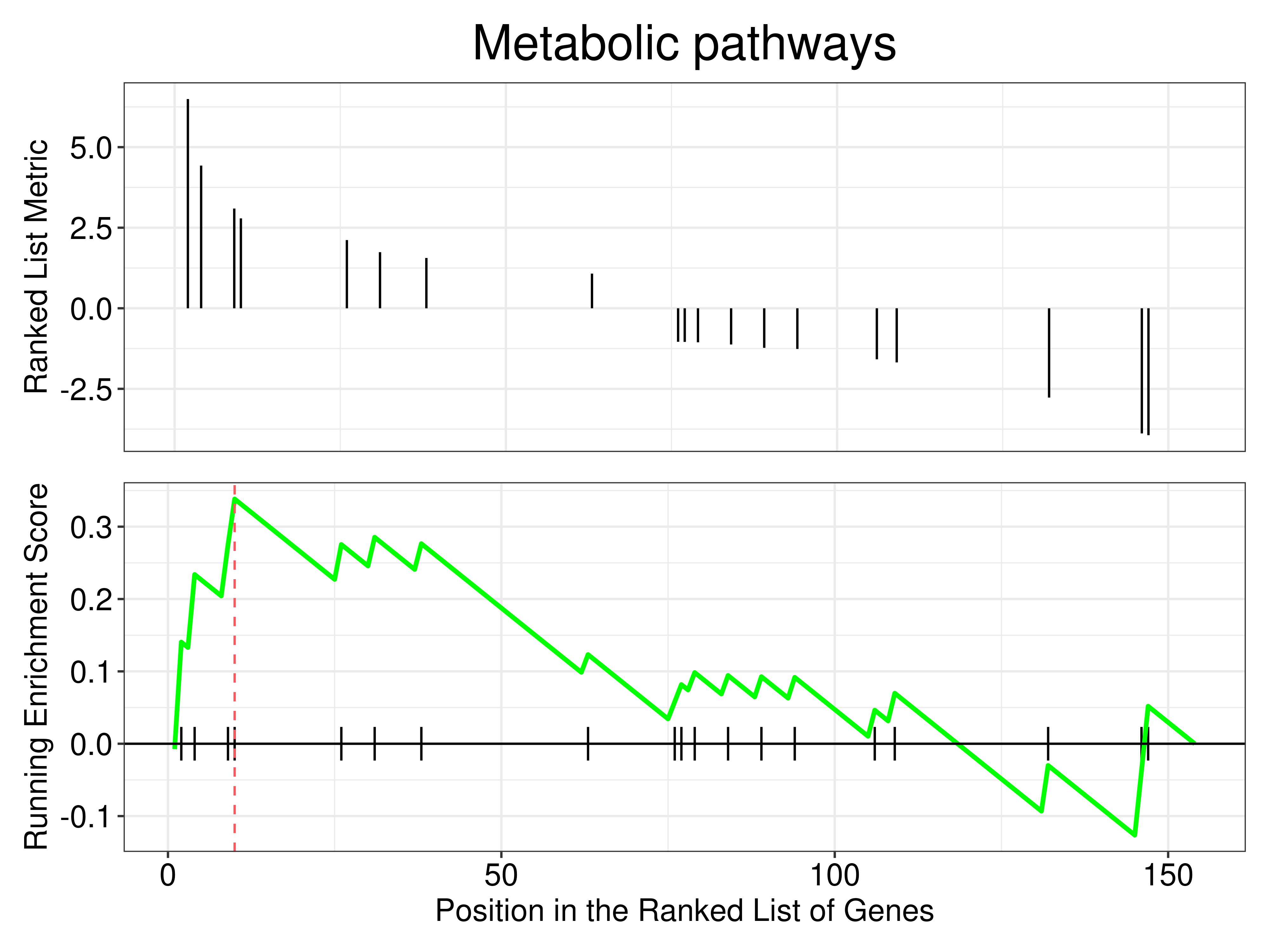

Supplement: Supplementary file 1 [file vaccines-12-00991-s001.zip › Supplementary File S3/proteome/4.Enrichment/gsea/3-infected_vs_3-uninfected/3-infected_vs_3-uninfected_KEGG_GSEA_gseaplot.png]

Metabolic pathways

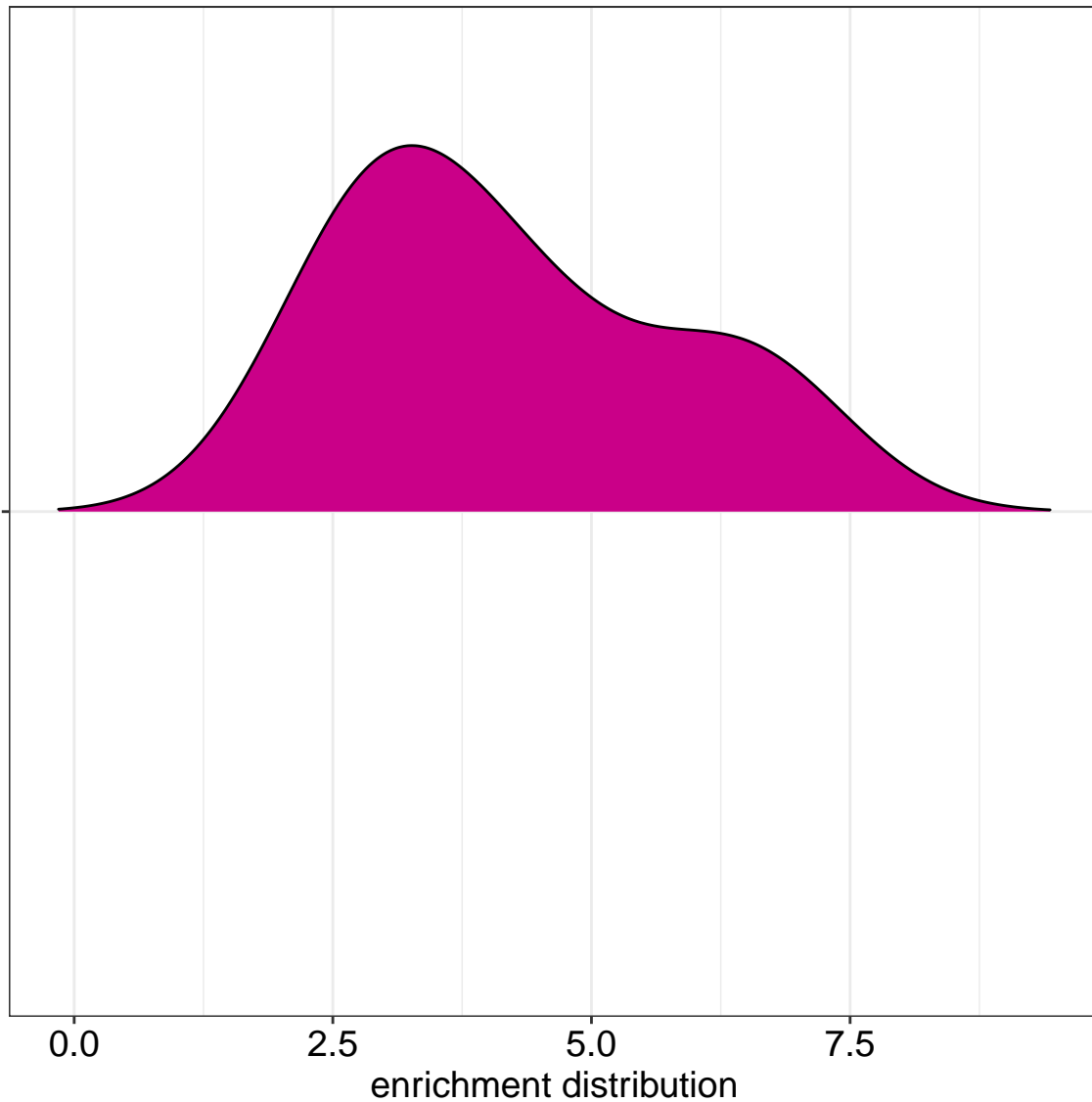

p.adjust

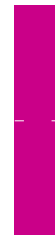

0.1525

Supplement: Supplementary file 1 [file vaccines-12-00991-s001.zip › Supplementary File S3/proteome/4.Enrichment/gsea/3-infected_vs_3-uninfected/3-infected_vs_3-uninfected_KEGG_GSEA_ridgeplot.pdf]

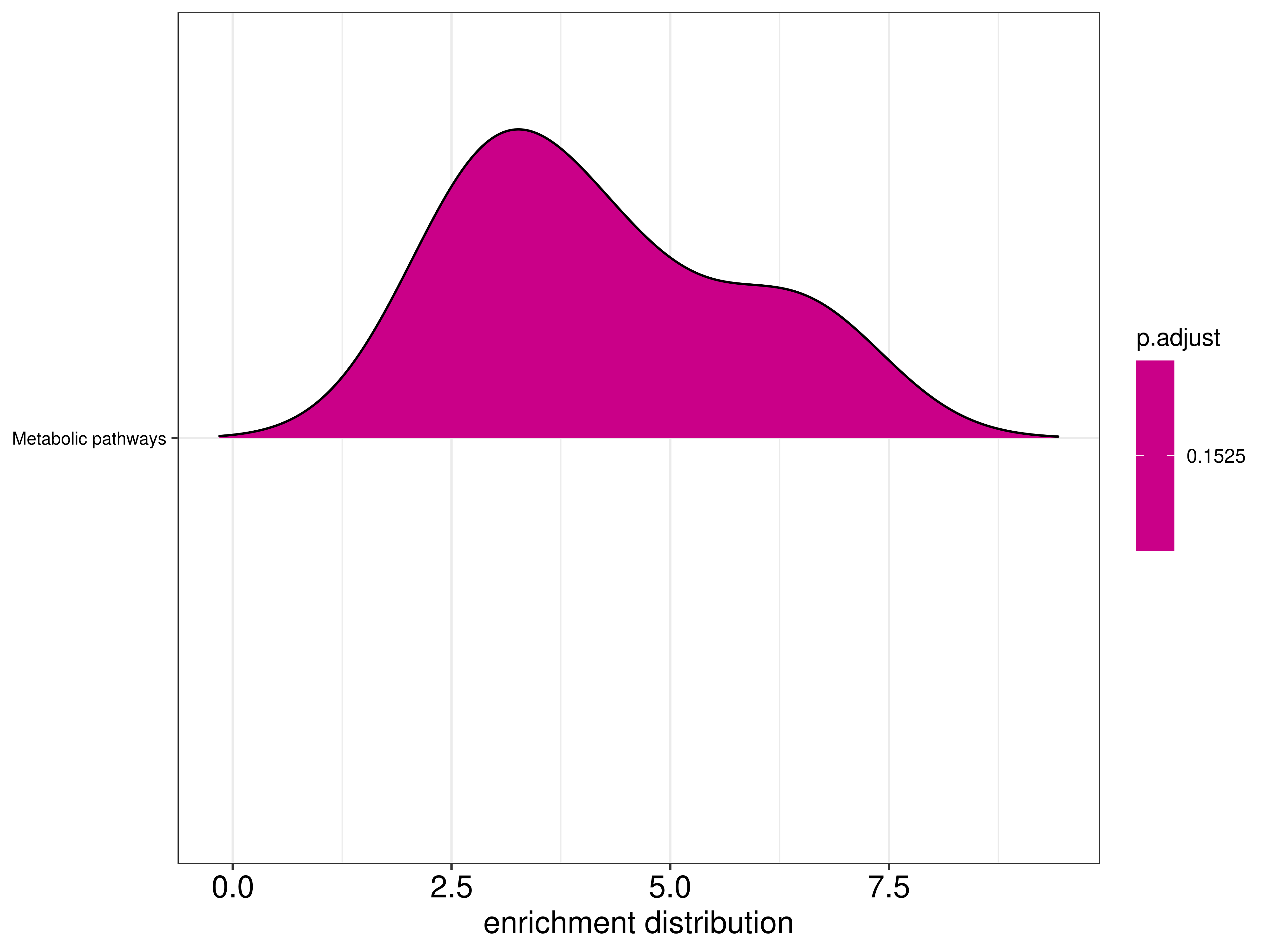

Supplement: Supplementary file 1 [file vaccines-12-00991-s001.zip › Supplementary File S3/proteome/4.Enrichment/gsea/3-infected_vs_3-uninfected/3-infected_vs_3-uninfected_KEGG_GSEA_ridgeplot.png]

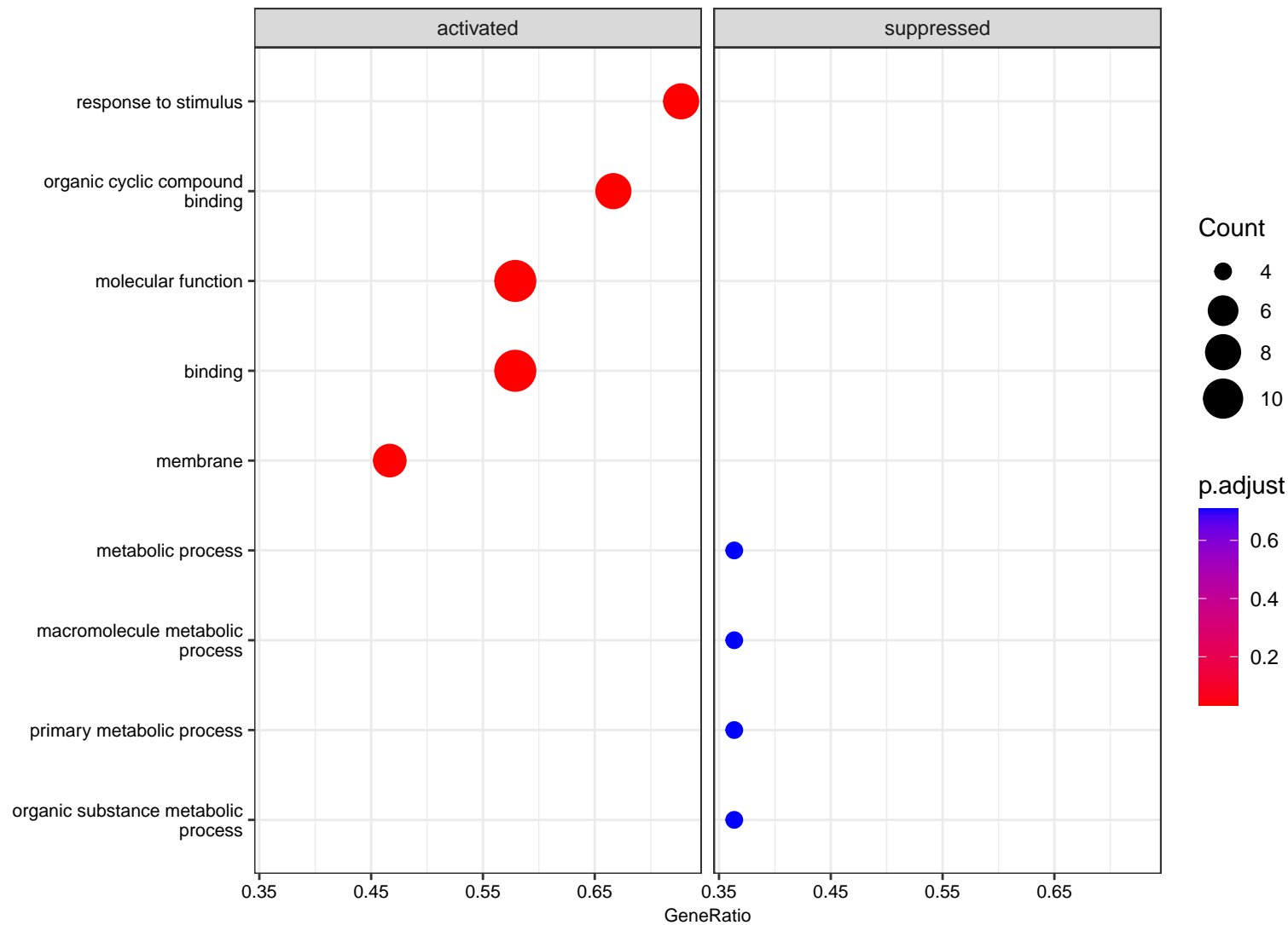

Supplement: Supplementary file 1 [file vaccines-12-00991-s001.zip › Supplementary File S3/proteome/4.Enrichment/gsea/5-infected_vs_5-uninfected/5-infected_vs_5-uninfected_GO_ALL_GSEA_dotplot.pdf]

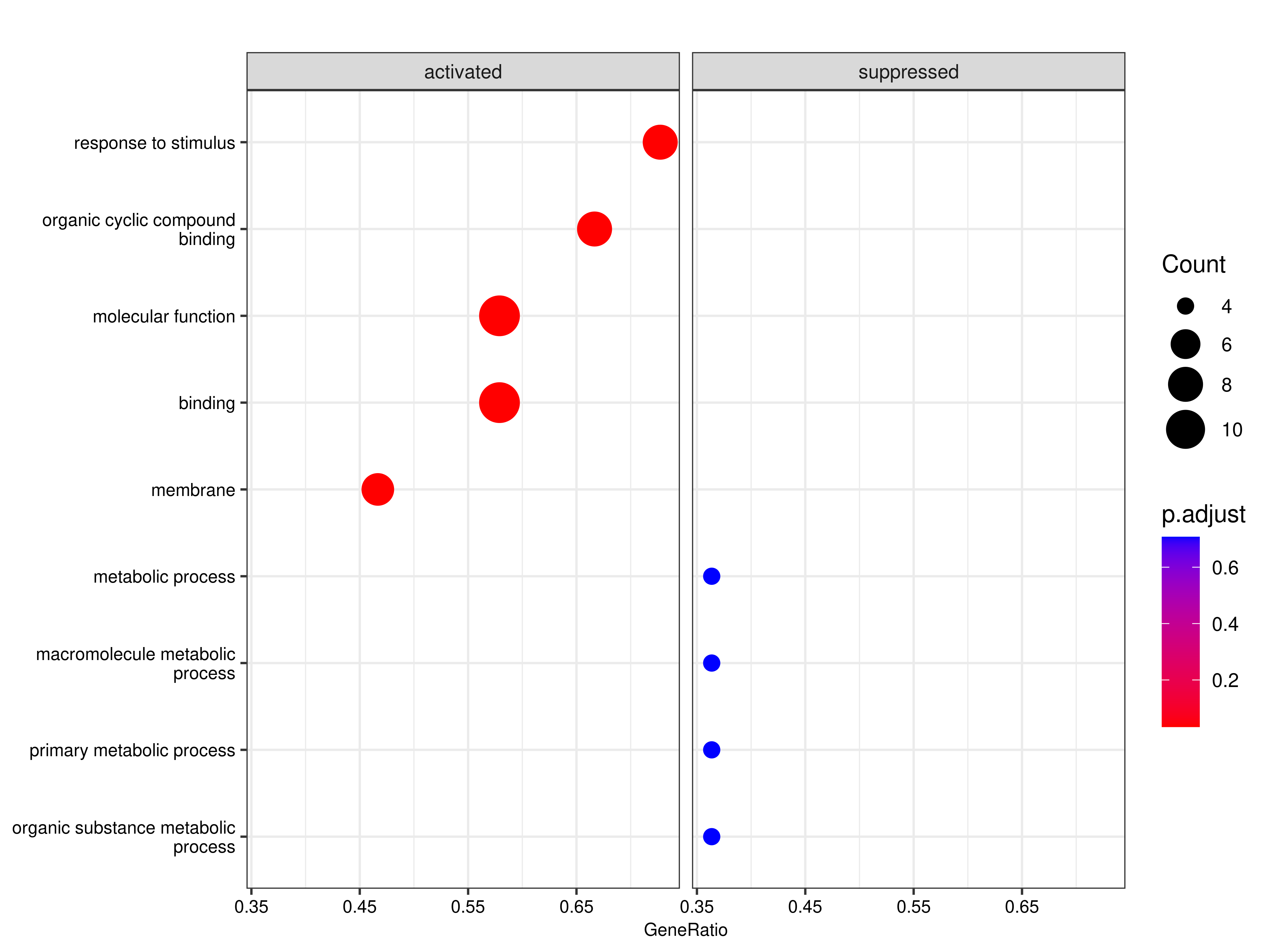

Supplement: Supplementary file 1 [file vaccines-12-00991-s001.zip › Supplementary File S3/proteome/4.Enrichment/gsea/5-infected_vs_5-uninfected/5-infected_vs_5-uninfected_GO_ALL_GSEA_dotplot.png]

# membrane

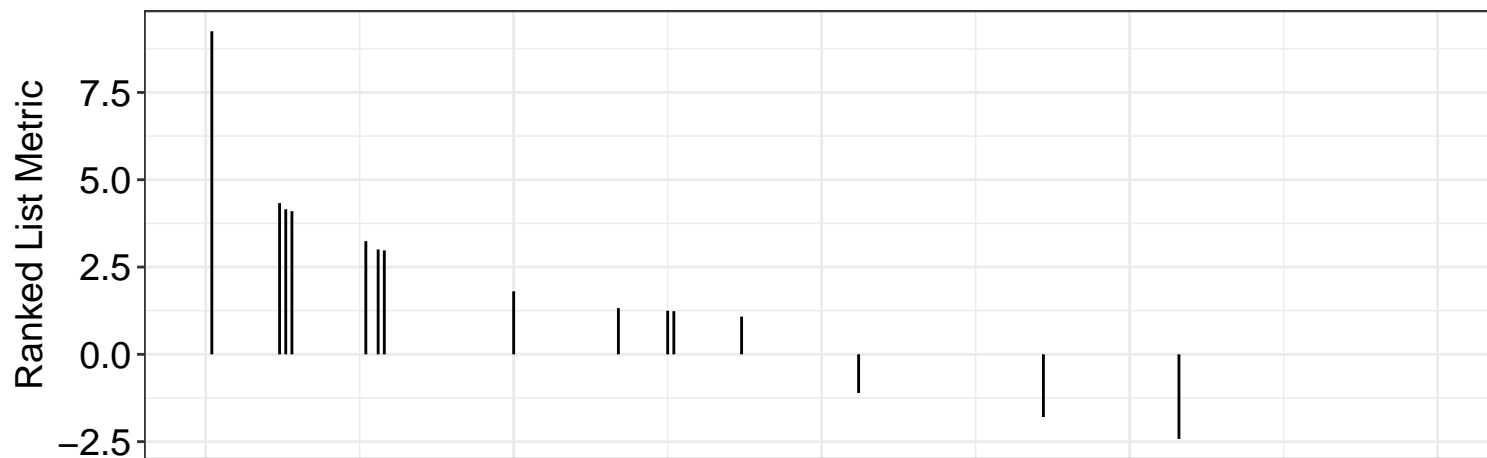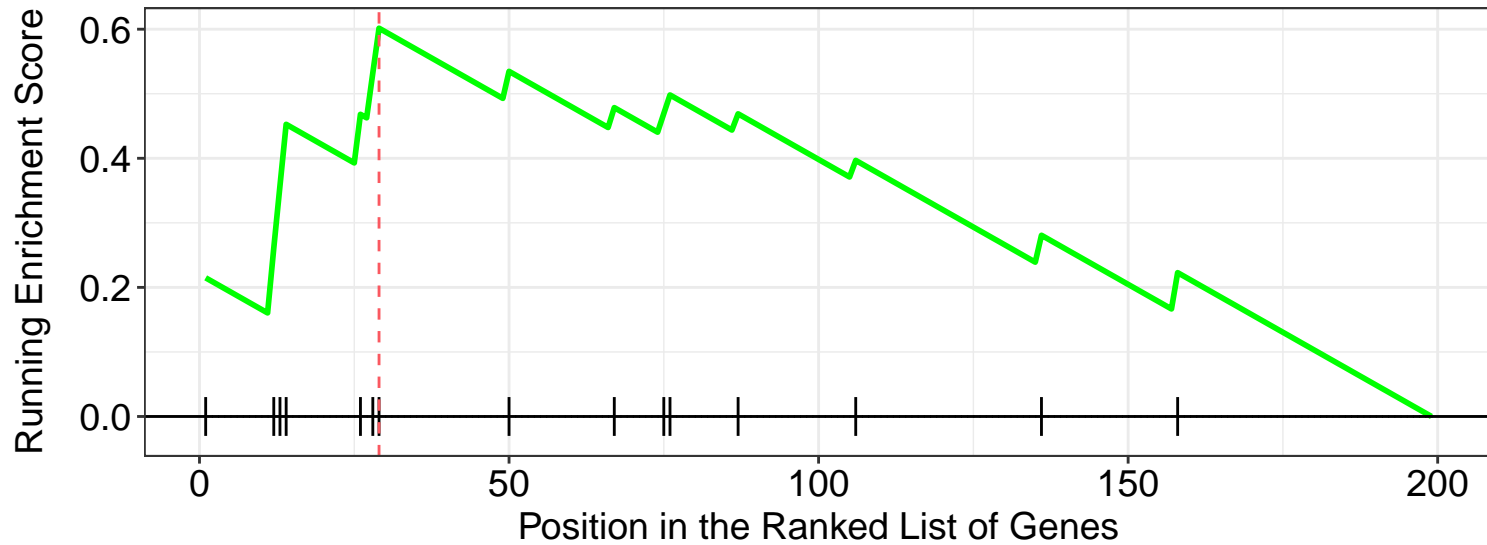

Supplement: Supplementary file 1 [file vaccines-12-00991-s001.zip › Supplementary File S3/proteome/4.Enrichment/gsea/5-infected_vs_5-uninfected/5-infected_vs_5-uninfected_GO_ALL_GSEA_gseaplot.pdf]

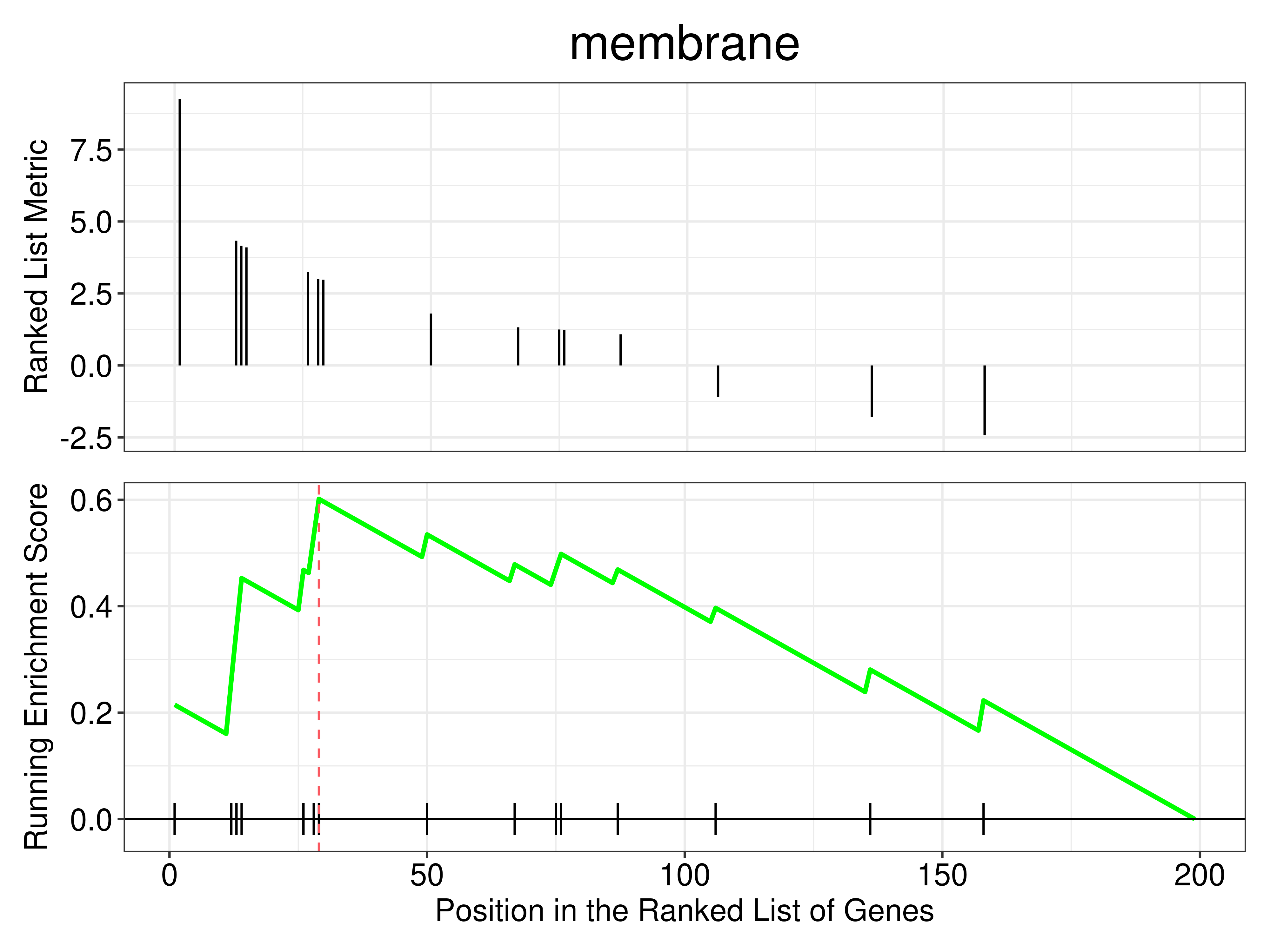

Supplement: Supplementary file 1 [file vaccines-12-00991-s001.zip › Supplementary File S3/proteome/4.Enrichment/gsea/5-infected_vs_5-uninfected/5-infected_vs_5-uninfected_GO_ALL_GSEA_gseaplot.png]

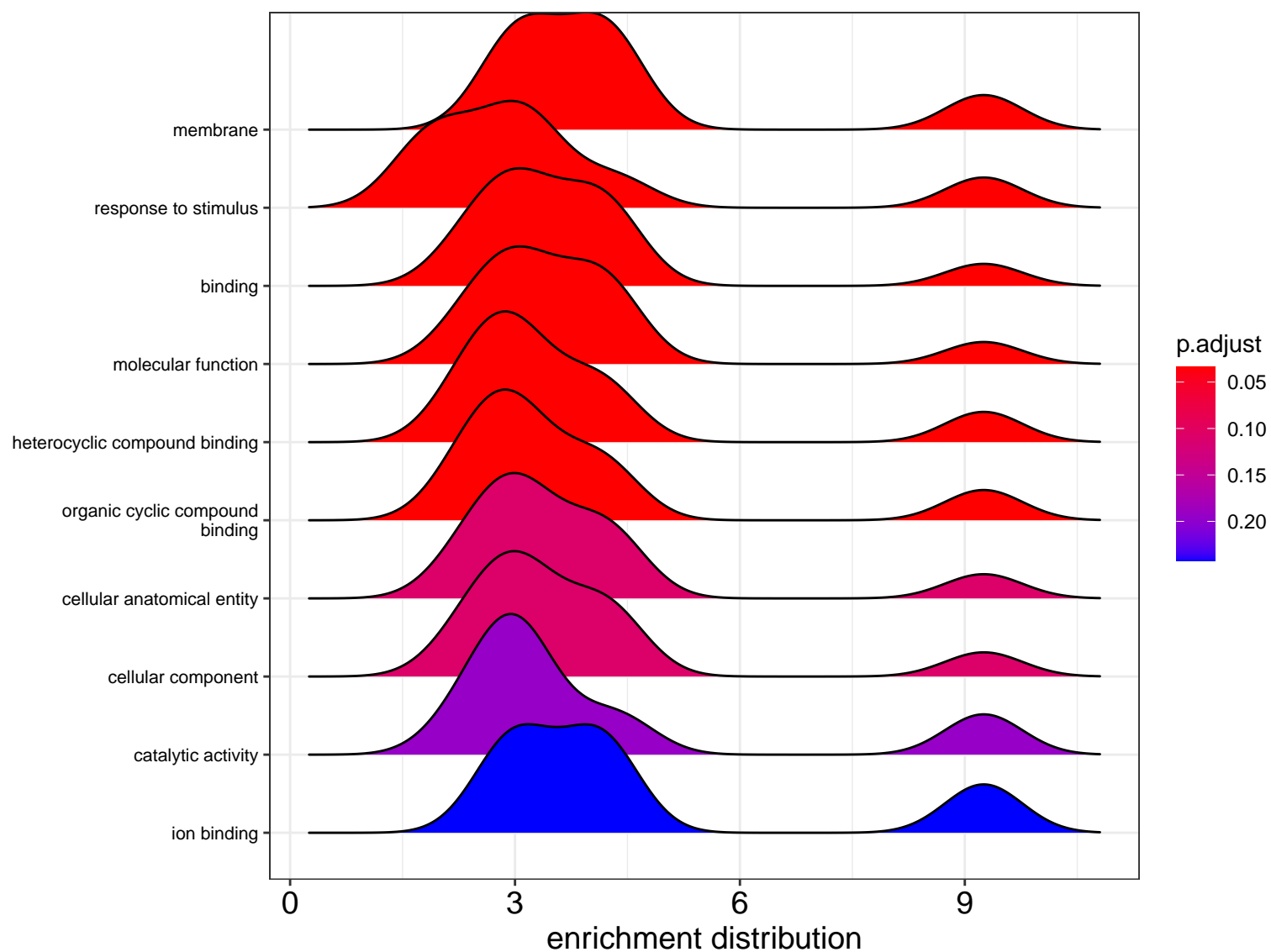

Supplement: Supplementary file 1 [file vaccines-12-00991-s001.zip › Supplementary File S3/proteome/4.Enrichment/gsea/5-infected_vs_5-uninfected/5-infected_vs_5-uninfected_GO_ALL_GSEA_ridgeplot.pdf]

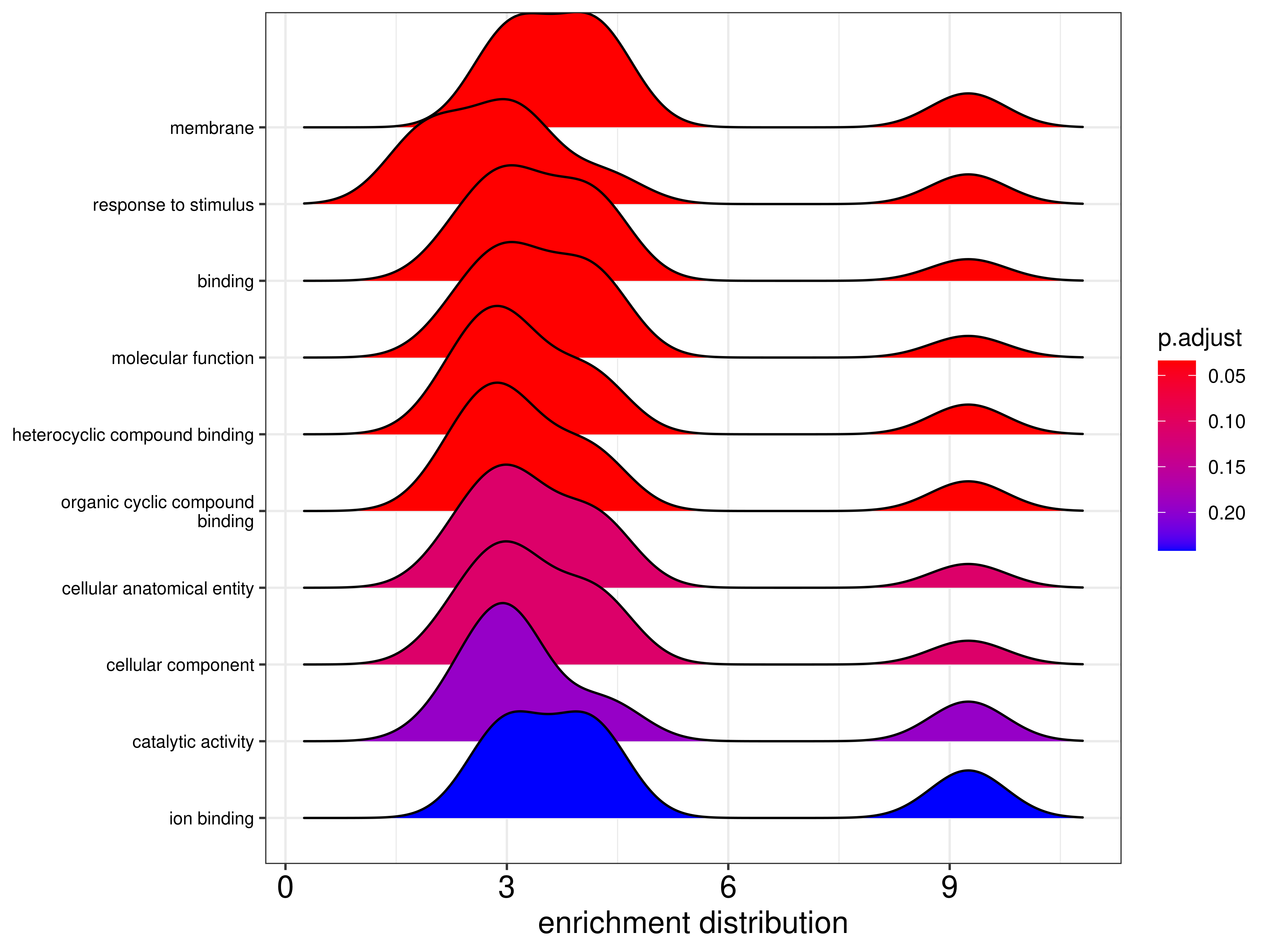

Supplement: Supplementary file 1 [file vaccines-12-00991-s001.zip › Supplementary File S3/proteome/4.Enrichment/gsea/5-infected_vs_5-uninfected/5-infected_vs_5-uninfected_GO_ALL_GSEA_ridgeplot.png]

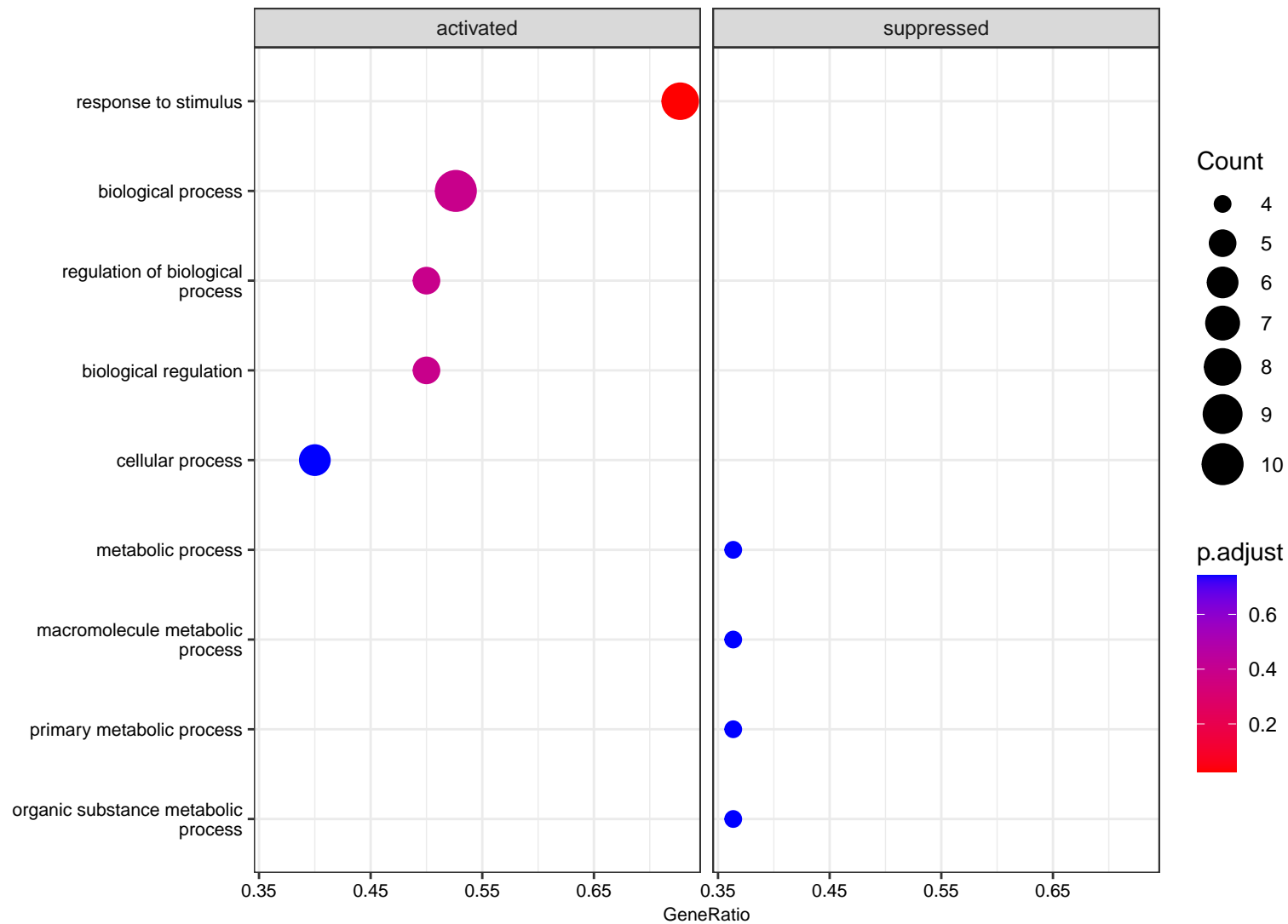

Supplement: Supplementary file 1 [file vaccines-12-00991-s001.zip › Supplementary File S3/proteome/4.Enrichment/gsea/5-infected_vs_5-uninfected/5-infected_vs_5-uninfected_GO_BP_GSEA_dotplot.pdf]

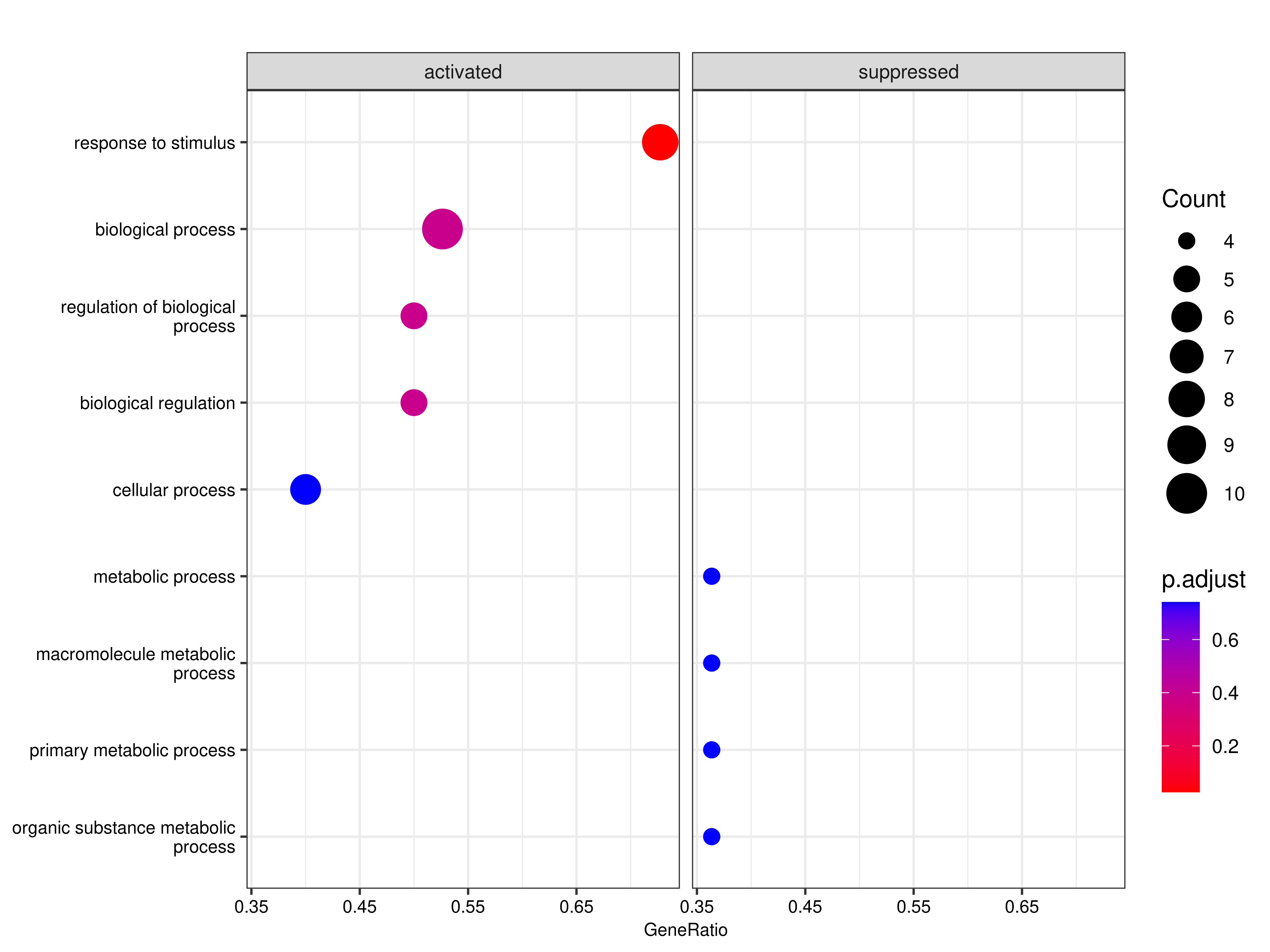

Supplement: Supplementary file 1 [file vaccines-12-00991-s001.zip › Supplementary File S3/proteome/4.Enrichment/gsea/5-infected_vs_5-uninfected/5-infected_vs_5-uninfected_GO_BP_GSEA_dotplot.png]

# response to stimulus

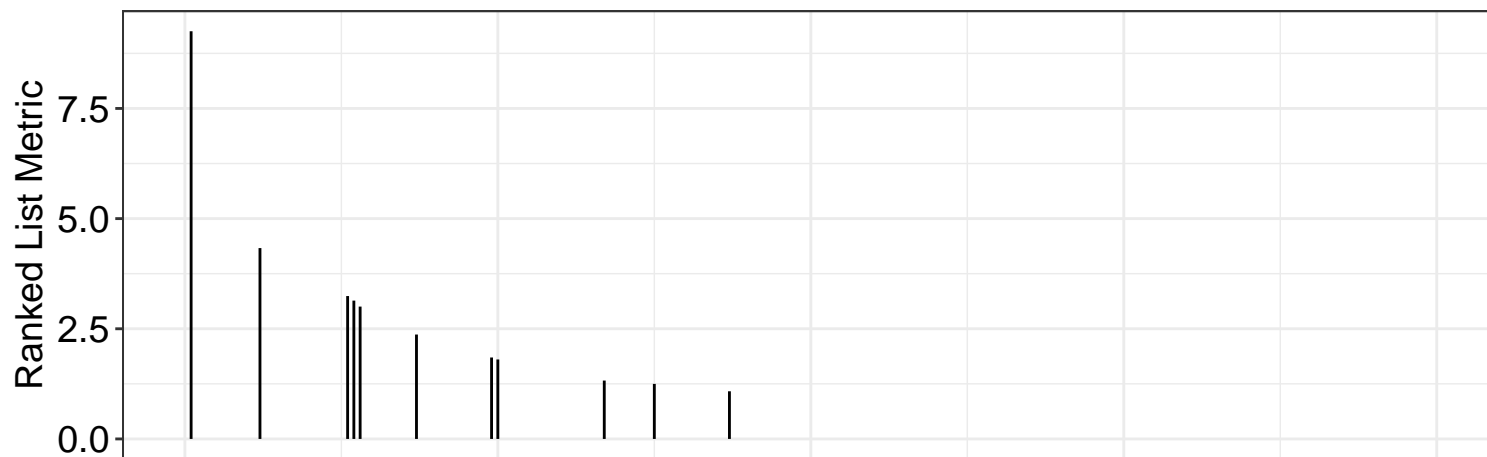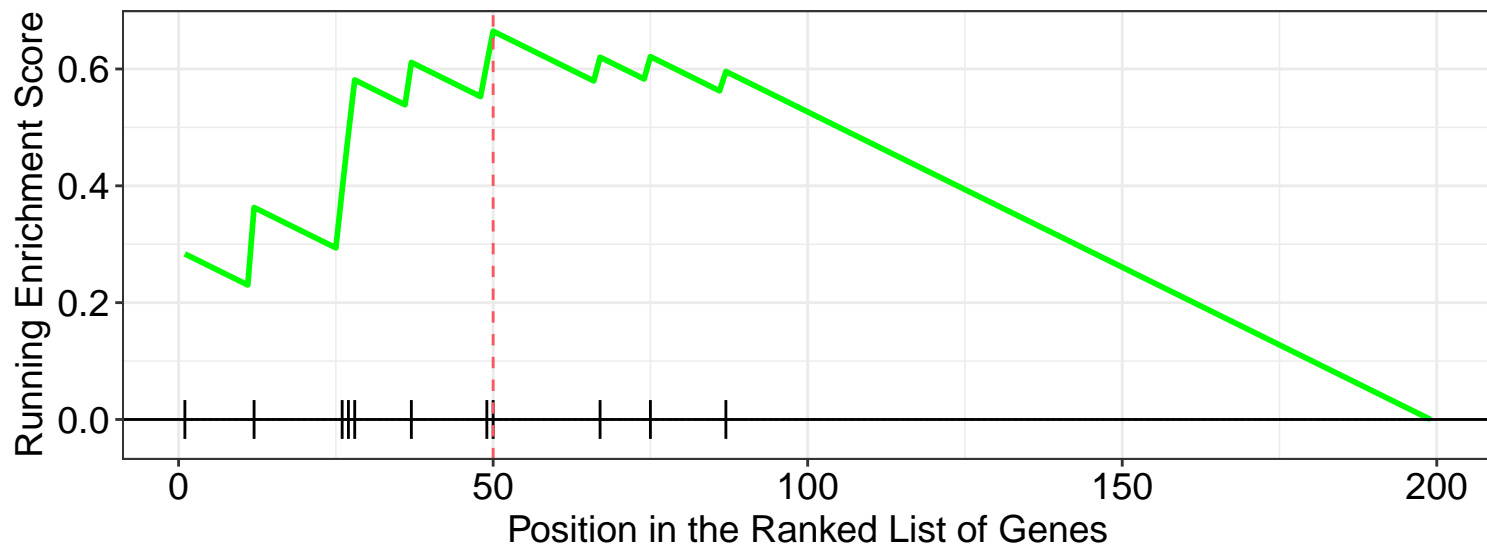

Supplement: Supplementary file 1 [file vaccines-12-00991-s001.zip › Supplementary File S3/proteome/4.Enrichment/gsea/5-infected_vs_5-uninfected/5-infected_vs_5-uninfected_GO_BP_GSEA_gseaplot.pdf]
