# Supplementary material for: Production, Passaging Stability, and Histological Analysis of Madin–Darby Canine Kidney Cells Cultured in a Low-Serum Medium
Source: Vaccines (Basel). 2024 Aug 30;12(9):991. doi: 10.3390/vaccines12090991 (PMC11435615; doi:10.3390/vaccines12090991)
Supplement: Supplementary file 1 [file vaccines-12-00991-s001.zip › Supplementary File S3/transcriptome/1.QualityControl/trim/3-2-infected_trim.pdf]

3−2−infected

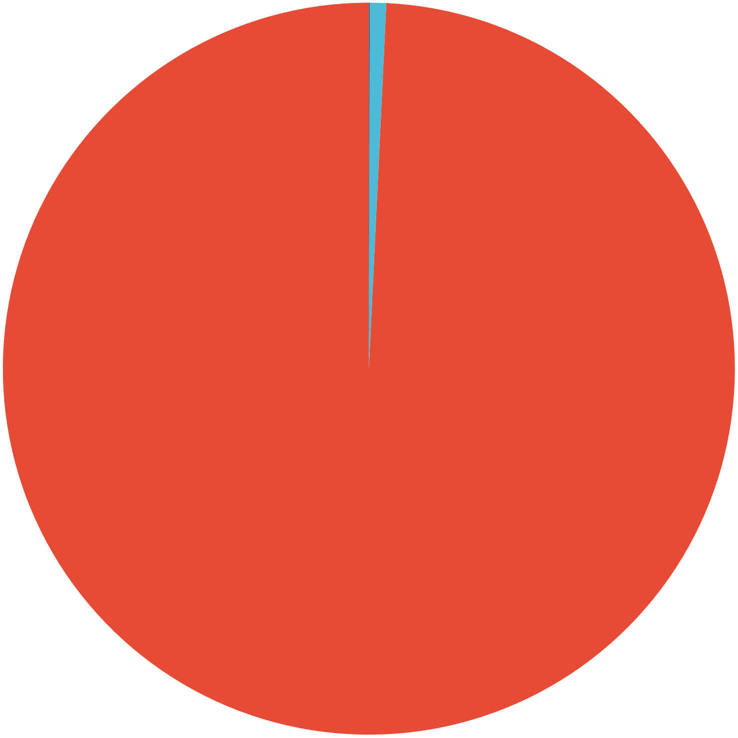

Passed_Filter_Reads: 61,849,412 (99.24%)
Low_Quality_Reads: 445,750 (0.72%)
Too_Many_N_Reads: 1,462 (0%)
Too_Short_Reads: 26,710 (0.04%)
