# Supplementary material for: Production, Passaging Stability, and Histological Analysis of Madin–Darby Canine Kidney Cells Cultured in a Low-Serum Medium
Source: Vaccines (Basel). 2024 Aug 30;12(9):991. doi: 10.3390/vaccines12090991 (PMC11435615; doi:10.3390/vaccines12090991)
Supplement: Supplementary file 1 [file vaccines-12-00991-s001.zip › Supplementary File S3/transcriptome/1.QualityControl/trim/5-2-uninfected_trim.pdf]

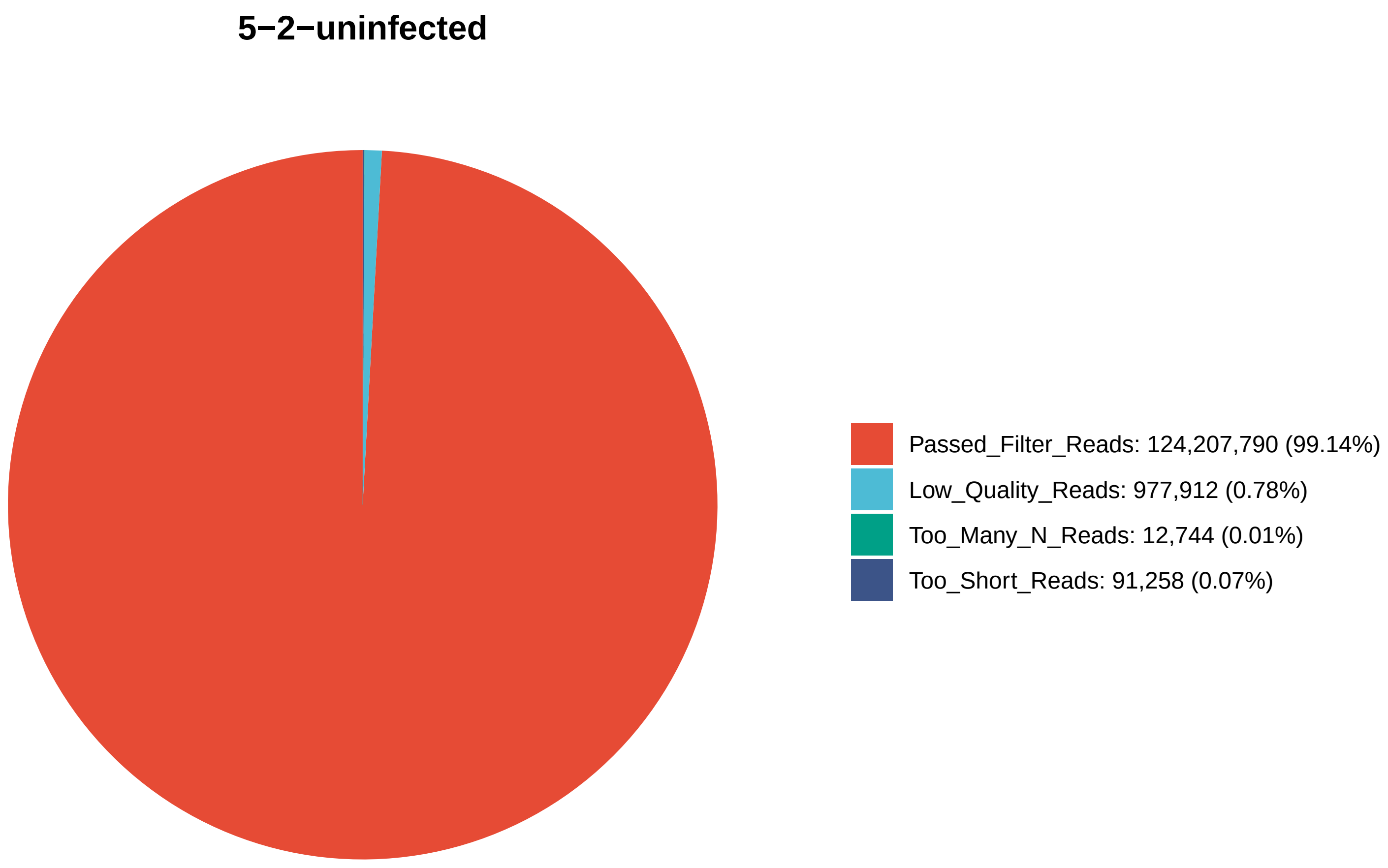
5−2−uninfected
Passed_Filter_Reads: 124,207,790 (99.14%)
Low_Quality_Reads: 977,912 (0.78%)
Too_Many_N_Reads: 12,744 (0.01%)
Too_Short_Reads: 91,258 (0.07%)
