# Supplementary material for: Production, Passaging Stability, and Histological Analysis of Madin–Darby Canine Kidney Cells Cultured in a Low-Serum Medium
Source: Vaccines (Basel). 2024 Aug 30;12(9):991. doi: 10.3390/vaccines12090991 (PMC11435615; doi:10.3390/vaccines12090991)
Supplement: Supplementary file 1 [file vaccines-12-00991-s001.zip › Supplementary File S3/transcriptome/2.Mapping/qualimap/3-3-infected/qualimapReport.html]

Qualimap report: RNA Seq QC


Qualimap Report: RNA Seq QC

## Input data and parameters

### Input

|  |  |
| --- | --- |
| Analysis date: | Tue Apr 23 14:35:17 CST 2024 |
| BAM file: | /data1/fubq/project/2024/WHNGS0032-LY\_Dog\_RNAseq/analysis/2.Mapping/align/3-3-infected/3-3-infected\_Aligned.sortedByCoord.out.bam |
| Counting algorithm: | uniquely-mapped-reads |
| GTF file: | /data1/fubq/genome/dog/ensembl/Canis\_lupus\_familiaris.ROS\_Cfam\_1.0.109.gtf |
| Number of bases for 5'-3' bias computation: | 100 |
| Number of transcripts for 5'-3' bias computation: | 1,000 |
| Paired-end sequencing: | no |
| Protocol: | non-strand-specific |
| Sorting performed: | no |

## Summary

### Reads alignment

|  |  |
| --- | --- |
| Number of mapped reads: | 47,769,784 |
| Total number of alignments: | 49,526,318 |
| Number of secondary alignments: | 1,756,534 |
| Number of non-unique alignments: | 2,733,356 |
| Aligned to genes: | 37,551,321 |
| Ambiguous alignments: | 424,013 |
| No feature assigned: | 8,811,126 |
| Missing chromosome in annotation: | 6,502 |
| Not aligned: | 6,697,146 |
| Strand specificity estimation (fwd/rev): | 0.47 / 0.53 |

### Reads genomic origin

|  |  |
| --- | --- |
| Exonic: | 37,551,321 / 81% |
| Intronic: | 5,216,272 / 11.25% |
| Intergenic: | 3,594,854 / 7.75% |
| Intronic/intergenic overlapping exon: | 3,189,261 / 6.88% |

### Transcript coverage profile

|  |  |
| --- | --- |
| 5' bias: | 0.67 |
| 3' bias: | 0.31 |
| 5'-3' bias: | 1.61 |

### Junction analysis

|  |  |
| --- | --- |
| Reads at junctions: | 27,596,849 |
| ACCT | 5.4% |
| AGGT | 4.79% |
| AGGA | 3.87% |
| TCCT | 3.78% |
| AGCT | 2.96% |
| CCCT | 2.9% |
| ATCT | 2.8% |
| AGAT | 2.74% |
| GCCT | 2.37% |
| AGGC | 2.34% |
| AGGG | 2.12% |

## Reads Genomic Origin

## Coverage Profile Along Genes (Total)

## Coverage Profile Along Genes (Low)

## Coverage Profile Along Genes (High)

## Coverage Histogram (0-50X)

## Junction Analysis

### Contents

- Input data & parameters
- Summary
- Reads Genomic Origin
- Coverage Profile Along Genes (Total)
- Coverage Profile Along Genes (Low)
- Coverage Profile Along Genes (High)
- Coverage Histogram (0-50X)
- Junction Analysis

2024/04/23 14:35:19

Generated by QualiMap v.2.3.1-dev
