# Supplementary material for: Production, Passaging Stability, and Histological Analysis of Madin–Darby Canine Kidney Cells Cultured in a Low-Serum Medium
Source: Vaccines (Basel). 2024 Aug 30;12(9):991. doi: 10.3390/vaccines12090991 (PMC11435615; doi:10.3390/vaccines12090991)
Supplement: Supplementary file 1 [file vaccines-12-00991-s001.zip › Supplementary File S3/transcriptome/2.Mapping/qualimap/5-1-infected/qualimapReport.html]

Qualimap report: RNA Seq QC


Qualimap Report: RNA Seq QC

## Input data and parameters

### Input

|  |  |
| --- | --- |
| Analysis date: | Tue Apr 23 14:36:49 CST 2024 |
| BAM file: | /data1/fubq/project/2024/WHNGS0032-LY\_Dog\_RNAseq/analysis/2.Mapping/align/5-1-infected/5-1-infected\_Aligned.sortedByCoord.out.bam |
| Counting algorithm: | uniquely-mapped-reads |
| GTF file: | /data1/fubq/genome/dog/ensembl/Canis\_lupus\_familiaris.ROS\_Cfam\_1.0.109.gtf |
| Number of bases for 5'-3' bias computation: | 100 |
| Number of transcripts for 5'-3' bias computation: | 1,000 |
| Paired-end sequencing: | no |
| Protocol: | non-strand-specific |
| Sorting performed: | no |

## Summary

### Reads alignment

|  |  |
| --- | --- |
| Number of mapped reads: | 46,607,708 |
| Total number of alignments: | 48,199,059 |
| Number of secondary alignments: | 1,591,351 |
| Number of non-unique alignments: | 2,509,954 |
| Aligned to genes: | 36,278,502 |
| Ambiguous alignments: | 424,627 |
| No feature assigned: | 8,979,550 |
| Missing chromosome in annotation: | 6,426 |
| Not aligned: | 3,734,400 |
| Strand specificity estimation (fwd/rev): | 0.48 / 0.52 |

### Reads genomic origin

|  |  |
| --- | --- |
| Exonic: | 36,278,502 / 80.16% |
| Intronic: | 5,655,496 / 12.5% |
| Intergenic: | 3,324,054 / 7.34% |
| Intronic/intergenic overlapping exon: | 3,127,359 / 6.91% |

### Transcript coverage profile

|  |  |
| --- | --- |
| 5' bias: | 0.66 |
| 3' bias: | 0.31 |
| 5'-3' bias: | 1.65 |

### Junction analysis

|  |  |
| --- | --- |
| Reads at junctions: | 26,463,978 |
| ACCT | 5.27% |
| AGGT | 4.83% |
| AGGA | 3.91% |
| TCCT | 3.81% |
| AGCT | 3.01% |
| ATCT | 2.83% |
| AGAT | 2.78% |
| CCCT | 2.76% |
| GCCT | 2.4% |
| AGGC | 2.37% |
| AGGG | 2.11% |

## Reads Genomic Origin

## Coverage Profile Along Genes (Total)

## Coverage Profile Along Genes (Low)

## Coverage Profile Along Genes (High)

## Coverage Histogram (0-50X)

## Junction Analysis

### Contents

- Input data & parameters
- Summary
- Reads Genomic Origin
- Coverage Profile Along Genes (Total)
- Coverage Profile Along Genes (Low)
- Coverage Profile Along Genes (High)
- Coverage Histogram (0-50X)
- Junction Analysis

2024/04/23 14:36:50

Generated by QualiMap v.2.3.1-dev
