# Supplementary material for: Production, Passaging Stability, and Histological Analysis of Madin–Darby Canine Kidney Cells Cultured in a Low-Serum Medium
Source: Vaccines (Basel). 2024 Aug 30;12(9):991. doi: 10.3390/vaccines12090991 (PMC11435615; doi:10.3390/vaccines12090991)
Supplement: Supplementary file 1 [file vaccines-12-00991-s001.zip › Supplementary File S3/transcriptome/2.Mapping/qualimap/5-1-uninfected/qualimapReport.html]

Qualimap report: RNA Seq QC


Qualimap Report: RNA Seq QC

## Input data and parameters

### Input

|  |  |
| --- | --- |
| Analysis date: | Tue Apr 23 14:43:41 CST 2024 |
| BAM file: | /data1/fubq/project/2024/WHNGS0032-LY\_Dog\_RNAseq/analysis/2.Mapping/align/5-1-uninfected/5-1-uninfected\_Aligned.sortedByCoord.out.bam |
| Counting algorithm: | uniquely-mapped-reads |
| GTF file: | /data1/fubq/genome/dog/ensembl/Canis\_lupus\_familiaris.ROS\_Cfam\_1.0.109.gtf |
| Number of bases for 5'-3' bias computation: | 100 |
| Number of transcripts for 5'-3' bias computation: | 1,000 |
| Paired-end sequencing: | no |
| Protocol: | non-strand-specific |
| Sorting performed: | no |

## Summary

### Reads alignment

|  |  |
| --- | --- |
| Number of mapped reads: | 93,599,102 |
| Total number of alignments: | 97,990,044 |
| Number of secondary alignments: | 4,390,942 |
| Number of non-unique alignments: | 6,617,702 |
| Aligned to genes: | 74,607,581 |
| Ambiguous alignments: | 952,375 |
| No feature assigned: | 15,802,940 |
| Missing chromosome in annotation: | 9,446 |
| Not aligned: | 5,799,486 |
| Strand specificity estimation (fwd/rev): | 0.48 / 0.52 |

### Reads genomic origin

|  |  |
| --- | --- |
| Exonic: | 74,607,581 / 82.52% |
| Intronic: | 9,316,090 / 10.3% |
| Intergenic: | 6,486,850 / 7.17% |
| Intronic/intergenic overlapping exon: | 6,701,770 / 7.41% |

### Transcript coverage profile

|  |  |
| --- | --- |
| 5' bias: | 0.69 |
| 3' bias: | 0.35 |
| 5'-3' bias: | 1.51 |

### Junction analysis

|  |  |
| --- | --- |
| Reads at junctions: | 59,577,175 |
| ACCT | 5.46% |
| AGGT | 4.71% |
| AGGA | 3.97% |
| TCCT | 3.77% |
| AGCT | 3.01% |
| CCCT | 2.79% |
| GCCT | 2.62% |
| AGAT | 2.62% |
| ATCT | 2.61% |
| AGGC | 2.52% |
| AGGG | 2.25% |

## Reads Genomic Origin

## Coverage Profile Along Genes (Total)

## Coverage Profile Along Genes (Low)

## Coverage Profile Along Genes (High)

## Coverage Histogram (0-50X)

## Junction Analysis

### Contents

- Input data & parameters
- Summary
- Reads Genomic Origin
- Coverage Profile Along Genes (Total)
- Coverage Profile Along Genes (Low)
- Coverage Profile Along Genes (High)
- Coverage Histogram (0-50X)
- Junction Analysis

2024/04/23 14:43:43

Generated by QualiMap v.2.3.1-dev
