# Supplementary material for: Production, Passaging Stability, and Histological Analysis of Madin–Darby Canine Kidney Cells Cultured in a Low-Serum Medium
Source: Vaccines (Basel). 2024 Aug 30;12(9):991. doi: 10.3390/vaccines12090991 (PMC11435615; doi:10.3390/vaccines12090991)
Supplement: Supplementary file 1 [file vaccines-12-00991-s001.zip › Supplementary File S3/transcriptome/3.Expression/stats/ly_2d_pca.pdf]

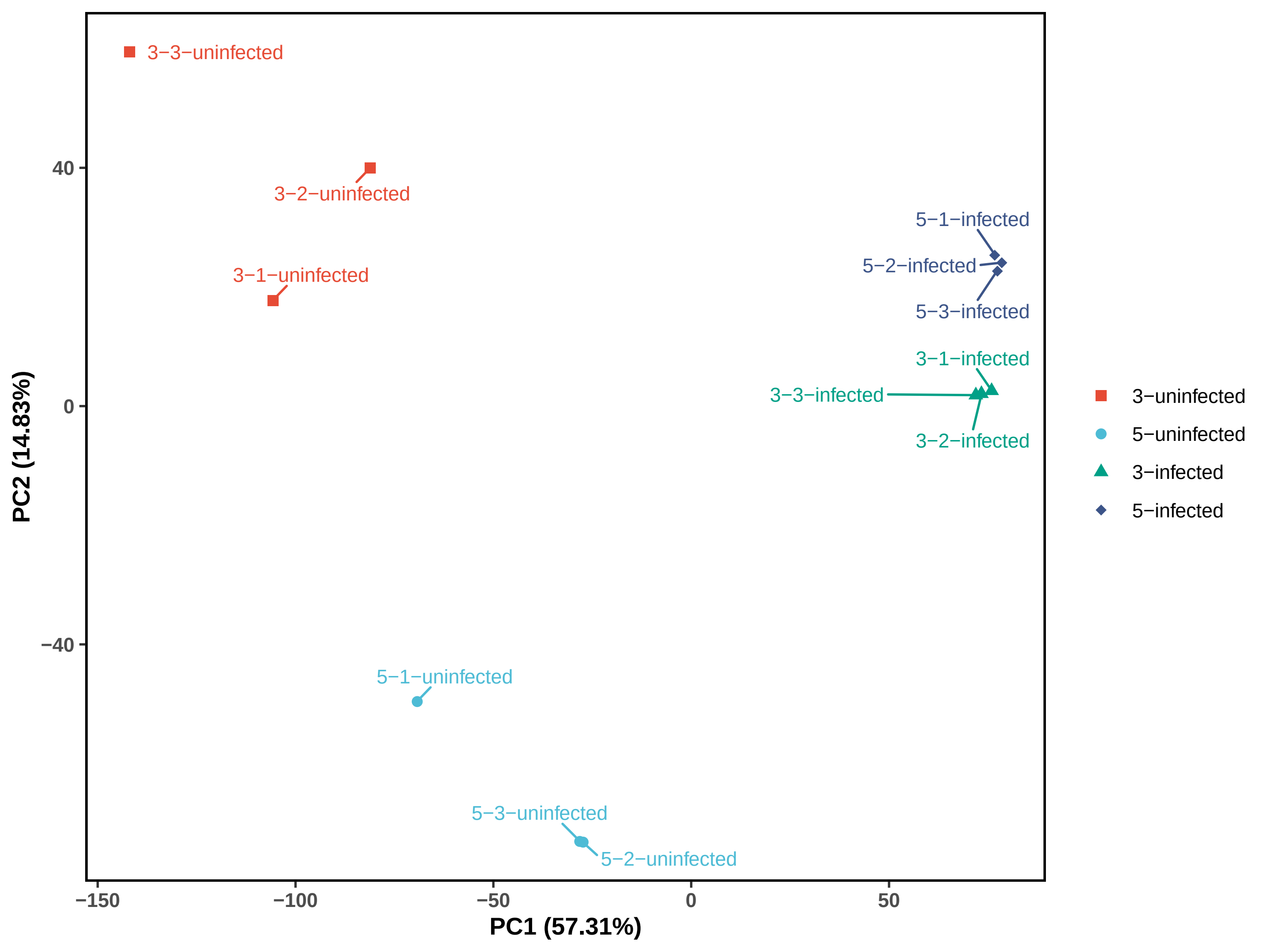
3−3−uninfected
3−2−uninfected
3−1−uninfected
5−1−infected
5−2−infected
5−3−infected
3−1−infected
3−3−infected
3−2−infected
5−1−uninfected
5−3−uninfected
5−2−uninfected
40
0
−40
−150
−100
−50
0
50
PC1 (57.31%)
PC2 (14.83%)
3−uninfected
5−uninfected
3−infected
5−infected
