# Supplementary material for: Production, Passaging Stability, and Histological Analysis of Madin–Darby Canine Kidney Cells Cultured in a Low-Serum Medium
Source: Vaccines (Basel). 2024 Aug 30;12(9):991. doi: 10.3390/vaccines12090991 (PMC11435615; doi:10.3390/vaccines12090991)
Supplement: Supplementary file 1 [file vaccines-12-00991-s001.zip › Supplementary File S3/transcriptome/3.Expression/stats/ly_correlation_heatmap.pdf]

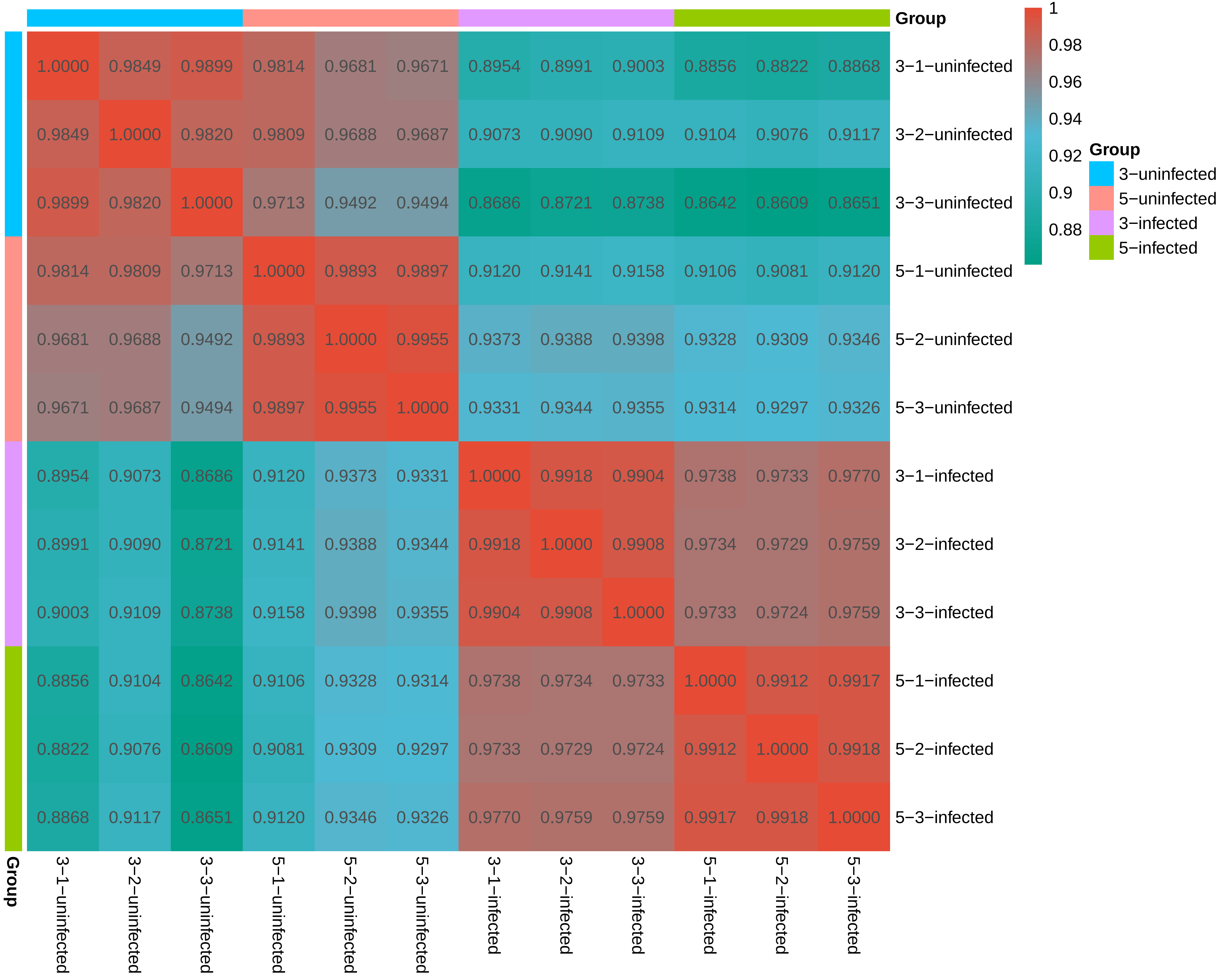
Group
1
0.98
0.96
0.94
0.92
0.9
0.88
Group
3−uninfected
5−uninfected
3−infected
5−infected
1.0000 0.9849 0.9899 0.9814 0.9681 0.9671 0.8954 0.8991 0.9003 0.8856 0.8822 0.8868 3−1−uninfected
0.9849 1.0000 0.9820 0.9809 0.9688 0.9687 0.9073 0.9090 0.9109 0.9104 0.9076 0.9117 3−2−uninfected
0.9899 0.9820 1.0000 0.9713 0.9492 0.9494 0.8686 0.8721 0.8738 0.8642 0.8609 0.8651 3−3−uninfected
0.9814 0.9809 0.9713 1.0000 0.9893 0.9897 0.9120 0.9141 0.9158 0.9106 0.9081 0.9120 5−1−uninfected
0.9681 0.9688 0.9492 0.9893 1.0000 0.9955 0.9373 0.9388 0.9398 0.9328 0.9309 0.9346 5−2−uninfected
0.9671 0.9687 0.9494 0.9897 0.9955 1.0000 0.9331 0.9344 0.9355 0.9314 0.9297 0.9326 5−3−uninfected
0.8954 0.9073 0.8686 0.9120 0.9373 0.9331 1.0000 0.9918 0.9904 0.9738 0.9733 0.9770 3−1−infected
0.8991 0.9090 0.8721 0.9141 0.9388 0.9344 0.9918 1.0000 0.9908 0.9734 0.9729 0.9759 3−2−infected
0.9003 0.9109 0.8738 0.9158 0.9398 0.9355 0.9904 0.9908 1.0000 0.9733 0.9724 0.9759 3−3−infected
0.8856 0.9104 0.8642 0.9106 0.9328 0.9314 0.9738 0.9734 0.9733 1.0000 0.9912 0.9917 5−1−infected
0.8822 0.9076 0.8609 0.9081 0.9309 0.9297 0.9733 0.9729 0.9724 0.9912 1.0000 0.9918 5−2−infected
0.8868 0.9117 0.8651 0.9120 0.9346 0.9326 0.9770 0.9759 0.9759 0.9917 0.9918 1.0000 5−3−infected
Group
3−1−uninfected
3−2−uninfected
3−3−uninfected
5−1−uninfected
5−2−uninfected
5−3−uninfected
3−1−infected
3−2−infected
3−3−infected
5−1−infected
5−2−infected
5−3−infected
