# Supplementary material for: Production, Passaging Stability, and Histological Analysis of Madin–Darby Canine Kidney Cells Cultured in a Low-Serum Medium
Source: Vaccines (Basel). 2024 Aug 30;12(9):991. doi: 10.3390/vaccines12090991 (PMC11435615; doi:10.3390/vaccines12090991)
Supplement: Supplementary file 1 [file vaccines-12-00991-s001.zip › Supplementary File S3/transcriptome/4.Difference/compare/3-infected_vs_5-infected/3-infected_vs_5-infected_diff_3d_pca.pdf]

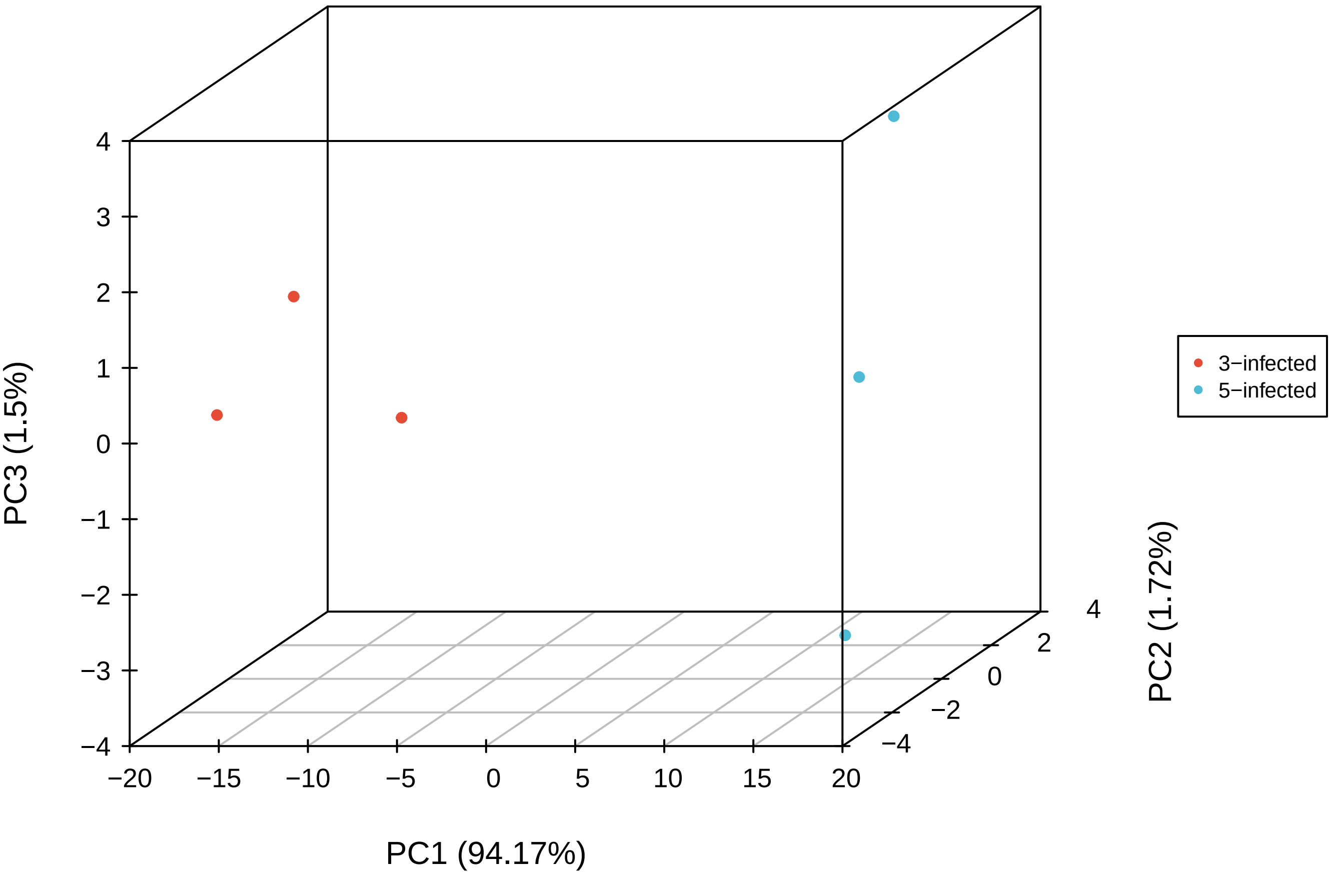
PC1 (94.17%)
PC2 (1.72%)
PC3 (1.5%)
3−infected
5−infected
−20
−15
−10
−5
0
5
10
15
20
−4
−2
0
2
4
−4
−3
−2
−1
0
1
2
3
4
