# Supplementary material for: Production, Passaging Stability, and Histological Analysis of Madin–Darby Canine Kidney Cells Cultured in a Low-Serum Medium
Source: Vaccines (Basel). 2024 Aug 30;12(9):991. doi: 10.3390/vaccines12090991 (PMC11435615; doi:10.3390/vaccines12090991)
Supplement: Supplementary file 1 [file vaccines-12-00991-s001.zip › Supplementary File S3/transcriptome/4.Difference/compare/3-infected_vs_5-infected/3-infected_vs_5-infected_gene_3d_pca.pdf]

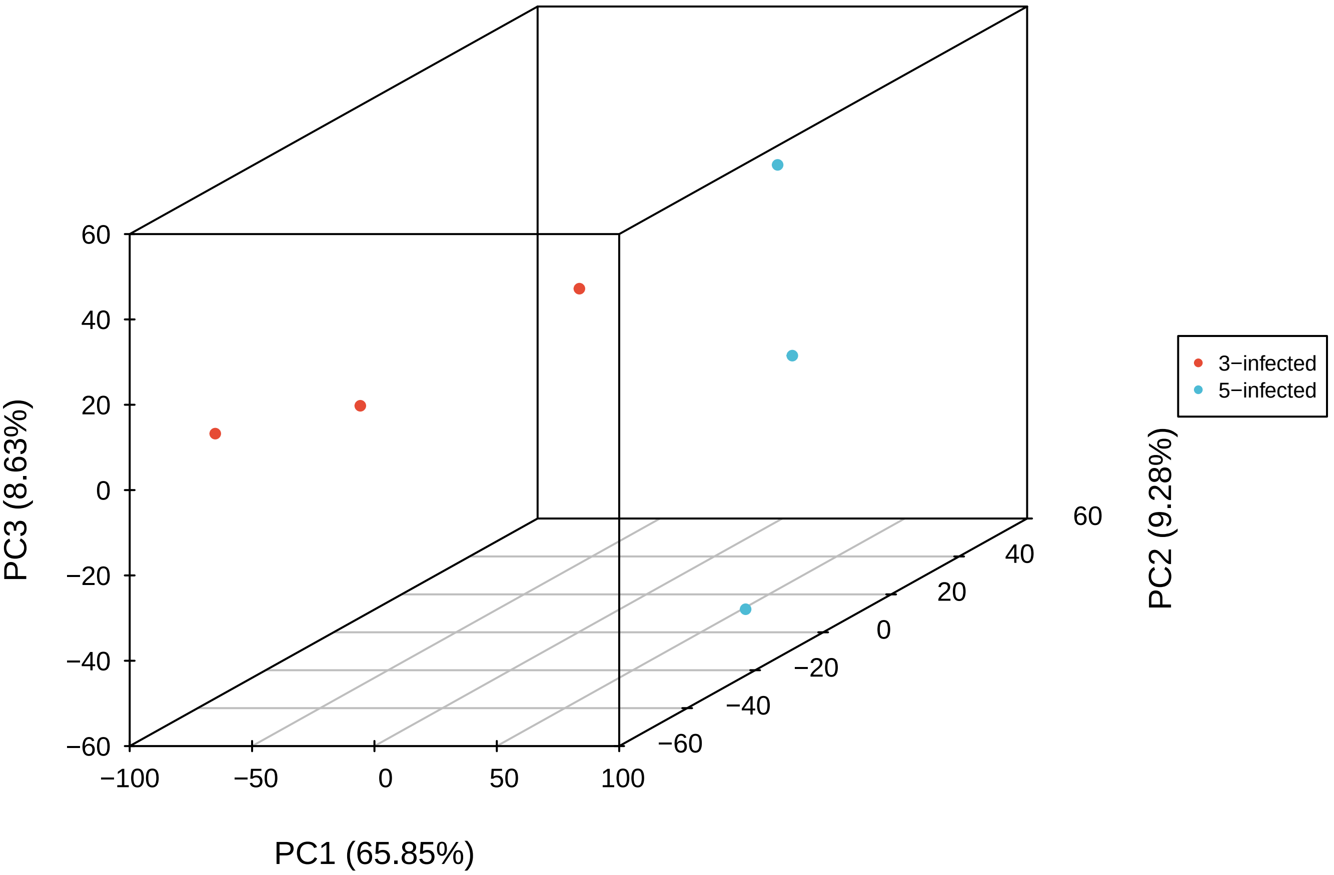

60
40
20
0
−20
−40
−60
PC3 (8.63%)
−100
−50
0
50
100
PC1 (65.85%)
60
40
20
0
−20
−40
−60
PC2 (9.28%)
3−infected
5−infected
