# Supplementary material for: Production, Passaging Stability, and Histological Analysis of Madin–Darby Canine Kidney Cells Cultured in a Low-Serum Medium
Source: Vaccines (Basel). 2024 Aug 30;12(9):991. doi: 10.3390/vaccines12090991 (PMC11435615; doi:10.3390/vaccines12090991)
Supplement: Supplementary file 1 [file vaccines-12-00991-s001.zip › Supplementary File S3/transcriptome/4.Difference/compare/3-infected_vs_5-infected/3-infected_vs_5-infected_gene_cluster_heatmap.pdf]

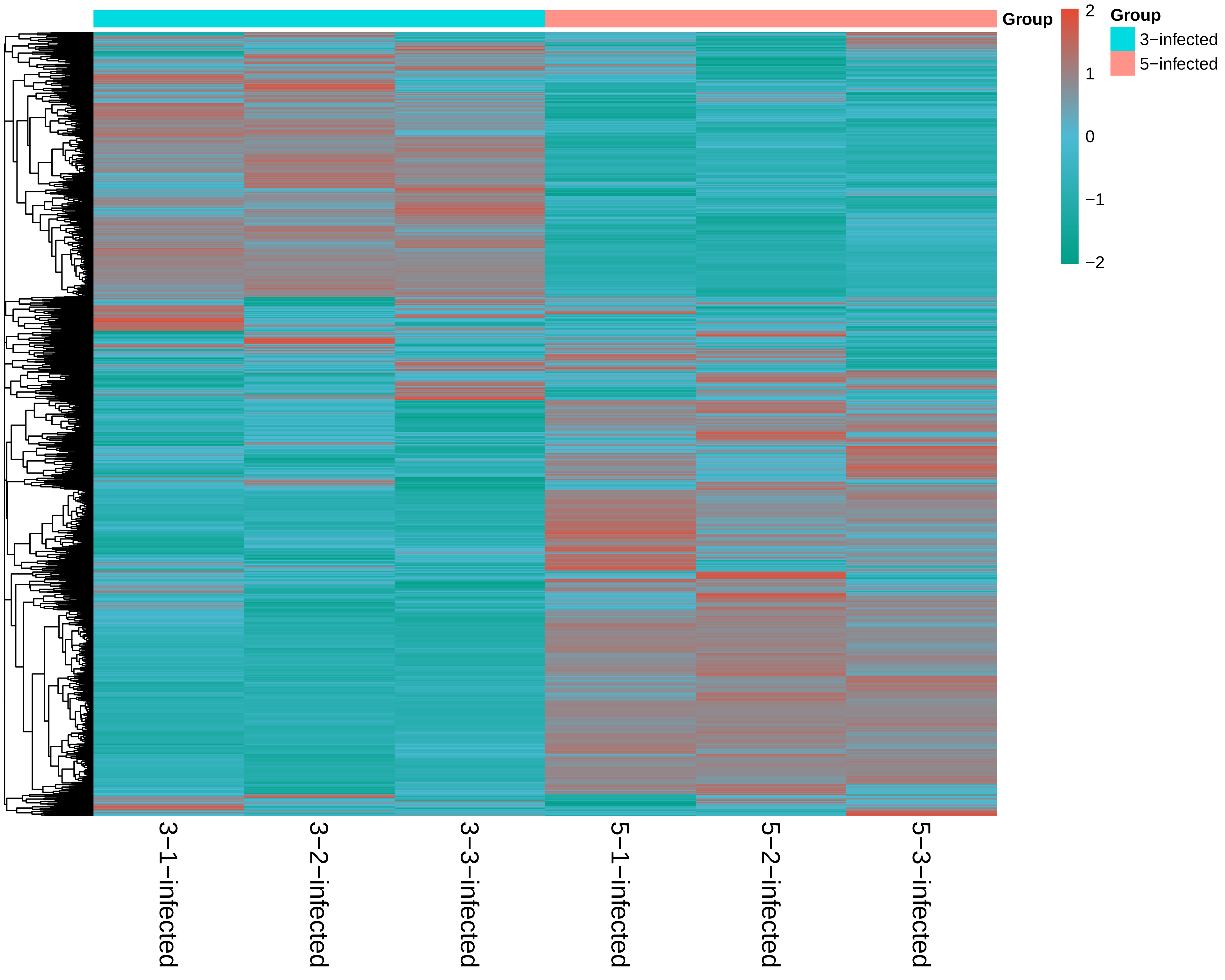

Group
2
1
0
−1
−2
Group
3−infected
5−infected
3−1−infected
3−2−infected
3−3−infected
5−1−infected
5−2−infected
5−3−infected
