# Supplementary material for: Production, Passaging Stability, and Histological Analysis of Madin–Darby Canine Kidney Cells Cultured in a Low-Serum Medium
Source: Vaccines (Basel). 2024 Aug 30;12(9):991. doi: 10.3390/vaccines12090991 (PMC11435615; doi:10.3390/vaccines12090991)
Supplement: Supplementary file 1 [file vaccines-12-00991-s001.zip › Supplementary File S3/transcriptome/4.Difference/compare/3-infected_vs_5-infected/3-infected_vs_5-infected_gene_volcano.pdf]

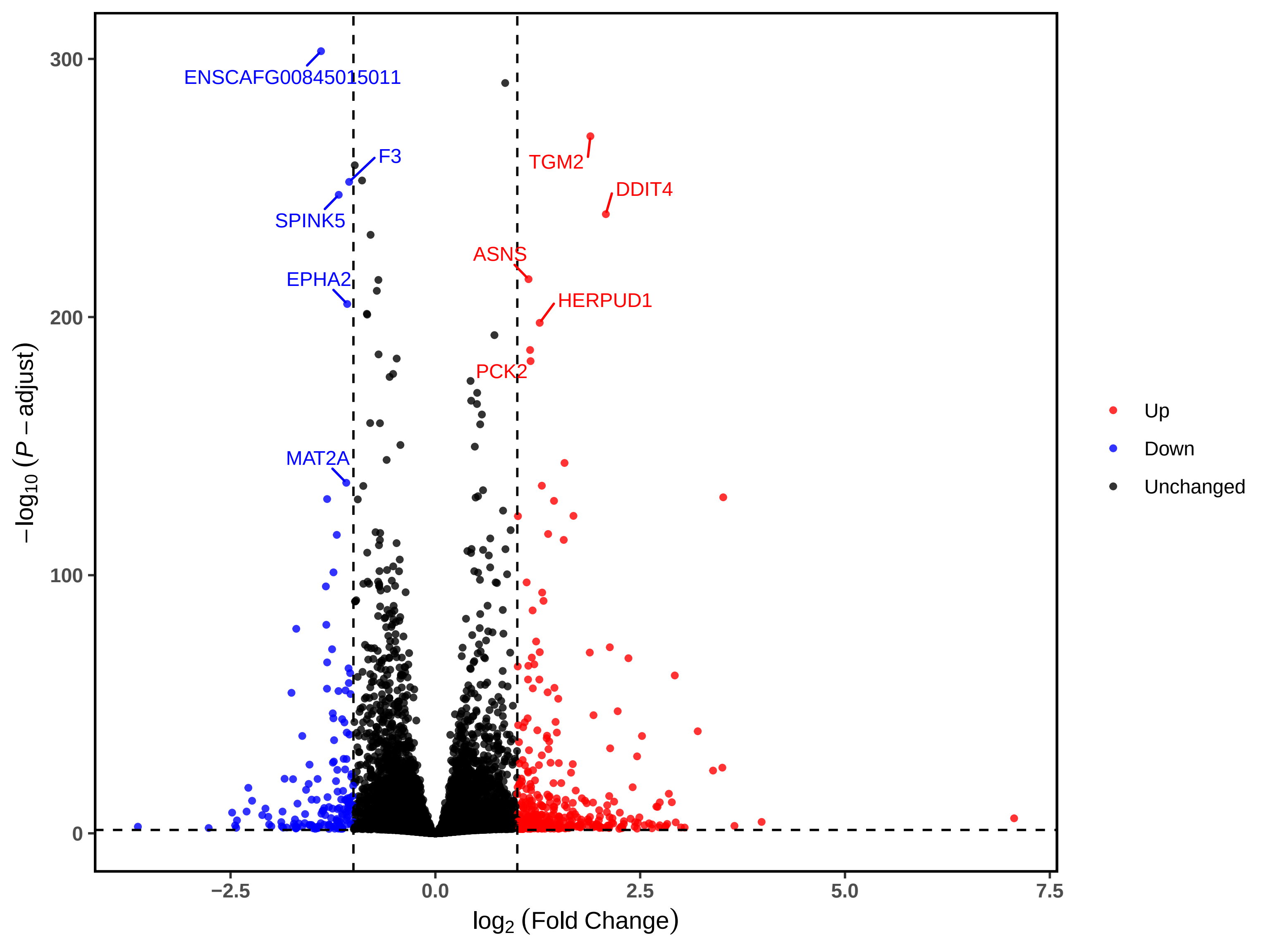
ENSCAFG00845015011
F3
SPINK5
EPHA2
MAT2A
TGM2
DDIT4
ASNS
HERPUD1
PCK2
300
200
100
0
−2.5
0.0
2.5
5.0
7.5
$-\log_{10}(P-\text{adjust})$
$\log_2(\text{Fold Change})$
Up
Down
Unchanged
