# Supplementary material for: Production, Passaging Stability, and Histological Analysis of Madin–Darby Canine Kidney Cells Cultured in a Low-Serum Medium
Source: Vaccines (Basel). 2024 Aug 30;12(9):991. doi: 10.3390/vaccines12090991 (PMC11435615; doi:10.3390/vaccines12090991)
Supplement: Supplementary file 1 [file vaccines-12-00991-s001.zip › Supplementary File S3/transcriptome/4.Difference/compare/3-infected_vs_5-infected/3-infected_vs_5-infected_top50_cluster_heatmap.pdf]

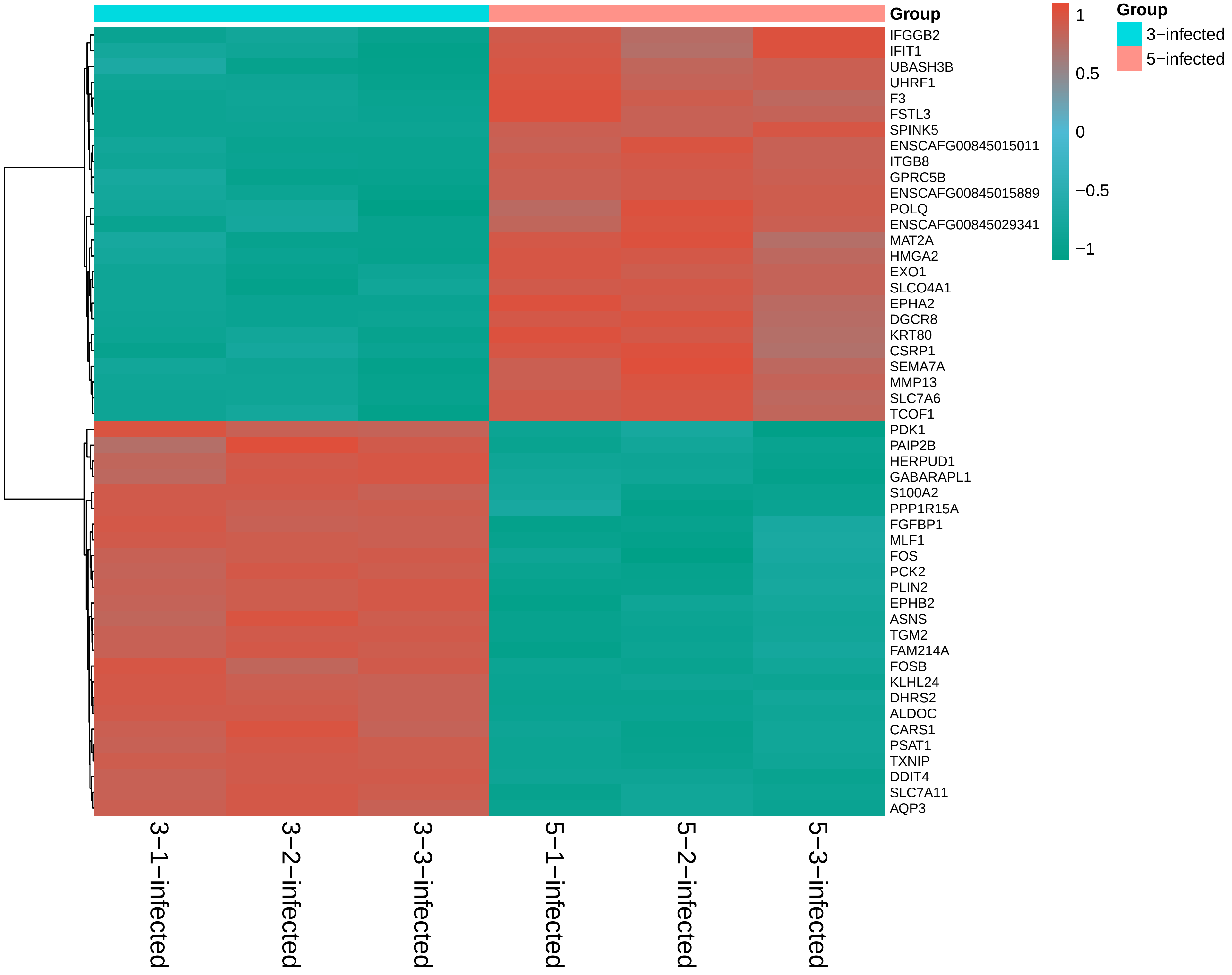
Group
3−infected
5−infected
1
0.5
0
−0.5
−1
IFGGB2
IFIT1
UBASH3B
UHRF1
F3
FSTL3
SPINK5
ENSCAFG00845015011
ITGB8
GPRC5B
ENSCAFG00845015889
POLQ
ENSCAFG00845029341
MAT2A
HMGA2
EXO1
SLCO4A1
EPHA2
DGCR8
KRT80
CSRP1
SEMA7A
MMP13
SLC7A6
TCOF1
PDK1
PAIP2B
HERPUD1
GABARAPL1
S100A2
PPP1R15A
FGFBP1
MLF1
FOS
PCK2
PLIN2
EPHB2
ASNS
TGM2
FAM214A
FOSB
KLHL24
DHRS2
ALDOC
CARS1
PSAT1
TXNIP
DDIT4
SLC7A11
AQP3
3−1−infected
3−2−infected
3−3−infected
5−1−infected
5−2−infected
5−3−infected
