# Supplementary material for: Production, Passaging Stability, and Histological Analysis of Madin–Darby Canine Kidney Cells Cultured in a Low-Serum Medium
Source: Vaccines (Basel). 2024 Aug 30;12(9):991. doi: 10.3390/vaccines12090991 (PMC11435615; doi:10.3390/vaccines12090991)
Supplement: Supplementary file 1 [file vaccines-12-00991-s001.zip › Supplementary File S3/transcriptome/4.Difference/compare/3-uninfected_vs_5-uninfected/3-uninfected_vs_5-uninfected_diff_3d_pca.pdf]

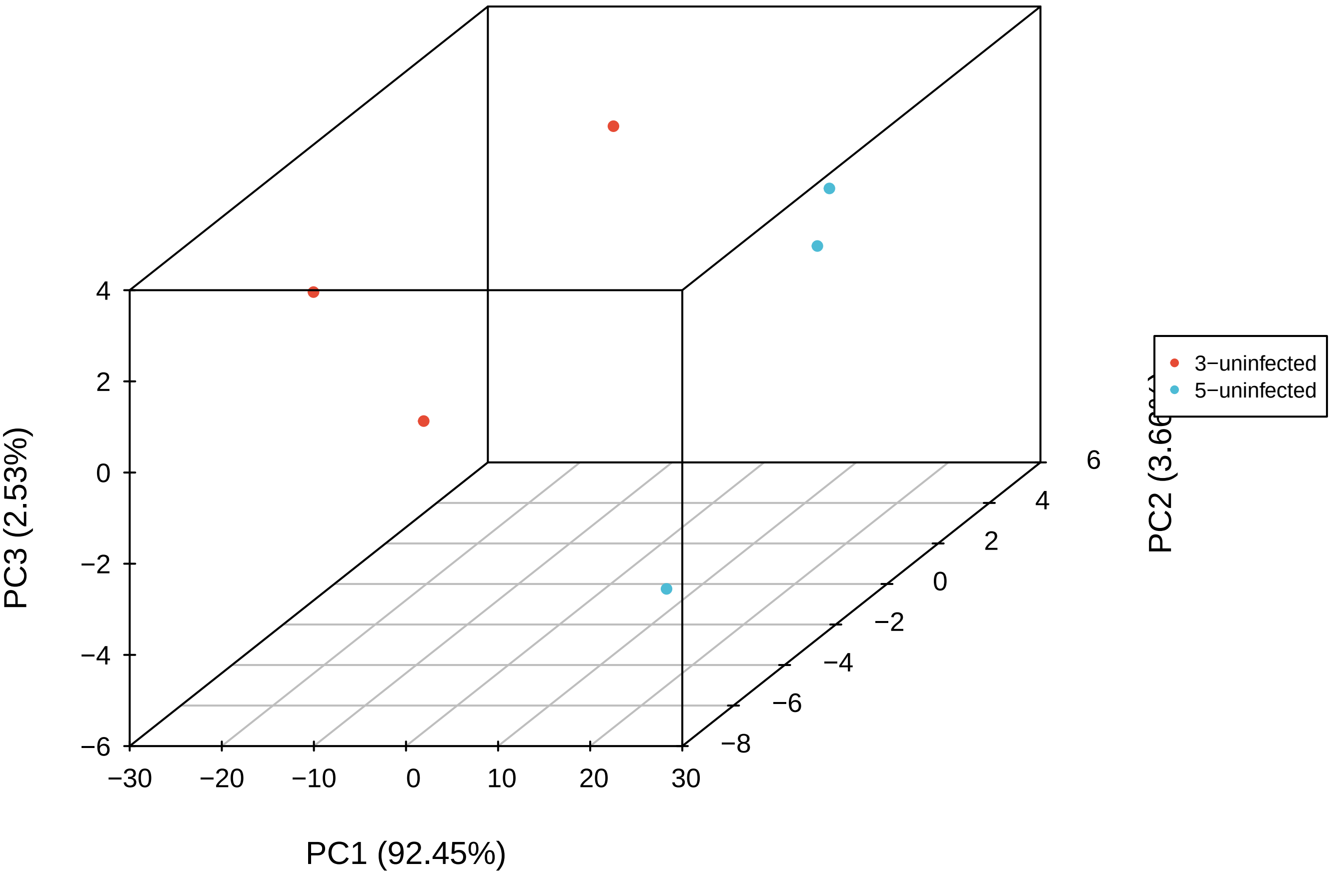

PC1 (92.45%)
PC3 (2.53%)
PC2
−30
−20
−10
0
10
20
30
−6
−4
−2
0
2
4
−8
−6
−4
−2
0
2
4
6
3−uninfected
5−uninfected
