# Supplementary material for: Production, Passaging Stability, and Histological Analysis of Madin–Darby Canine Kidney Cells Cultured in a Low-Serum Medium
Source: Vaccines (Basel). 2024 Aug 30;12(9):991. doi: 10.3390/vaccines12090991 (PMC11435615; doi:10.3390/vaccines12090991)
Supplement: Supplementary file 1 [file vaccines-12-00991-s001.zip › Supplementary File S3/transcriptome/4.Difference/compare/3-uninfected_vs_5-uninfected/3-uninfected_vs_5-uninfected_diff_cluster_heatmap.pdf]

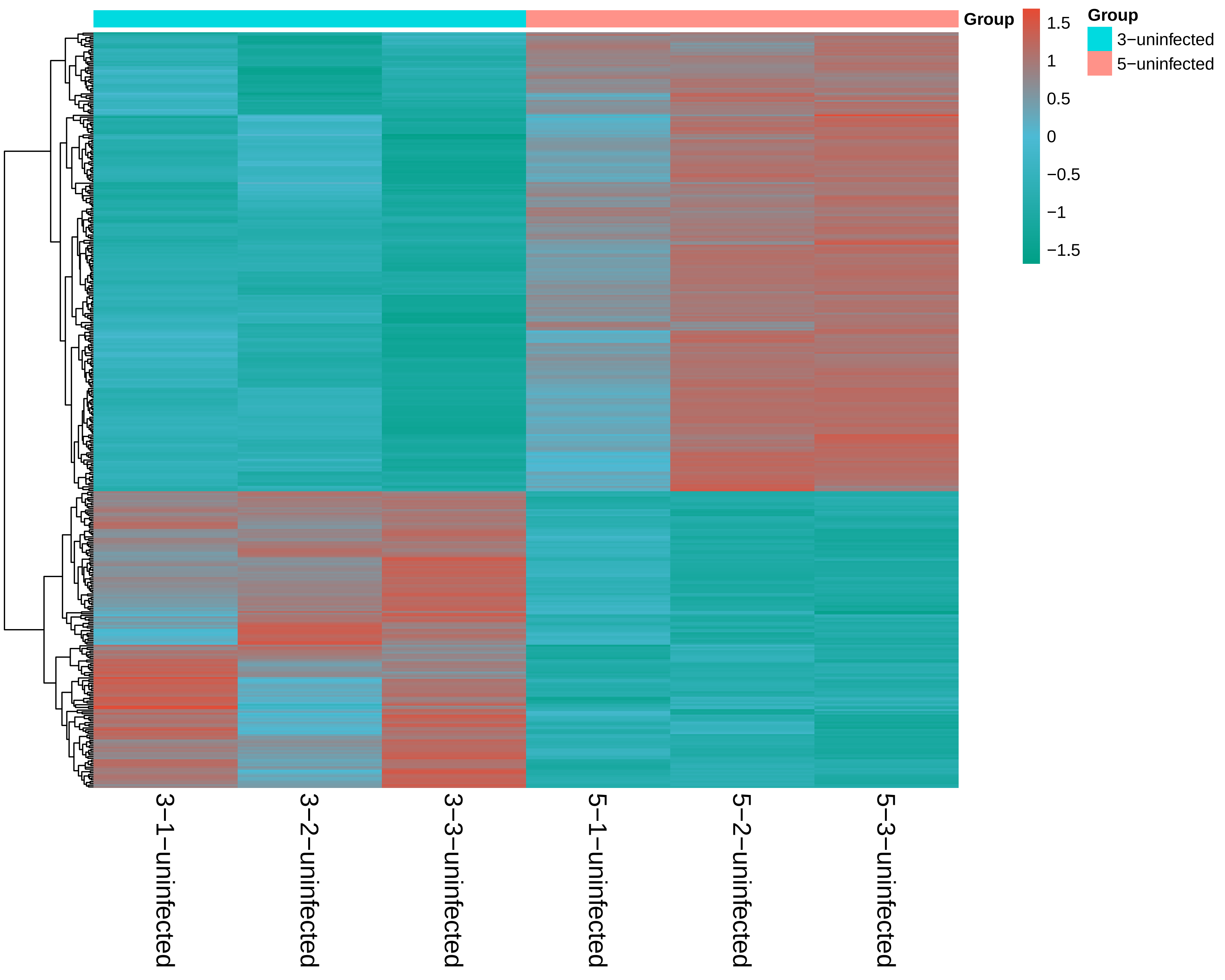
Group
1.5
1
0.5
0
−0.5
−1
−1.5
Group
3−uninfected
5−uninfected
3−1−uninfected
3−2−uninfected
3−3−uninfected
5−1−uninfected
5−2−uninfected
5−3−uninfected
