# Supplementary material for: Production, Passaging Stability, and Histological Analysis of Madin–Darby Canine Kidney Cells Cultured in a Low-Serum Medium
Source: Vaccines (Basel). 2024 Aug 30;12(9):991. doi: 10.3390/vaccines12090991 (PMC11435615; doi:10.3390/vaccines12090991)
Supplement: Supplementary file 1 [file vaccines-12-00991-s001.zip › Supplementary File S3/transcriptome/4.Difference/compare/3-uninfected_vs_5-uninfected/3-uninfected_vs_5-uninfected_gene_3d_pca.pdf]

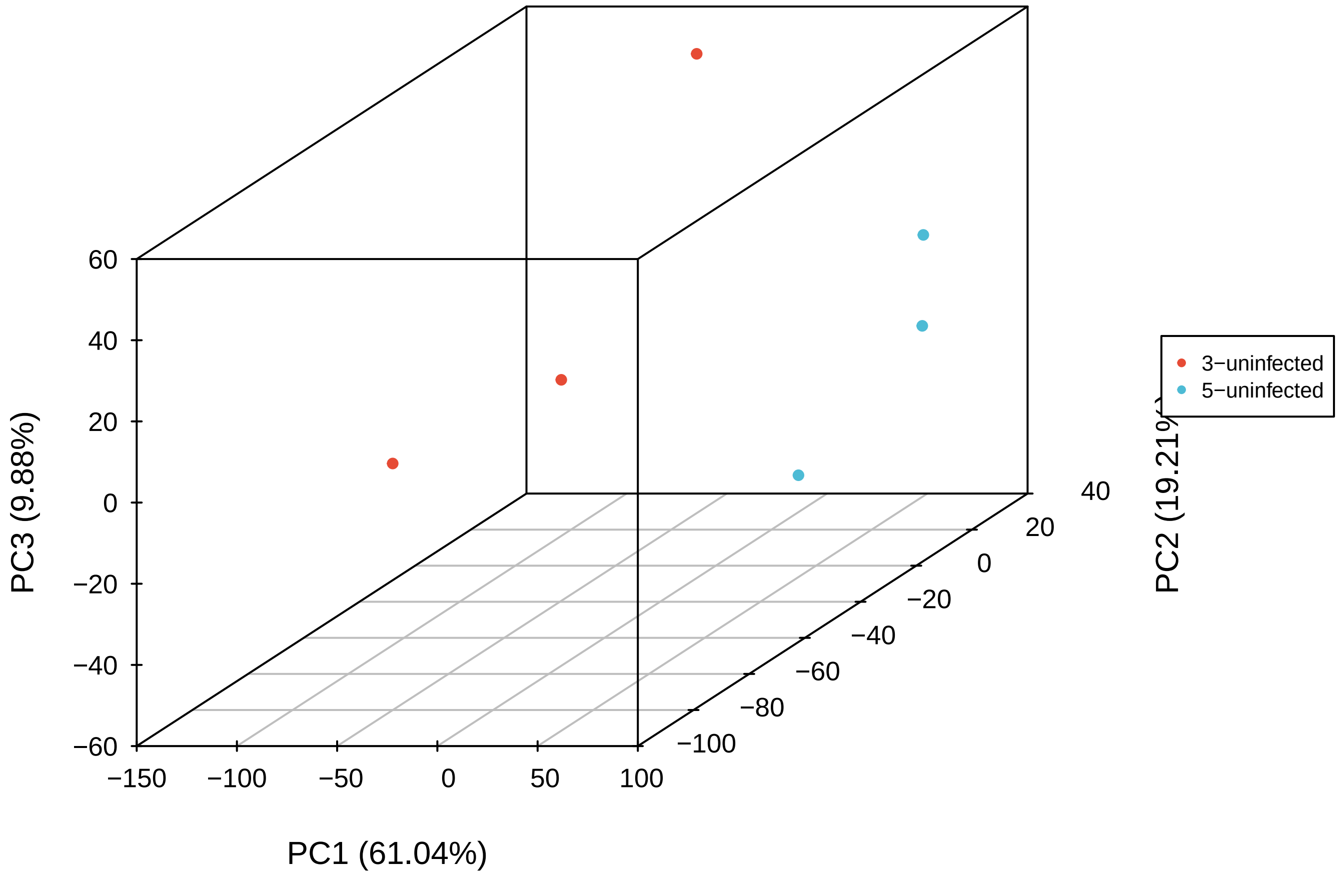
PC1 (61.04%)
PC2 (19.21%)
PC3 (9.88%)
−150
−100
−50
0
50
100
−100
−80
−60
−40
−20
0
20
40
−60
−40
−20
0
20
40
60
3−uninfected
5−uninfected
