# Supplementary material for: Production, Passaging Stability, and Histological Analysis of Madin–Darby Canine Kidney Cells Cultured in a Low-Serum Medium
Source: Vaccines (Basel). 2024 Aug 30;12(9):991. doi: 10.3390/vaccines12090991 (PMC11435615; doi:10.3390/vaccines12090991)
Supplement: Supplementary file 1 [file vaccines-12-00991-s001.zip › Supplementary File S3/transcriptome/4.Difference/compare/3-uninfected_vs_5-uninfected/3-uninfected_vs_5-uninfected_gene_volcano.pdf]

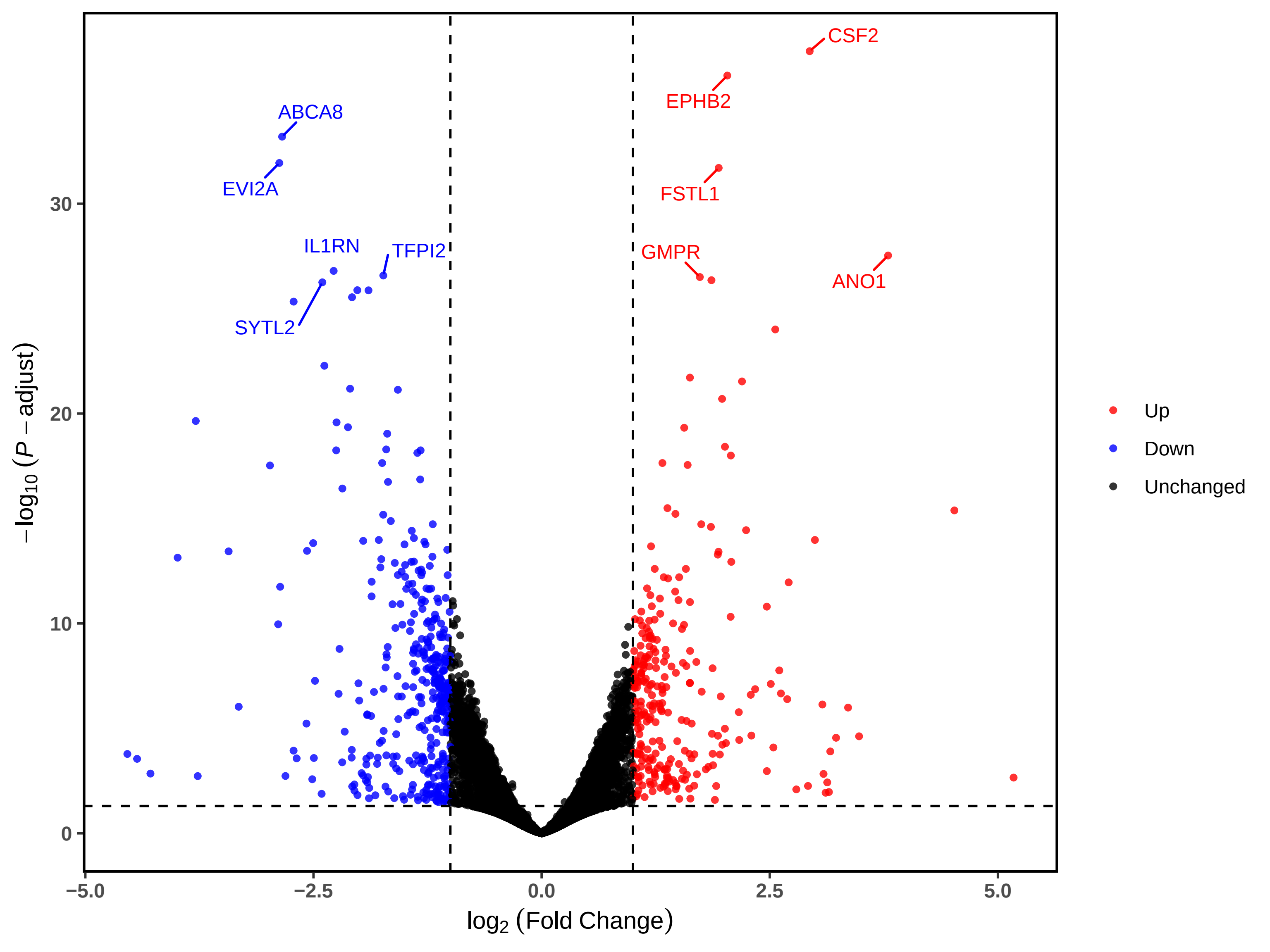

CSF2
EPHB2
ABCA8
EVI2A
FSTL1
IL1RN
TFPI2
GMPR
ANO1
SYTL2
30
20
10
0
−5.0
−2.5
0.0
2.5
5.0
$-\log_{10}(P-\text{adjust})$
$\log_2(\text{Fold Change})$
Up
Down
Unchanged
