# Supplementary material for: Production, Passaging Stability, and Histological Analysis of Madin–Darby Canine Kidney Cells Cultured in a Low-Serum Medium
Source: Vaccines (Basel). 2024 Aug 30;12(9):991. doi: 10.3390/vaccines12090991 (PMC11435615; doi:10.3390/vaccines12090991)
Supplement: Supplementary file 1 [file vaccines-12-00991-s001.zip › Supplementary File S3/transcriptome/4.Difference/compare/3-uninfected_vs_5-uninfected/3-uninfected_vs_5-uninfected_top50_cluster_heatmap.pdf]

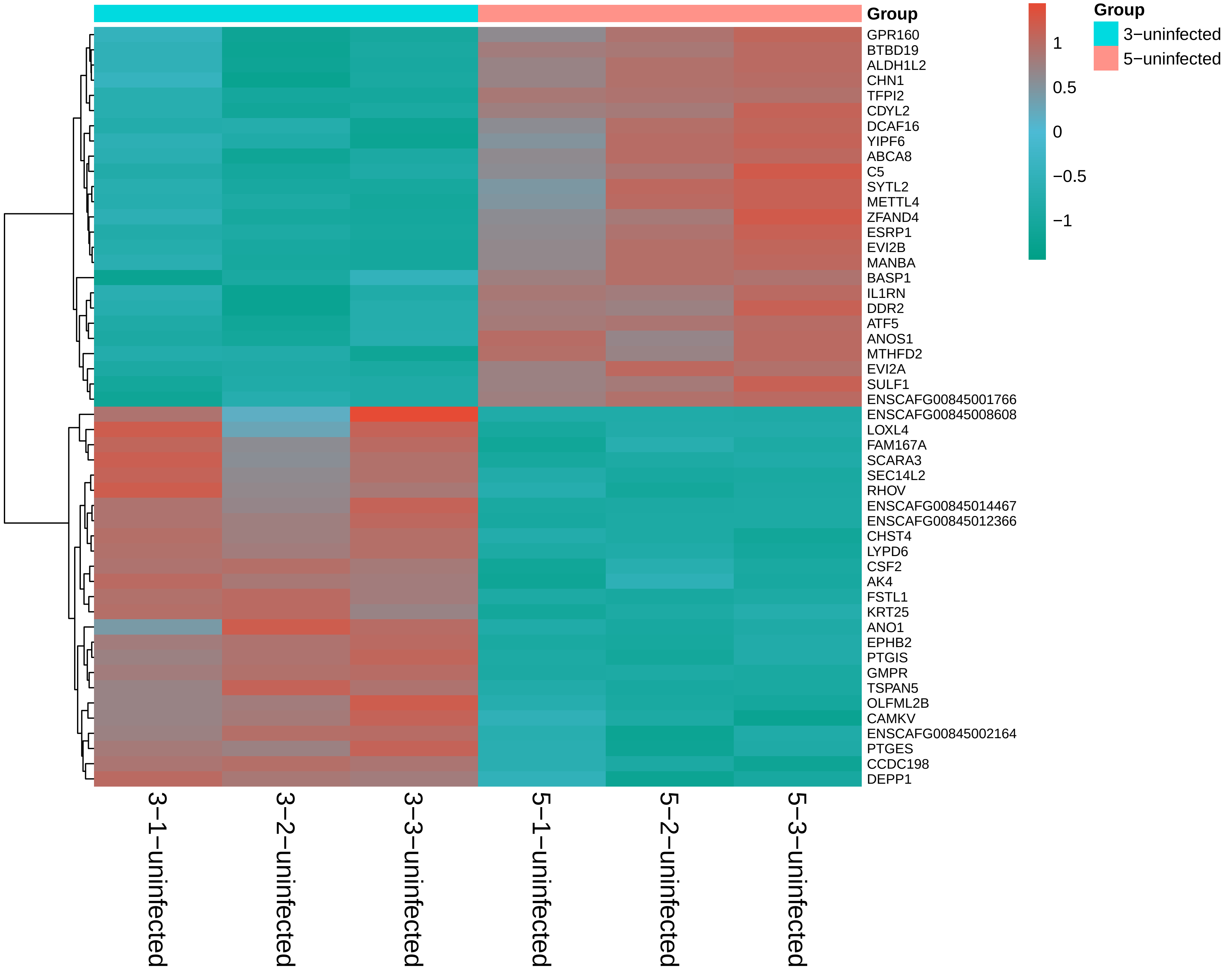

Group
1
0.5
0
−0.5
−1
Group
3−uninfected
5−uninfected
GPR160
BTBD19
ALDH1L2
CHN1
TFPI2
CDYL2
DCAF16
YIPF6
ABCA8
C5
SYTL2
METTL4
ZFAND4
ESRP1
EVI2B
MANBA
BASP1
IL1RN
DDR2
ATF5
ANOS1
MTHFD2
EVI2A
SULF1
ENSCAFG00845001766
ENSCAFG00845008608
LOXL4
FAM167A
SCARA3
SEC14L2
RHOV
ENSCAFG00845014467
ENSCAFG00845012366
CHST4
LYPD6
CSF2
AK4
FSTL1
KRT25
ANO1
EPHB2
PTGIS
GMPR
TSPAN5
OLFML2B
CAMKV
ENSCAFG00845002164
PTGES
CCDC198
DEPP1
3−1−uninfected
3−2−uninfected
3−3−uninfected
5−1−uninfected
5−2−uninfected
5−3−uninfected
