# Supplementary material for: Production, Passaging Stability, and Histological Analysis of Madin–Darby Canine Kidney Cells Cultured in a Low-Serum Medium
Source: Vaccines (Basel). 2024 Aug 30;12(9):991. doi: 10.3390/vaccines12090991 (PMC11435615; doi:10.3390/vaccines12090991)
Supplement: Supplementary file 1 [file vaccines-12-00991-s001.zip › Supplementary File S3/transcriptome/4.Difference/stats/ly_diff_venn.pdf]

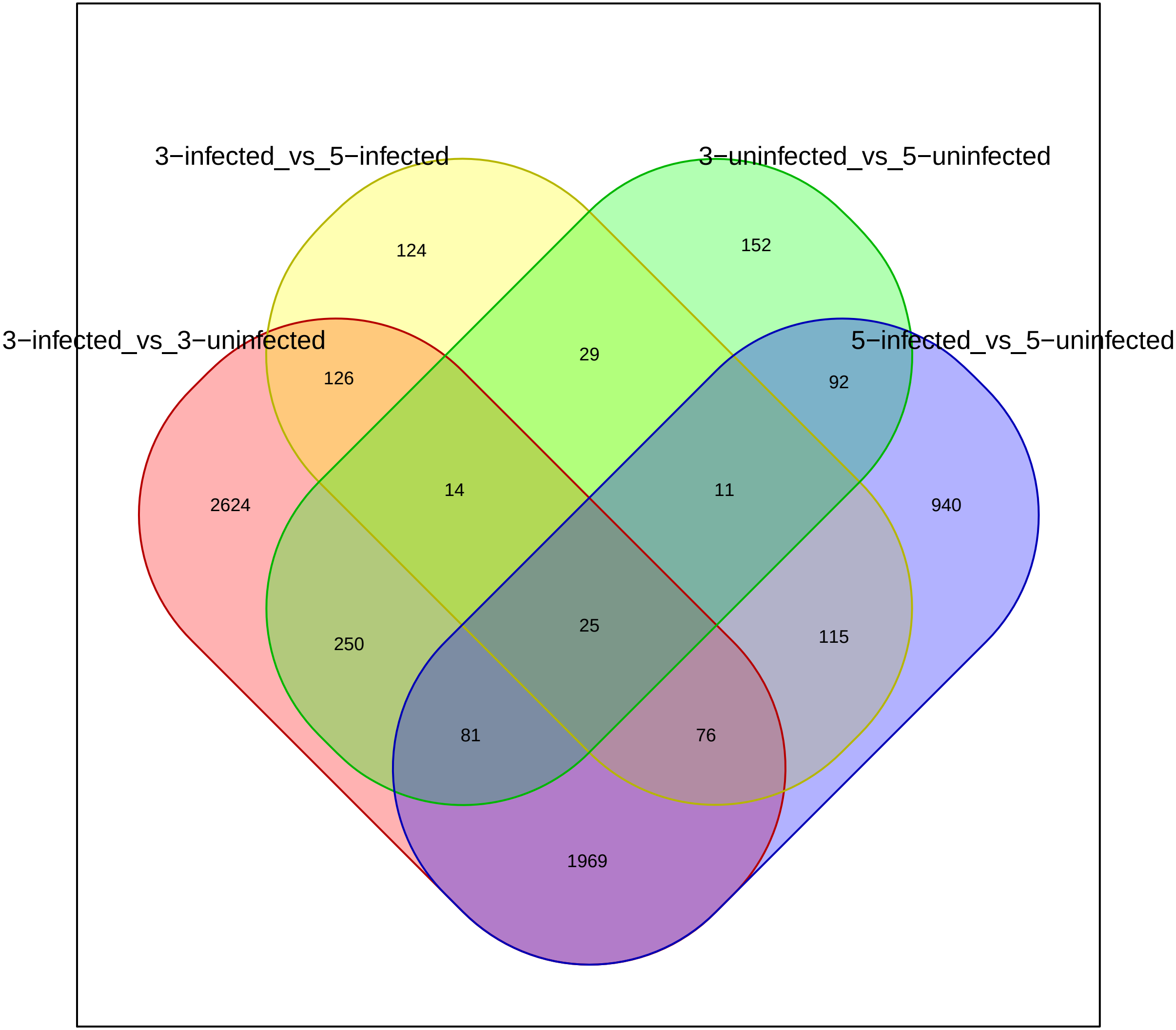
3−infected_vs_5−infected
3−uninfected_vs_5−uninfected
3−infected_vs_3−uninfected
5−infected_vs_5−uninfected
124
152
29
126
92
2624
14
11
940
25
250
115
81
76
1969
