# Supplementary material for: Production, Passaging Stability, and Histological Analysis of Madin–Darby Canine Kidney Cells Cultured in a Low-Serum Medium
Source: Vaccines (Basel). 2024 Aug 30;12(9):991. doi: 10.3390/vaccines12090991 (PMC11435615; doi:10.3390/vaccines12090991)
Supplement: Supplementary file 1 [file vaccines-12-00991-s001.zip › Supplementary File S3/transcriptome/5.Enrichment/gsea/3-infected_vs_5-infected/3-infected_vs_5-infected_GO_ALL_GSEA_dotplot.pdf]

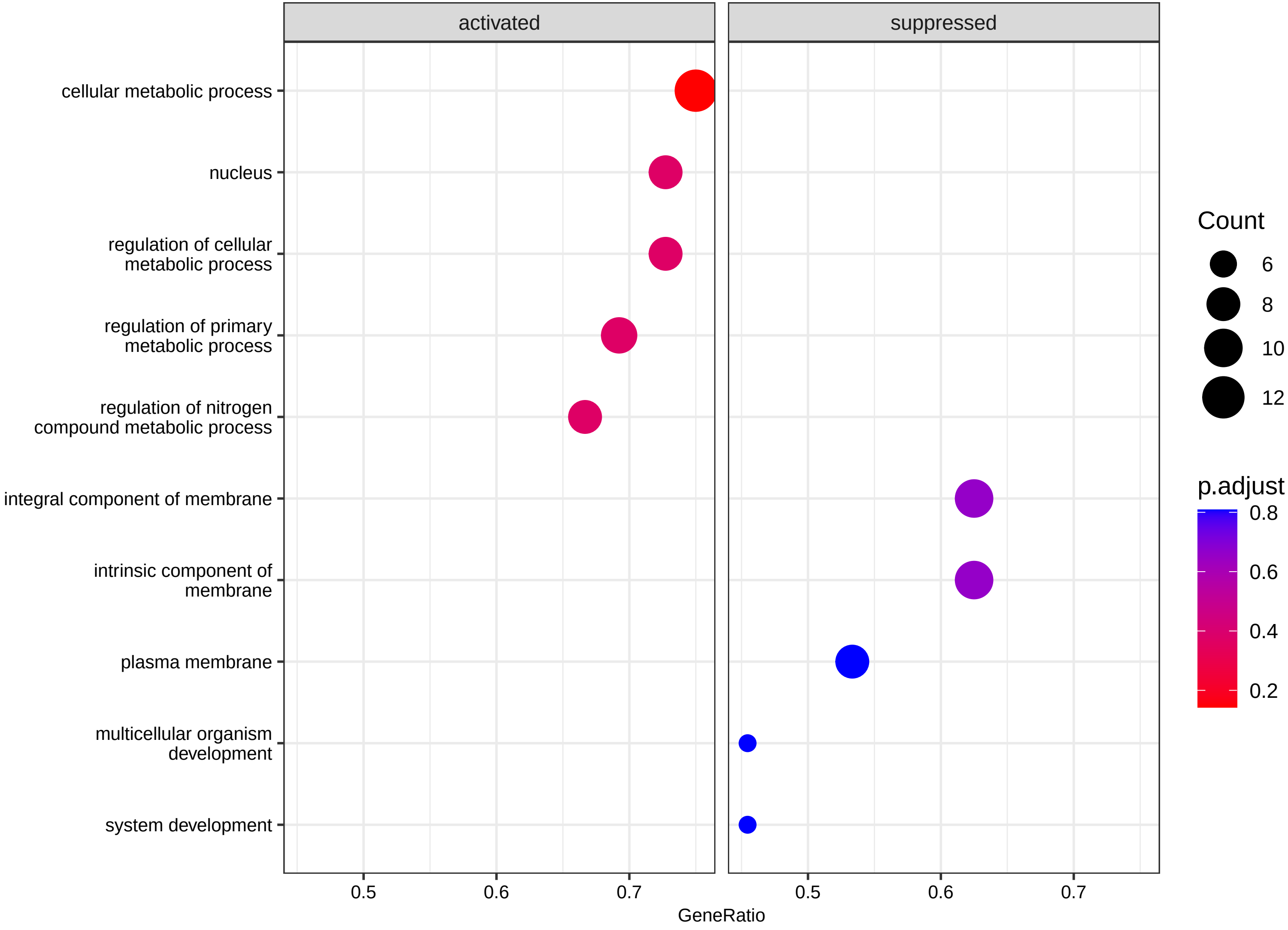
activated
suppressed
cellular metabolic process
nucleus
regulation of cellular metabolic process
regulation of primary metabolic process
regulation of nitrogen compound metabolic process
integral component of membrane
intrinsic component of membrane
plasma membrane
multicellular organism development
system development
0.5
0.6
0.7
0.5
0.6
0.7
GeneRatio
Count
6
8
10
12
p.adjust
0.8
0.6
0.4
0.2
