# Supplementary material for: Production, Passaging Stability, and Histological Analysis of Madin–Darby Canine Kidney Cells Cultured in a Low-Serum Medium
Source: Vaccines (Basel). 2024 Aug 30;12(9):991. doi: 10.3390/vaccines12090991 (PMC11435615; doi:10.3390/vaccines12090991)
Supplement: Supplementary file 1 [file vaccines-12-00991-s001.zip › Supplementary File S3/transcriptome/5.Enrichment/gsea/3-infected_vs_5-infected/3-infected_vs_5-infected_GO_ALL_GSEA_gseaplot.pdf]

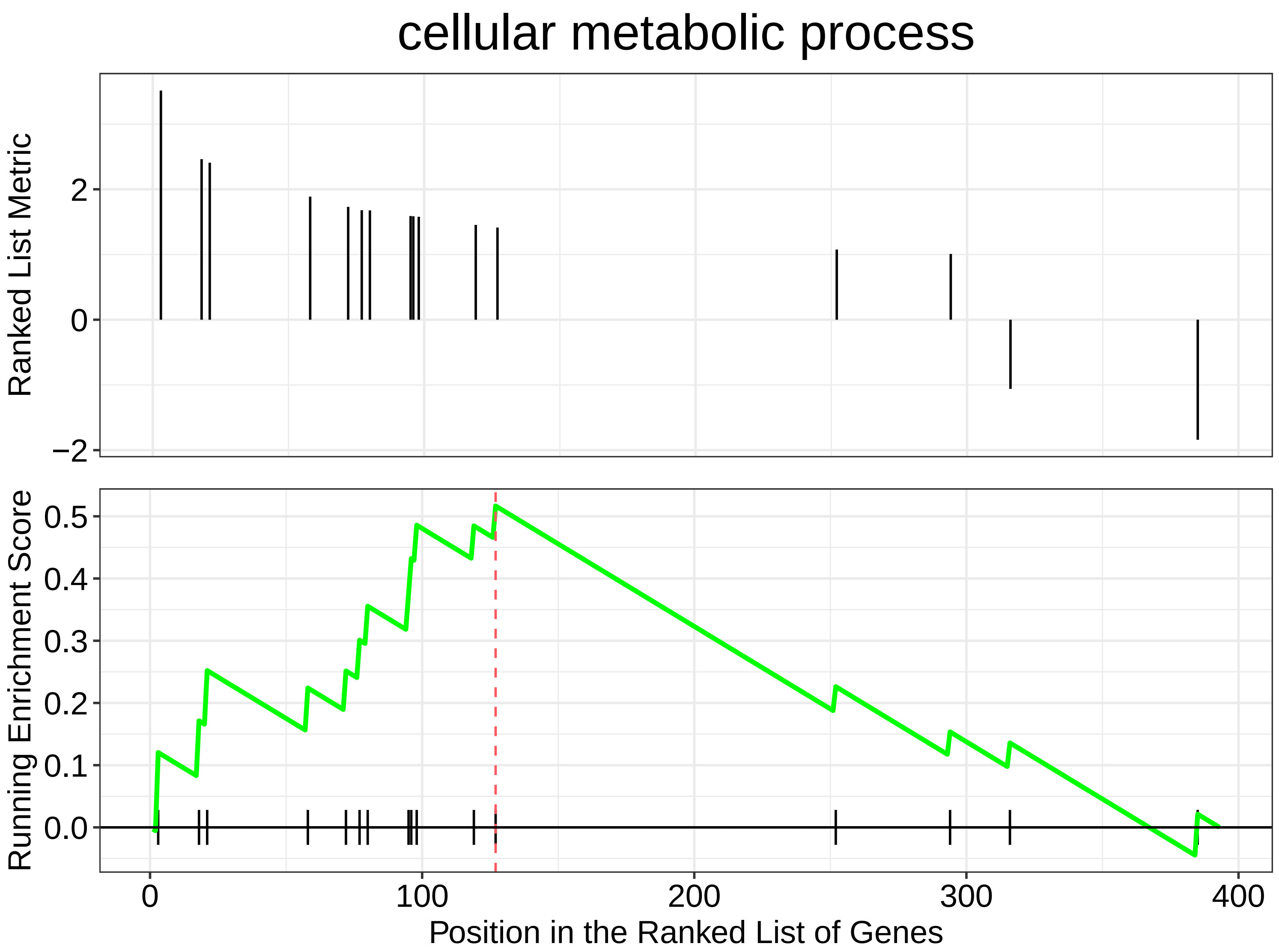
cellular metabolic process
Ranked List Metric
2
0
−2
Running Enrichment Score
0.5
0.4
0.3
0.2
0.1
0.0
0
100
200
300
400
Position in the Ranked List of Genes
