# Supplementary material for: Production, Passaging Stability, and Histological Analysis of Madin–Darby Canine Kidney Cells Cultured in a Low-Serum Medium
Source: Vaccines (Basel). 2024 Aug 30;12(9):991. doi: 10.3390/vaccines12090991 (PMC11435615; doi:10.3390/vaccines12090991)
Supplement: Supplementary file 1 [file vaccines-12-00991-s001.zip › Supplementary File S3/transcriptome/5.Enrichment/gsea/3-infected_vs_5-infected/3-infected_vs_5-infected_GO_ALL_GSEA_ridgeplot.pdf]

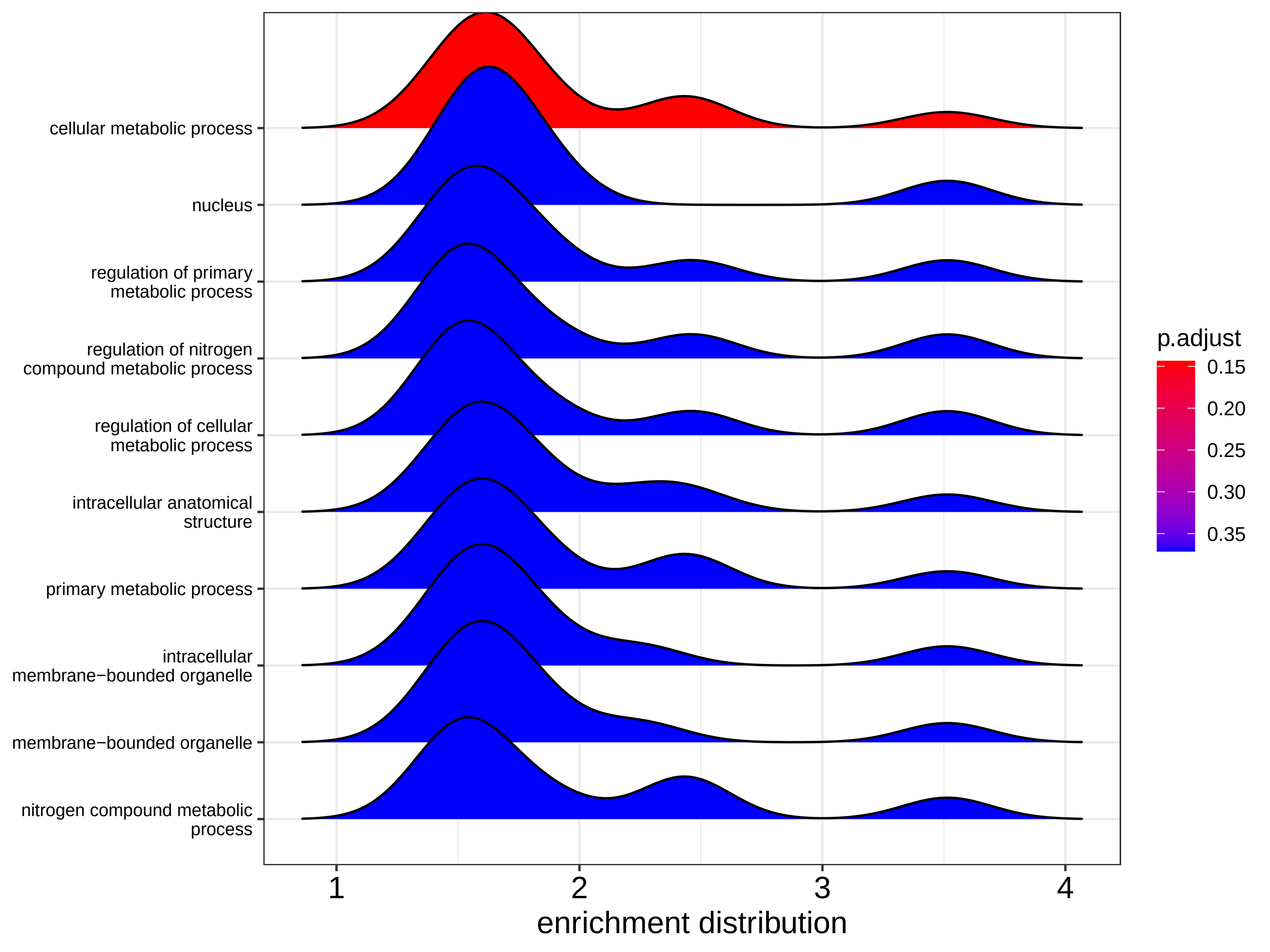
cellular metabolic process
nucleus
regulation of primary metabolic process
regulation of nitrogen compound metabolic process
regulation of cellular metabolic process
intracellular anatomical structure
primary metabolic process
intracellular membrane−bounded organelle
membrane−bounded organelle
nitrogen compound metabolic process
1
2
3
4
enrichment distribution
p.adjust
0.15
0.20
0.25
0.30
0.35
