# Supplementary material for: Production, Passaging Stability, and Histological Analysis of Madin–Darby Canine Kidney Cells Cultured in a Low-Serum Medium
Source: Vaccines (Basel). 2024 Aug 30;12(9):991. doi: 10.3390/vaccines12090991 (PMC11435615; doi:10.3390/vaccines12090991)
Supplement: Supplementary file 1 [file vaccines-12-00991-s001.zip › Supplementary File S3/transcriptome/5.Enrichment/gsea/3-infected_vs_5-infected/3-infected_vs_5-infected_GO_BP_GSEA_ridgeplot.pdf]

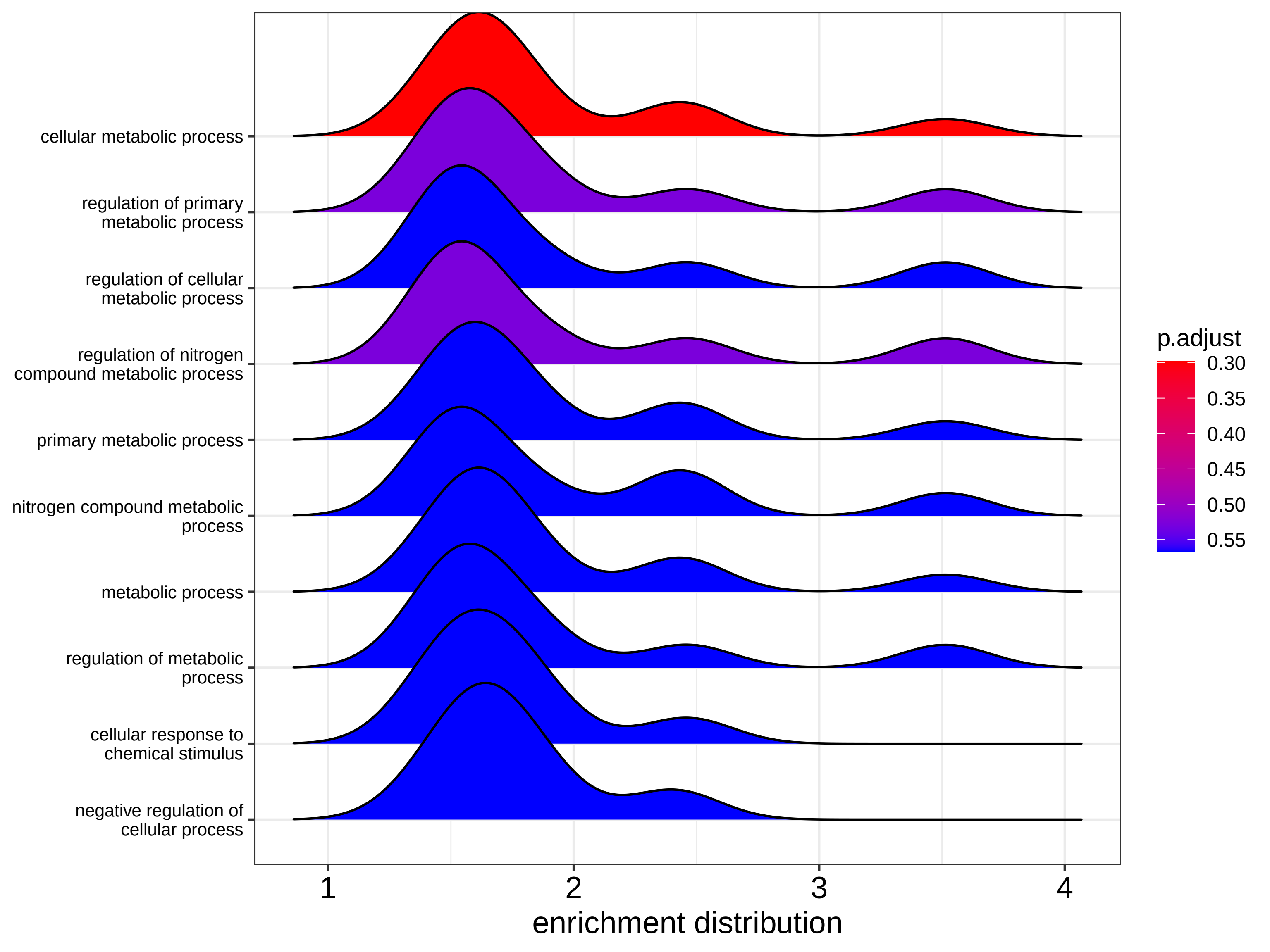

cellular metabolic process
regulation of primary metabolic process
regulation of cellular metabolic process
regulation of nitrogen compound metabolic process
primary metabolic process
nitrogen compound metabolic process
metabolic process
regulation of metabolic process
cellular response to chemical stimulus
negative regulation of cellular process
1
2
3
4
enrichment distribution
p.adjust
0.30
0.35
0.40
0.45
0.50
0.55
