# Supplementary material for: Production, Passaging Stability, and Histological Analysis of Madin–Darby Canine Kidney Cells Cultured in a Low-Serum Medium
Source: Vaccines (Basel). 2024 Aug 30;12(9):991. doi: 10.3390/vaccines12090991 (PMC11435615; doi:10.3390/vaccines12090991)
Supplement: Supplementary file 1 [file vaccines-12-00991-s001.zip › Supplementary File S3/transcriptome/5.Enrichment/gsea/3-infected_vs_5-infected/3-infected_vs_5-infected_GO_CC_GSEA_dotplot.pdf]

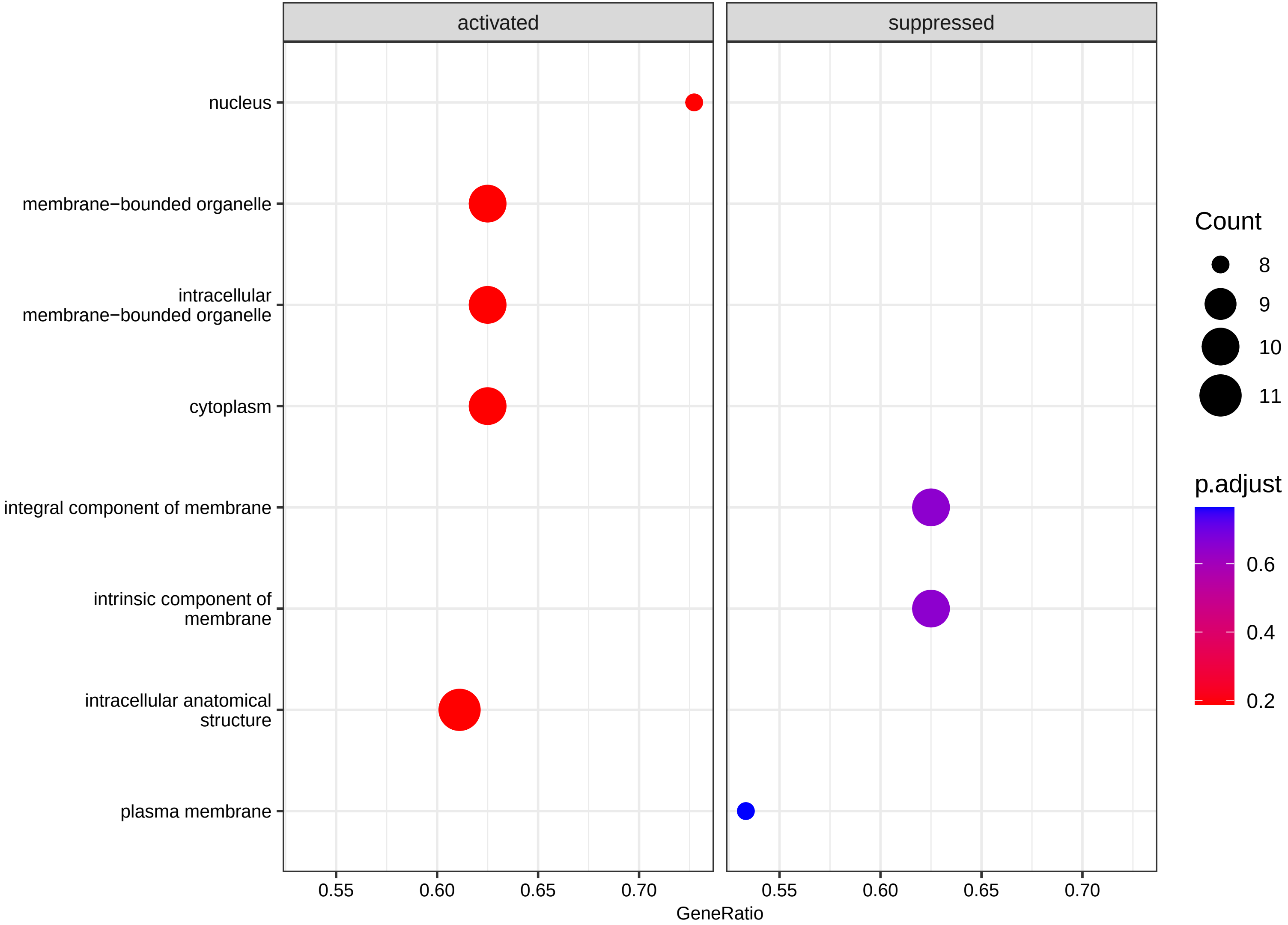

activated
suppressed
nucleus
membrane−bounded organelle
intracellular
membrane−bounded organelle
cytoplasm
integral component of membrane
intrinsic component of
membrane
intracellular anatomical
structure
plasma membrane
0.55
0.60
0.65
0.70
GeneRatio
Count
8
9
10
11
p.adjust
0.6
0.4
0.2
