# Supplementary material for: Production, Passaging Stability, and Histological Analysis of Madin–Darby Canine Kidney Cells Cultured in a Low-Serum Medium
Source: Vaccines (Basel). 2024 Aug 30;12(9):991. doi: 10.3390/vaccines12090991 (PMC11435615; doi:10.3390/vaccines12090991)
Supplement: Supplementary file 1 [file vaccines-12-00991-s001.zip › Supplementary File S3/transcriptome/5.Enrichment/gsea/3-infected_vs_5-infected/3-infected_vs_5-infected_GO_MF_GSEA_dotplot.pdf]

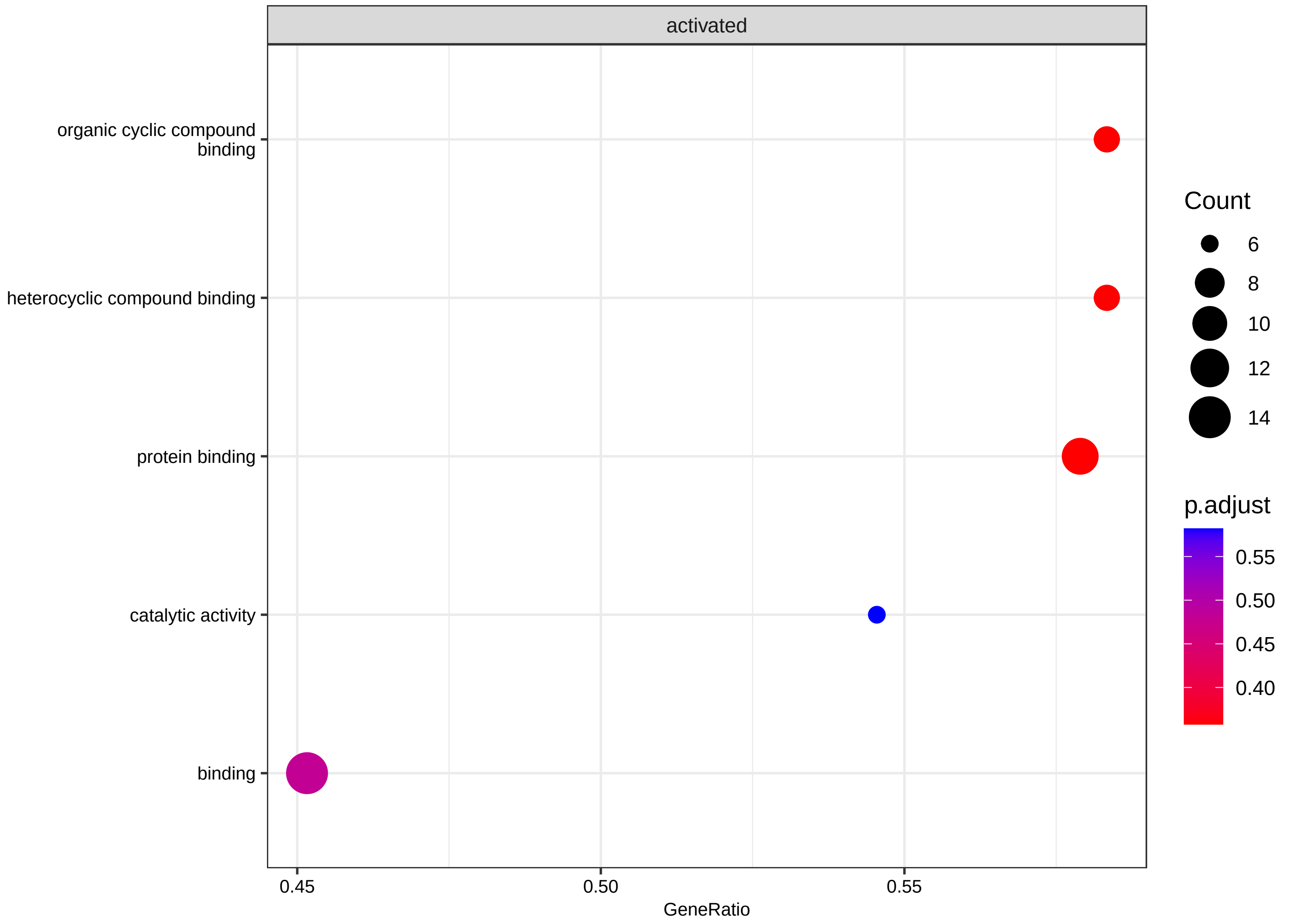

activated
organic cyclic compound binding
heterocyclic compound binding
protein binding
catalytic activity
binding
0.45
0.50
0.55
GeneRatio
Count
6
8
10
12
14
p.adjust
0.55
0.50
0.45
0.40
