# Supplementary material for: Production, Passaging Stability, and Histological Analysis of Madin–Darby Canine Kidney Cells Cultured in a Low-Serum Medium
Source: Vaccines (Basel). 2024 Aug 30;12(9):991. doi: 10.3390/vaccines12090991 (PMC11435615; doi:10.3390/vaccines12090991)
Supplement: Supplementary file 1 [file vaccines-12-00991-s001.zip › Supplementary File S3/transcriptome/5.Enrichment/gsea/3-infected_vs_5-infected/3-infected_vs_5-infected_KEGG_GSEA_ridgeplot.pdf]

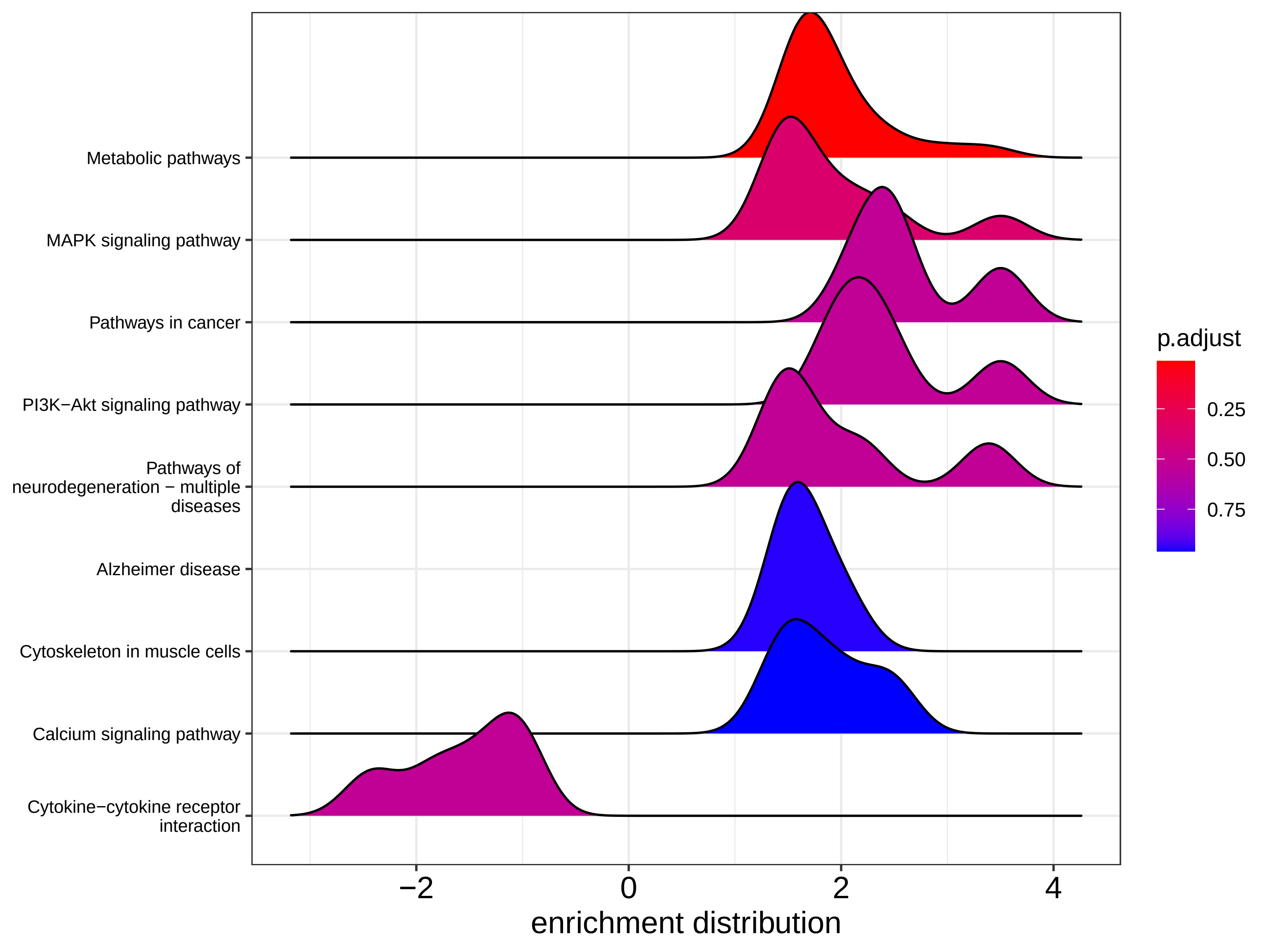

Metabolic pathways
MAPK signaling pathway
Pathways in cancer
PI3K−Akt signaling pathway
Pathways of neurodegeneration − multiple diseases
Alzheimer disease
Cytoskeleton in muscle cells
Calcium signaling pathway
Cytokine−cytokine receptor interaction
−2
0
2
4
enrichment distribution
p.adjust
0.25
0.50
0.75
