# Supplementary material for: Production, Passaging Stability, and Histological Analysis of Madin–Darby Canine Kidney Cells Cultured in a Low-Serum Medium
Source: Vaccines (Basel). 2024 Aug 30;12(9):991. doi: 10.3390/vaccines12090991 (PMC11435615; doi:10.3390/vaccines12090991)
Supplement: Supplementary file 1 [file vaccines-12-00991-s001.zip › Supplementary File S3/transcriptome/5.Enrichment/gsea/3-uninfected_vs_5-uninfected/3-uninfected_vs_5-uninfected_GO_ALL_GSEA_dotplot.pdf]

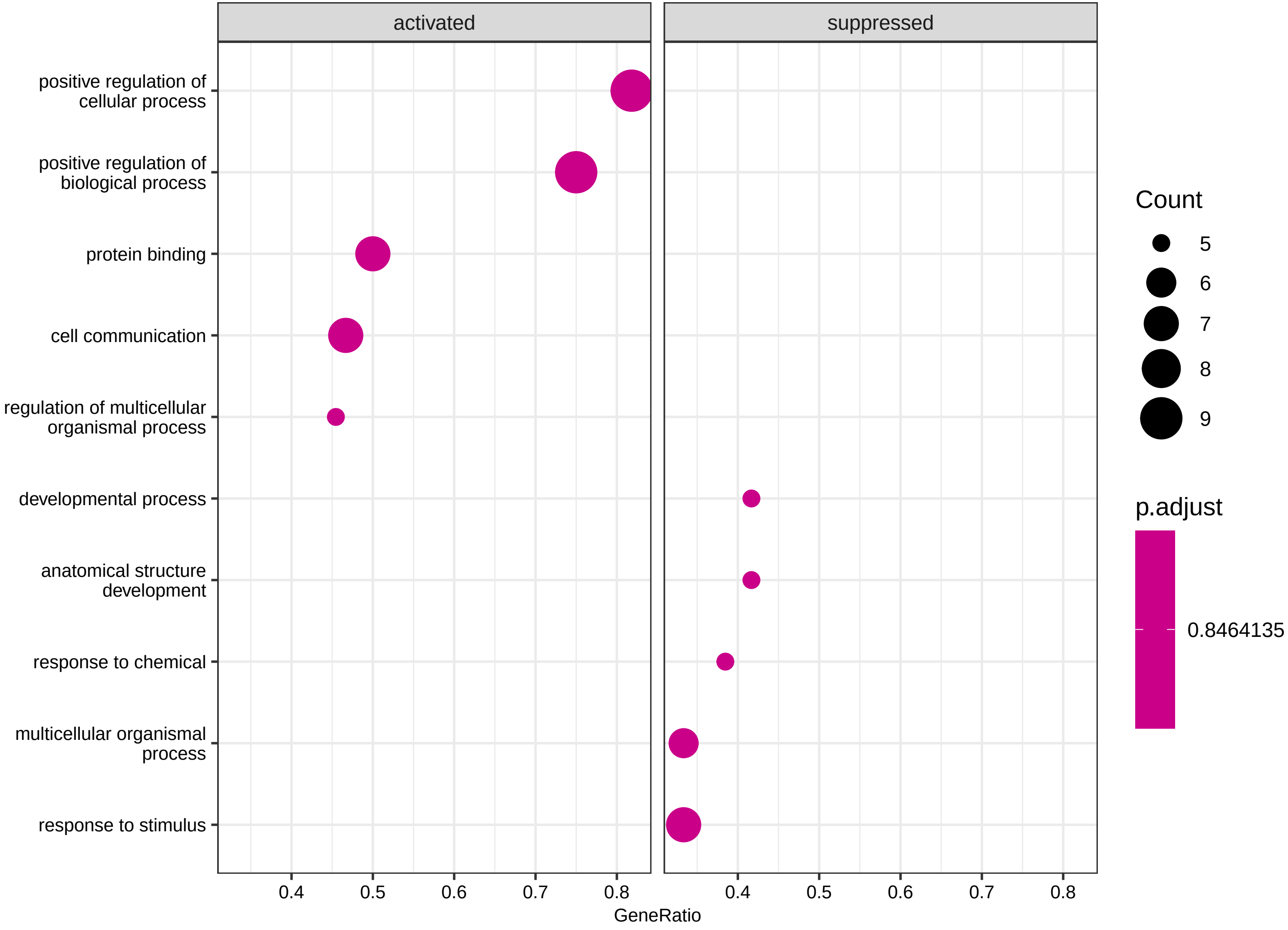
activated
suppressed
positive regulation of cellular process
positive regulation of biological process
protein binding
cell communication
regulation of multicellular organismal process
developmental process
anatomical structure development
response to chemical
multicellular organismal process
response to stimulus
0.4
0.5
0.6
0.7
0.8
GeneRatio
Count
5
6
7
8
9
p.adjust
0.8464135
