# Supplementary material for: Production, Passaging Stability, and Histological Analysis of Madin–Darby Canine Kidney Cells Cultured in a Low-Serum Medium
Source: Vaccines (Basel). 2024 Aug 30;12(9):991. doi: 10.3390/vaccines12090991 (PMC11435615; doi:10.3390/vaccines12090991)
Supplement: Supplementary file 1 [file vaccines-12-00991-s001.zip › Supplementary File S3/transcriptome/5.Enrichment/gsea/3-uninfected_vs_5-uninfected/3-uninfected_vs_5-uninfected_GO_BP_GSEA_dotplot.pdf]

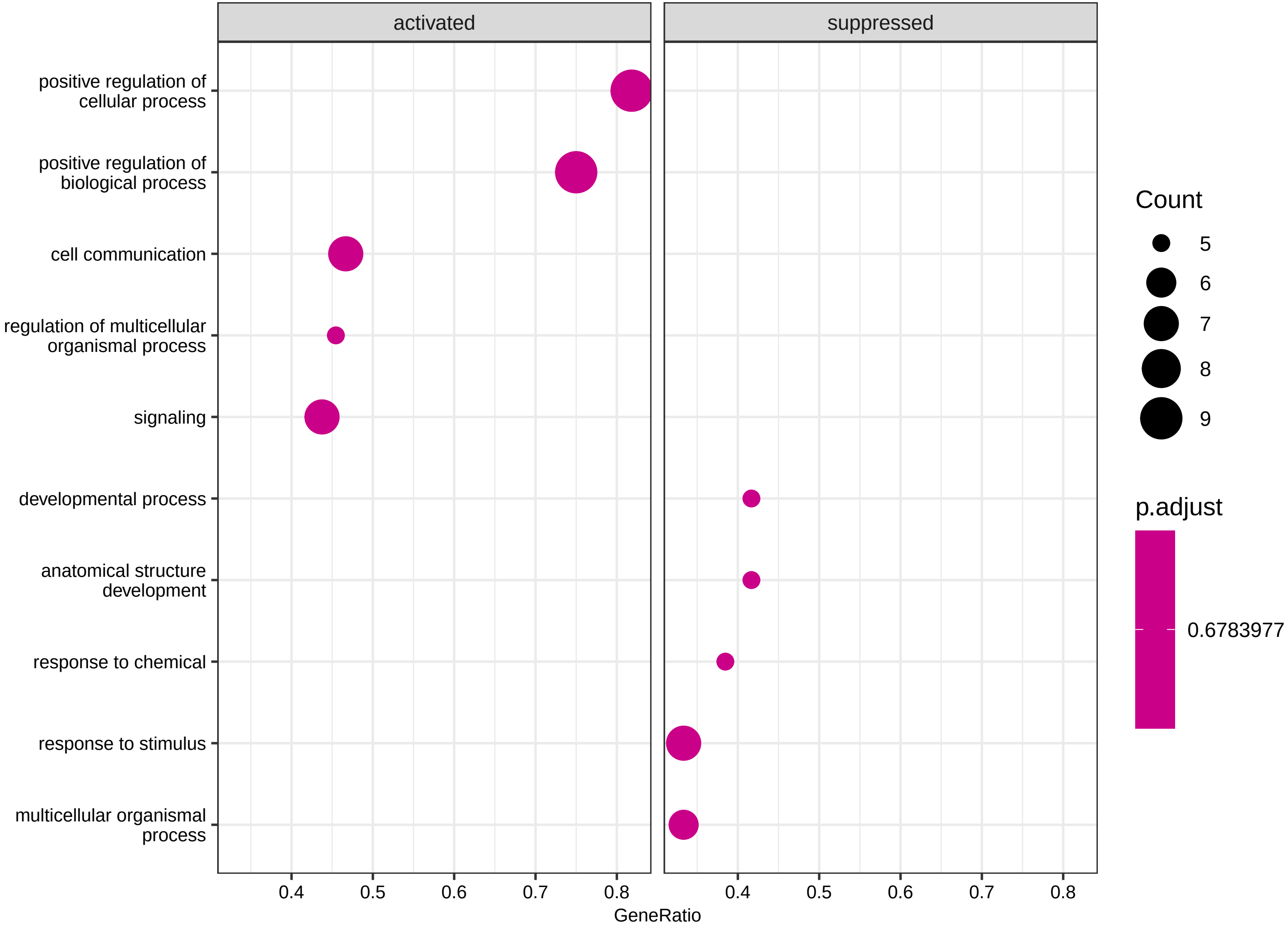
activated
suppressed
positive regulation of cellular process
positive regulation of biological process
cell communication
regulation of multicellular organismal process
signaling
developmental process
anatomical structure development
response to chemical
response to stimulus
multicellular organismal process
0.4
0.5
0.6
0.7
0.8
GeneRatio
Count
5
6
7
8
9
p.adjust
0.6783977
