# Supplementary material for: Production, Passaging Stability, and Histological Analysis of Madin–Darby Canine Kidney Cells Cultured in a Low-Serum Medium
Source: Vaccines (Basel). 2024 Aug 30;12(9):991. doi: 10.3390/vaccines12090991 (PMC11435615; doi:10.3390/vaccines12090991)
Supplement: Supplementary file 1 [file vaccines-12-00991-s001.zip › Supplementary File S3/transcriptome/5.Enrichment/gsea/3-uninfected_vs_5-uninfected/3-uninfected_vs_5-uninfected_GO_BP_GSEA_ridgeplot.pdf]

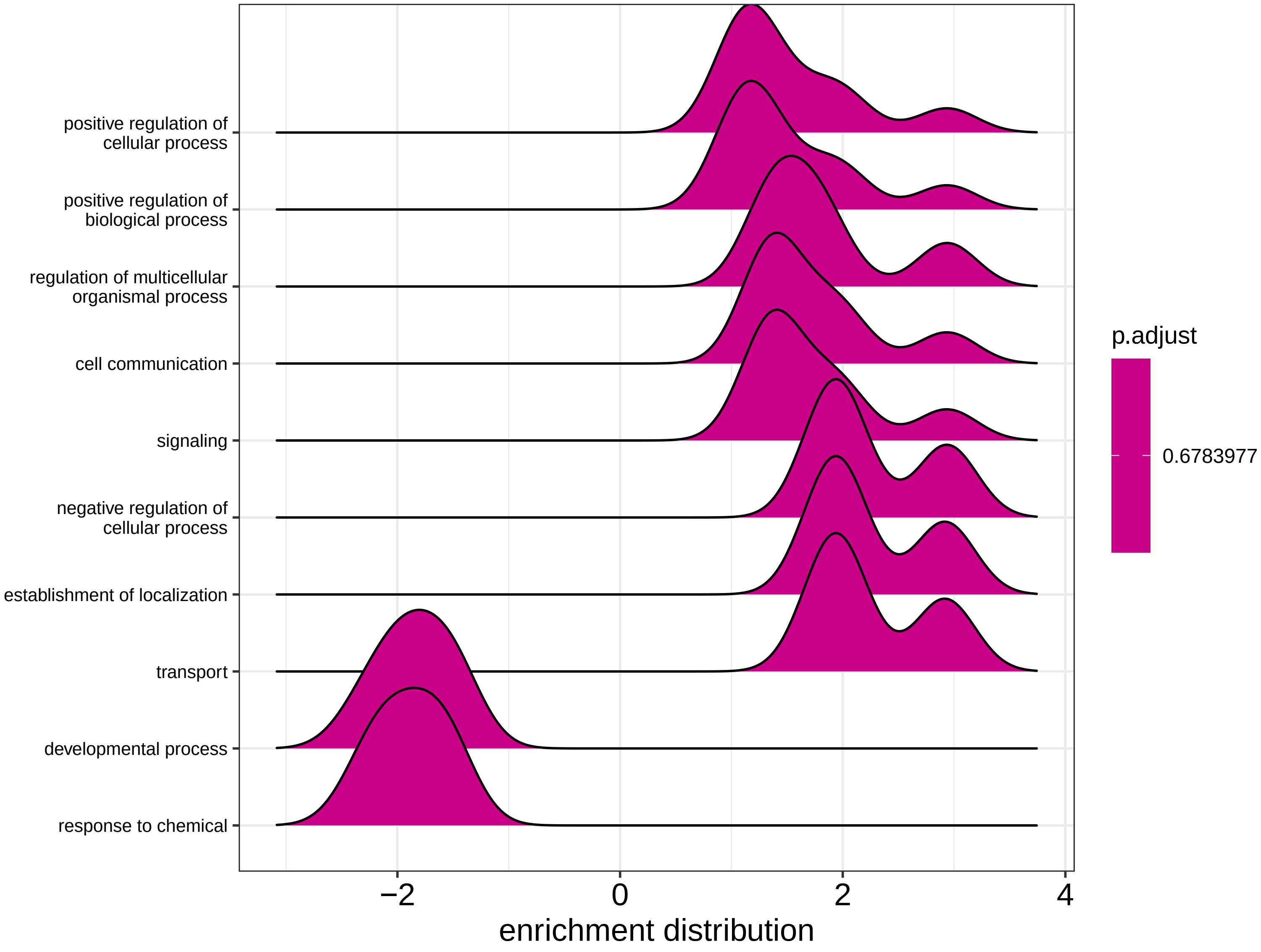
positive regulation of cellular process
positive regulation of biological process
regulation of multicellular organismal process
cell communication
signaling
negative regulation of cellular process
establishment of localization
transport
developmental process
response to chemical
−2
0
2
4
enrichment distribution
p.adjust
0.6783977
