# Supplementary material for: Production, Passaging Stability, and Histological Analysis of Madin–Darby Canine Kidney Cells Cultured in a Low-Serum Medium
Source: Vaccines (Basel). 2024 Aug 30;12(9):991. doi: 10.3390/vaccines12090991 (PMC11435615; doi:10.3390/vaccines12090991)
Supplement: Supplementary file 1 [file vaccines-12-00991-s001.zip › Supplementary File S3/transcriptome/5.Enrichment/gsea/3-uninfected_vs_5-uninfected/3-uninfected_vs_5-uninfected_GO_CC_GSEA_dotplot.pdf]

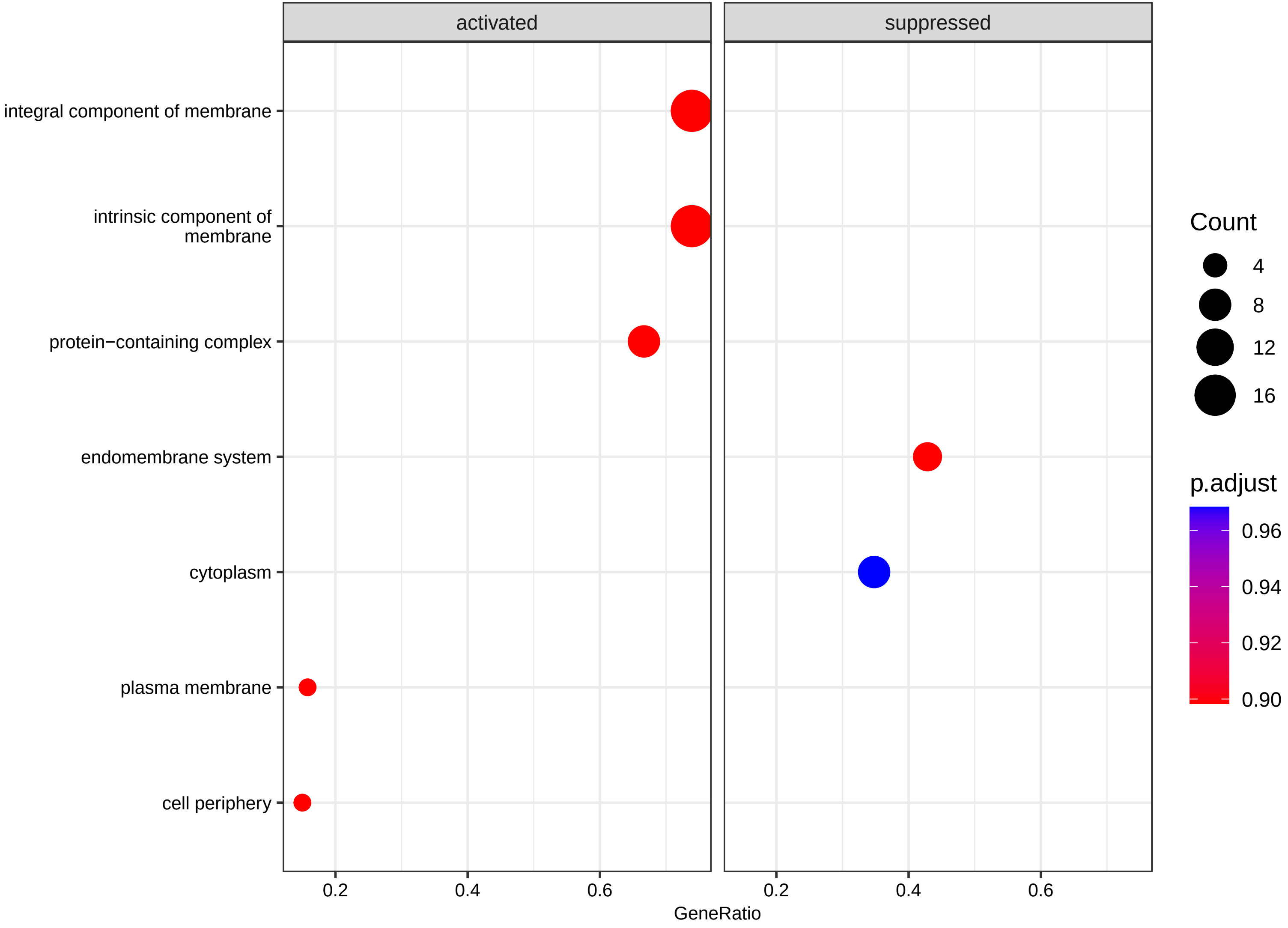

activated
suppressed
integral component of membrane
intrinsic component of membrane
protein−containing complex
endomembrane system
cytoplasm
plasma membrane
cell periphery
0.2
0.4
0.6
GeneRatio
Count
4
8
12
16
p.adjust
0.96
0.94
0.92
0.90
