# Supplementary material for: Production, Passaging Stability, and Histological Analysis of Madin–Darby Canine Kidney Cells Cultured in a Low-Serum Medium
Source: Vaccines (Basel). 2024 Aug 30;12(9):991. doi: 10.3390/vaccines12090991 (PMC11435615; doi:10.3390/vaccines12090991)
Supplement: Supplementary file 1 [file vaccines-12-00991-s001.zip › Supplementary File S3/transcriptome/5.Enrichment/gsea/3-uninfected_vs_5-uninfected/3-uninfected_vs_5-uninfected_GO_CC_GSEA_ridgeplot.pdf]

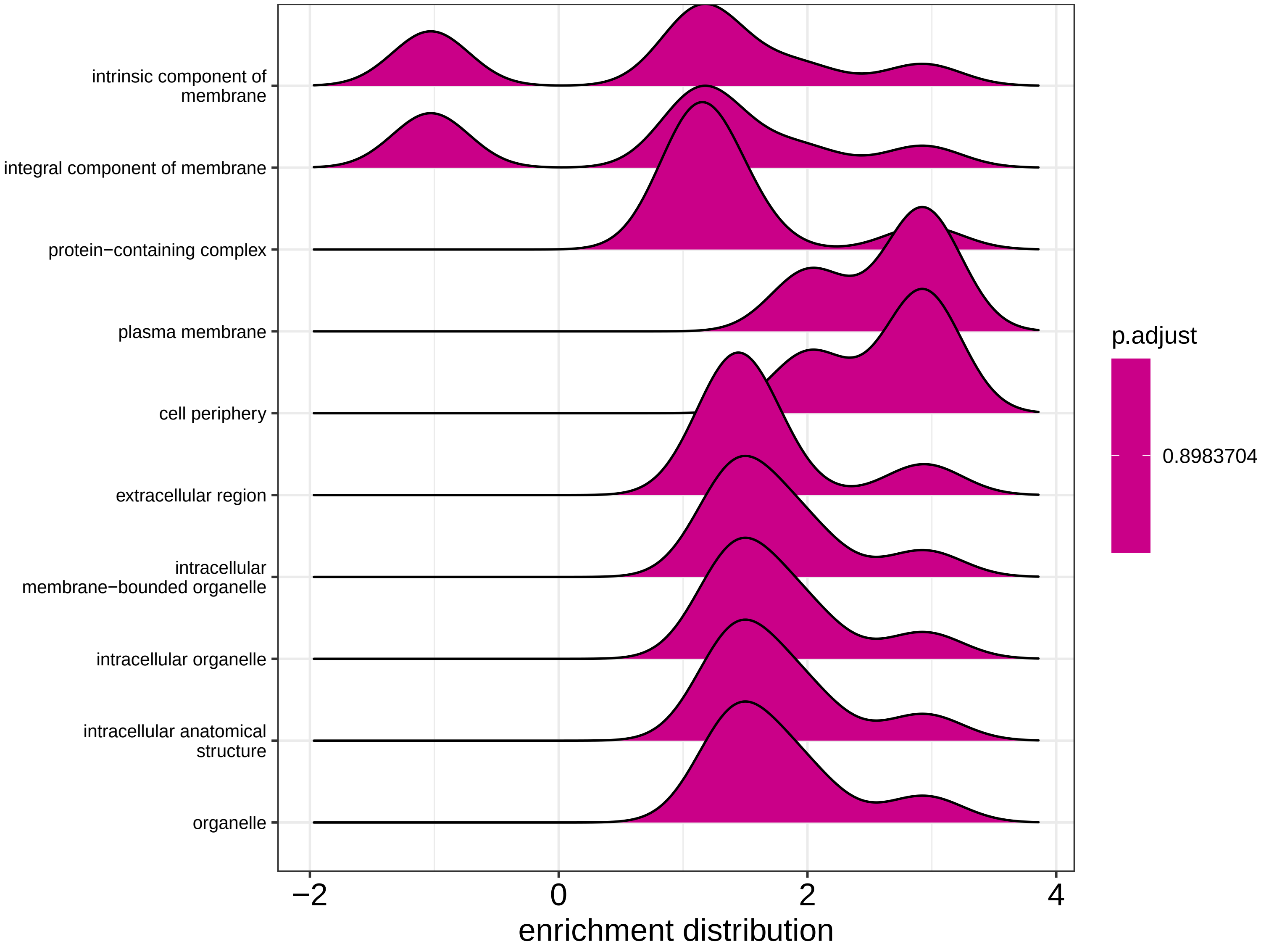

intrinsic component of membrane
integral component of membrane
protein−containing complex
plasma membrane
cell periphery
extracellular region
intracellular membrane−bounded organelle
intracellular organelle
intracellular anatomical structure
organelle
−2
0
2
4
enrichment distribution
p.adjust
0.8983704
