# Supplementary material for: Production, Passaging Stability, and Histological Analysis of Madin–Darby Canine Kidney Cells Cultured in a Low-Serum Medium
Source: Vaccines (Basel). 2024 Aug 30;12(9):991. doi: 10.3390/vaccines12090991 (PMC11435615; doi:10.3390/vaccines12090991)
Supplement: Supplementary file 1 [file vaccines-12-00991-s001.zip › Supplementary File S3/transcriptome/5.Enrichment/gsea/3-uninfected_vs_5-uninfected/3-uninfected_vs_5-uninfected_GO_MF_GSEA_dotplot.pdf]

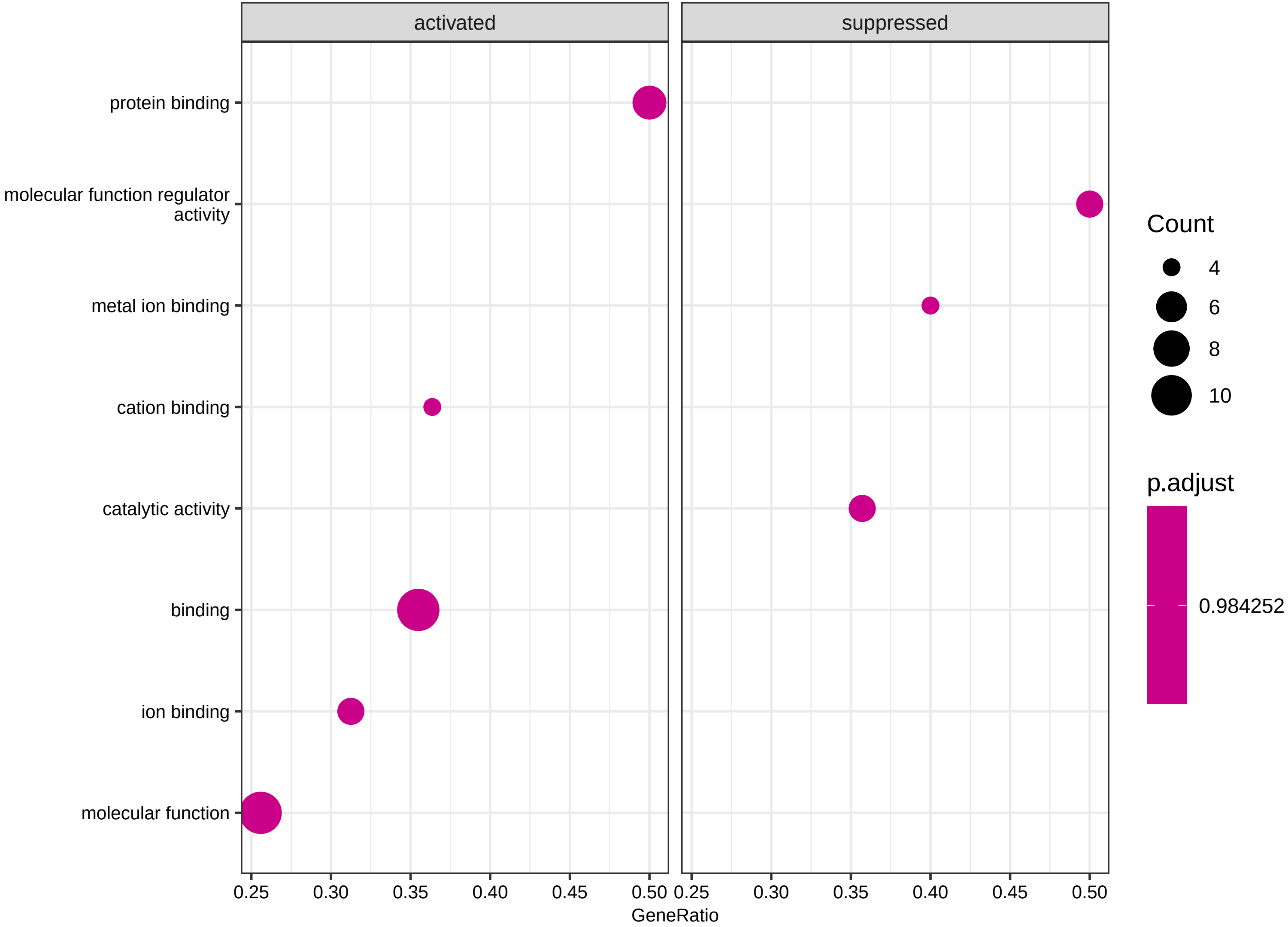

activated
suppressed
protein binding
molecular function regulator activity
metal ion binding
cation binding
catalytic activity
binding
ion binding
molecular function
0.25
0.30
0.35
0.40
0.45
0.50
GeneRatio
Count
4
6
8
10
p.adjust
0.984252
