# Supplementary material for: Production, Passaging Stability, and Histological Analysis of Madin–Darby Canine Kidney Cells Cultured in a Low-Serum Medium
Source: Vaccines (Basel). 2024 Aug 30;12(9):991. doi: 10.3390/vaccines12090991 (PMC11435615; doi:10.3390/vaccines12090991)
Supplement: Supplementary file 1 [file vaccines-12-00991-s001.zip › Supplementary File S3/transcriptome/5.Enrichment/gsea/3-uninfected_vs_5-uninfected/3-uninfected_vs_5-uninfected_KEGG_GSEA_dotplot.pdf]

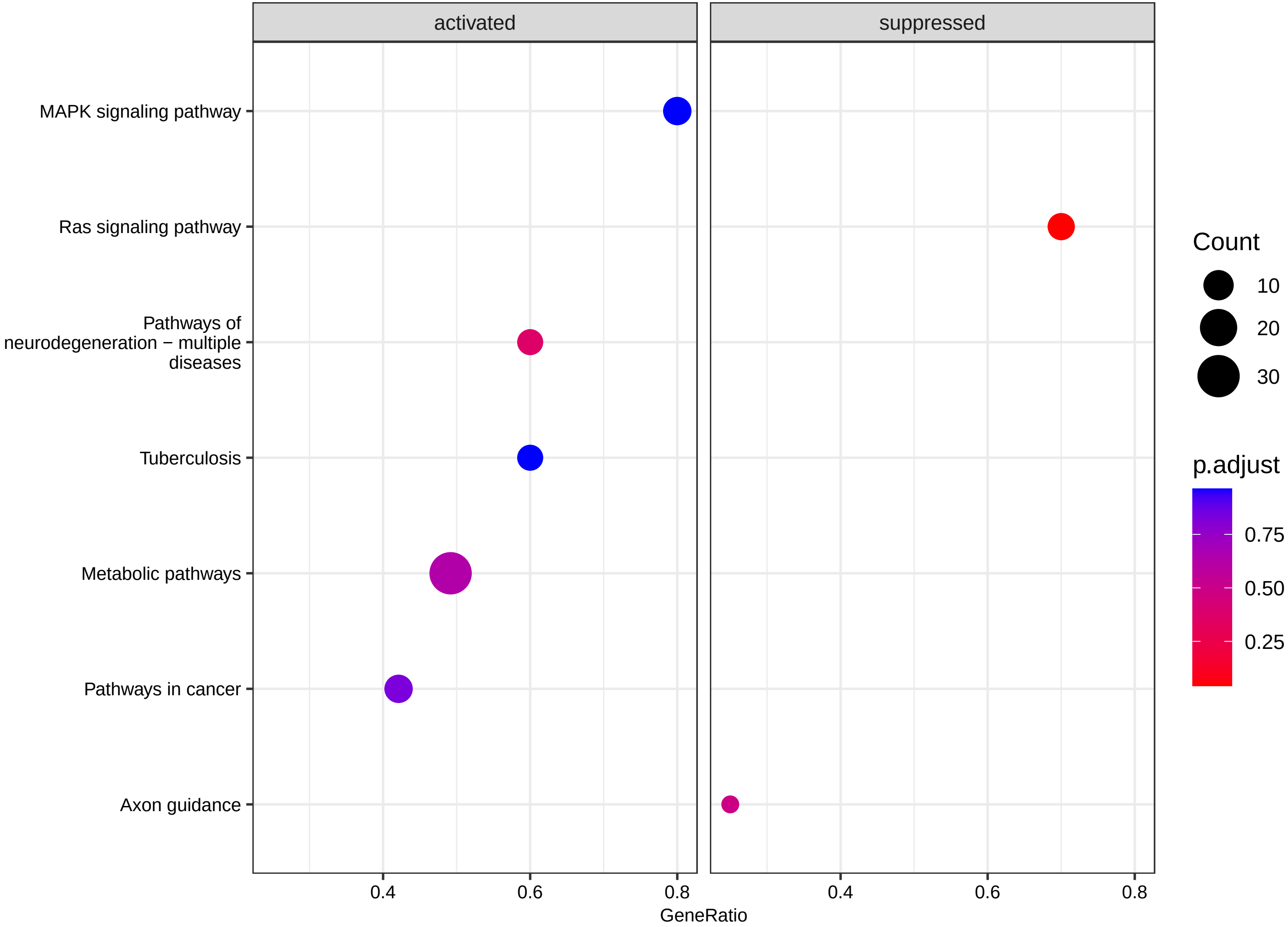
activated
suppressed
MAPK signaling pathway
Ras signaling pathway
Pathways of neurodegeneration − multiple diseases
Tuberculosis
Metabolic pathways
Pathways in cancer
Axon guidance
0.4
0.6
0.8
0.4
0.6
0.8
GeneRatio
Count
10
20
30
p.adjust
0.75
0.50
0.25
