# Supplementary material for: Production, Passaging Stability, and Histological Analysis of Madin–Darby Canine Kidney Cells Cultured in a Low-Serum Medium
Source: Vaccines (Basel). 2024 Aug 30;12(9):991. doi: 10.3390/vaccines12090991 (PMC11435615; doi:10.3390/vaccines12090991)
Supplement: Supplementary file 1 [file vaccines-12-00991-s001.zip › Supplementary File S3/transcriptome/5.Enrichment/gsea/3-uninfected_vs_5-uninfected/3-uninfected_vs_5-uninfected_KEGG_GSEA_ridgeplot.pdf]

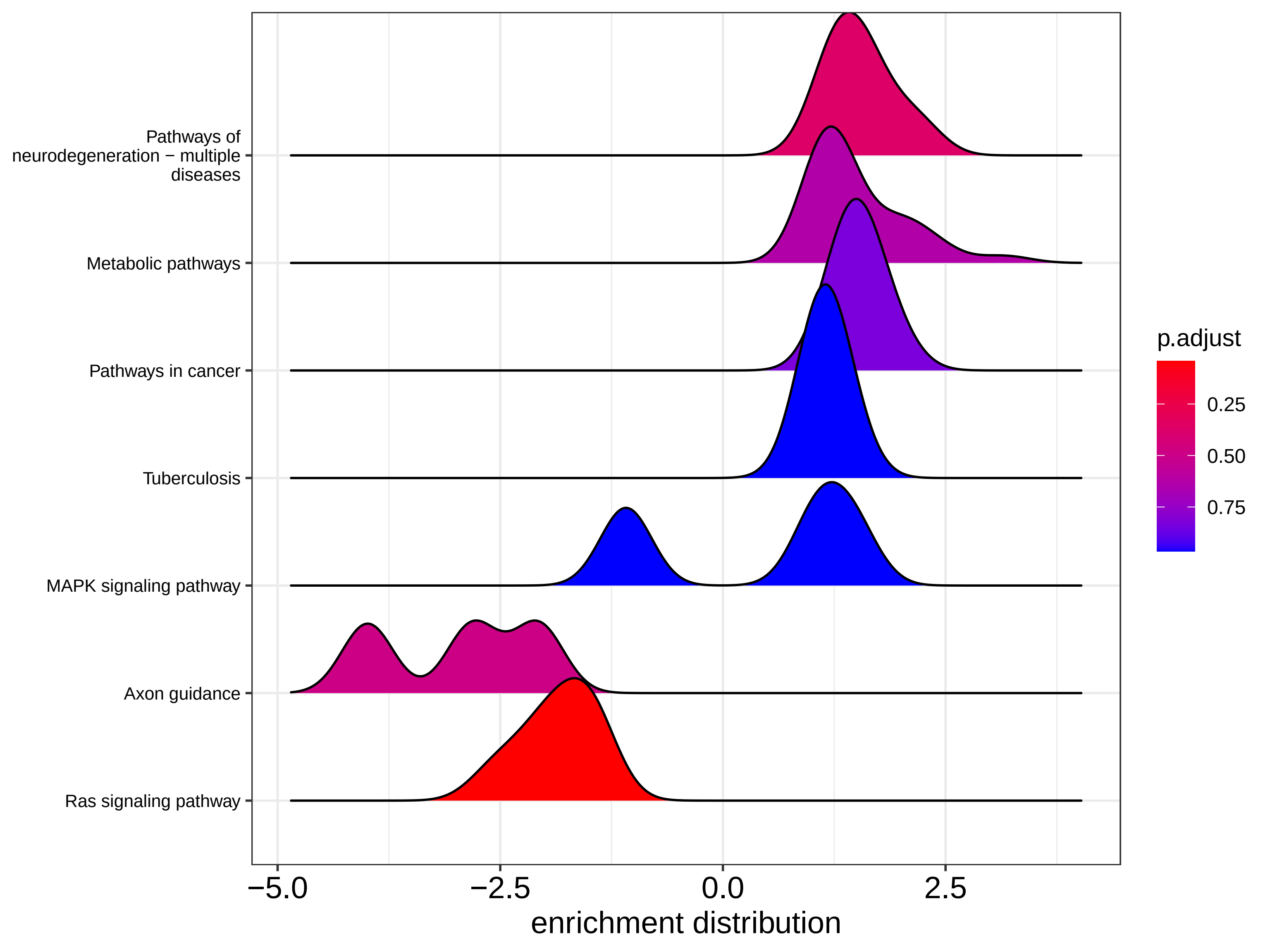
Pathways of neurodegeneration − multiple diseases
Metabolic pathways
Pathways in cancer
Tuberculosis
MAPK signaling pathway
Axon guidance
Ras signaling pathway
−5.0
−2.5
0.0
2.5
enrichment distribution
p.adjust
0.25
0.50
0.75
